# Supplementary material for: CDK12/CDK13 inhibition disrupts transcriptional elongation and replication fork progression in glioblastoma
Source: EMBO Mol Med. 2026 Mar 25;18(5):1592–624. doi: 10.1038/s44321-026-00393-w (PMC13179391; doi:10.1038/s44321-026-00393-w)
Supplement: Supplementary file 4 — Appendix [file 44321_2026_393_MOESM4_ESM.pdf]

# Appendix to *CDK12/13 inhibition disrupts transcriptional elongation and fork progression in glioblastoma*

**This document contains:**

**Appendix Figures** .......... **2**

Appendix Figure S1 .......... 2

Appendix Figure S2 .......... 3

**Appendix Tables** .......... **4**

Appendix Table S1: Characteristics of the patients included in the IHC .......... 4

Appendix Table S2: Brain concentrations of SR-4835 in tolerability studies .......... 4

Appendix Table S3: Antibody dilution .......... 5

Appendix Table S4: P-values .......... 5

# Appendix Figures

## Appendix Figure S1

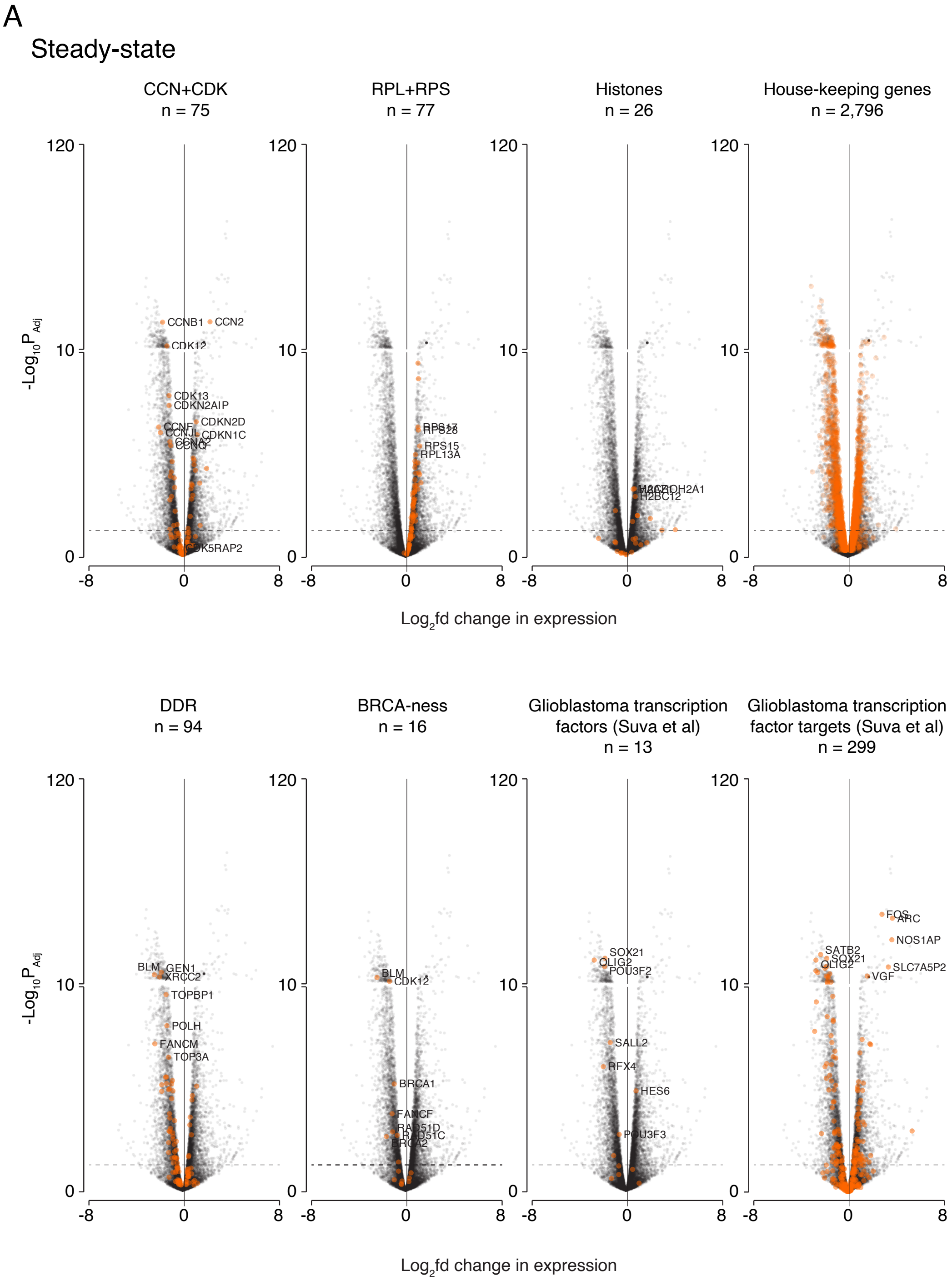


(**A**) Volcano plots showing the overall transcriptional differences in steady-state transcripts as in Figure 3D, but with certain gene populations highlighted. X-axes show the log2 fold difference in transcription in G7 cells treated with THZ531 for 6 h compared to DMSO controls. Y-axes show the -log10 transformed p-values Benjamini-Hochberg corrected for multiple testing. Colored dots illustrate the transcriptional changes of the listed gene populations.

## Appendix Figure S2

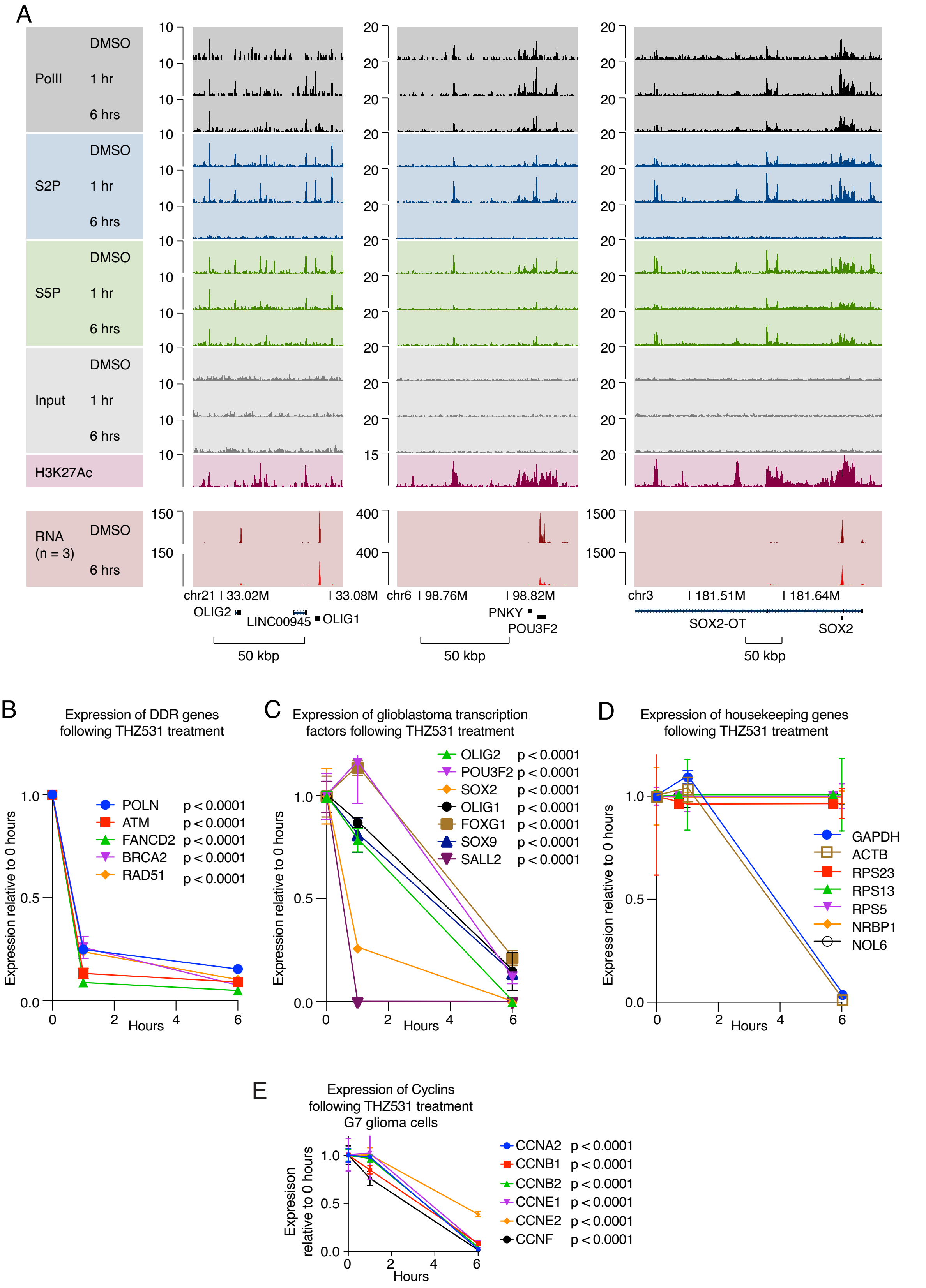


(**A**) Genome browser tracks of Cut&Run signal density and total transcript-levels (bottom) at the Olig2, Pou3f2 and Sox2 loci at indicated treatments. (**B**)-(**E**) RT-qPCR analyses of the mRNA levels of DDR genes (**B**), glioblastoma transcription factors (**C**), housekeeping genes

(**D**) or cell cycle regulators (**E**) in G7 cells treated with 500 nM THZ531 for 1 h and 6 h. Data represent mean ± SD of two replicates. Data were analyzed by two-way ANOVA followed by Tukey's multiple comparisons test, significant differences as compared to the starting point (0h).

## Appendix Tables

### Appendix Table S1: Characteristics of the patients included in the IHC

| Patient No. | Slide No. | Age (y) | Sex | Tissue | Diagnosis |
|---|---|---|---|---|---|
| 1 | C12 | 81 | Female | Fascia | Fasciitis |
| 2 | C18 | 40 | Male | CNS | Cavernous hemangioma |
| 3 | C15 | 74 | Female | CNS | Cerebral amyloid angiopathy (CAA) |
| 4 | C14 | 59 | Female | CNS | Glioblastoma, CNS WHO grade 4 |
| 5 | C16 | 85 | Female | CNS | Glioblastoma, CNS WHO grade 4 |
| 6 | C17 | 69 | Female | CNS | Glioblastoma, CNS WHO grade 4 |
| 7 | C19 | 64 | Male | CNS | Glioblastoma, CNS WHO grade 4 |
| 8 | C21 | 55 | Male | CNS | Recurrent Glioblastoma, CNS WHO grade 4 |

### Appendix Table S2: Brain concentrations of SR-4835 in tolerability studies

| Dose (mg/kg) | Regimen | Sample ID | Brain Concentration (ng/mL) | Detection |
|---|---|---|---|---|
| 20 | Daily | #1/1 | <LLOQ | Not detected |
| 20 | Daily | #2/1 | <LLOQ | Not detected |
| 20 | Daily | #3/1 | <LLOQ | Not detected |
| 30 | Daily | #4/2 | <LLOQ | Not detected |
| 30 | Daily | #5/2 | 7.6 | Detected (low) |
| 30 | Daily | #6/2 | <LLOQ | Not detected |
| 30 | Alternate days | #7/3 | <LLOQ | Not detected |
| 30 | Alternate days | #8/3 | <LLOQ | Not detected |
| 30 | Alternate days | #9/3 | <LLOQ | Not detected |

Lower Limit of Quantification. LLOQ = 1.00 ng/mL

**Appendix Table S3: Antibody dilution**

| Antibody | Manufacturer | Catalog number | Dilution for CUT&RUN | Dilution for WB | Dilution for IF |
|---|---|---|---|---|---|
| RNAPII total | MBL | MABI0601 | 1:100 | 1:10 000 | |
| RNAPII pSer2 | MBL | MABI0602 | 1:100 | 1:10 000 | |
| RNAPII pSer5 | MBL | MABI0603 | 1:100 | 1:10 000 | |
| H3K27ac | CST | D5E4 | 1:100 | 1:1000 | |
| H3K27me3 | CST | C36B11 | 1:100 | 1:1000 | |
| RNAPII pThr4 | Active Motif | 61461 | NA | 1:1000 | |
| RNAPII pSer7 | Active Motif | 61087 | NA | 1:1000 | |
| Vinculin | Sigma | V9131 | NA | 1:10 000 | |
| PARP | CST | 9542 | NA | 1:1000 | |
| GAPDH | Santa Cruz Biotechnology | sc25778 | NA | 1:20 000 | |
| IgG | Abcam | ab6721 | 1:100 | NA | |
| RFP | BioNordika (Rockland) | 600-401-379 | | | 1:200 |

**Appendix Table S4: P-values**

| Figure 1C | | |
|---|---|---|
| Two-way ANOVA | | |
| **Tukey's multiple comparisons test** | **Summary** | **P Value** |
| | | |
| **Cell type: G7** | | |
| 16 days | | |
| DMSO vs. 100nM THZ531 | *** | 0,000807565680004 |
| DMSO vs. 500nM THZ531 | *** | 0,000806253394743 |

| | | |
|---|---|---|
| 100nM THZ531 vs. 500nM THZ531 | ns | 0,999999617121843 |

**Cell type: G144**

| | | |
|---|---|---|
| 16 days | | |
| DMSO vs. 100nM THZ531 | **** | <0,000000000000001 |
| DMSO vs. 500nM THZ531 | **** | <0,000000000000001 |
| 100nM THZ531 vs. 500nM THZ531 | ns | 0,997777369708783 |

**Cell type: Hela**

| | | |
|---|---|---|
| 14 days | | |
| DMSO vs. 100 nM THZ531 | **** | 0,000000467842452 |
| DMSO vs. 500 nM THZ531 | **** | 0,000000000400941 |
| 100 nM THZ531 vs. 500 nM THZ531 | **** | <0,000000000000001 |

**Figure 1G**

**Two-way ANOVA**

| Tukey's multiple comparisons test | Summary | P Value |
|---|---|---|
| **NC** | | |
| day 0 vs. day 7 | ns | 0,999976191524814 |
| day 0 vs. day 14 | ns | 0,970651962495543 |
| day 0 vs. day 21 | ns | 0,983512179424494 |
| **sg_MCM2** | | |
| day 0 vs. day 7 | ns | 0,621972681697333 |
| day 0 vs. day 14 | **** | 0,000001724614945 |
| day 0 vs. day 21 | **** | 0,000000003441056 |
| **sg_RPS19** | | |
| day 0 vs. day 7 | * | 0,032946463771858 |
| day 0 vs. day 14 | **** | 0,00000885195386 |
| day 0 vs. day 21 | **** | 0,000000906224435 |
| **sg1_CDK9** | | |
| day 0 vs. day 7 | ns | 0,093696896150888 |
| day 0 vs. day 14 | **** | 0,000022282175578 |
| day 0 vs. day 21 | **** | 0,000000152732645 |
| **sg2_CDK9** | | |
| day 0 vs. day 7 | ns | 0,781276693650005 |
| day 0 vs. day 14 | ns | 0,420027399424275 |
| day 0 vs. day 21 | * | 0,048822205674024 |

| | | |
|---|---|---|
| **sg3_CDK9** | | |
| day 0 vs. day 7 | * | 0,01495519069165 |
| day 0 vs. day 14 | *** | 0,000221124721562 |
| day 0 vs. day 21 | **** | 0,000018827665707 |
| | | |
| **sg1_CDK12** | | |
| day 0 vs. day 7 | ns | 0,118741854461276 |
| day 0 vs. day 14 | * | 0,014955190691651 |
| day 0 vs. day 21 | ** | 0,002497995965609 |
| | | |
| **sg2_CDK12** | | |
| day 0 vs. day 7 | ns | 0,075425742134187 |
| day 0 vs. day 14 | * | 0,011873203341995 |
| day 0 vs. day 21 | ** | 0,003220572882048 |
| | | |
| **sg3_CDK12** | | |
| day 0 vs. day 7 | ns | 0,327886559841536 |
| day 0 vs. day 14 | ns | 0,226308696537047 |
| day 0 vs. day 21 | ** | 0,004904037932036 |
| | | |
| **sg1_CDK13** | | |
| day 0 vs. day 7 | ns | 0,139901702807128 |
| day 0 vs. day 14 | * | 0,011444028413037 |
| day 0 vs. day 21 | *** | 0,00057409884113 |
| | | |
| **sg2_CDK13** | | |
| day 0 vs. day 7 | * | 0,034448799757469 |
| day 0 vs. day 14 | ** | 0,002272987932976 |
| day 0 vs. day 21 | **** | 0,000038563662239 |
| | | |
| **sg3_CDK13** | | |
| day 0 vs. day 7 | ns | 0,074672056266458 |
| day 0 vs. day 14 | ns | 0,451311004268309 |
| day 0 vs. day 21 | **** | 0,000000387327652 |

**Figure 3A**

Independent two-sample t-test (SciPy library)

| | **Summary** | **P value** |
|---|---|---|
| **G7** | | |
| control-gefitinib | ** | 0,0035407 |
| control-THZ531 | **** | 0,000000003146 |

| | | |
|---|---|---|
| Gefitinib-THZ531 | **** | 0,0000021645 |
| **G144** | | |
| control-gefitinib | **** | 0,000098938 |
| control-THZ531 | **** | 0,000000005489 |
| Gefitinib-THZ531 | **** | 0,0000011153 |
| **G14** | | |
| control-gefitinib | ** | 0,0032866 |
| control-THZ531 | *** | 0,0002912 |
| Gefitinib-THZ531 | ns | 0,10051 |
| **G166** | | |
| control-gefitinib | * | 0,013192 |
| control-THZ531 | **** | 0,0000031442 |
| Gefitinib-THZ531 | *** | 0,00029259 |

**Figure EV1D**

**Two-way ANOVA**

| Tukey's multiple comparisons test | Summary | P Value |
|---|---|---|
| **THZ531** | | |
| Cell line: G14 | | |
| Day 10 | | |
| DMSO vs. 100nM THZ531 | **** | 0,00000127 |
| DMSO vs. 500nM THZ531 | **** | 0,00000019 |
| 100nM THZ531 vs. 500nM THZ531 | ns | 0,192712 |
| Cell line: G166 | | |
| Day 10 | | |
| DMSO vs. 100nM THZ531 | **** | 0,0000104 |
| DMSO vs. 500nM THZ531 | **** | 0,000000466 |
| 100nM THZ531 vs. 500nM THZ531 | * | 0,045061 |
| **SR-4835** | | |
| Cell line: G14 | | |
| Day 10 | | |
| DMSO vs. 100nM SR-4835 | **** | 0,000000290 |
| DMSO vs. 500nM SR-4835 | **** | 0,000000156 |
| 100nM SR-4835 vs. 500nM SR-4835 | ns | 0,797952 |
| Cell line: G144 | | |

| | | |
|---|---|---|
| Day 9 | | |
| DMSO vs. 100nM SR-4835 | **** | 0,000000242 |
| DMSO vs. 500nM SR-4835 | **** | 0,00000024 |
| 100nM SR-4835 vs. 500nM SR-4835 | ns | 0,999972 |
| | | |
| Cell line: G166 | | |
| Day 10 | | |
| DMSO vs. 100nM SR-4835 | **** | 0,000000000000417 |
| DMSO vs. 500nM SR-4835 | **** | 0,000000000000395 |
| 100nM SR-4835 vs. 500nM SR-4835 | ns | 0,957067 |
