# Supplementary material for: CDK12/CDK13 inhibition disrupts transcriptional elongation and replication fork progression in glioblastoma
Source: EMBO Mol Med. 2026 Mar 25;18(5):1592–624. doi: 10.1038/s44321-026-00393-w (PMC13179391; doi:10.1038/s44321-026-00393-w)
Supplement: Supplementary file 8 — Source data Fig. 1 [file 44321_2026_393_MOESM8_ESM.zip › Figure 1/1F/Readme.rtf]

README – Figure 1F (TUNEL Flow Cytometry Assay – G7 Cells)Files included: G7 par II_6h 500 nM_015.fcs, G7 par II_6h DMSO_008.fcs, G7 par II_24h 500 nM_005.fcs, G7 par II_24h DMSO_009.fcsDescription: These files contain the raw flow cytometry data (FCS format) used to generate Figure 1F, which reports the percentage of apoptotic G7 glioma stem cells using a TUNEL assay following THZ531 treatment.Experimental ConditionsEach file corresponds to a single sample:Timepoint	Treatment	File6 h	DMSO (control)	G7 par II_6h DMSO_008.fcs6 h	THZ531 500 nM	G7 par II_6h 500 nM_015.fcs24 h	DMSO (control)	G7 par II_24h DMSO_009.fcs24 h	THZ531 500 nM	G7 par II_24h 500 nM_005.fcs
