# Supplementary material for: CDK12/CDK13 inhibition disrupts transcriptional elongation and replication fork progression in glioblastoma
Source: EMBO Mol Med. 2026 Mar 25;18(5):1592–624. doi: 10.1038/s44321-026-00393-w (PMC13179391; doi:10.1038/s44321-026-00393-w)
Supplement: Supplementary file 8 — Source data Fig. 1 [file 44321_2026_393_MOESM8_ESM.zip › Figure 1/1A/Readme.rtf]

README – Figure 1A (Dose–Response MTT Assays)Data file: 1A_THZ531_dose_response.csvDescriptionThis file contains the raw viability measurements used to generate the dose–response curves for 11 high-grade glioma/GSC lines and 7 non-glioma cell lines treated with THZ531, as shown in Figure 1A.Data Structurelog(Concentration) – Log10-transformed THZ531 concentrations.Replicate columns for each cell line (e.g., G7, G7.1, G7.2; G166, G166.1, G166.2; MCF7, MCF7.1, etc.).Values represent % viability relative to DMSO control.Notes:Semicolon-separated; decimal values use commas (,).Some missing values (NaN) reflect concentrations not measured for all lines.UsageThese data were used to:Plot dose–response curves (mean ± SD).Perform nonlinear curve fitting to compute IC50 values (reported in Fig. 1B).
