# Supplementary material for: CDK12/CDK13 inhibition disrupts transcriptional elongation and replication fork progression in glioblastoma
Source: EMBO Mol Med. 2026 Mar 25;18(5):1592–624. doi: 10.1038/s44321-026-00393-w (PMC13179391; doi:10.1038/s44321-026-00393-w)
Supplement: Supplementary file 8 — Source data Fig. 1 [file 44321_2026_393_MOESM8_ESM.zip › Figure 1/1G/Readme.rtf]

README – Figure 1G (CRISPR Competition Assay in G7 Cells)File: 1G_hGBM_G7.csvDescription: This file contains the raw sgRNA abundance data used to generate Figure 1G, which shows competition dynamics of sgRNAs targeting CDK9, CDK12, CDK13, positive control essential genes (MCM2, RPS19), and a non-targeting control (NC) in G7 glioblastoma stem cells.The data represent percent abundance over time relative to Day 0.Data StructureColumns in the CSV correspond to: Timepoints: "day 0", "day 7", "day 14", "day 21"Replicates: Two columns per timepoint (e.g., "day 0", "day 0")Rows correspond to sgRNAs.Values represent normalized percent abundance (%).
