# Supplementary material for: CDK12/CDK13 inhibition disrupts transcriptional elongation and replication fork progression in glioblastoma
Source: EMBO Mol Med. 2026 Mar 25;18(5):1592–624. doi: 10.1038/s44321-026-00393-w (PMC13179391; doi:10.1038/s44321-026-00393-w)
Supplement: Supplementary file 8 — Source data Fig. 1 [file 44321_2026_393_MOESM8_ESM.zip › Figure 1/1B/Readme.rtf]

README – Figure 1B (THZ531 IC50 Values)File: 1B_THZ531_IC50.csvThis file contains the IC50 values (nM) calculated from the THZ531 MTT dose–response assays shown in Figure 1A. These values were used to generate the bar graph in Figure 1B, comparing GSC and non-GSC cell lines.Data StructureEach row contains:Cell line nameIC50 (nM)NotesIC50 values were derived using nonlinear regression from the viability curves in Figure 1A.These values were used for the Mann–Whitney statistical comparison described in the legend.
