# Supplementary material for: CDK12/CDK13 inhibition disrupts transcriptional elongation and replication fork progression in glioblastoma
Source: EMBO Mol Med. 2026 Mar 25;18(5):1592–624. doi: 10.1038/s44321-026-00393-w (PMC13179391; doi:10.1038/s44321-026-00393-w)
Supplement: Supplementary file 8 — Source data Fig. 1 [file 44321_2026_393_MOESM8_ESM.zip › Figure 1/1E/1E_westerns_uncropped_annotated.pptx]

## Slide 1
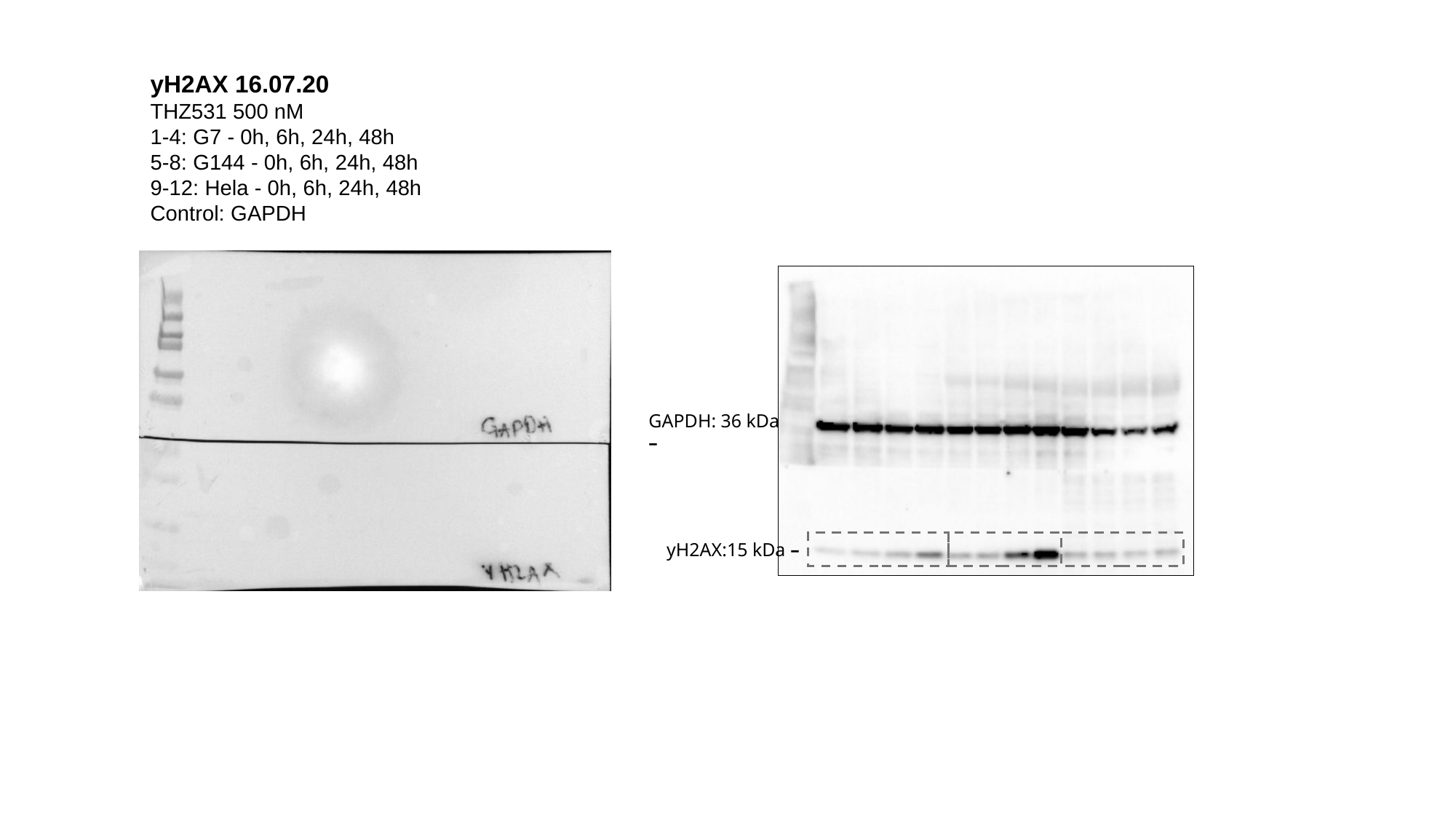

yH2AX 16.07.20
THZ531 500 nM
1-4: G7 - 0h, 6h, 24h, 48h
5-8: G144 - 0h, 6h, 24h, 48h
9-12: Hela - 0h, 6h, 24h, 48h
Control: GAPDH
GAPDH: 36 kDa –
yH2AX:15 kDa –

## Slide 2
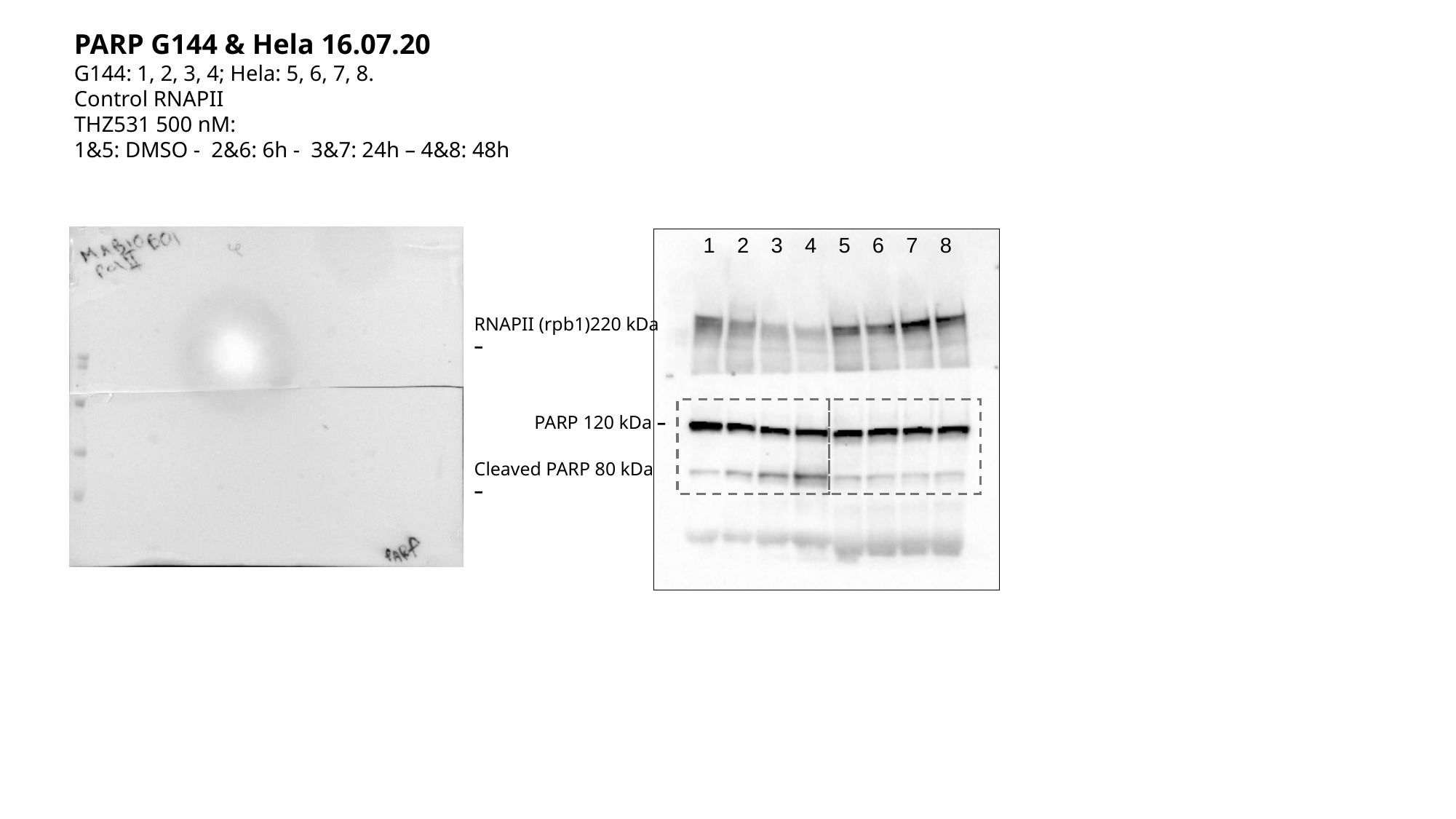

PARP G144 & Hela 16.07.20
G144: 1, 2, 3, 4; Hela: 5, 6, 7, 8.
Control RNAPII
THZ531 500 nM:
1&5: DMSO - 2&6: 6h - 3&7: 24h – 4&8: 48h
1
2
3
4
5
6
7
8
PARP 120 kDa –
Cleaved PARP 80 kDa –
RNAPII (rpb1)220 kDa –

## Slide 3
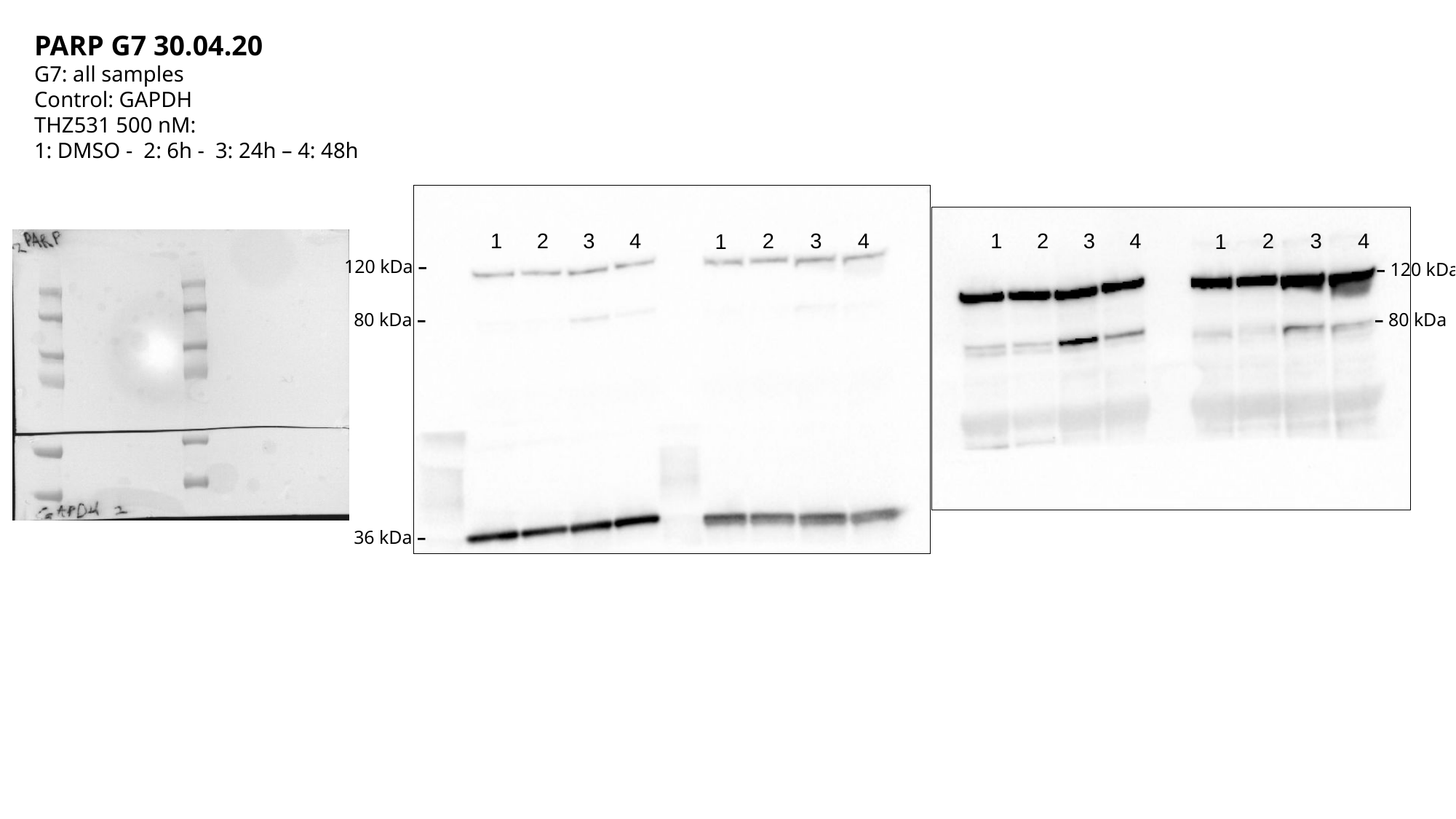

PARP G7 30.04.20
G7: all samples
Control: GAPDH
THZ531 500 nM:
1: DMSO - 2: 6h - 3: 24h – 4: 48h
4
4
1
2
3
2
3
4
1
2
3
2
3
4
1
1
120 kDa –
– 120 kDa
80 kDa –
– 80 kDa
36 kDa –
