# Supplementary material for: CDK12/CDK13 inhibition disrupts transcriptional elongation and replication fork progression in glioblastoma
Source: EMBO Mol Med. 2026 Mar 25;18(5):1592–624. doi: 10.1038/s44321-026-00393-w (PMC13179391; doi:10.1038/s44321-026-00393-w)
Supplement: Supplementary file 8 — Source data Fig. 1 [file 44321_2026_393_MOESM8_ESM.zip › Figure 1/1E/Readme.rtf]

README – Figure 1E (Immunoblot Analysis of PARP Cleavage)Current file(s): 1E_Western_blots.docxDescriptionThis folder contains the assembled western blot panels used in Figure 1E, showing levels of total PARP, cleaved PARP (cPARP), and Vinculin (loading control) in G7, G144, and HeLa cells treated with DMSO or 500 nM THZ531 for 6 h, 24 h, and 48 h.The current file shows the representative cropped blots used for figure assembly.Data included are immunoblots for:PARP and cleaved PARP (cPARP)Vinculingamma-H2AXThree cell lines: G7, G144, HeLaFour treatment conditions per cell line: DMSO, 6 h THZ531, 24 h THZ531, 48 h THZ531Antibody details are available in the reagent table.Data Pending (to be added when available)Uncropped western blot images for all probed proteins
