# Supplementary material for: CDK12/CDK13 inhibition disrupts transcriptional elongation and replication fork progression in glioblastoma
Source: EMBO Mol Med. 2026 Mar 25;18(5):1592–624. doi: 10.1038/s44321-026-00393-w (PMC13179391; doi:10.1038/s44321-026-00393-w)
Supplement: Supplementary file 8 — Source data Fig. 1 [file 44321_2026_393_MOESM8_ESM.zip › Figure 1/1D/Readme.rtf]

README – Figure 1D (Clonogenic Survival Assay)Files included: 1D_G7_CSA.JPG, 1D_G144_CSA.JPG, 1D_Hela_CSA.JPGDescriptionThese images represent the raw clonogenic survival assay plates shown in Figure 1D. The assay measures the ability of G7, G144, and HeLa cells to form colonies after treatment with THZ531 at specified concentrations.Each plate displays:Two replicate wells for DMSO controlsTwo replicate wells for 100 nM THZ531Two replicate wells for 500 nM THZ531(Corresponding positioning is documented in the laboratory notebook.)Image DetailsStain: Crystal violetPlate format: 6-well platesImages captured using standard lab imaging setup (white background, overhead illumination).No processing beyond cropping/rotation has been applied.
