# Supplementary material for: CDK12/CDK13 inhibition disrupts transcriptional elongation and replication fork progression in glioblastoma
Source: EMBO Mol Med. 2026 Mar 25;18(5):1592–624. doi: 10.1038/s44321-026-00393-w (PMC13179391; doi:10.1038/s44321-026-00393-w)
Supplement: Supplementary file 8 — Source data Fig. 1 [file 44321_2026_393_MOESM8_ESM.zip › Figure 1/1C/Readme.rtf]

README – Figure 1C (Proliferation Assays for G7, G144, and HeLa Cells)Files: 1C_G7_proliferation_assay.csv, 1C_G144_proliferation_assay.csv, 1C_Hela_proliferation_assay.csvDescriptionThese files contain the raw proliferation assay data used to generate the line plots shown in Figure 1C, comparing the growth of G7, G144, and HeLa cells under:DMSO control (two replicates),100 nM THZ531 (two replicates), 500 nM THZ531 (two replicates)Cell numbers were measured across multiple timepoints (e.g., day 0, day 4, day 8, etc.).Data StructureEach CSV includes:Row: Time point (e.g., day 0, day 4, day 8 …)Columns: Two replicates for DMSO, Two replicates for 100 nM THZ531, Two replicates for 500 nM THZ531Values represent absolute cell counts.Notes:The CSVs use semicolon separators and include spacing artifacts from spreadsheet export.Some values may appear in exponential notation depending on assay growth dynamics.UsageThese data were used to:Plot proliferation curves (mean ± SD).Perform two-way ANOVA with Tukey’s multiple comparisons test, as described in the legend.
