# Supplementary figures and images for: CDK12/CDK13 inhibition disrupts transcriptional elongation and replication fork progression in glioblastoma (part 1 of 2)
Source: EMBO Mol Med. 2026 Mar 25;18(5):1592–624. doi: 10.1038/s44321-026-00393-w (PMC13179391; doi:10.1038/s44321-026-00393-w)

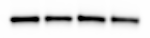

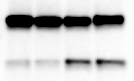

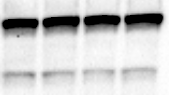

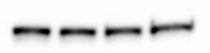

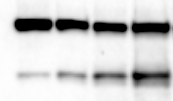

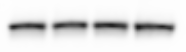

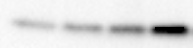

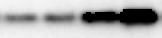

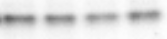


Vinculin

PARP

cPARP

G7

DMSO 6h 24h 48h

G144

Hela

p-H2AX

DMSO 6h 24h 48h

DMSO 6h 24h 48h

Supplement: Supplementary file 8 — Source data Fig. 1 [file 44321_2026_393_MOESM8_ESM.zip › Figure 1/1E/1E_Western_blots.docx]

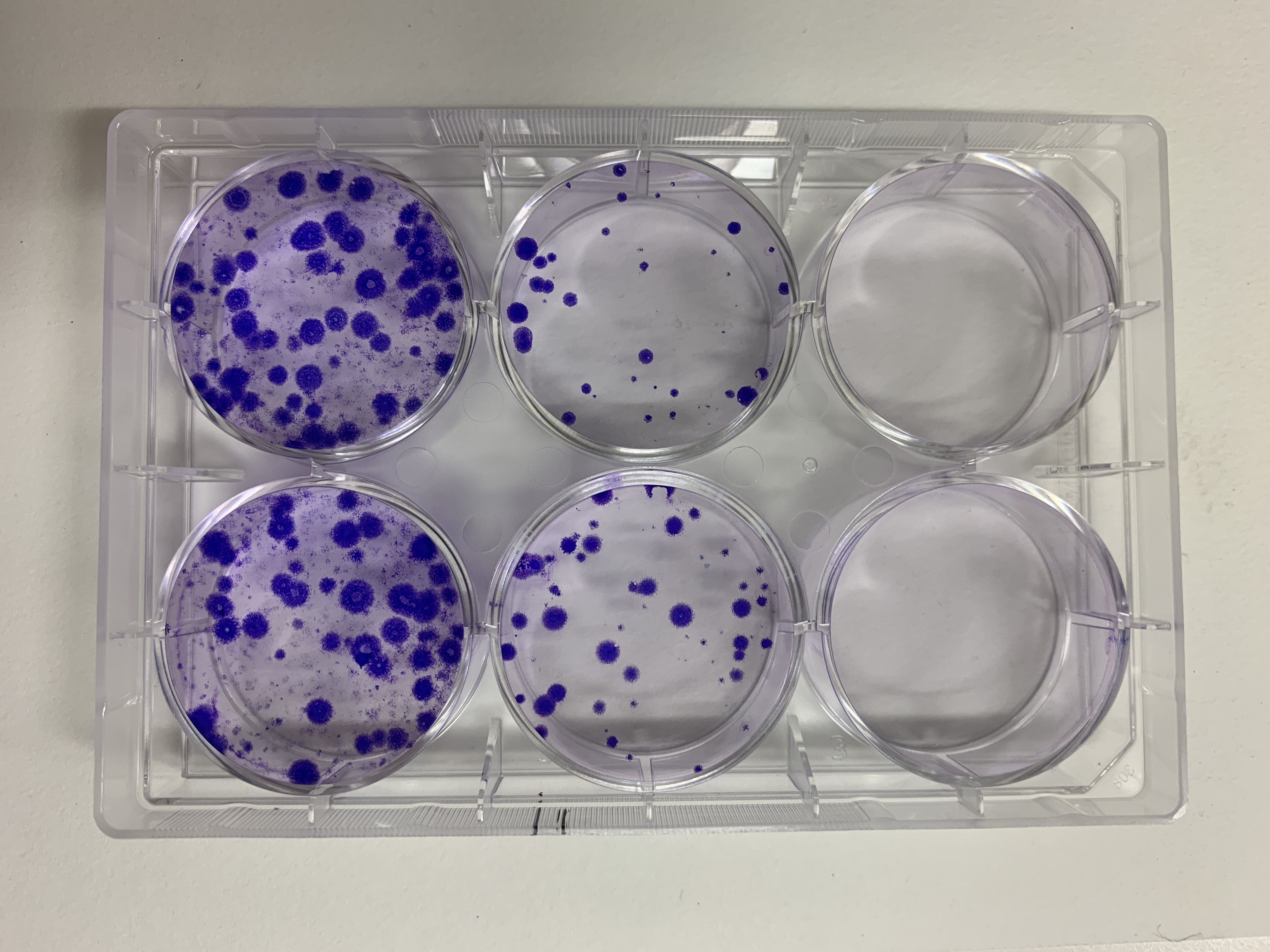

Supplement: Supplementary file 8 — Source data Fig. 1 [file 44321_2026_393_MOESM8_ESM.zip › Figure 1/1D/1D_G7_CSA.JPG]

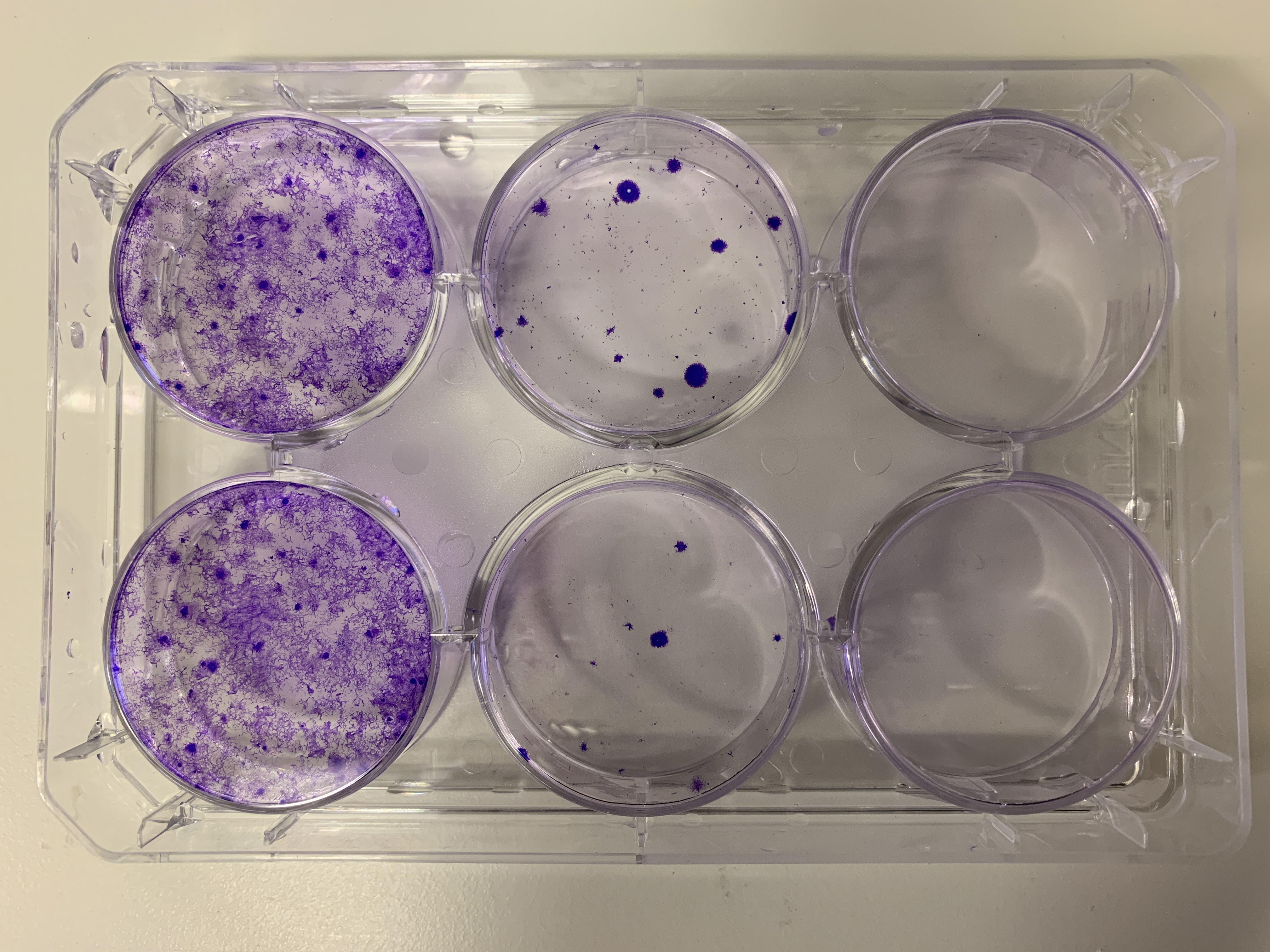

Supplement: Supplementary file 8 — Source data Fig. 1 [file 44321_2026_393_MOESM8_ESM.zip › Figure 1/1D/1D_G144_CSA.JPG]

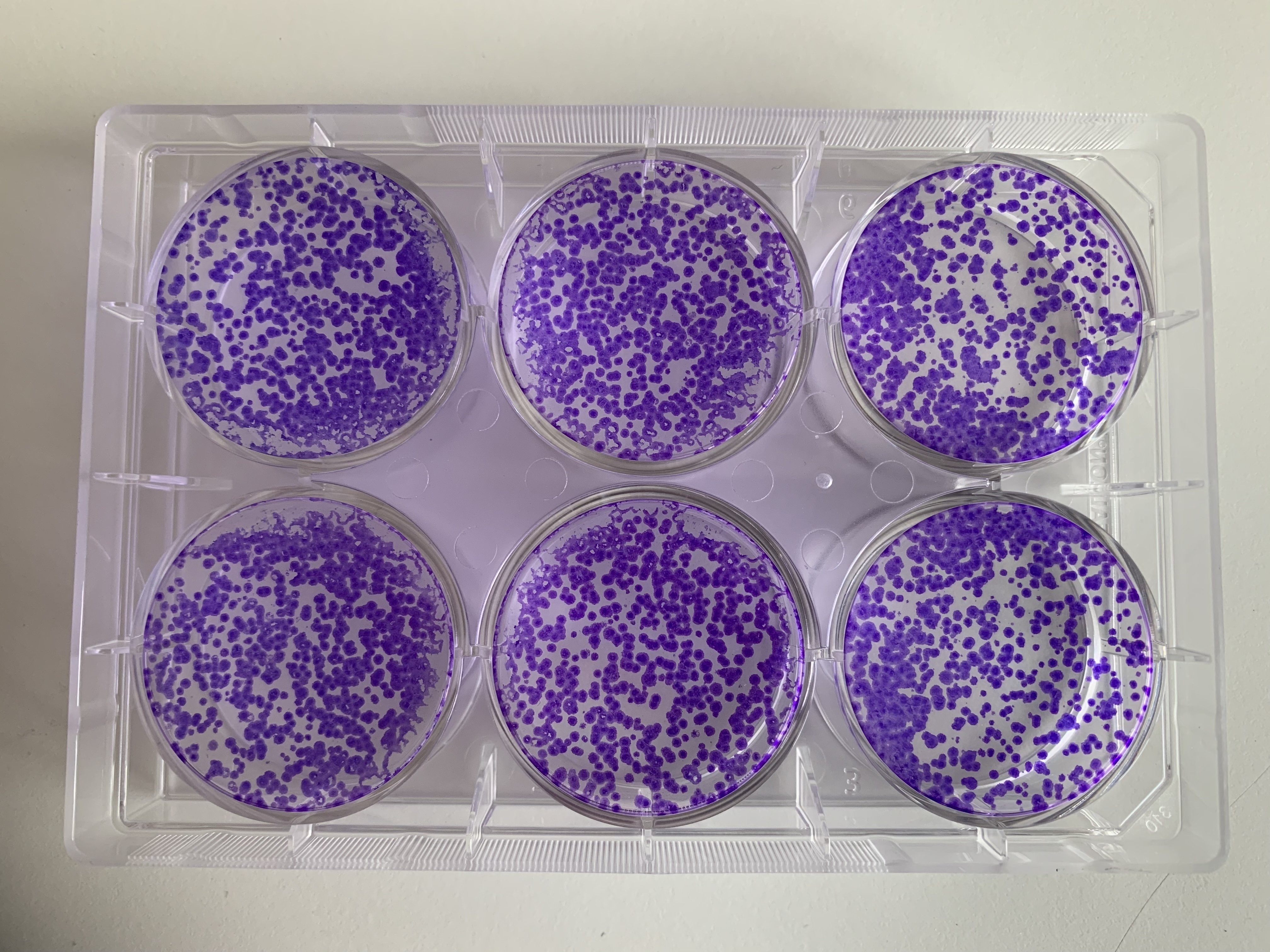

Supplement: Supplementary file 8 — Source data Fig. 1 [file 44321_2026_393_MOESM8_ESM.zip › Figure 1/1D/1D_Hela_CSA.JPG]

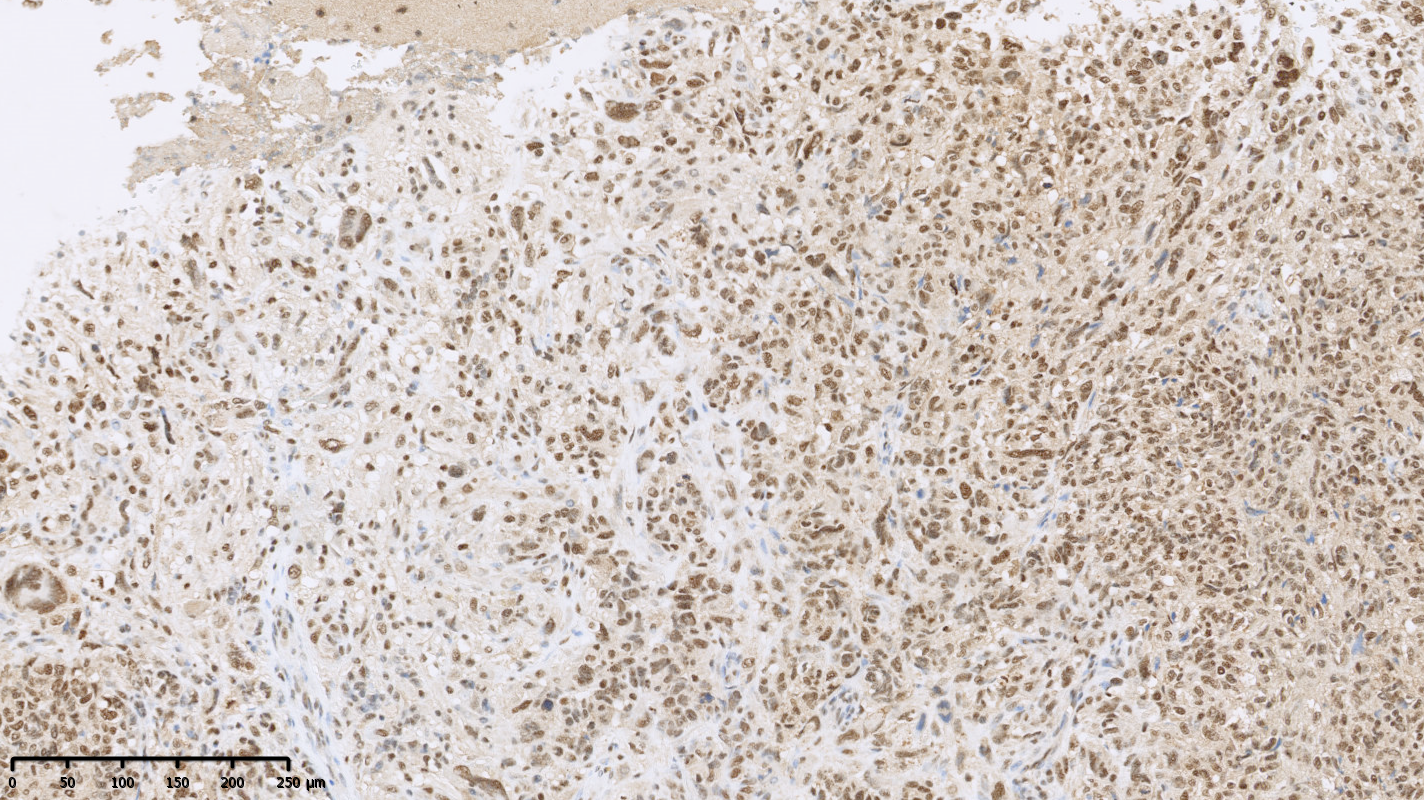

Supplement: Supplementary file 9 — Source data Fig. 2 [file 44321_2026_393_MOESM9_ESM.zip › Figure 2/2A/2A Glioblastoma C17_CDK12.tif]

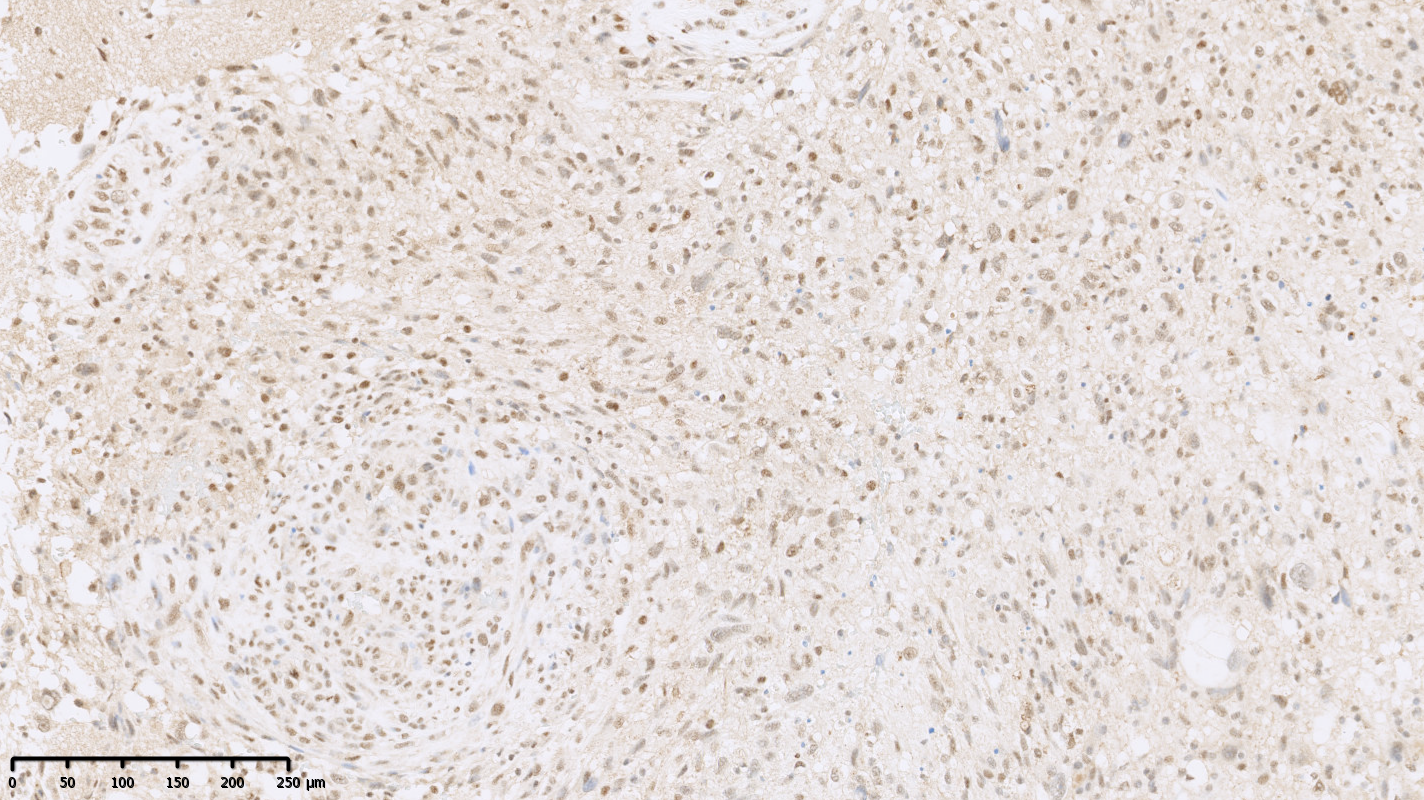

Supplement: Supplementary file 9 — Source data Fig. 2 [file 44321_2026_393_MOESM9_ESM.zip › Figure 2/2A/2A Glioblastoma C21_CDK12.tif]

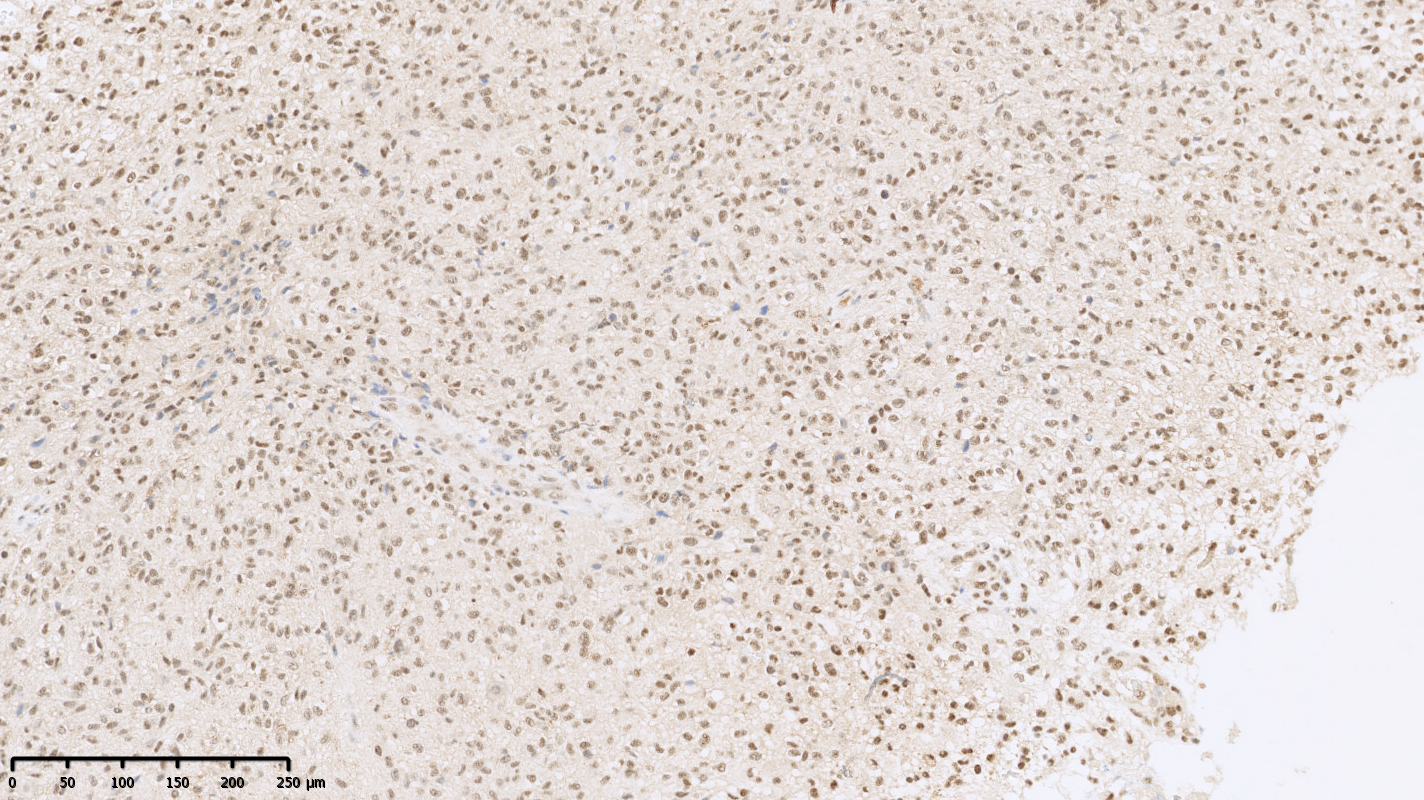

Supplement: Supplementary file 9 — Source data Fig. 2 [file 44321_2026_393_MOESM9_ESM.zip › Figure 2/2A/2A Glioblastoma C19_CDK12.tif]

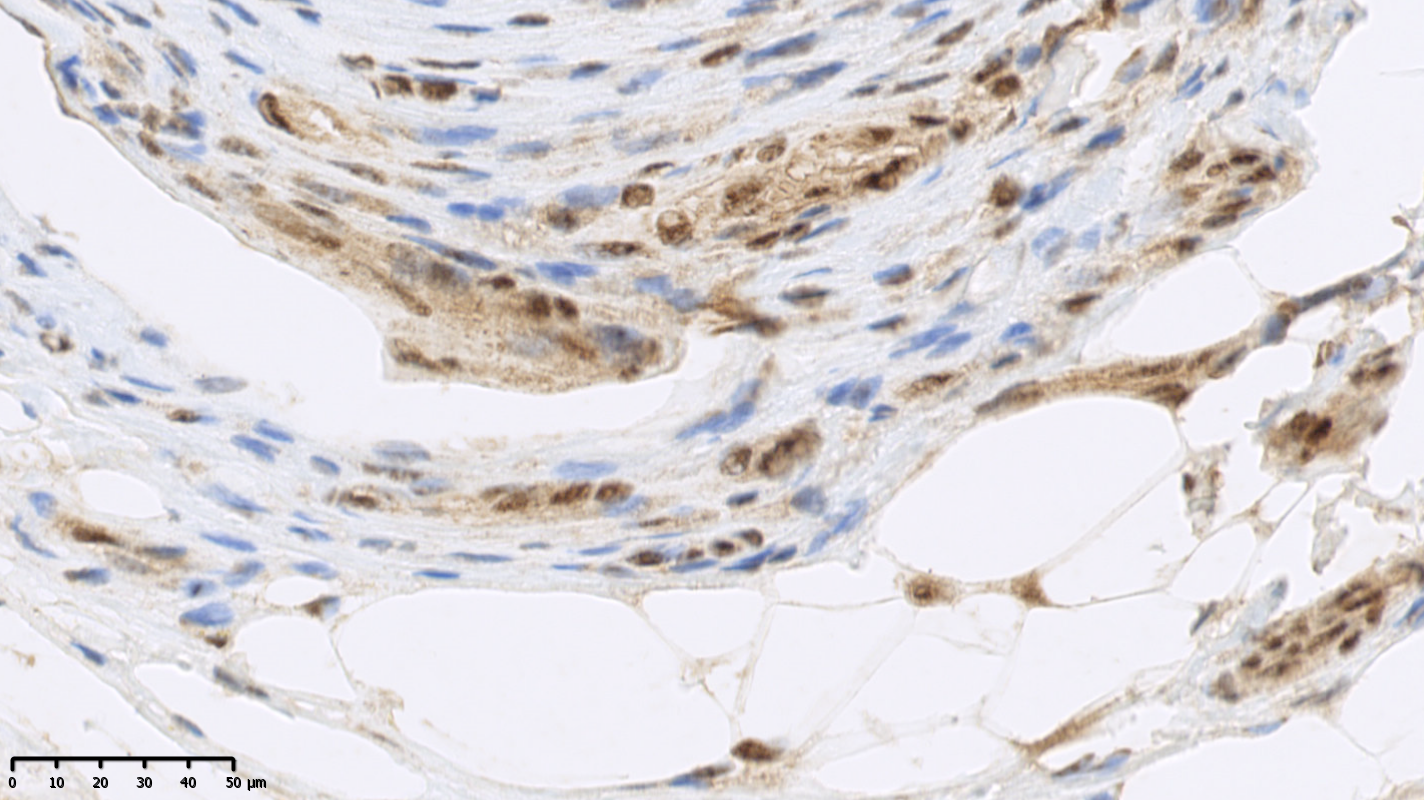

Supplement: Supplementary file 9 — Source data Fig. 2 [file 44321_2026_393_MOESM9_ESM.zip › Figure 2/2A/2A Control tissue C12_CDK12 close-up_40x.tif]

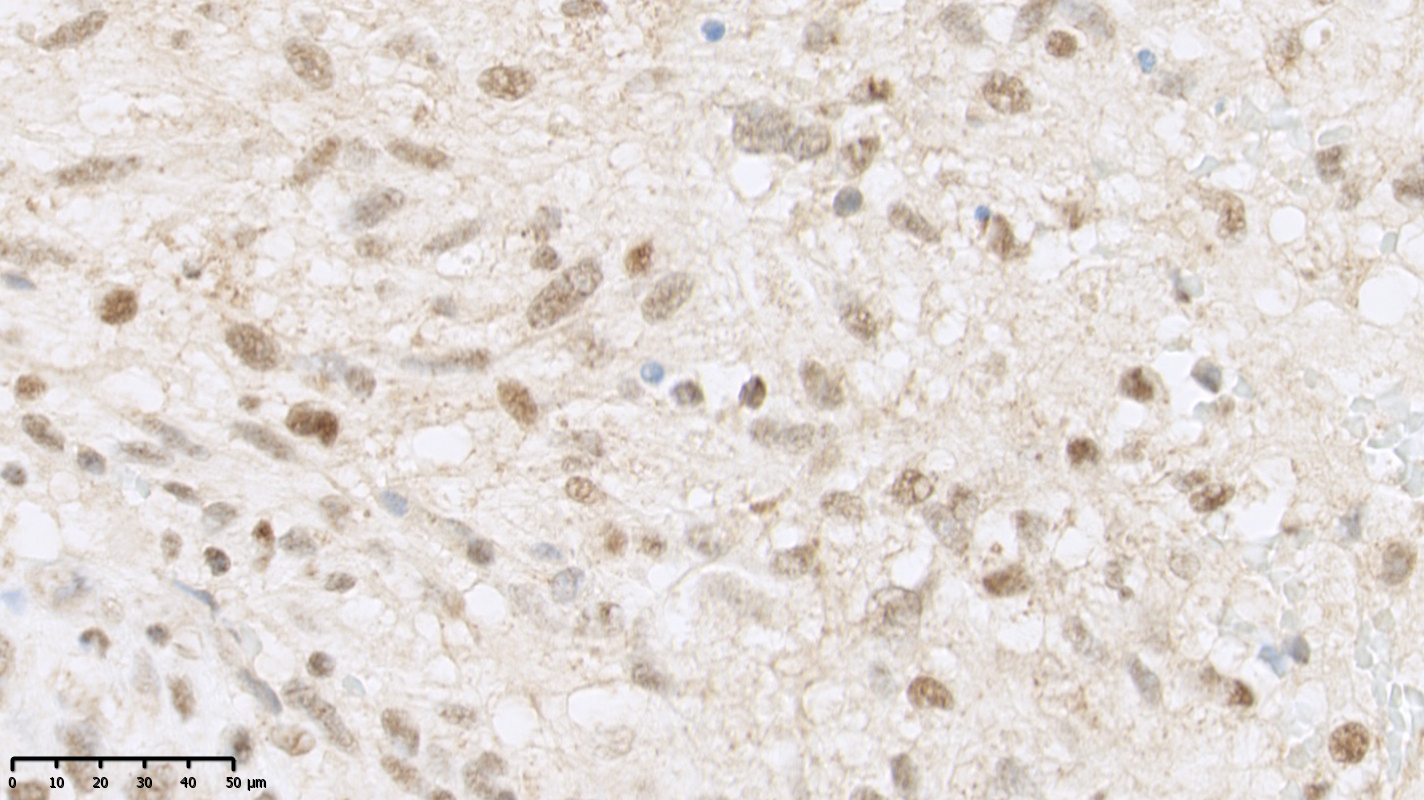

Supplement: Supplementary file 9 — Source data Fig. 2 [file 44321_2026_393_MOESM9_ESM.zip › Figure 2/2A/2A Glioblastoma C21_CDK12 close-up_40x.tif]

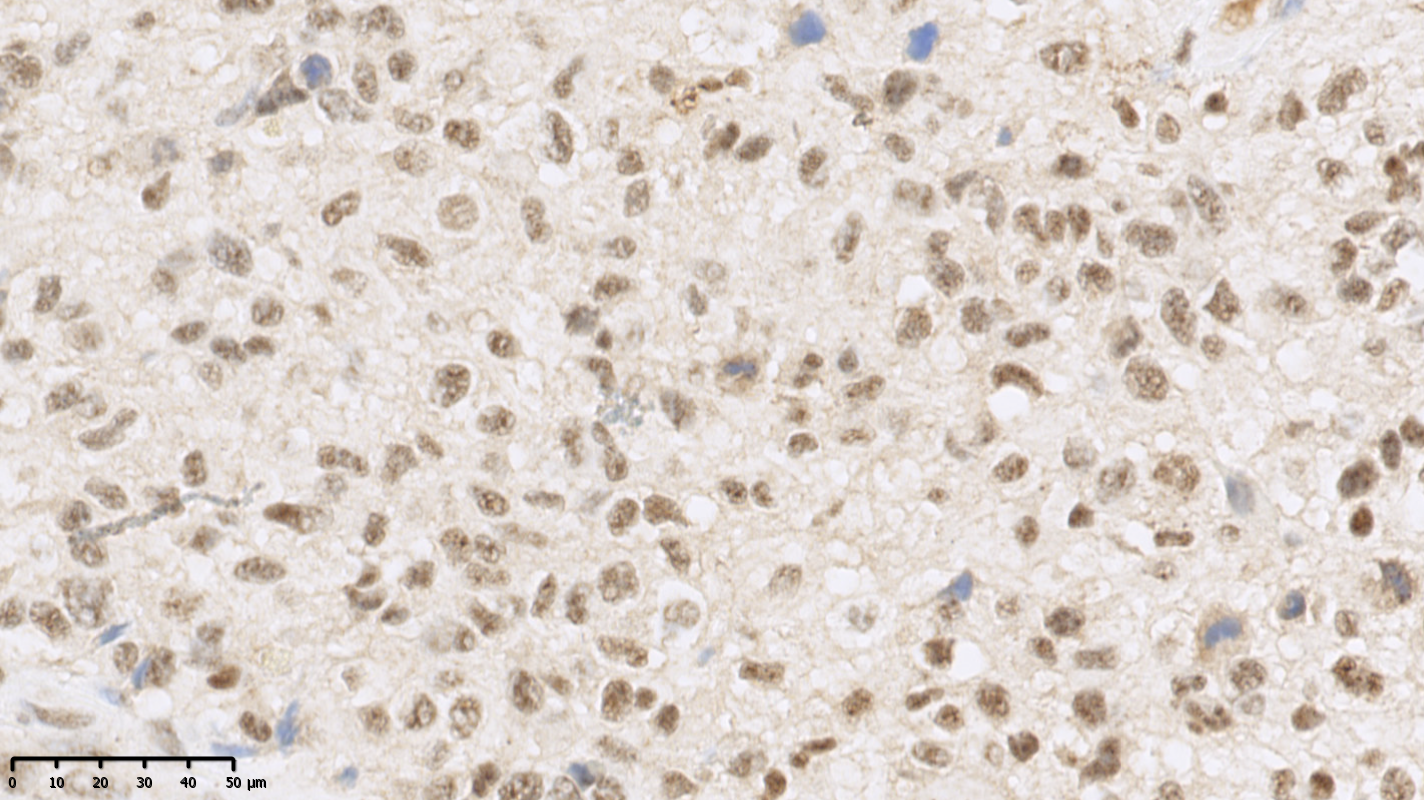

Supplement: Supplementary file 9 — Source data Fig. 2 [file 44321_2026_393_MOESM9_ESM.zip › Figure 2/2A/2A Glioblastoma C19 CDK12 close-up_40x.tif]

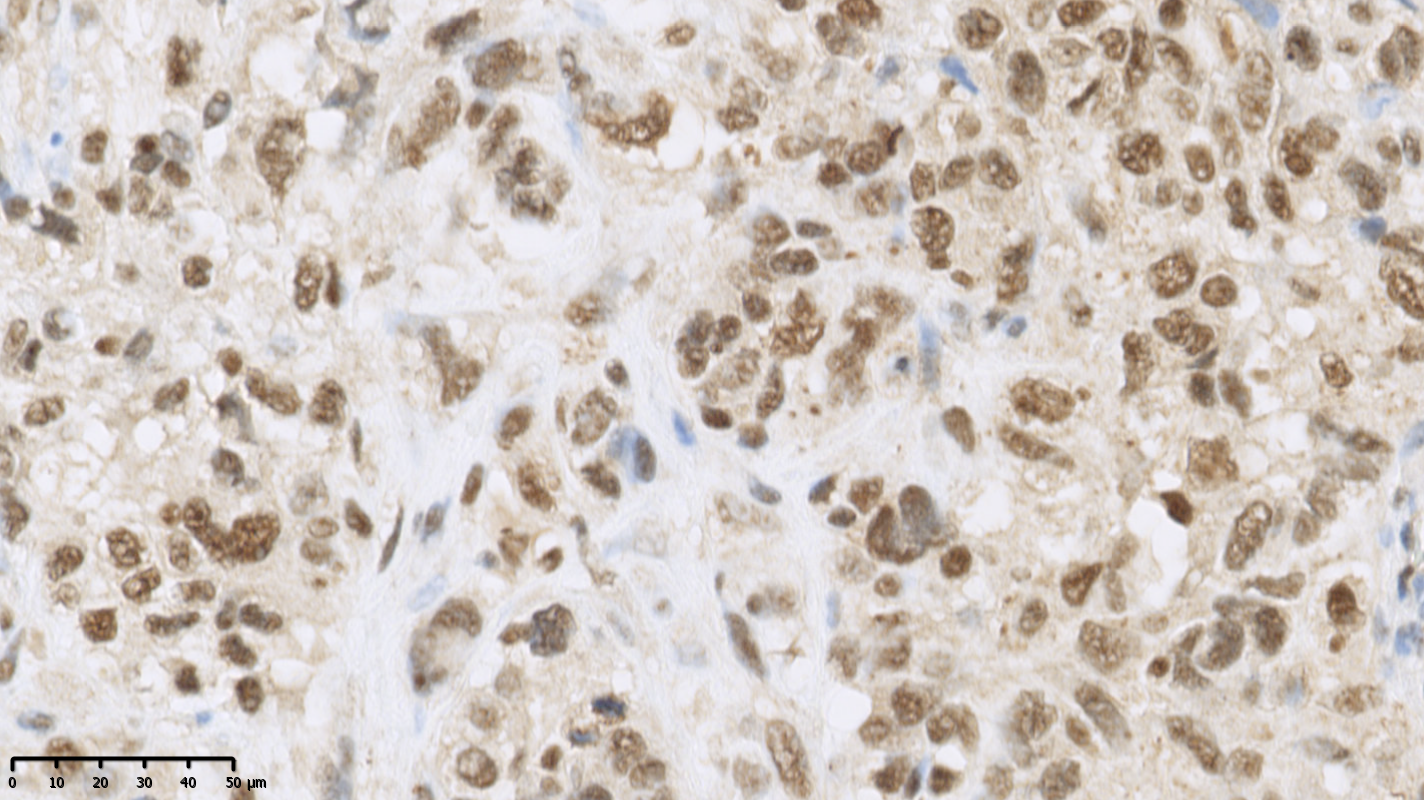

Supplement: Supplementary file 9 — Source data Fig. 2 [file 44321_2026_393_MOESM9_ESM.zip › Figure 2/2A/2A Glioblastoma C17_CDK12 close-up_40x.tif]

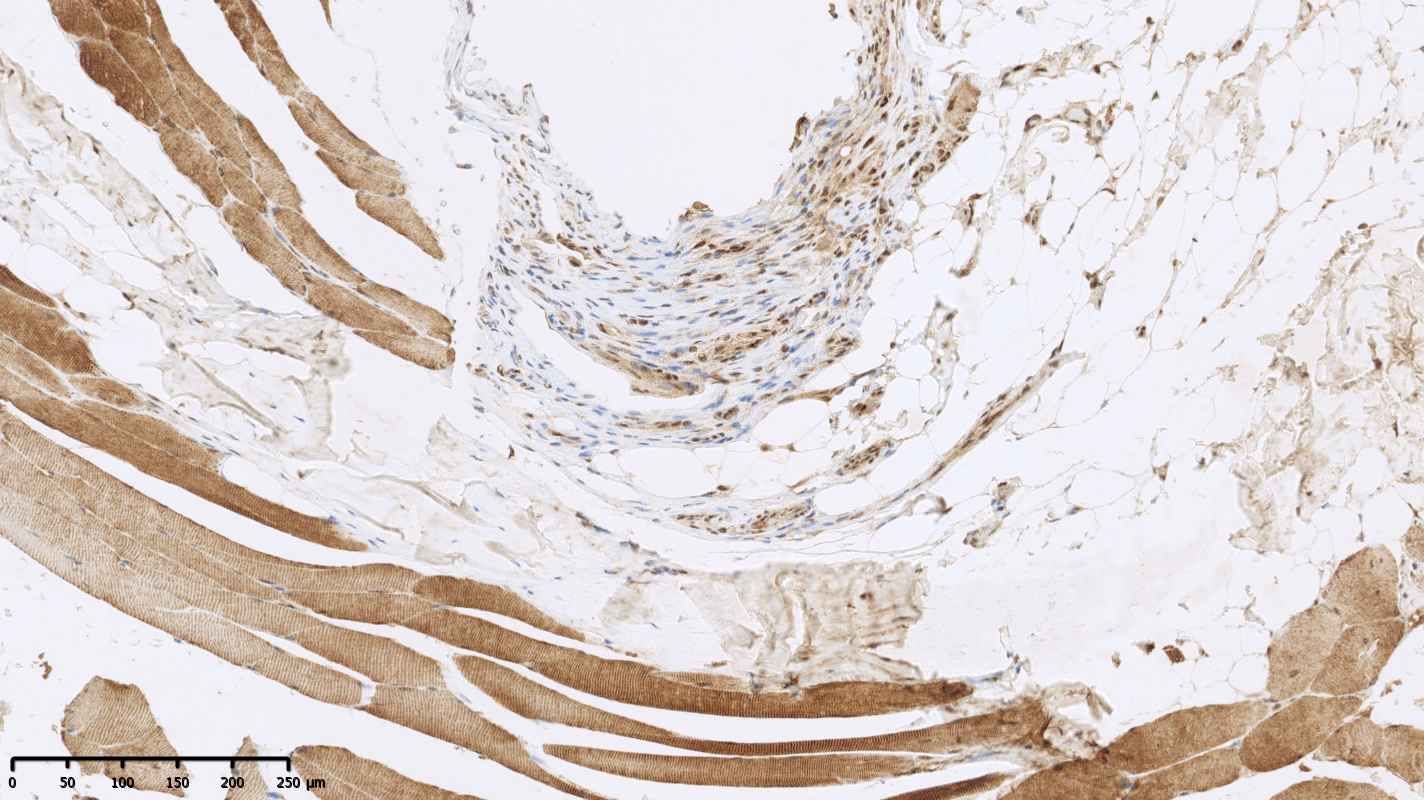

Supplement: Supplementary file 9 — Source data Fig. 2 [file 44321_2026_393_MOESM9_ESM.zip › Figure 2/2A/2A Control tissue C12_CDK12.tif]

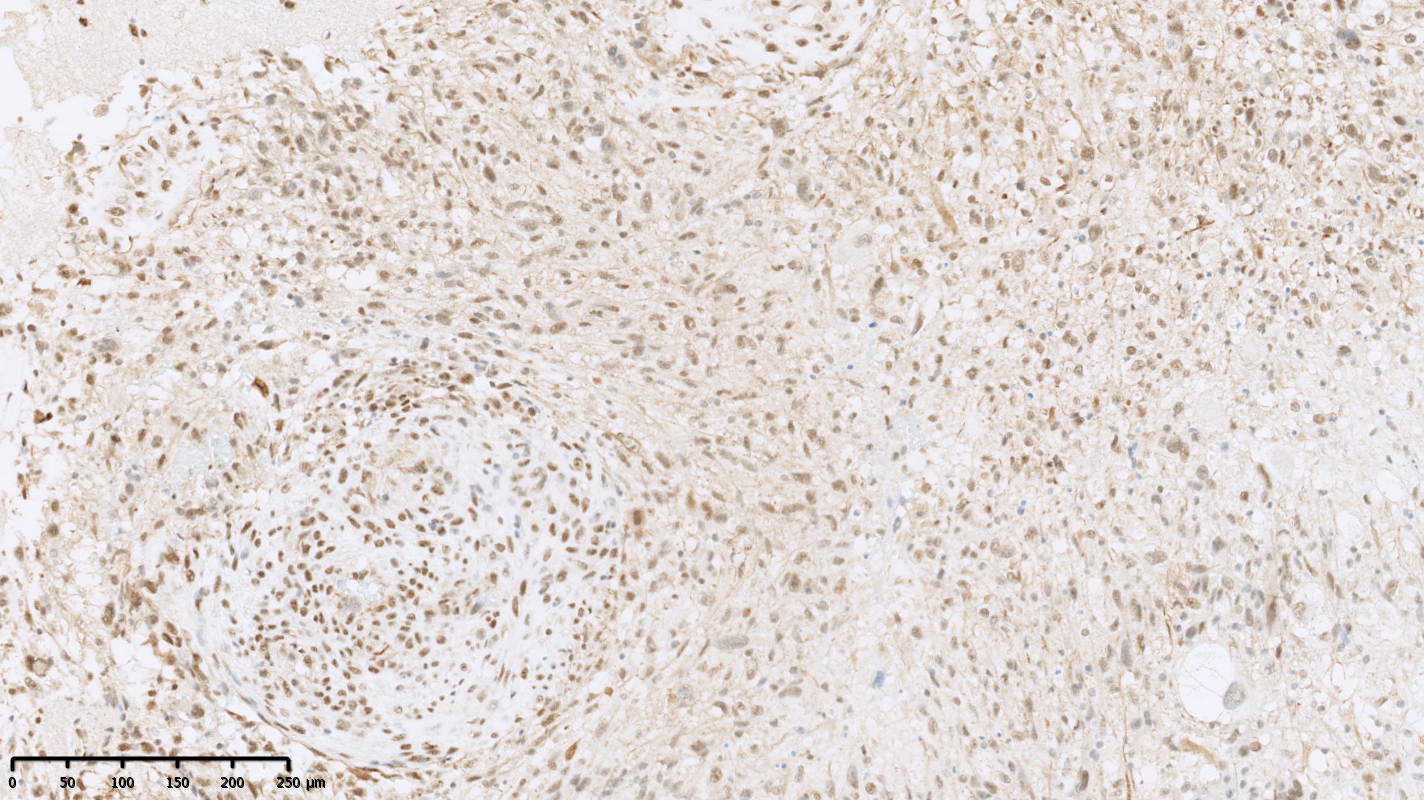

Supplement: Supplementary file 9 — Source data Fig. 2 [file 44321_2026_393_MOESM9_ESM.zip › Figure 2/2B/2B Glioblastoma C21_CDK13.tif]

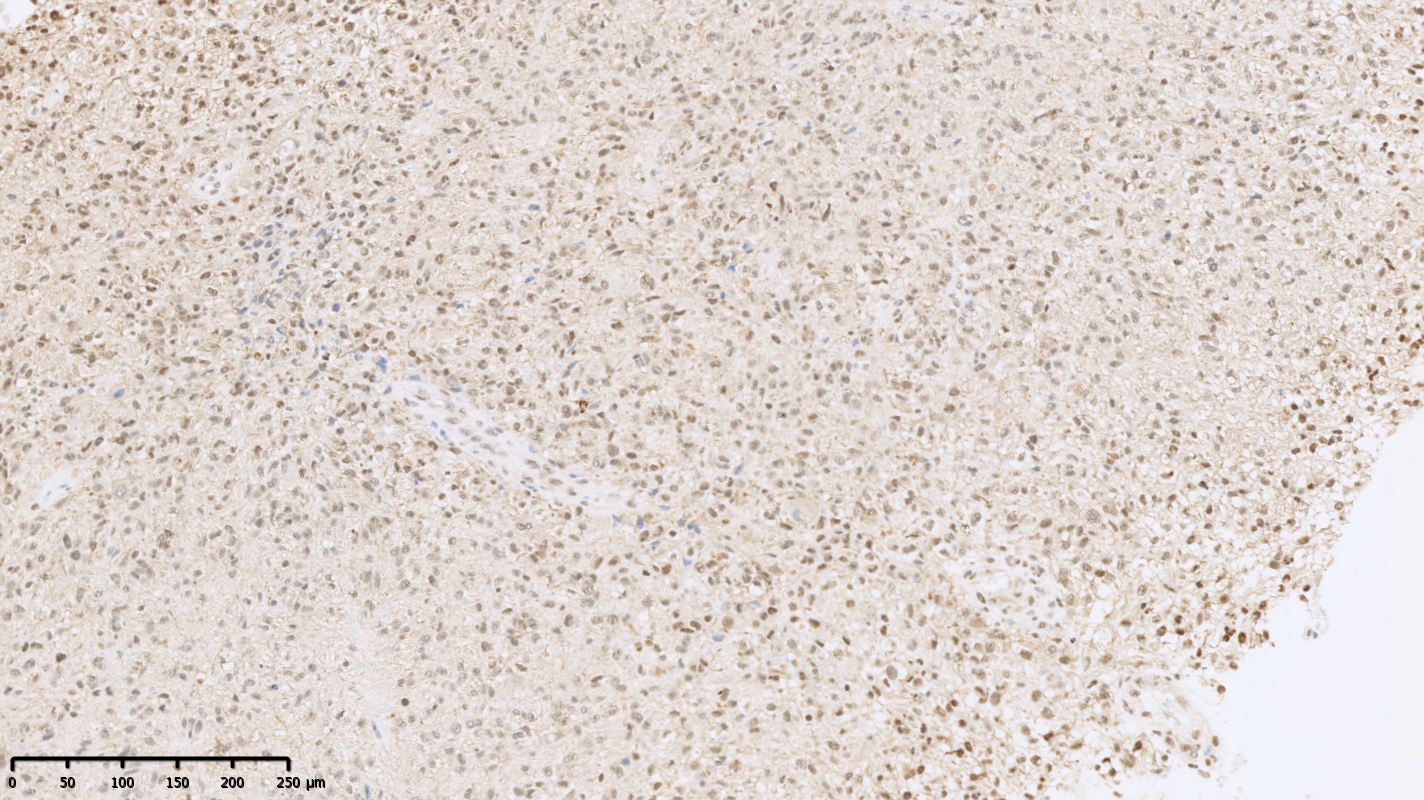

Supplement: Supplementary file 9 — Source data Fig. 2 [file 44321_2026_393_MOESM9_ESM.zip › Figure 2/2B/2B Glioblastoma C19_CDK13.tif]

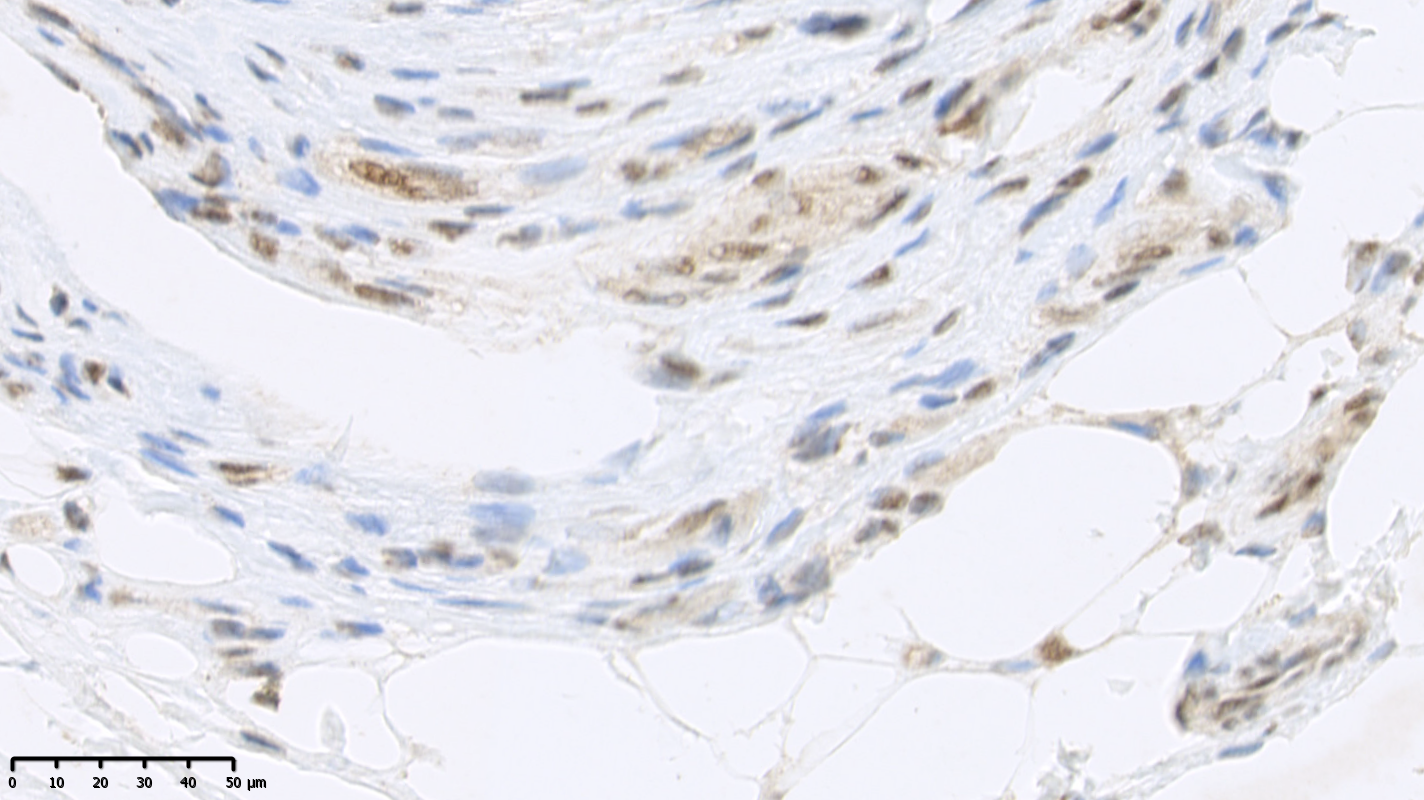

Supplement: Supplementary file 9 — Source data Fig. 2 [file 44321_2026_393_MOESM9_ESM.zip › Figure 2/2B/2B Control tissue C12_CDK13 close-up_40x.tif]

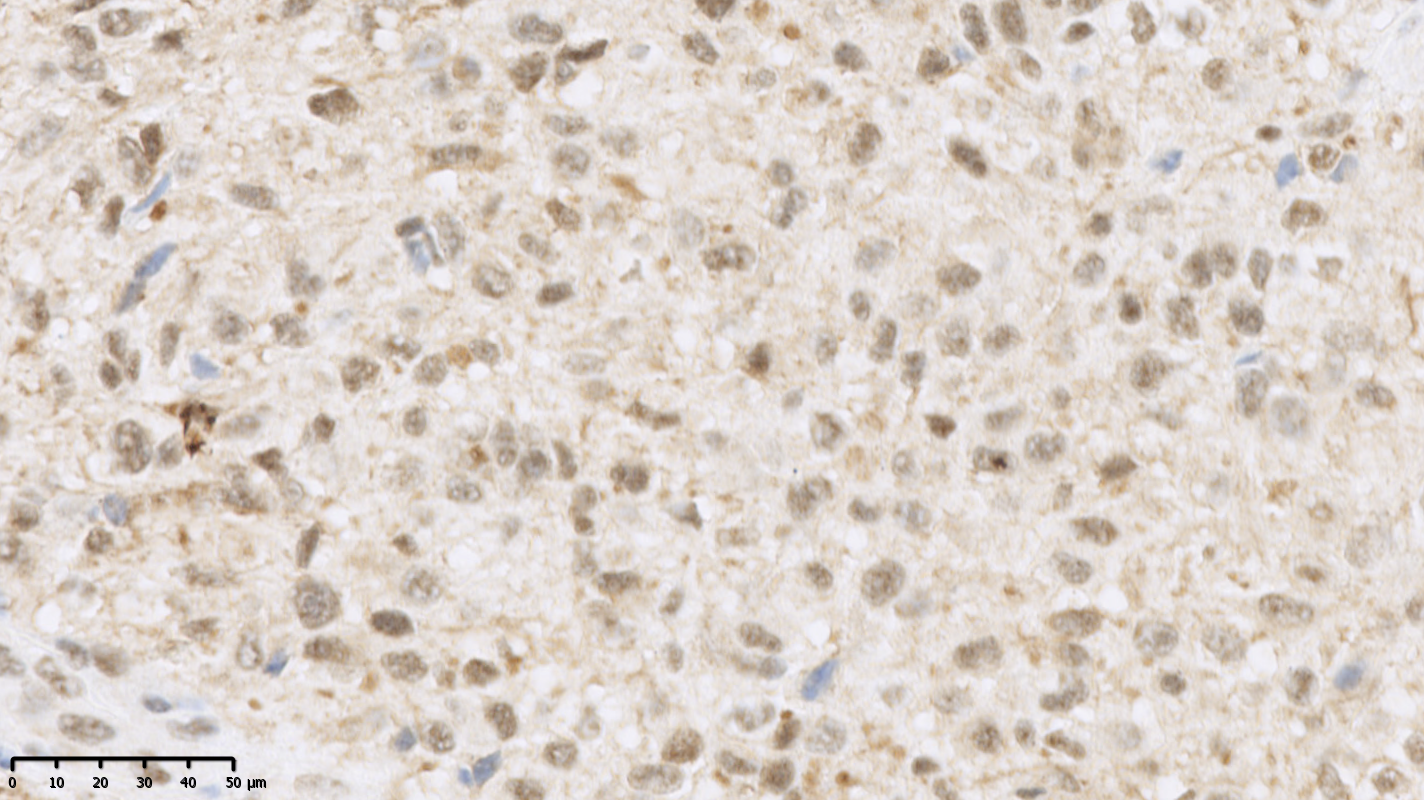

Supplement: Supplementary file 9 — Source data Fig. 2 [file 44321_2026_393_MOESM9_ESM.zip › Figure 2/2B/2B Glioblastoma C19_CDK13 close-up_40x.tif]

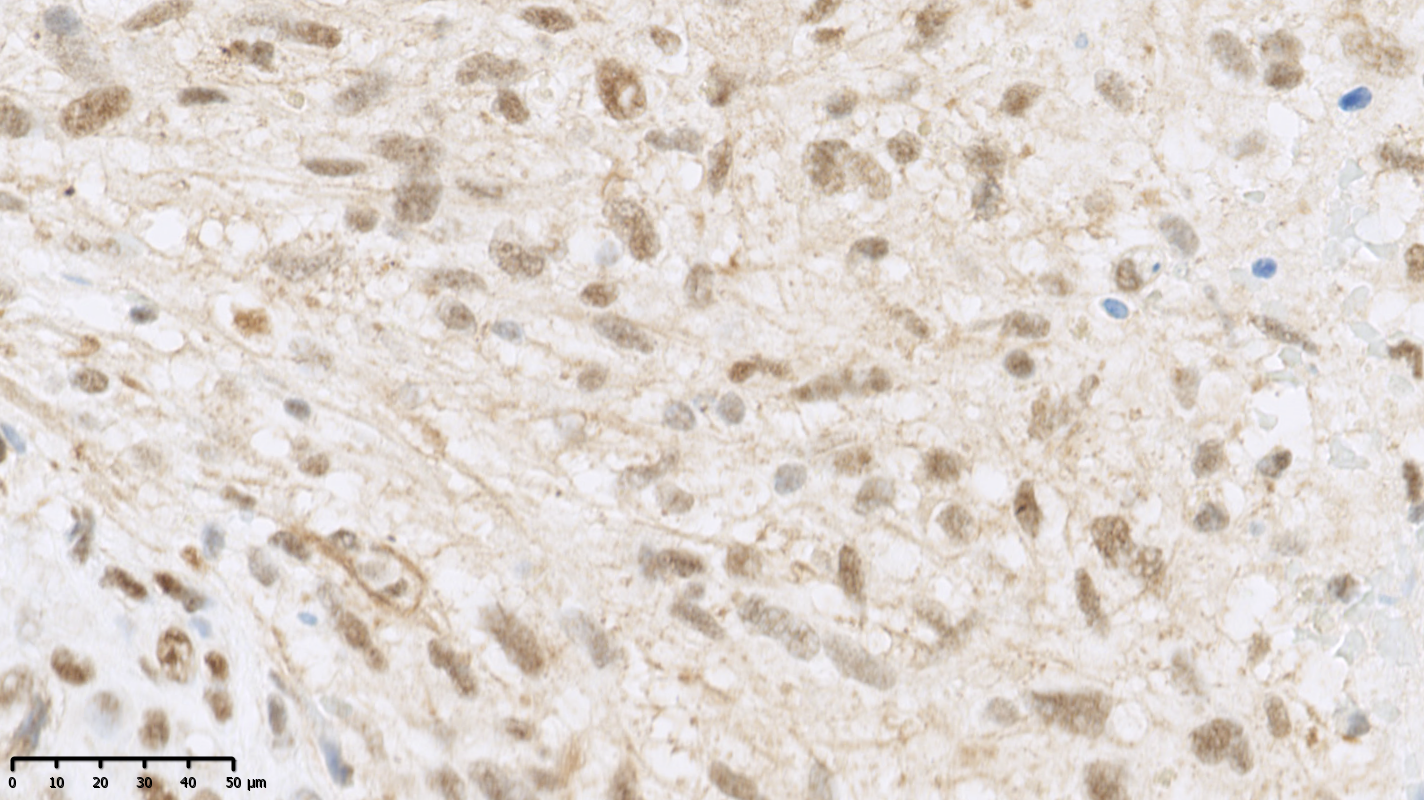

Supplement: Supplementary file 9 — Source data Fig. 2 [file 44321_2026_393_MOESM9_ESM.zip › Figure 2/2B/2B Glioblastoma C21_CDK13 close-up_40x.tif]

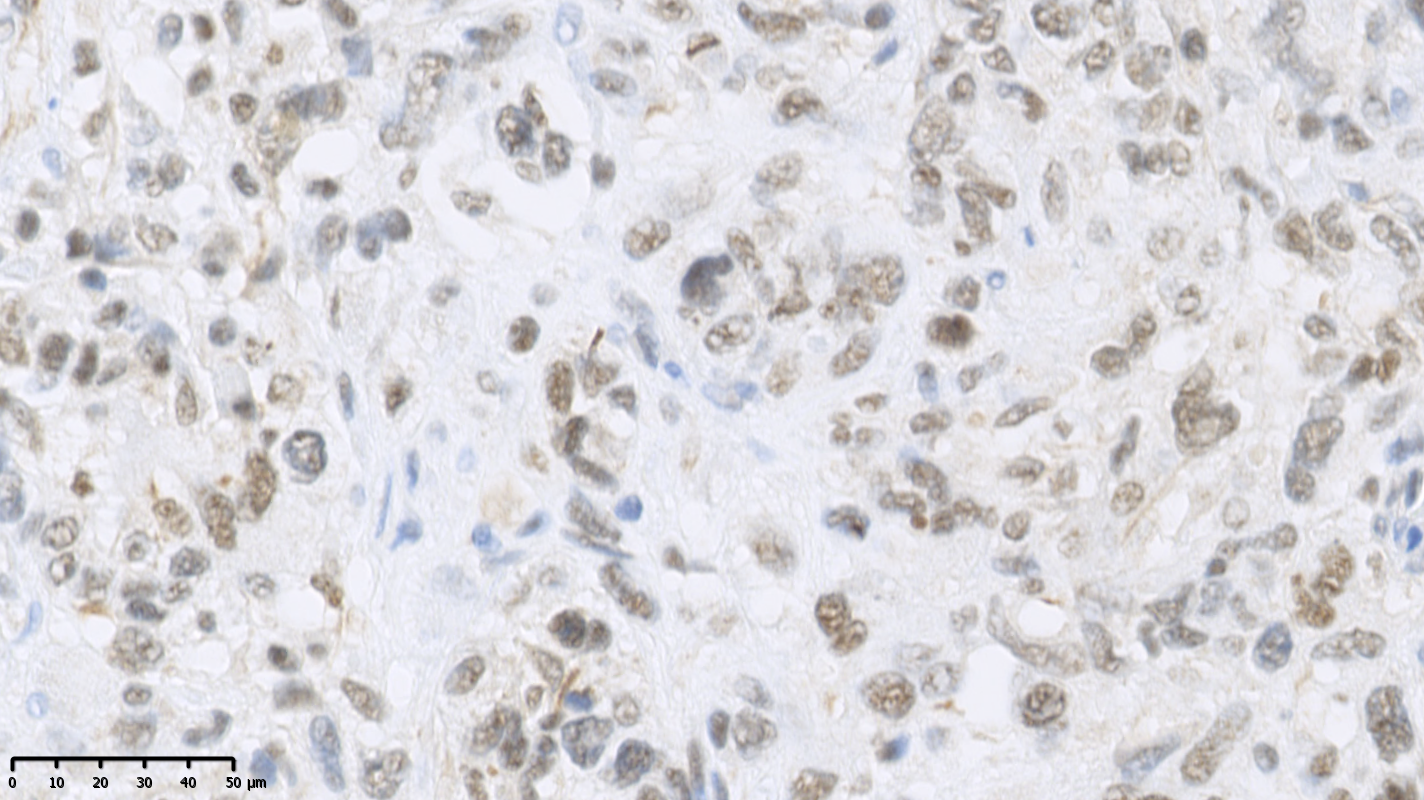

Supplement: Supplementary file 9 — Source data Fig. 2 [file 44321_2026_393_MOESM9_ESM.zip › Figure 2/2B/2B Glioblastoma C17_CDK13 close-up_40x.tif]

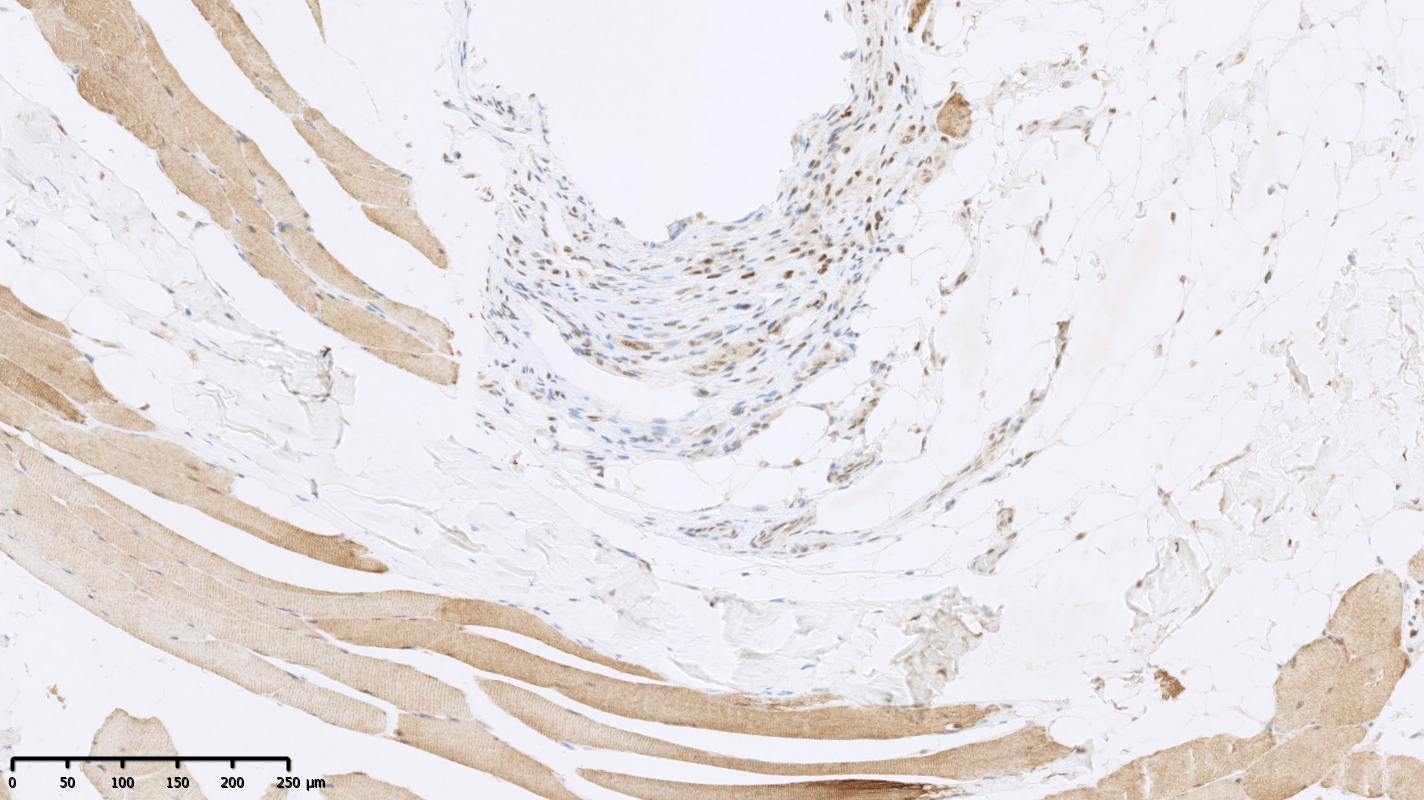

Supplement: Supplementary file 9 — Source data Fig. 2 [file 44321_2026_393_MOESM9_ESM.zip › Figure 2/2B/2B Control tissue C12_CDK13.tif]

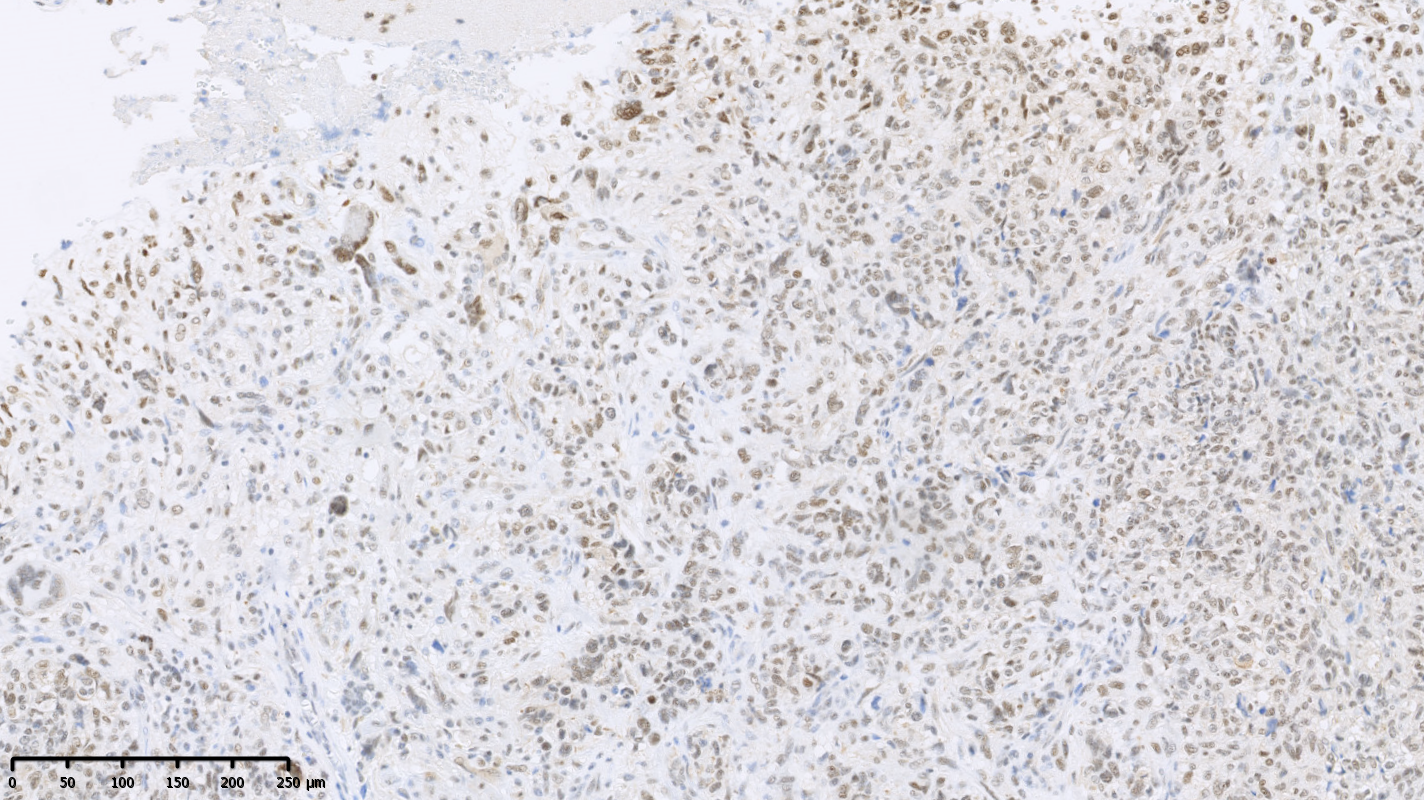

Supplement: Supplementary file 9 — Source data Fig. 2 [file 44321_2026_393_MOESM9_ESM.zip › Figure 2/2B/2B Glioblastoma C17_CDK13.tif]

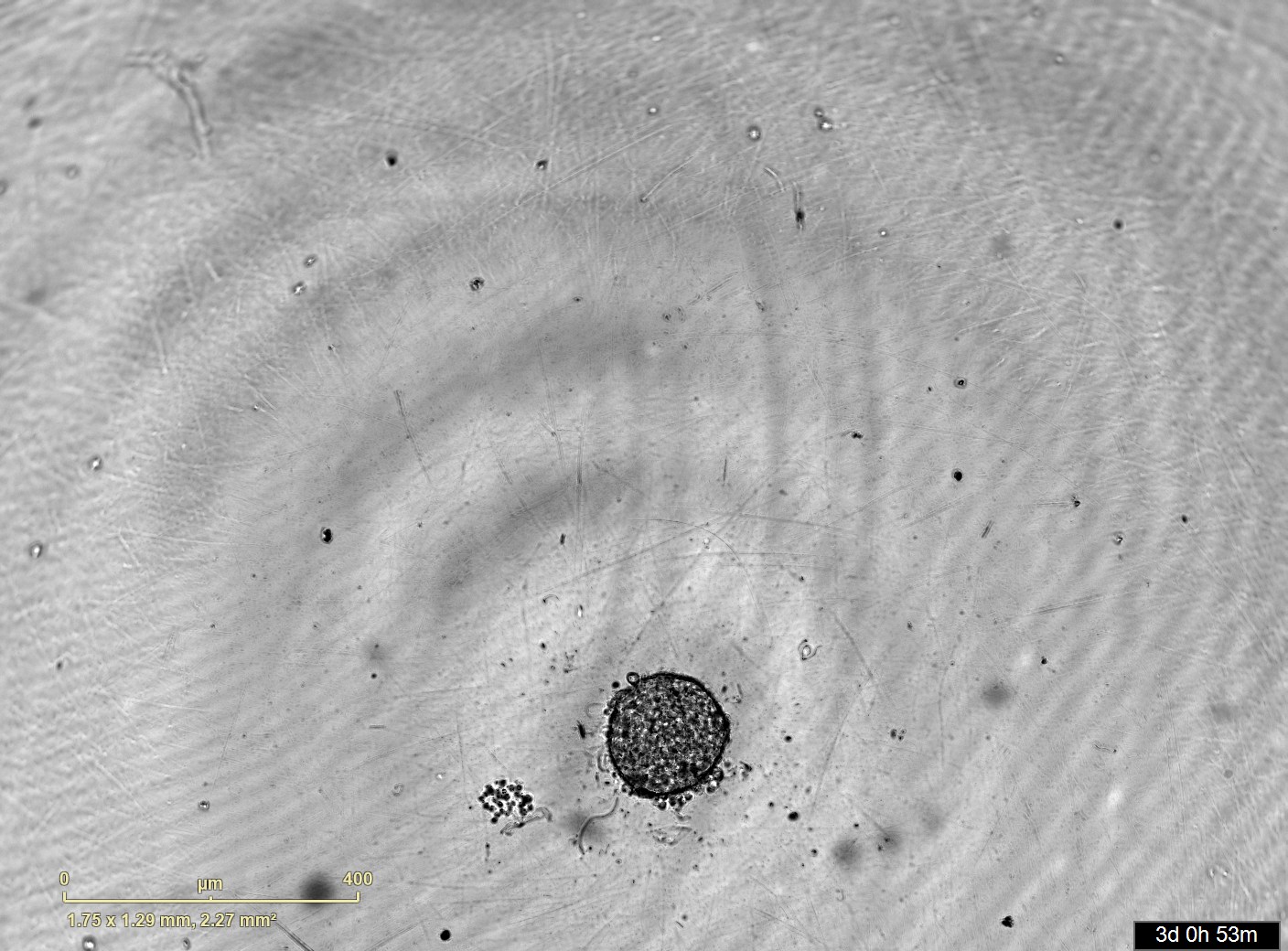

Supplement: Supplementary file 9 — Source data Fig. 2 [file 44321_2026_393_MOESM9_ESM.zip › Figure 2/2C/2C/2C_Abemaciclib_72h_control.jpg]

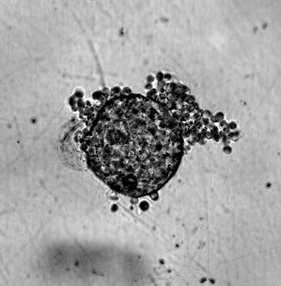

Supplement: Supplementary file 9 — Source data Fig. 2 [file 44321_2026_393_MOESM9_ESM.zip › Figure 2/2C/2C/2C_SR4835_0h_control.jpg]

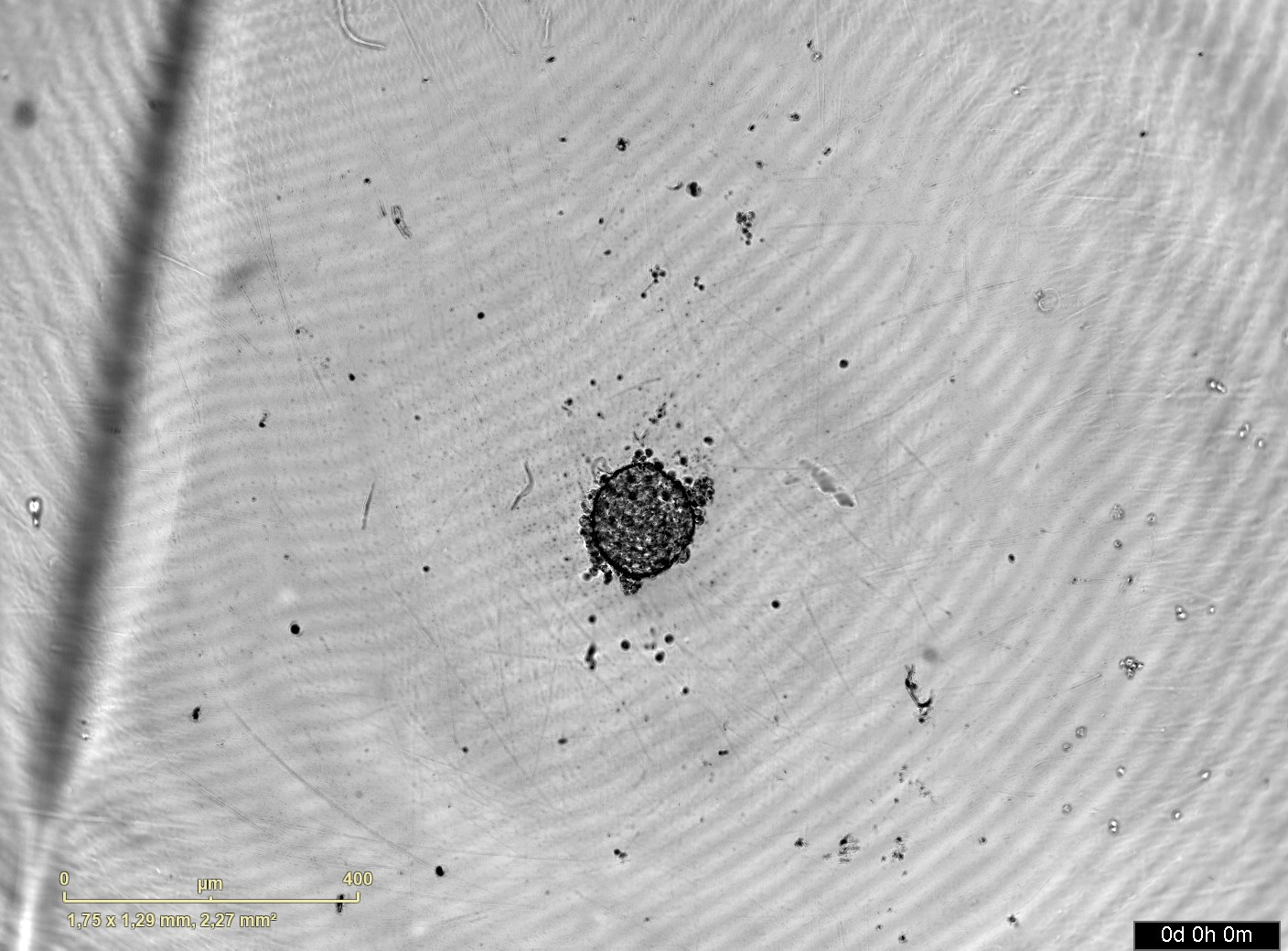

Supplement: Supplementary file 9 — Source data Fig. 2 [file 44321_2026_393_MOESM9_ESM.zip › Figure 2/2C/2C/2C_Abemaciclib_0h_1uM.tiff]

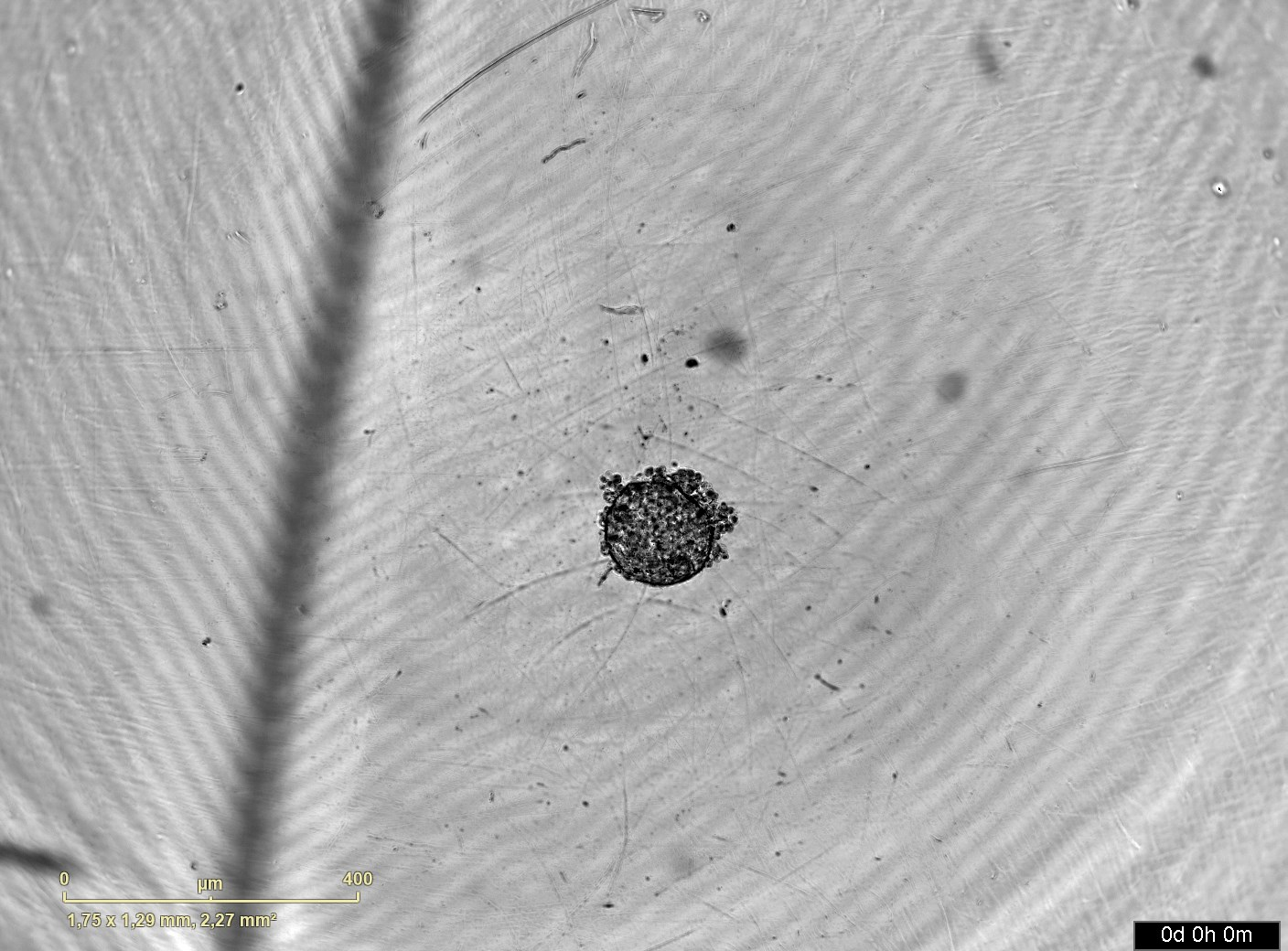

Supplement: Supplementary file 9 — Source data Fig. 2 [file 44321_2026_393_MOESM9_ESM.zip › Figure 2/2C/2C/2C_Abemaciclib_0h_0.5uM.tiff]

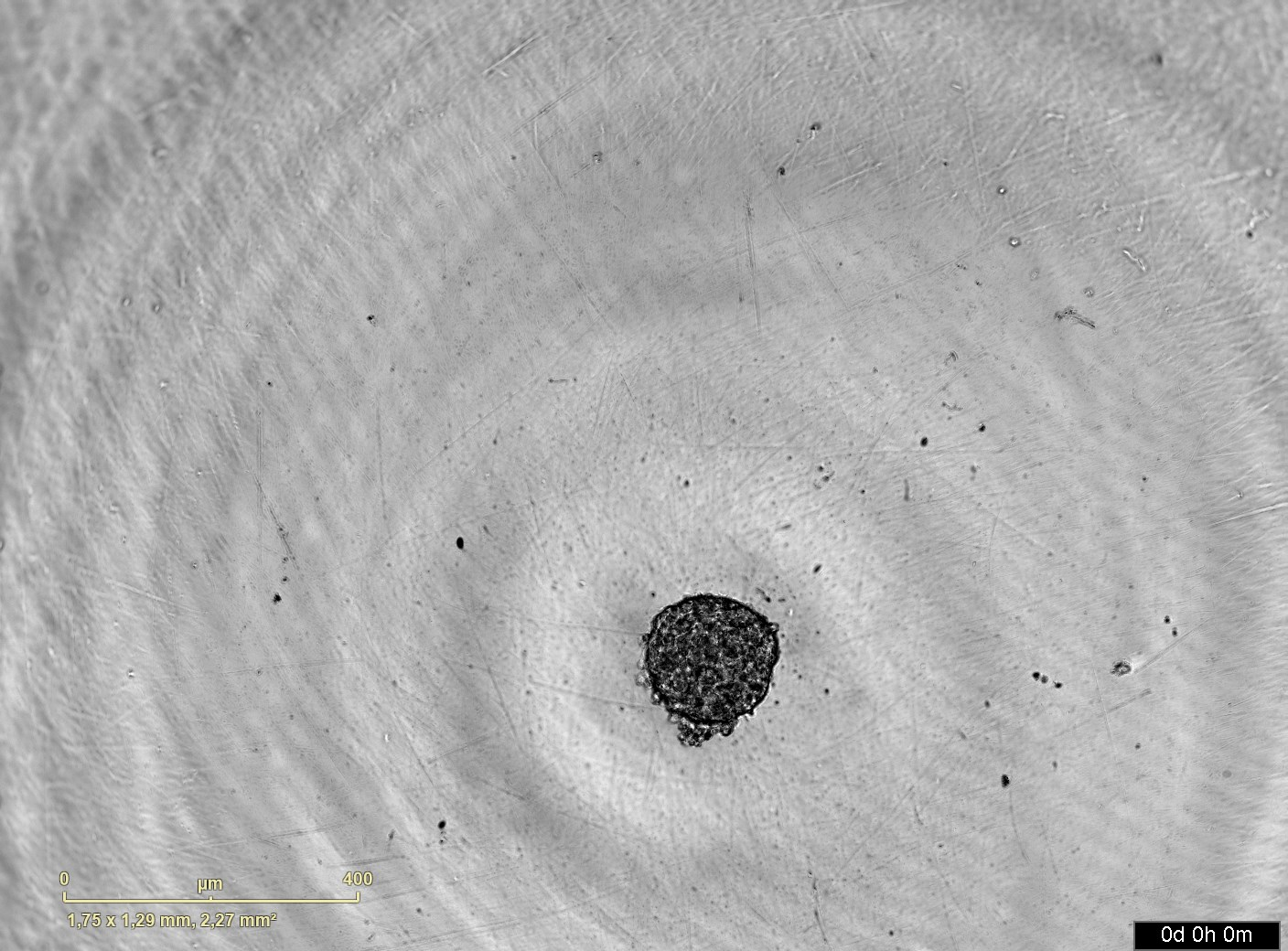

Supplement: Supplementary file 9 — Source data Fig. 2 [file 44321_2026_393_MOESM9_ESM.zip › Figure 2/2C/2C/2C_Abemaciclib_72h_2uM.tiff]

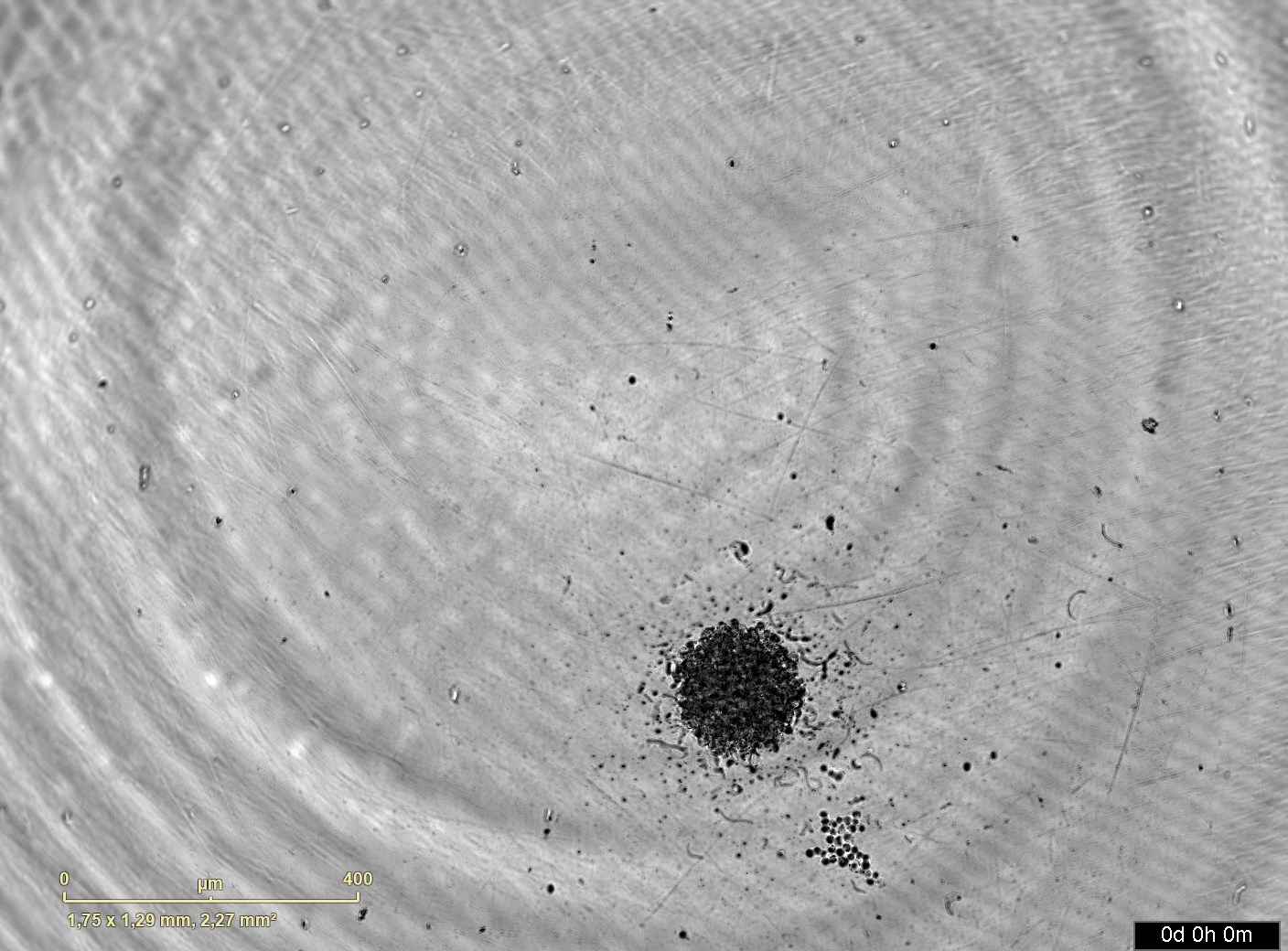

Supplement: Supplementary file 9 — Source data Fig. 2 [file 44321_2026_393_MOESM9_ESM.zip › Figure 2/2C/2C/2C_Abemaciclib_72h_16uM.tiff]

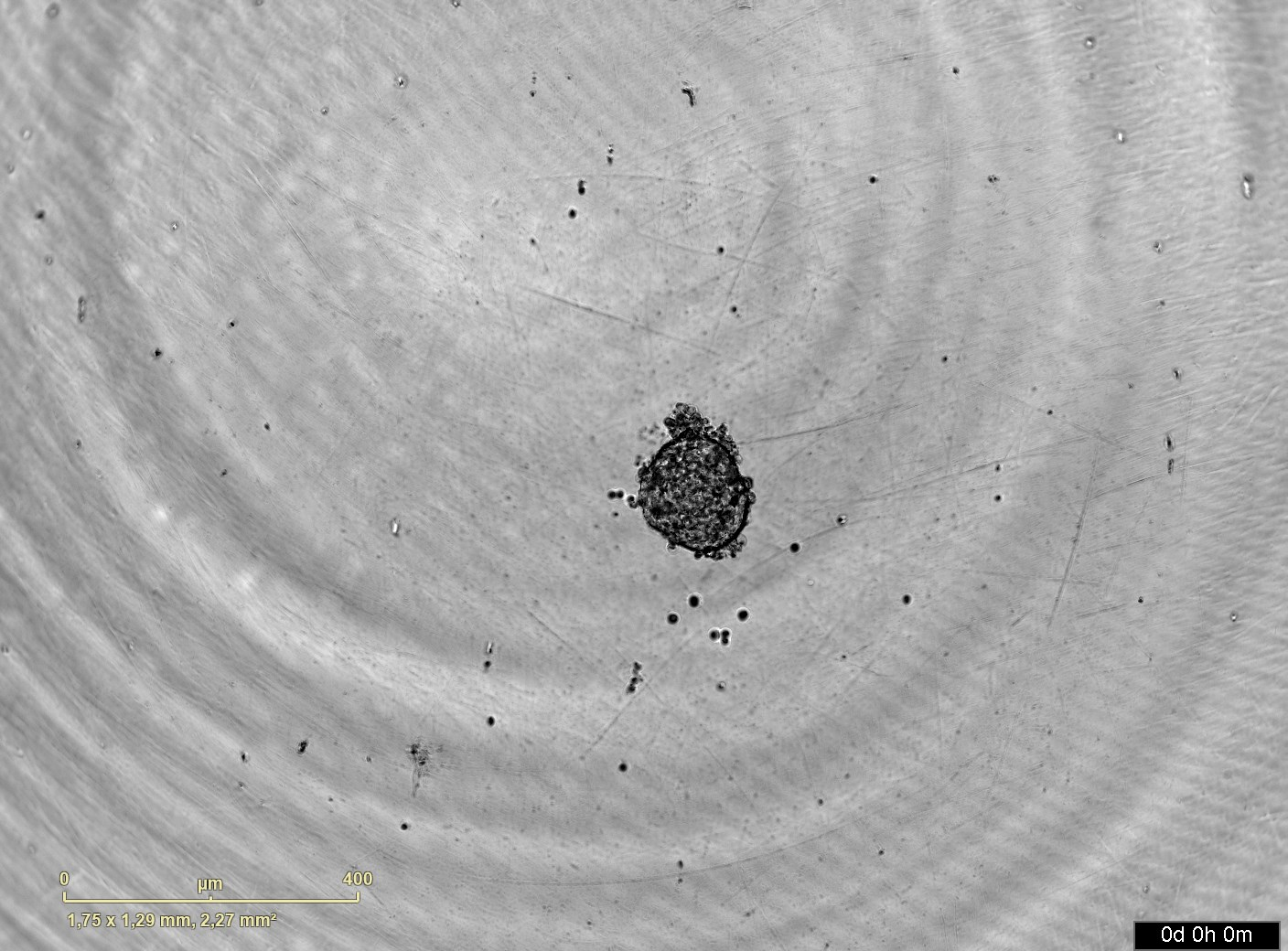

Supplement: Supplementary file 9 — Source data Fig. 2 [file 44321_2026_393_MOESM9_ESM.zip › Figure 2/2C/2C/2C_Abemaciclib_0h_16uM.tiff]

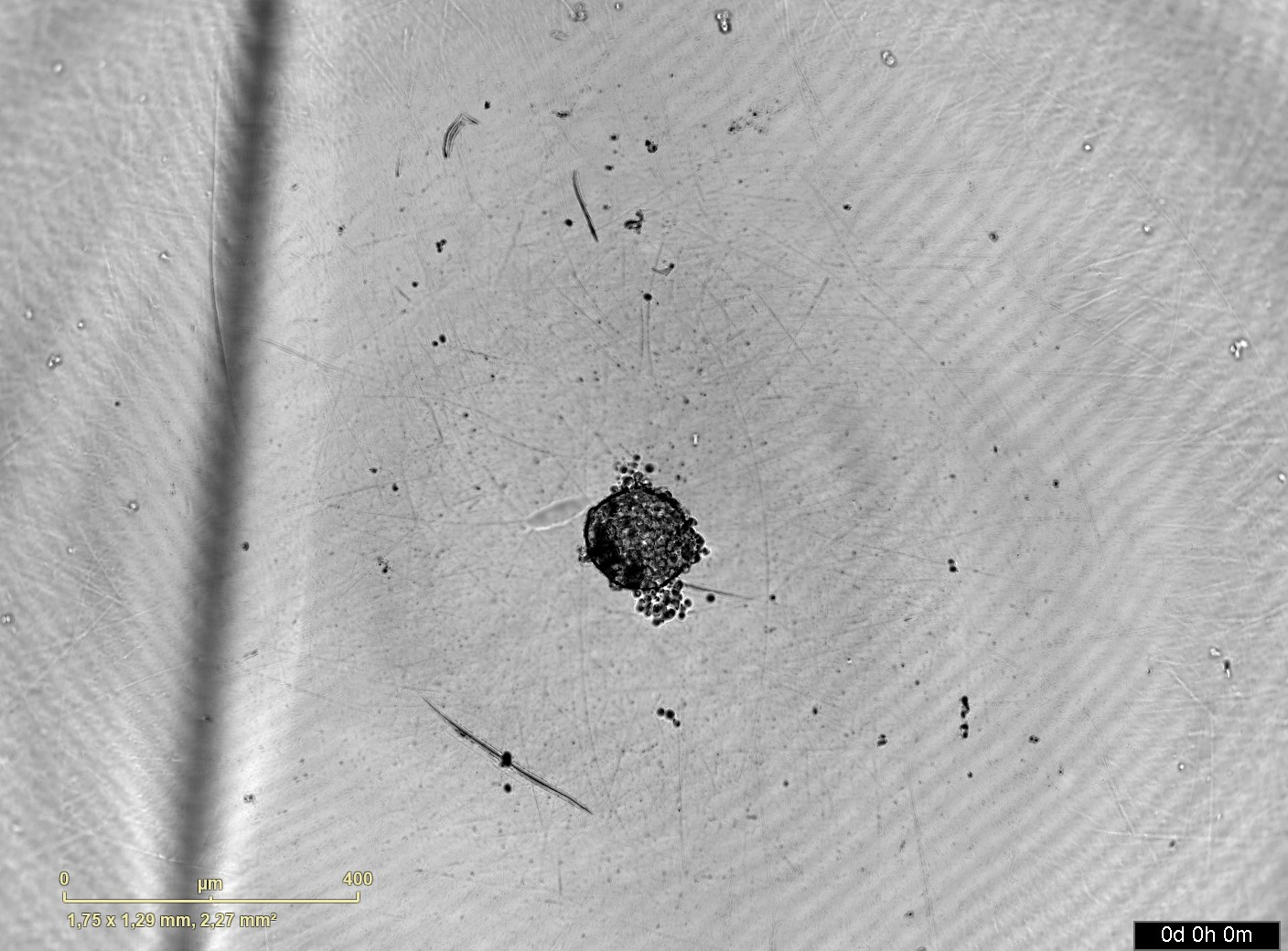

Supplement: Supplementary file 9 — Source data Fig. 2 [file 44321_2026_393_MOESM9_ESM.zip › Figure 2/2C/2C/2C_Abemaciclib_0h_32uM.tiff]

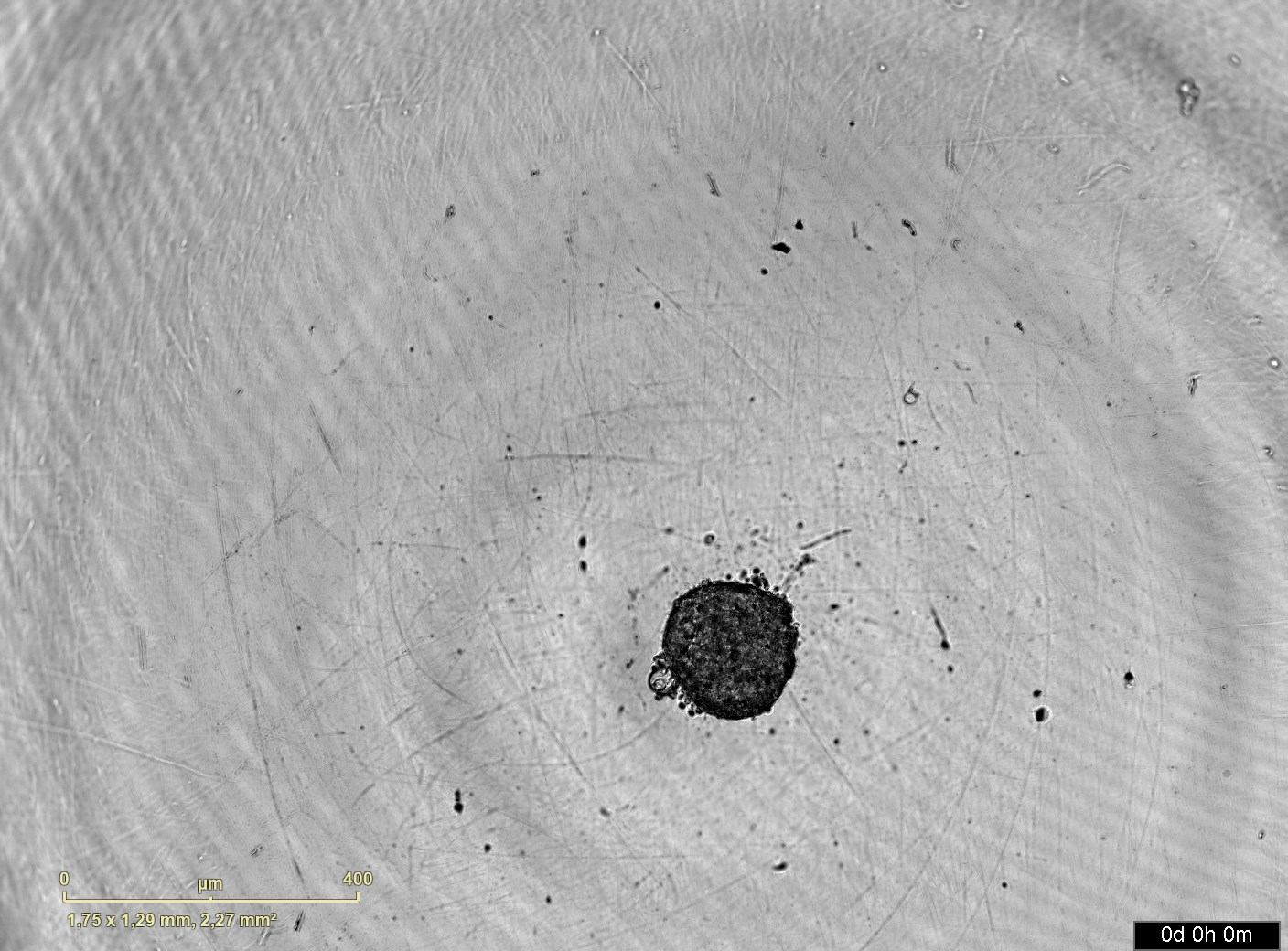

Supplement: Supplementary file 9 — Source data Fig. 2 [file 44321_2026_393_MOESM9_ESM.zip › Figure 2/2C/2C/2C_Abemaciclib_72h_8uM.tiff]

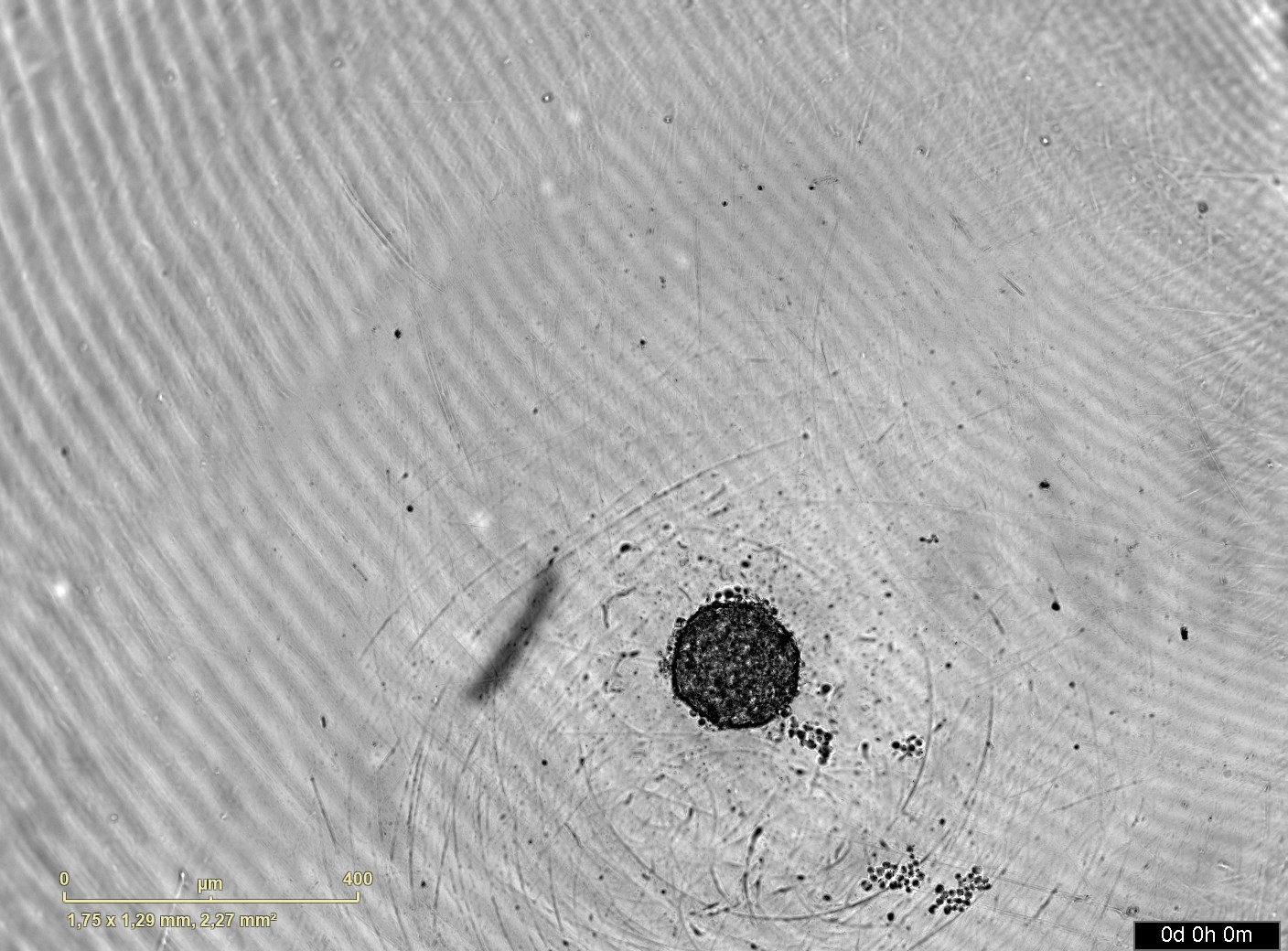

Supplement: Supplementary file 9 — Source data Fig. 2 [file 44321_2026_393_MOESM9_ESM.zip › Figure 2/2C/2C/2C_Abemaciclib_72h_4uM.tiff]

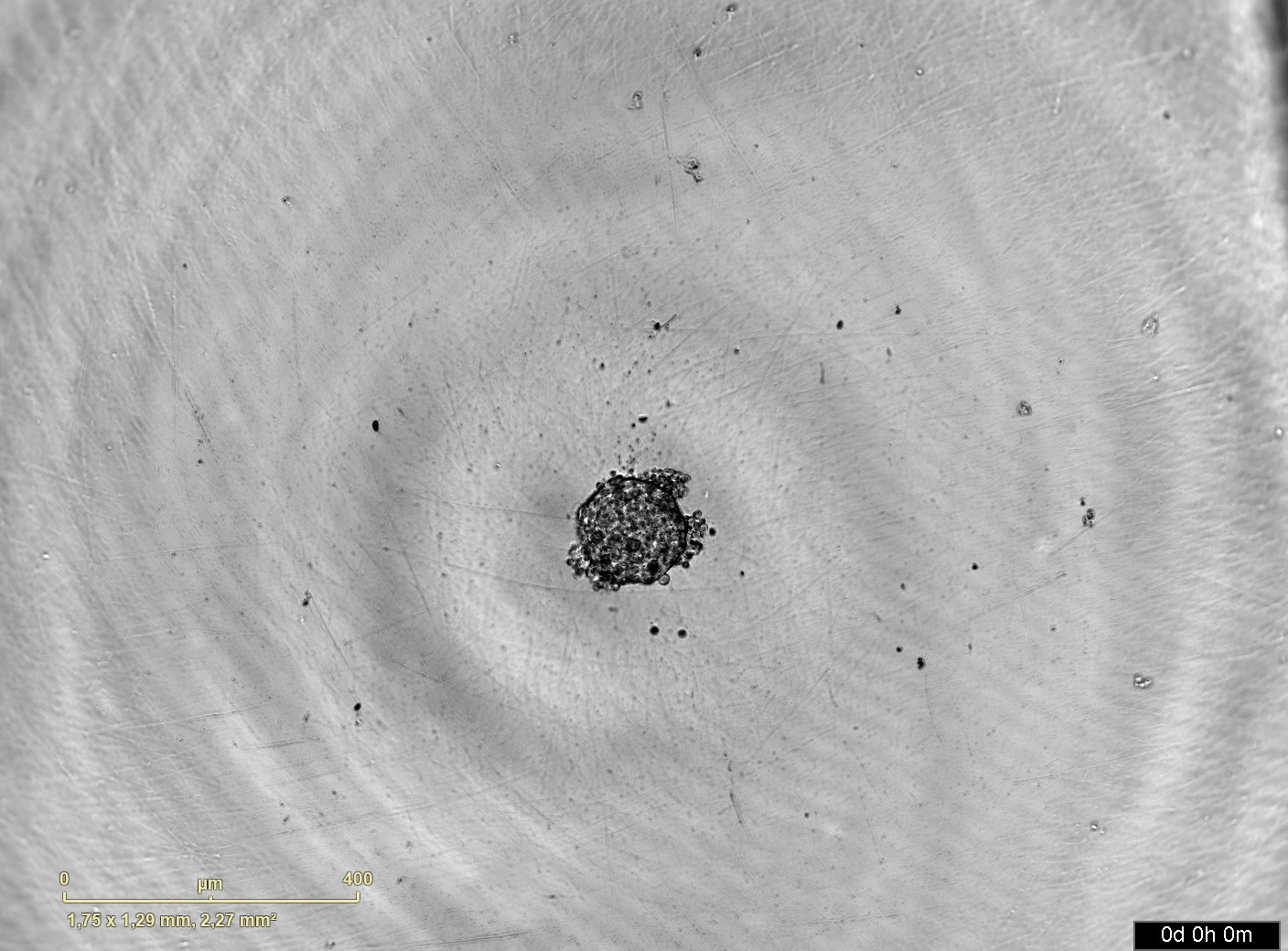

Supplement: Supplementary file 9 — Source data Fig. 2 [file 44321_2026_393_MOESM9_ESM.zip › Figure 2/2C/2C/2C_Abemaciclib_0h_2uM.tiff]

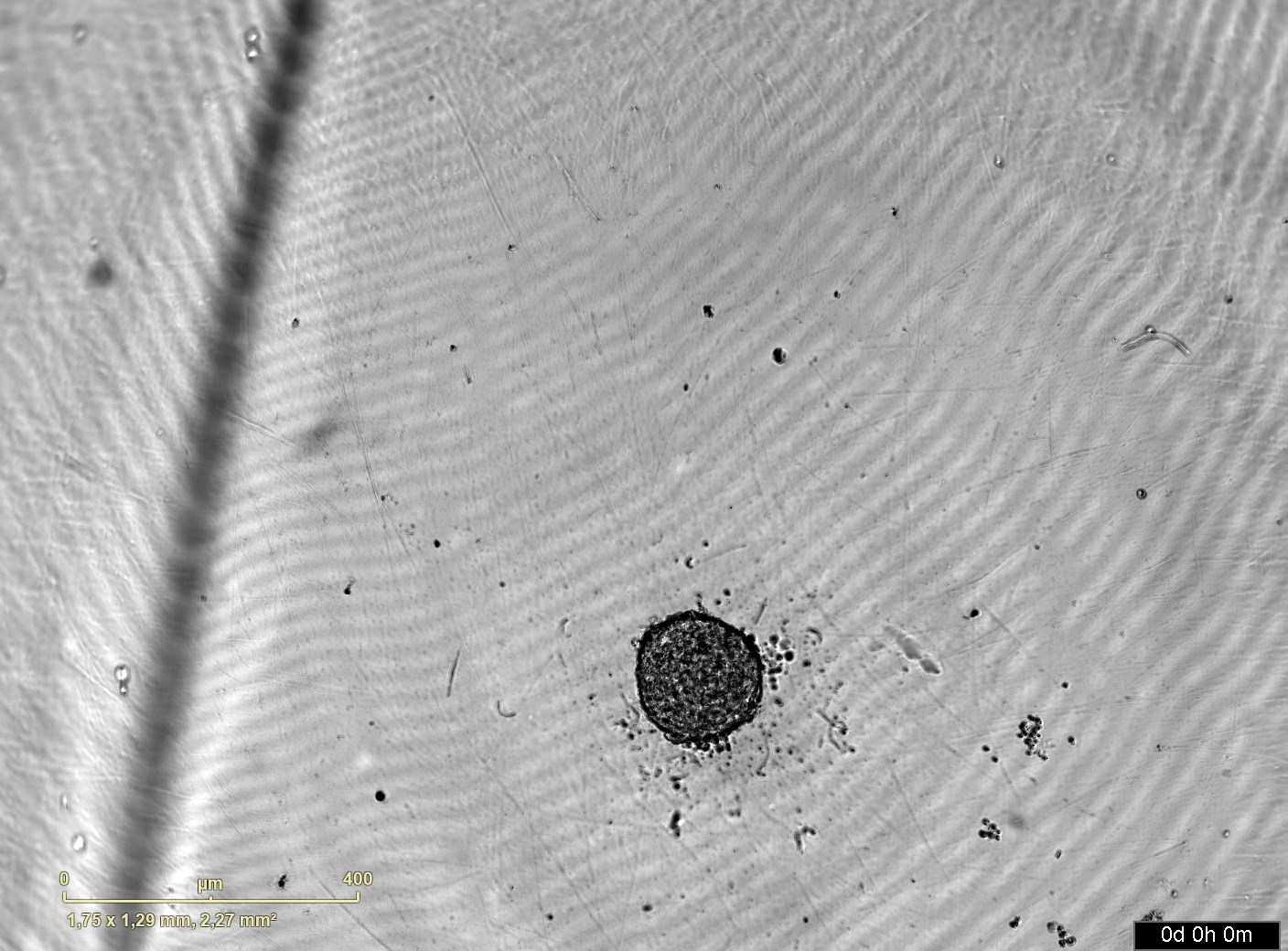

Supplement: Supplementary file 9 — Source data Fig. 2 [file 44321_2026_393_MOESM9_ESM.zip › Figure 2/2C/2C/2C_Abemaciclib_72h_1uM.tiff]

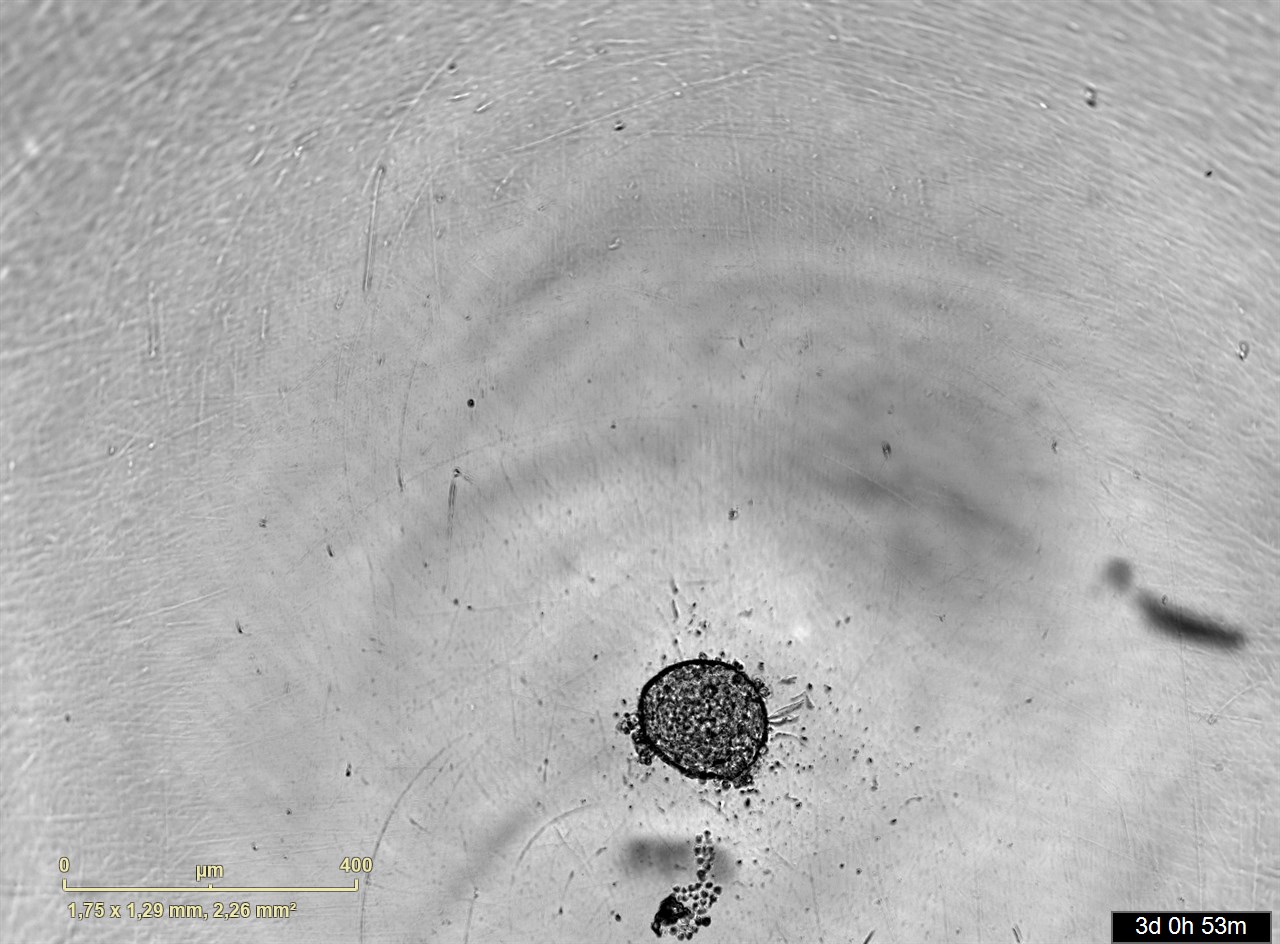

Supplement: Supplementary file 9 — Source data Fig. 2 [file 44321_2026_393_MOESM9_ESM.zip › Figure 2/2C/2C/2C_SR4835_72h_control.jpg]

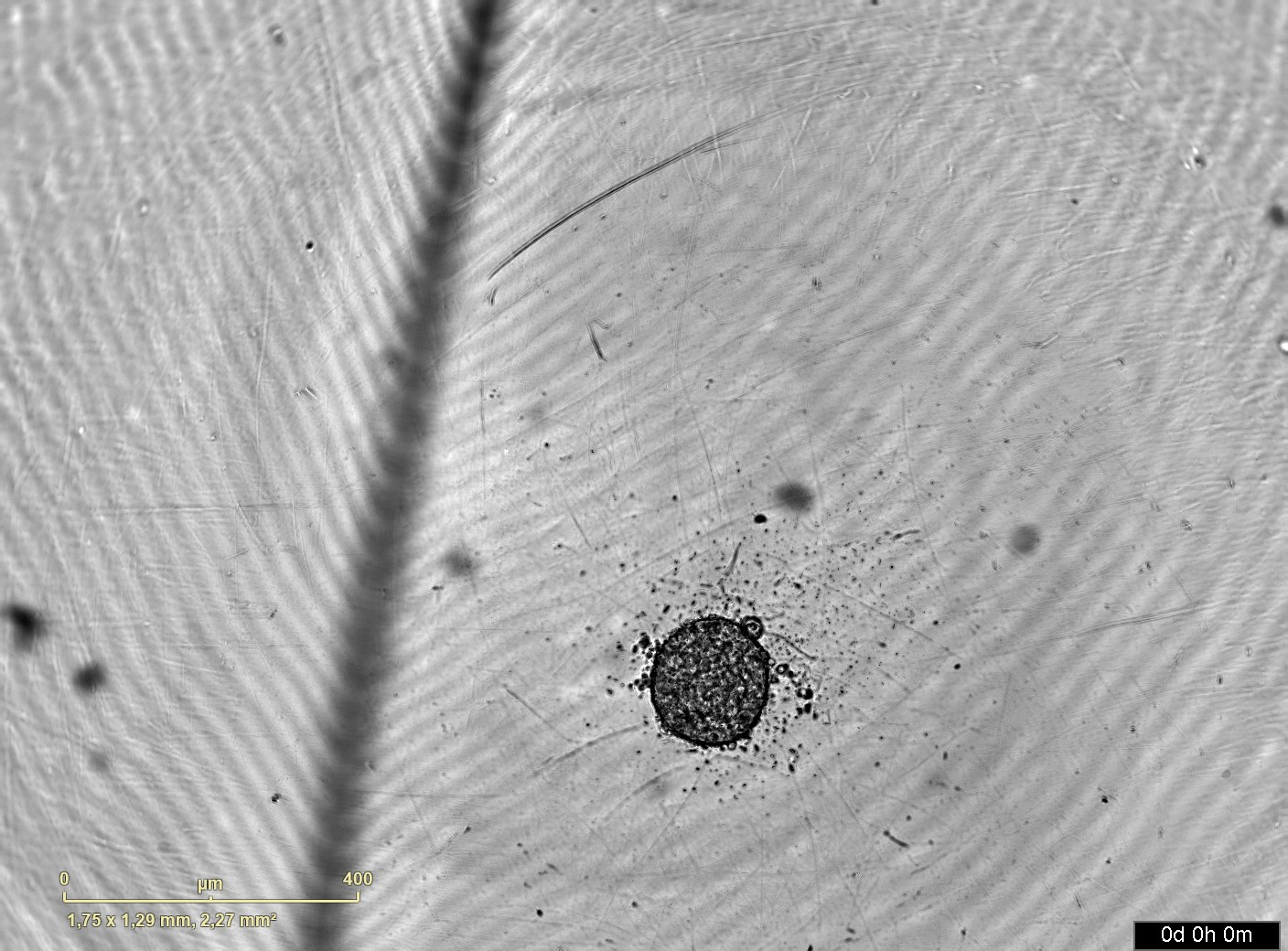

Supplement: Supplementary file 9 — Source data Fig. 2 [file 44321_2026_393_MOESM9_ESM.zip › Figure 2/2C/2C/2C_Abemaciclib_72h_0.5uM.tiff]

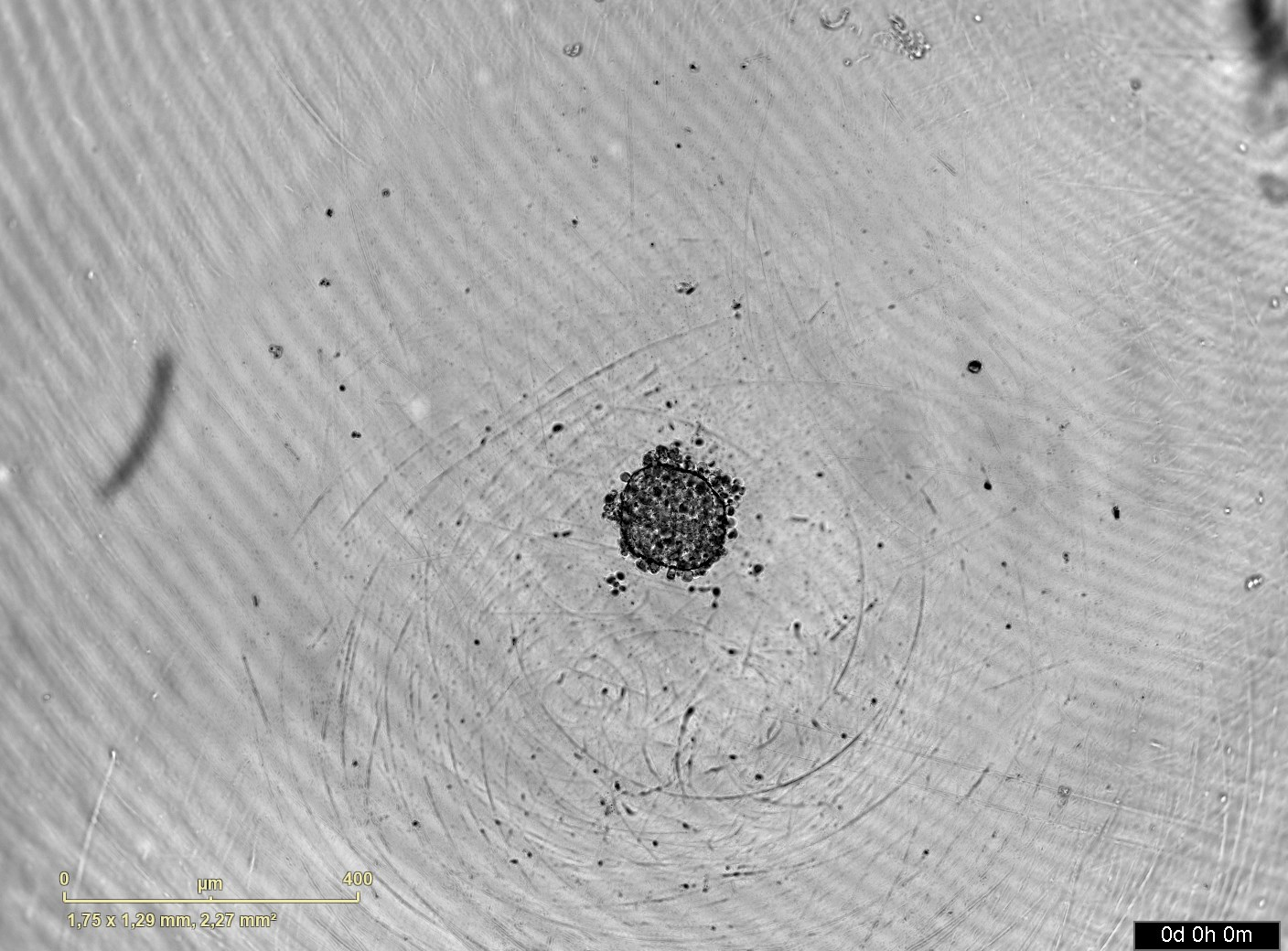

Supplement: Supplementary file 9 — Source data Fig. 2 [file 44321_2026_393_MOESM9_ESM.zip › Figure 2/2C/2C/2C_Abemaciclib_0h_4uM.tiff]

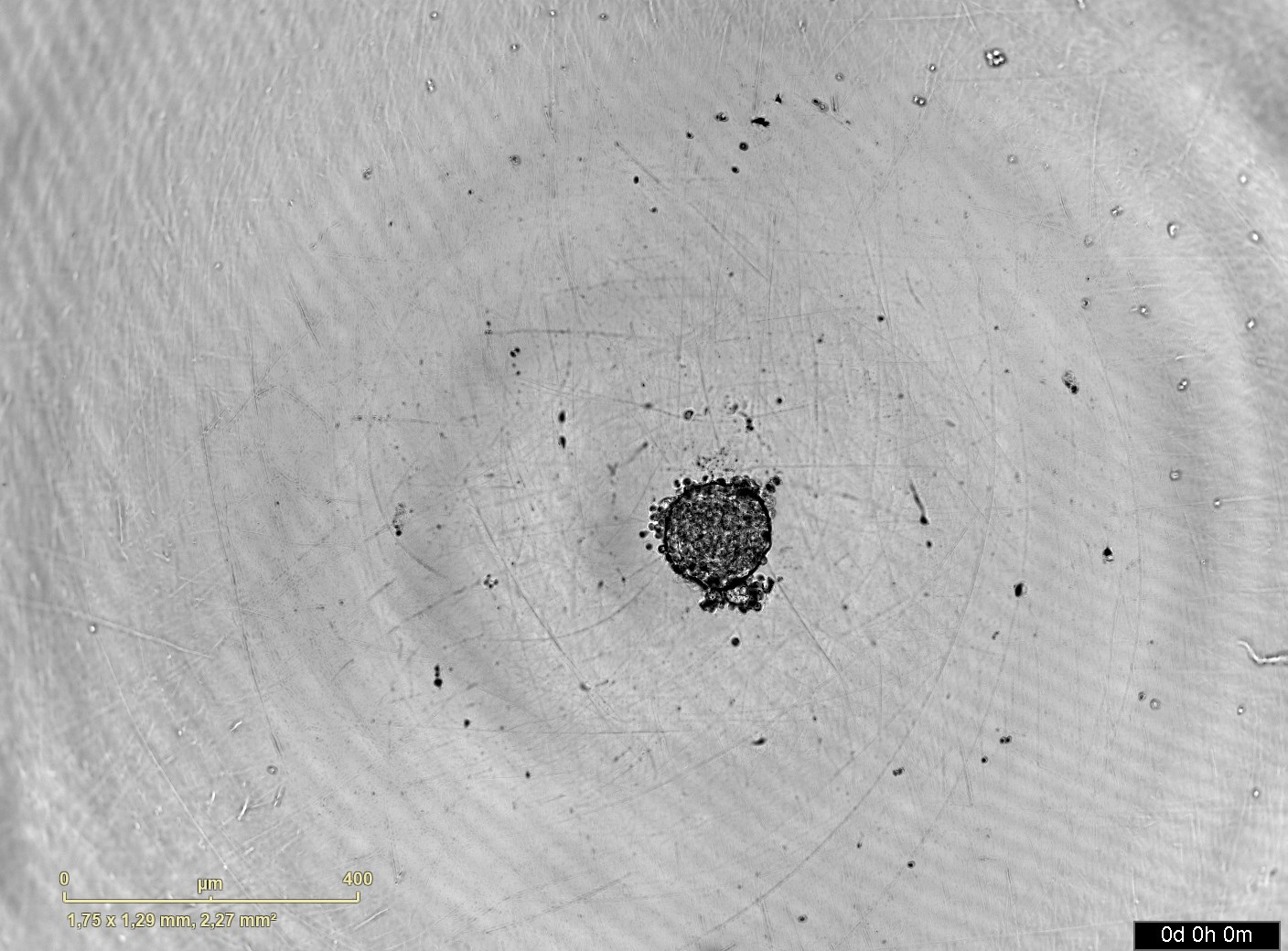

Supplement: Supplementary file 9 — Source data Fig. 2 [file 44321_2026_393_MOESM9_ESM.zip › Figure 2/2C/2C/2C_Abemaciclib_0h_8uM.tiff]

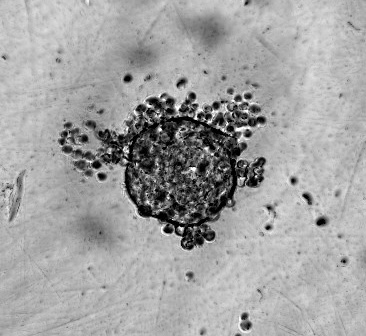

Supplement: Supplementary file 9 — Source data Fig. 2 [file 44321_2026_393_MOESM9_ESM.zip › Figure 2/2C/2C/2C_Abemaciclib_0h_control.jpg]

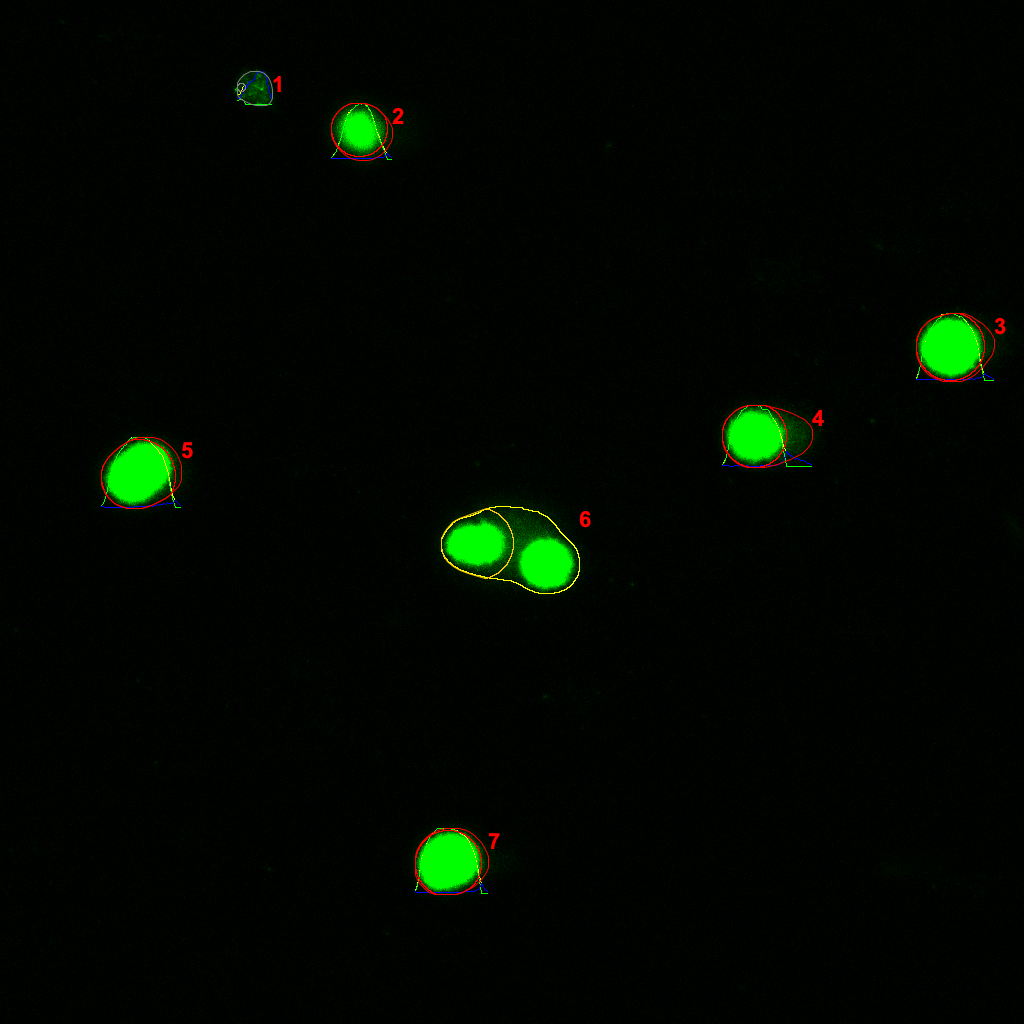

Supplement: Supplementary file 13 — Source data Fig. 6 [file 44321_2026_393_MOESM13_ESM.zip › Figure 6/6B/220915 Comet assay alkaline/output NVP2 6h 1/101_NVP2_6h_10x_Ccenter15_1AUall_rep1_Maximum.ome.tif_out.tif]

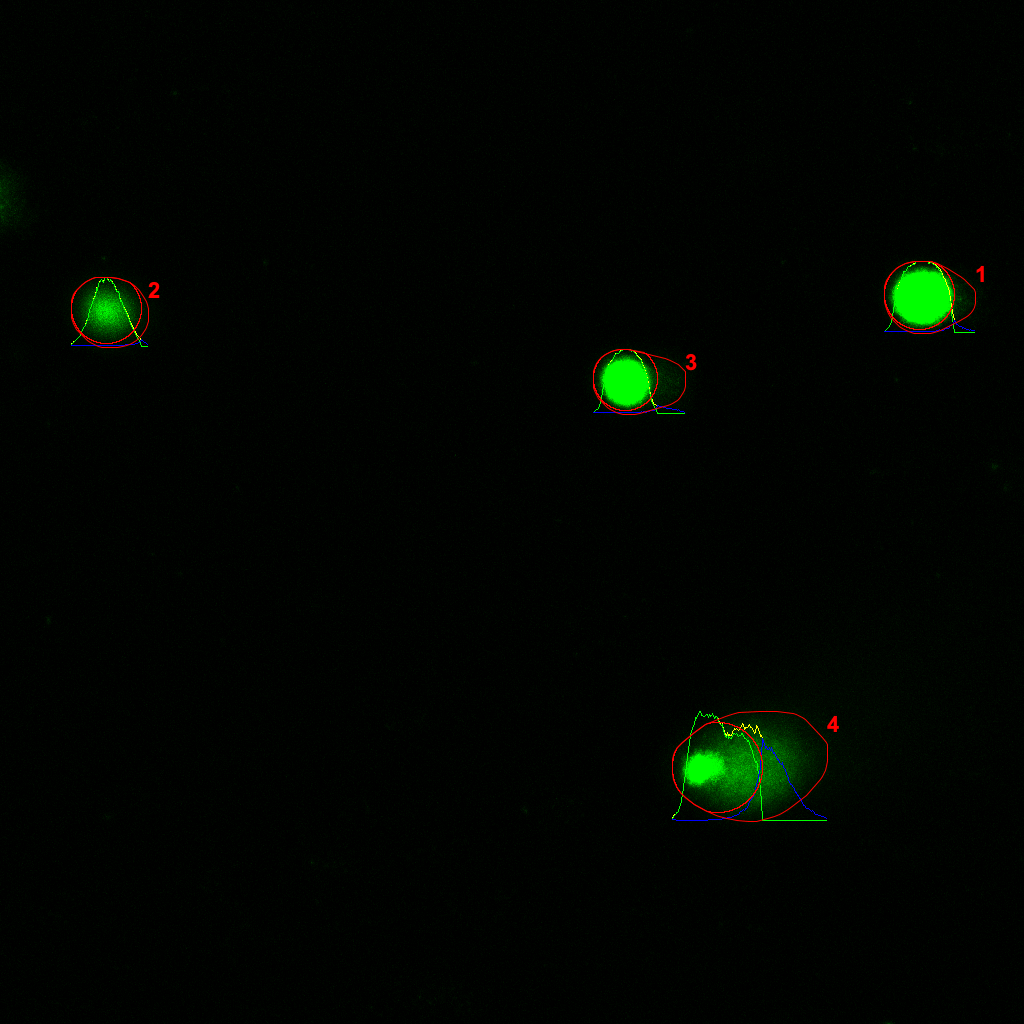

Supplement: Supplementary file 13 — Source data Fig. 6 [file 44321_2026_393_MOESM13_ESM.zip › Figure 6/6B/220915 Comet assay alkaline/output NVP2 6h 1/102_NVP2_6h_10x_Ccenter15_1AUall_rep1_Maximum.ome.tif_out.tif]

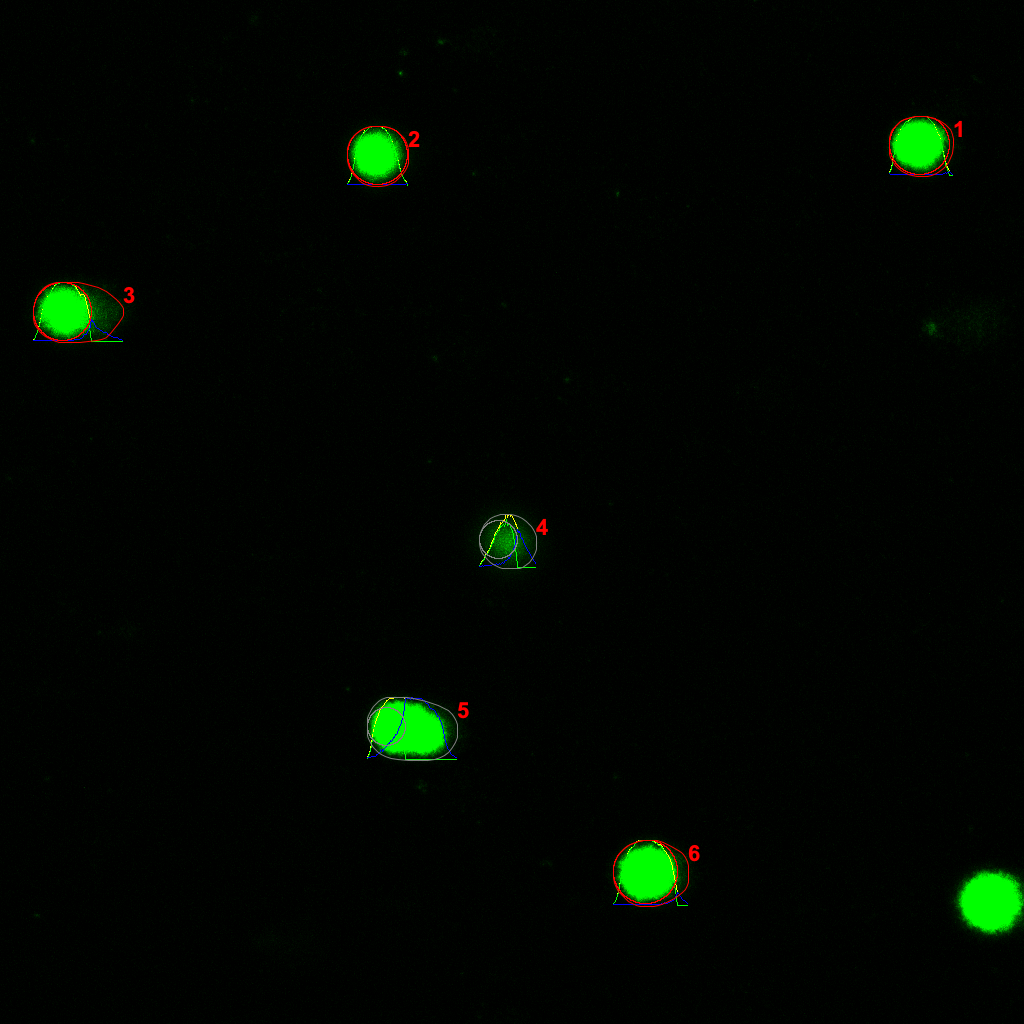

Supplement: Supplementary file 13 — Source data Fig. 6 [file 44321_2026_393_MOESM13_ESM.zip › Figure 6/6B/220915 Comet assay alkaline/output NVP2 6h 1/110_NVP2_6h_10x_Ccenter15_1AUall_rep1_Maximum.ome.tif_out.tif]

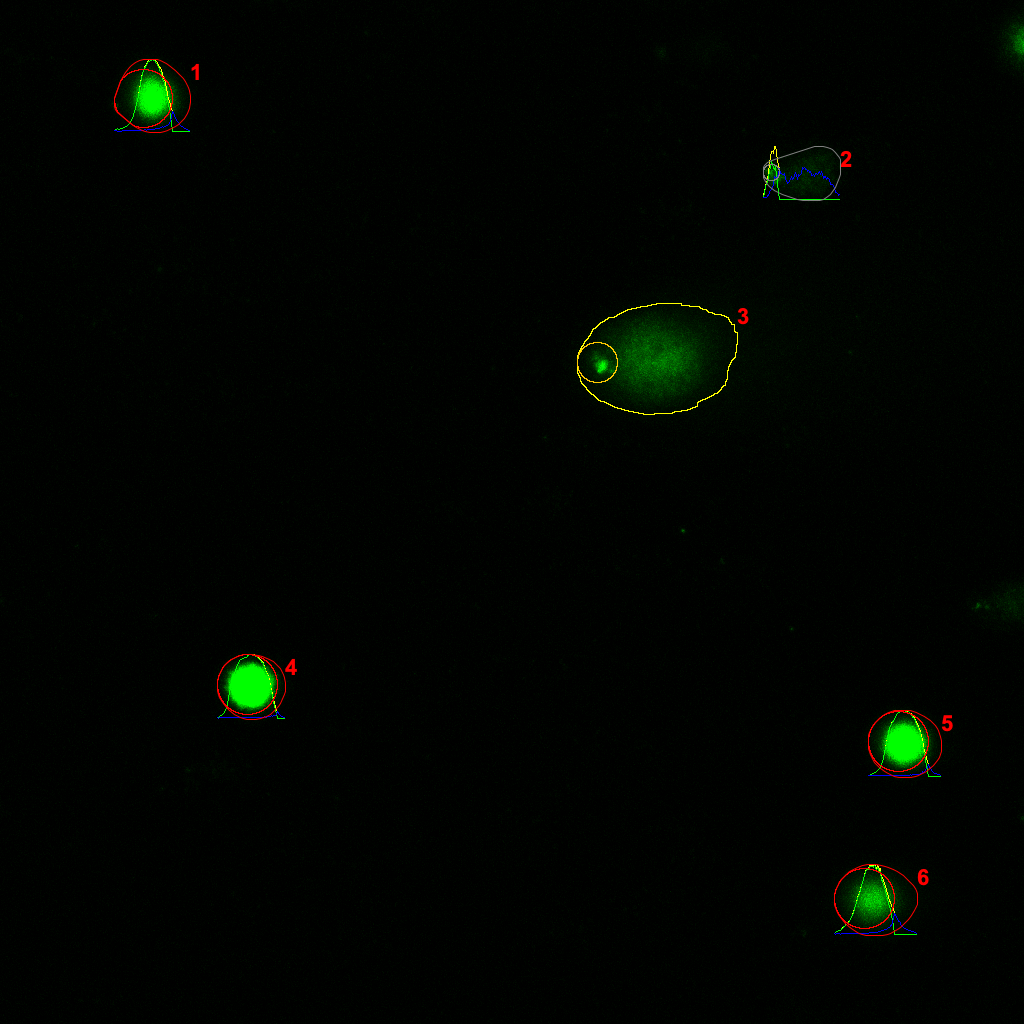

Supplement: Supplementary file 13 — Source data Fig. 6 [file 44321_2026_393_MOESM13_ESM.zip › Figure 6/6B/220915 Comet assay alkaline/output NVP2 6h 1/103_NVP2_6h_10x_Ccenter15_1AUall_rep1_Maximum.ome.tif_out.tif]

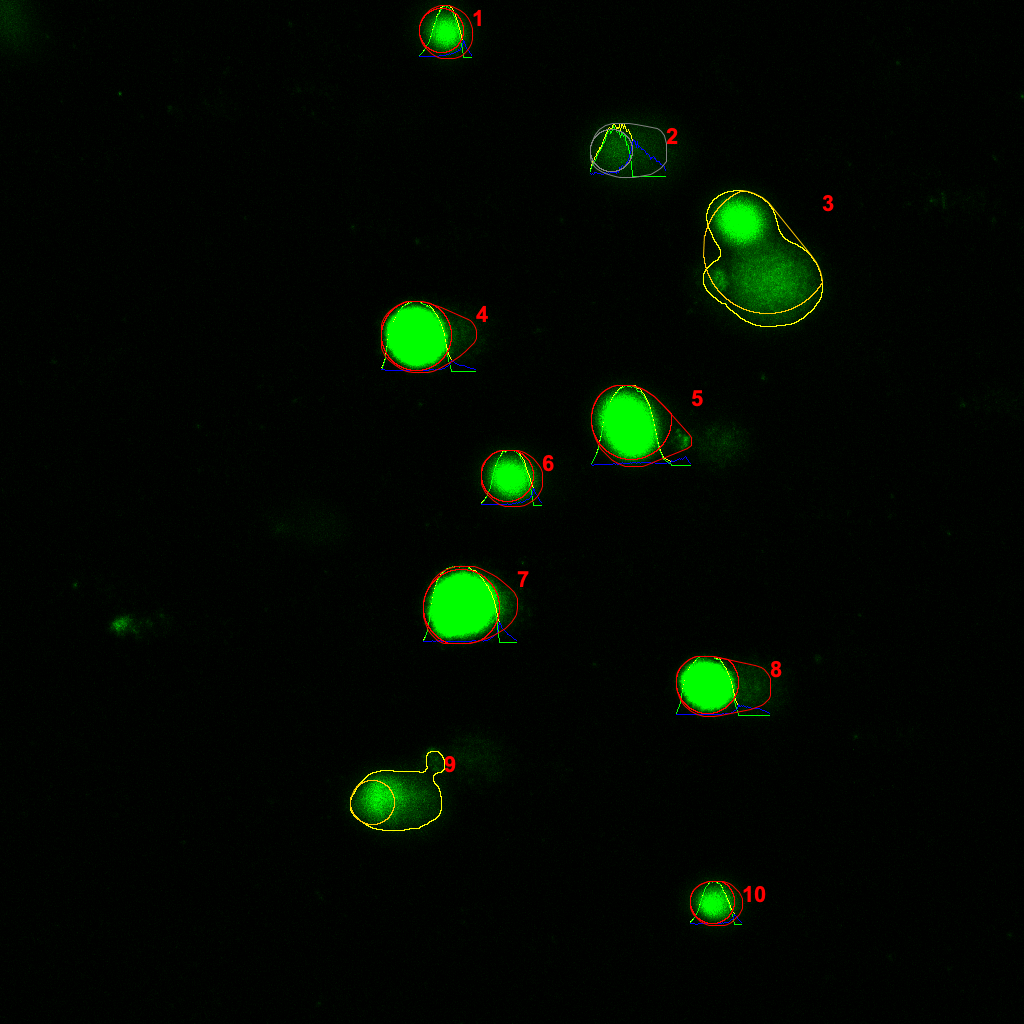

Supplement: Supplementary file 13 — Source data Fig. 6 [file 44321_2026_393_MOESM13_ESM.zip › Figure 6/6B/220915 Comet assay alkaline/output NVP2 6h 1/108_NVP2_6h_10x_Ccenter15_1AUall_rep1_Maximum.ome.tif_out.tif]

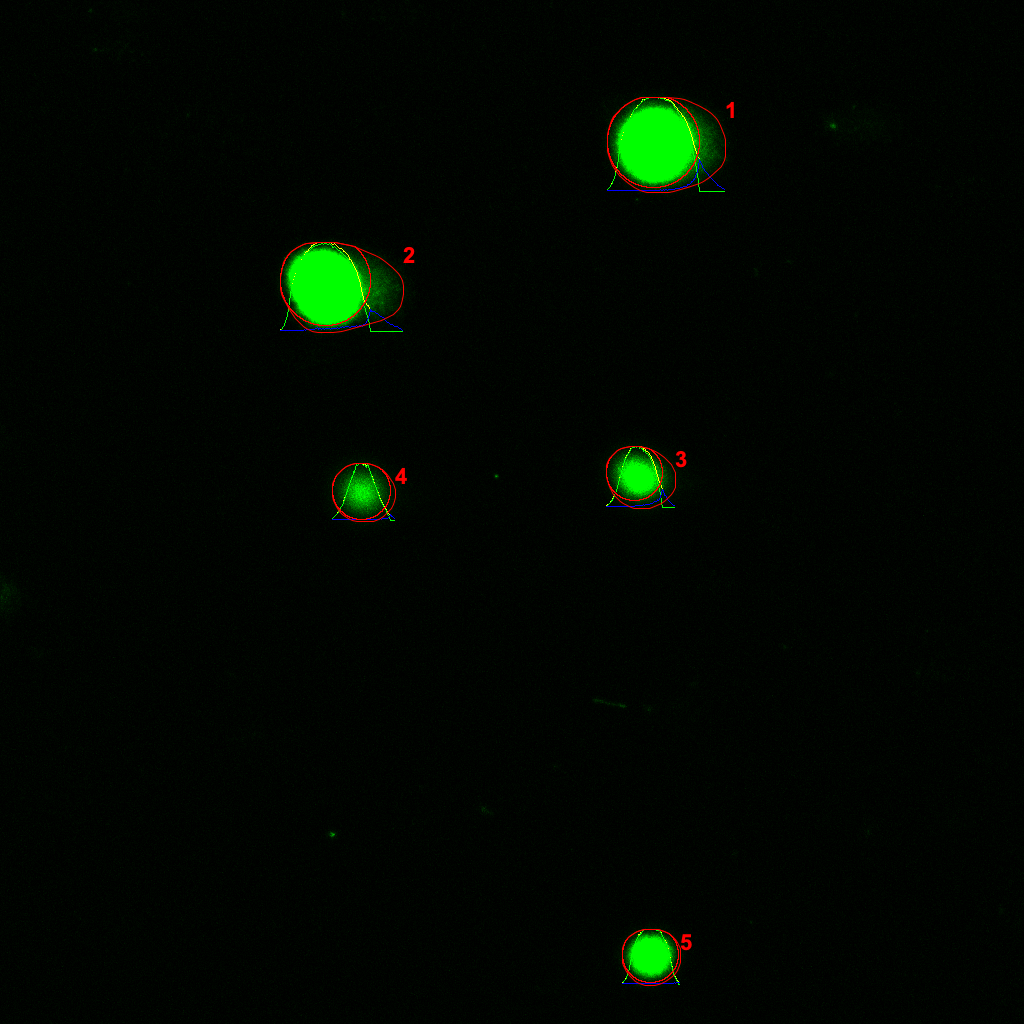

Supplement: Supplementary file 13 — Source data Fig. 6 [file 44321_2026_393_MOESM13_ESM.zip › Figure 6/6B/220915 Comet assay alkaline/output NVP2 6h 1/106_NVP2_6h_10x_Ccenter15_1AUall_rep1_Maximum.ome.tif_out.tif]

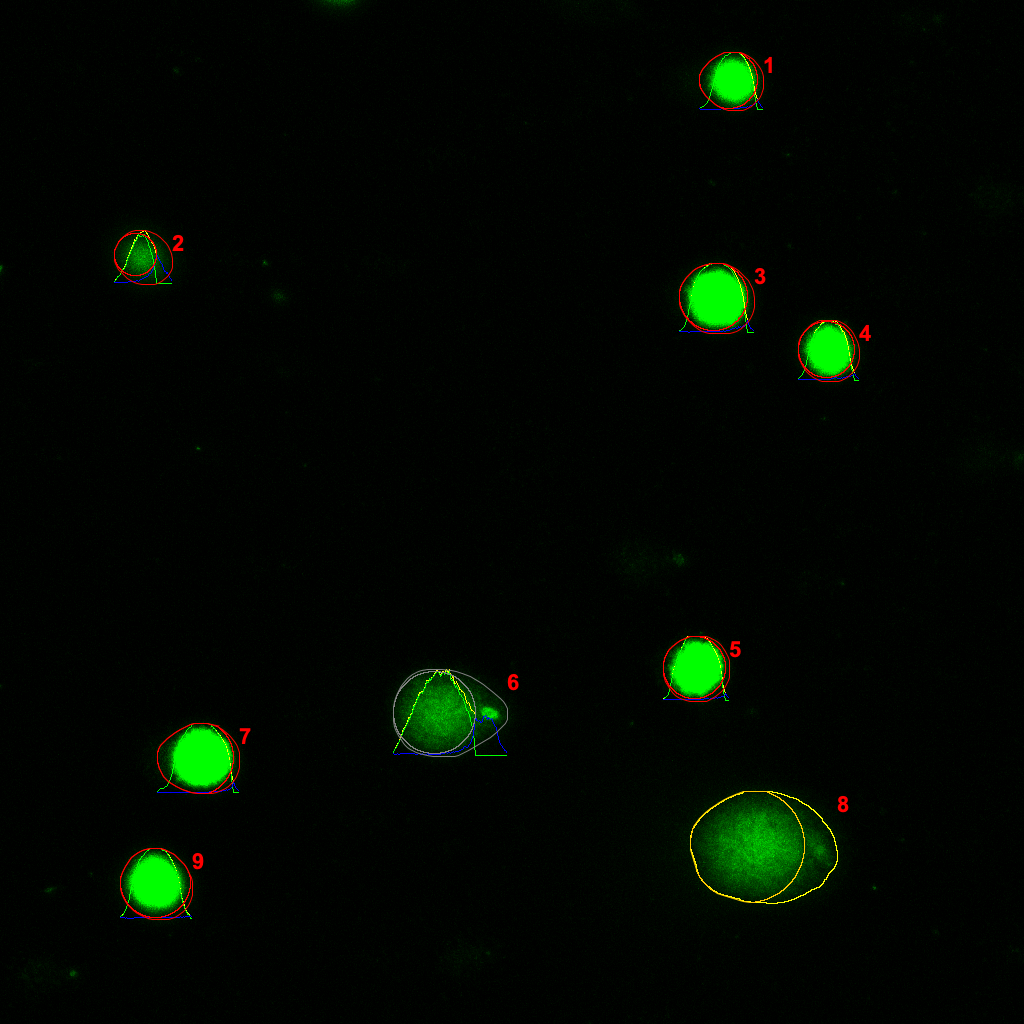

Supplement: Supplementary file 13 — Source data Fig. 6 [file 44321_2026_393_MOESM13_ESM.zip › Figure 6/6B/220915 Comet assay alkaline/output NVP2 6h 1/109_NVP2_6h_10x_Ccenter15_1AUall_rep1_Maximum.ome.tif_out.tif]

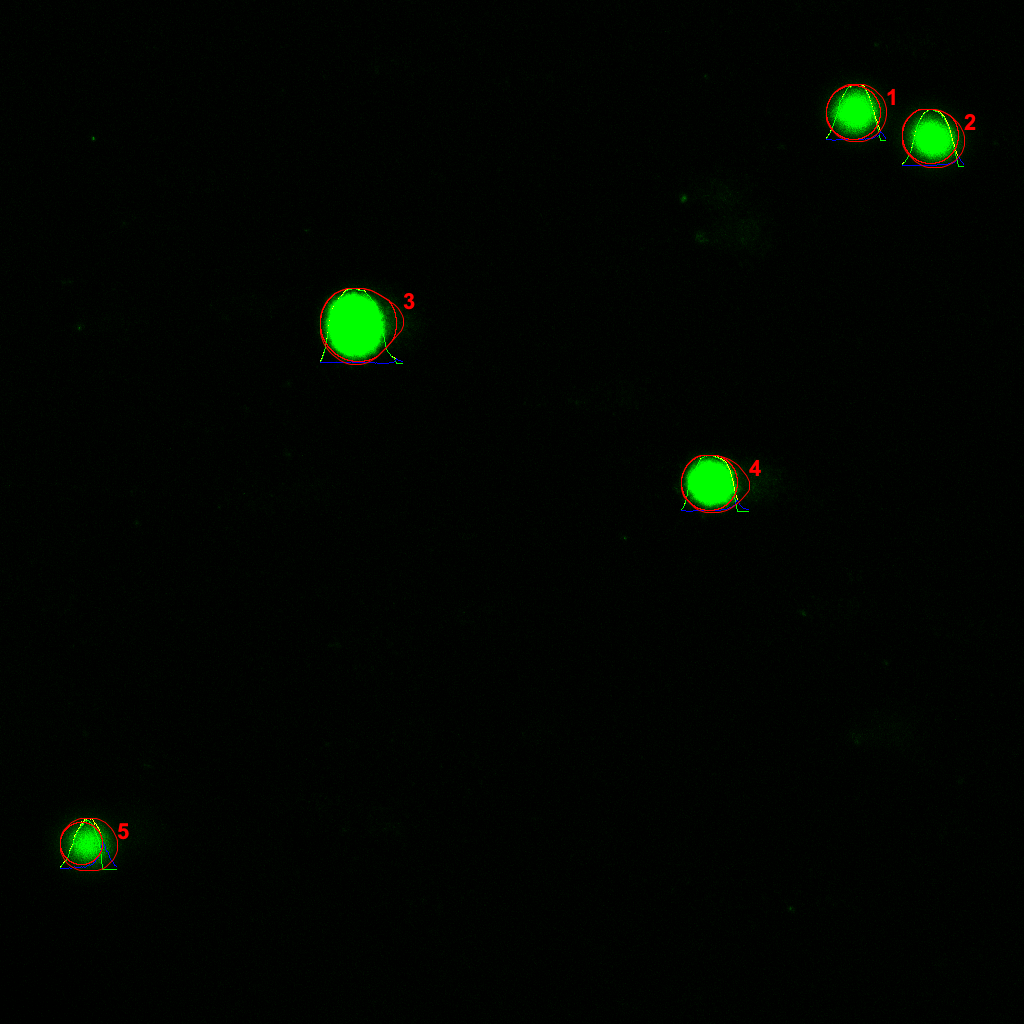

Supplement: Supplementary file 13 — Source data Fig. 6 [file 44321_2026_393_MOESM13_ESM.zip › Figure 6/6B/220915 Comet assay alkaline/output NVP2 6h 1/107_NVP2_6h_10x_Ccenter15_1AUall_rep1_Maximum.ome.tif_out.tif]

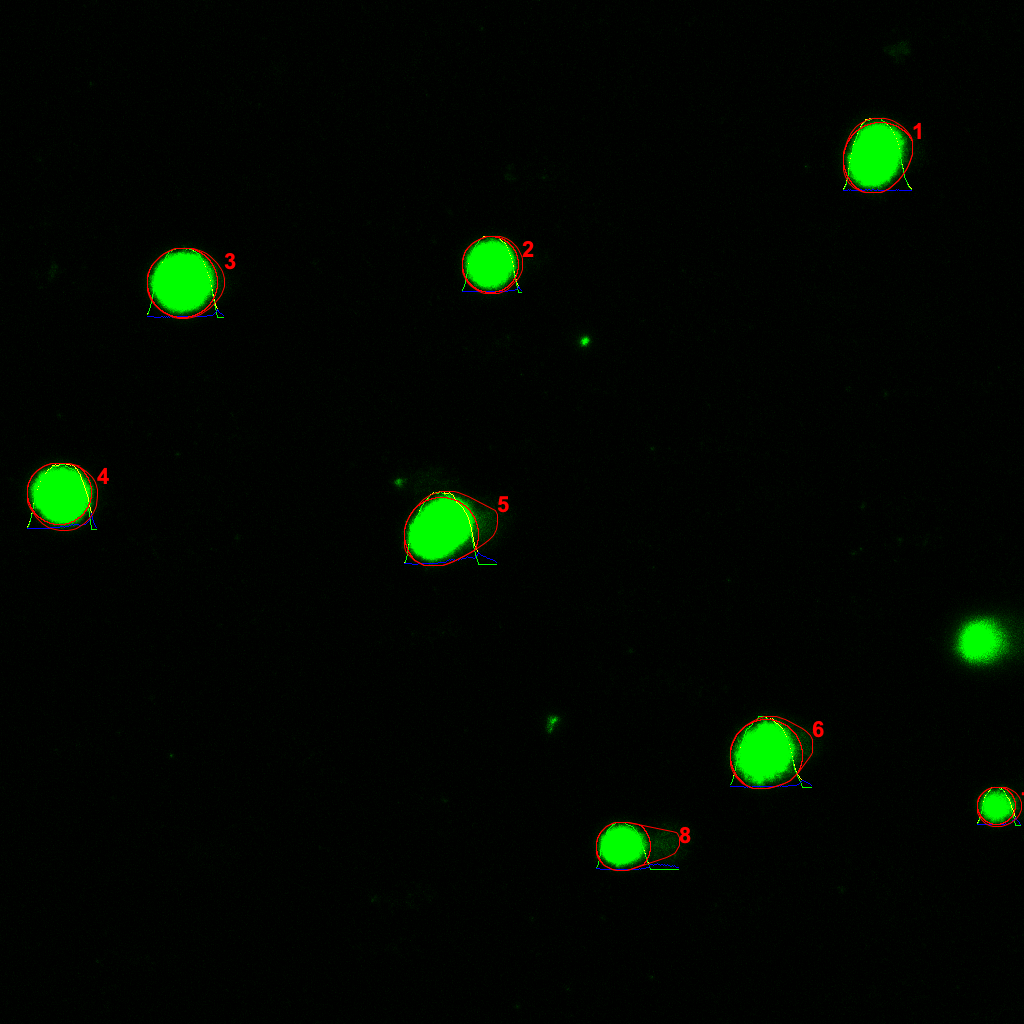

Supplement: Supplementary file 13 — Source data Fig. 6 [file 44321_2026_393_MOESM13_ESM.zip › Figure 6/6B/220915 Comet assay alkaline/output NVP2 6h 1/105_NVP2_6h_10x_Ccenter15_1AUall_rep1_Maximum.ome.tif_out.tif]

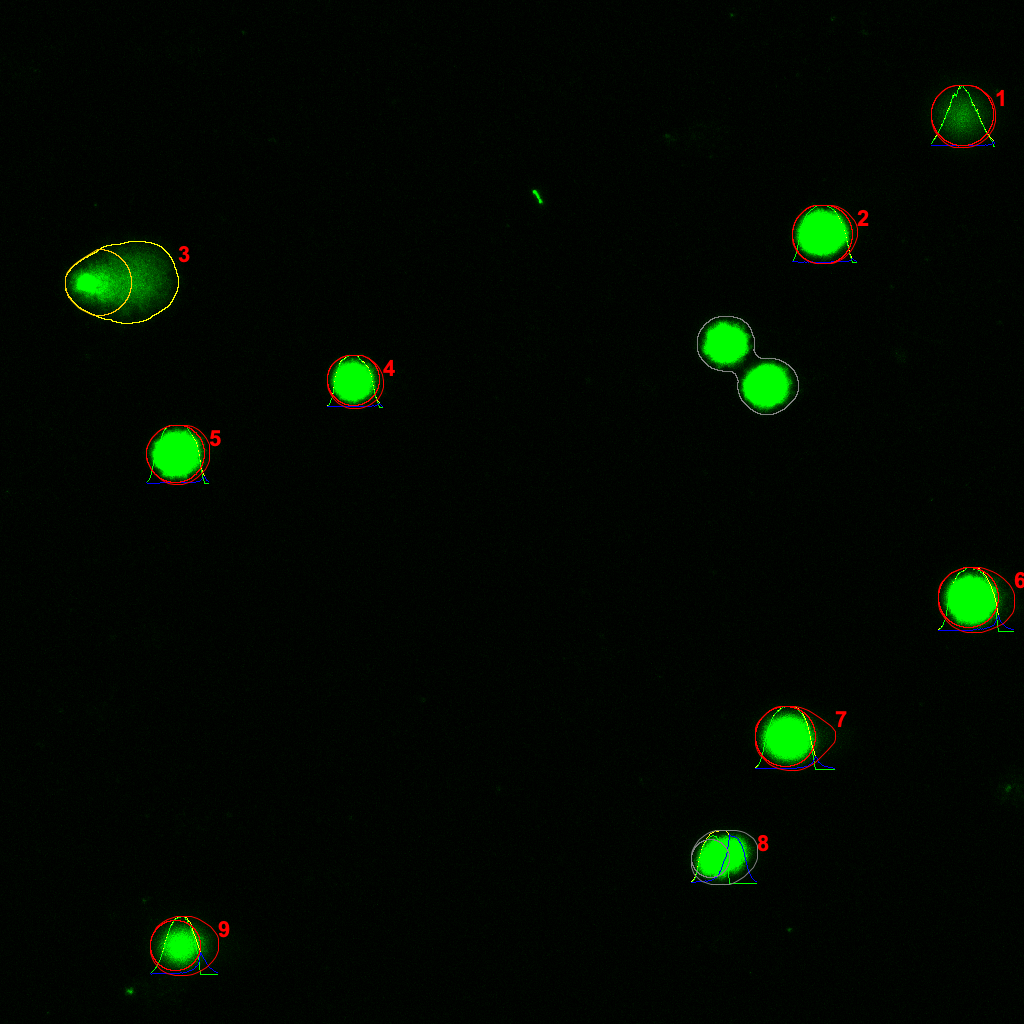

Supplement: Supplementary file 13 — Source data Fig. 6 [file 44321_2026_393_MOESM13_ESM.zip › Figure 6/6B/220915 Comet assay alkaline/output NVP2 6h 1/104_NVP2_6h_10x_Ccenter15_1AUall_rep1_Maximum.ome.tif_out.tif]

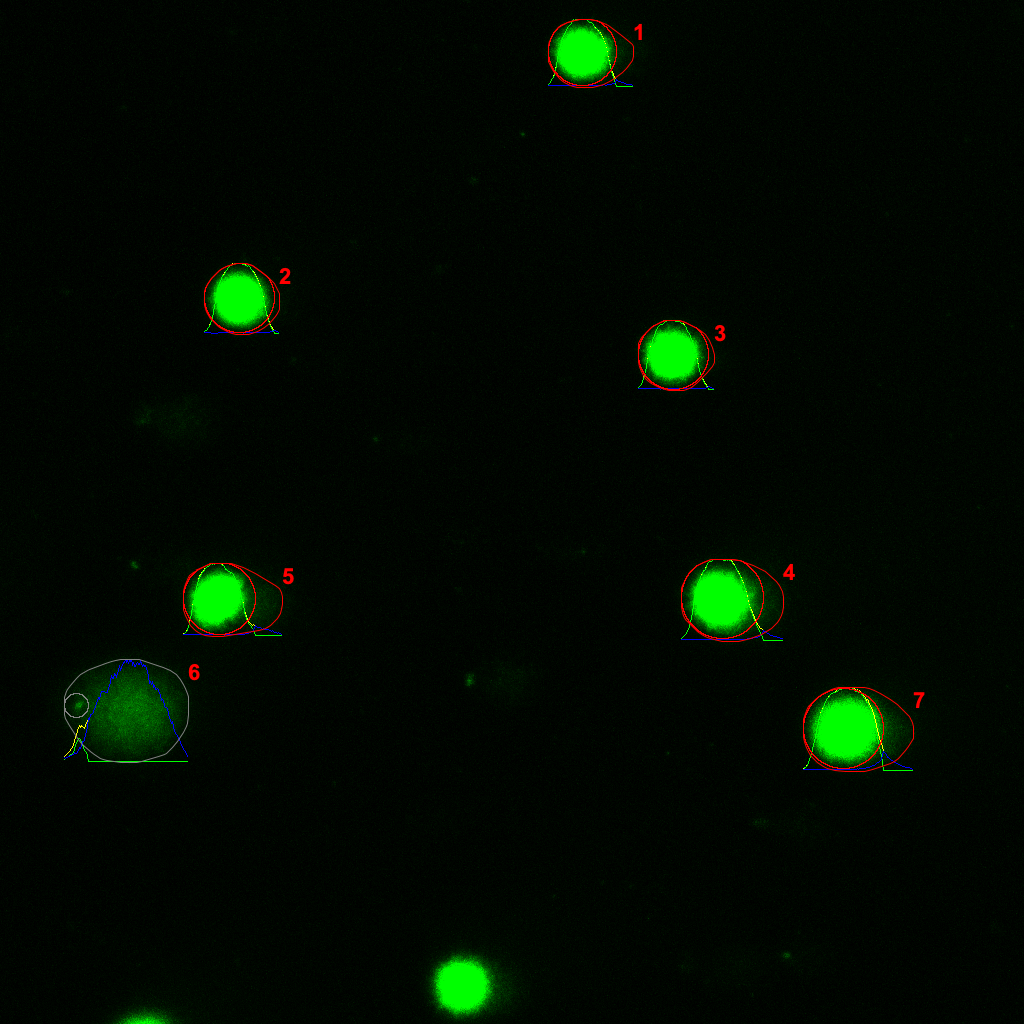

Supplement: Supplementary file 13 — Source data Fig. 6 [file 44321_2026_393_MOESM13_ESM.zip › Figure 6/6B/220915 Comet assay alkaline/output THZ531 6h 1/61_THZ531_6h_10x_Ccenter15_1AUall_rep1_Maximum.ome.tif_out.tif]

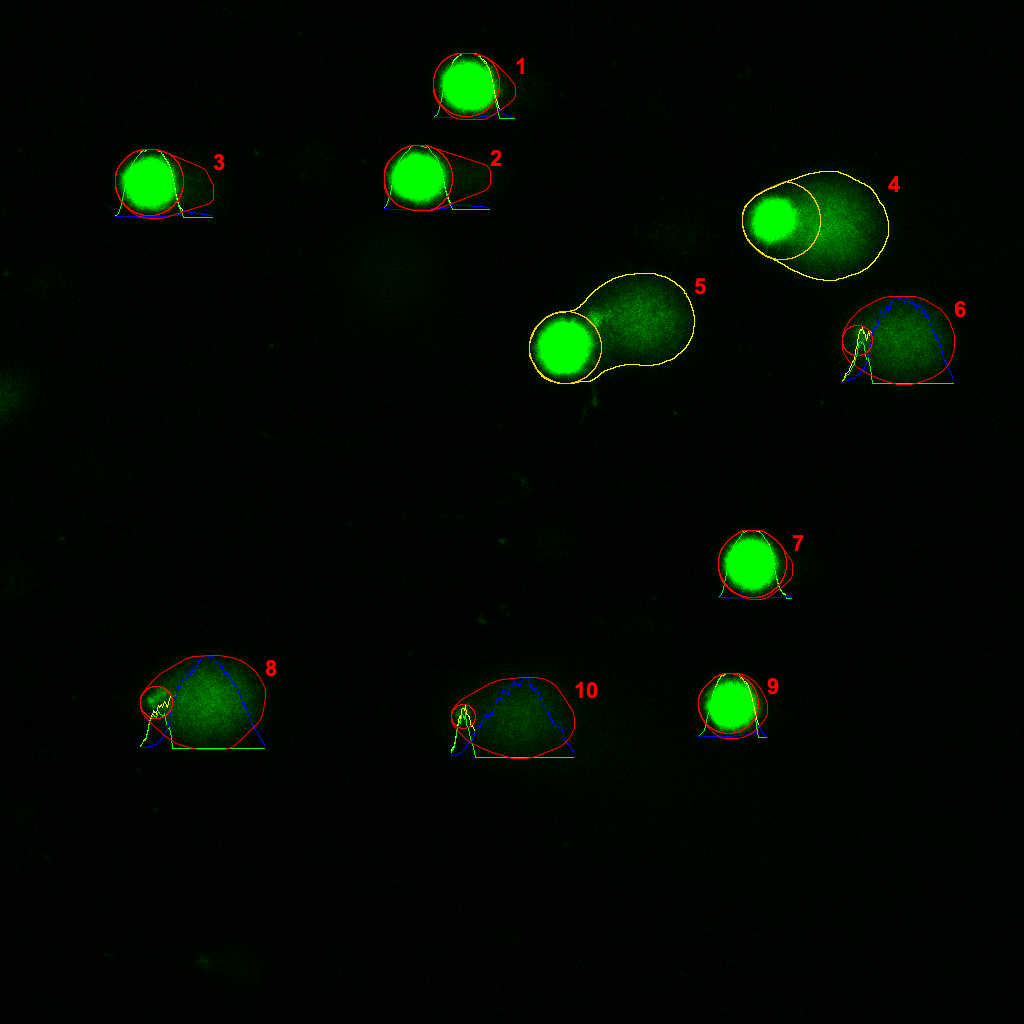

Supplement: Supplementary file 13 — Source data Fig. 6 [file 44321_2026_393_MOESM13_ESM.zip › Figure 6/6B/220915 Comet assay alkaline/output THZ531 6h 1/63_THZ531_6h_10x_Ccenter15_1AUall_rep1_Maximum.ome.tif_out.tif]

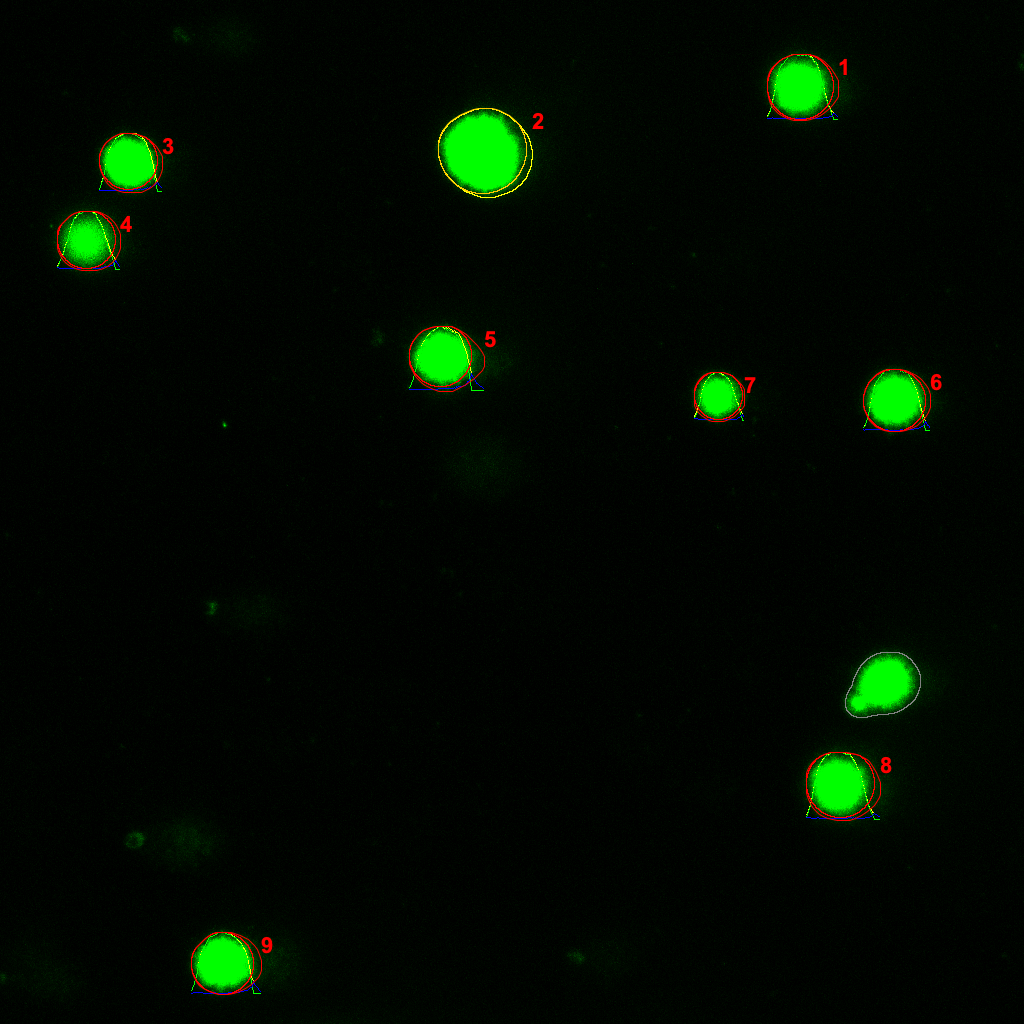

Supplement: Supplementary file 13 — Source data Fig. 6 [file 44321_2026_393_MOESM13_ESM.zip › Figure 6/6B/220915 Comet assay alkaline/output THZ531 6h 1/62_THZ531_6h_10x_Ccenter15_1AUall_rep1_Maximum.ome.tif_out.tif]

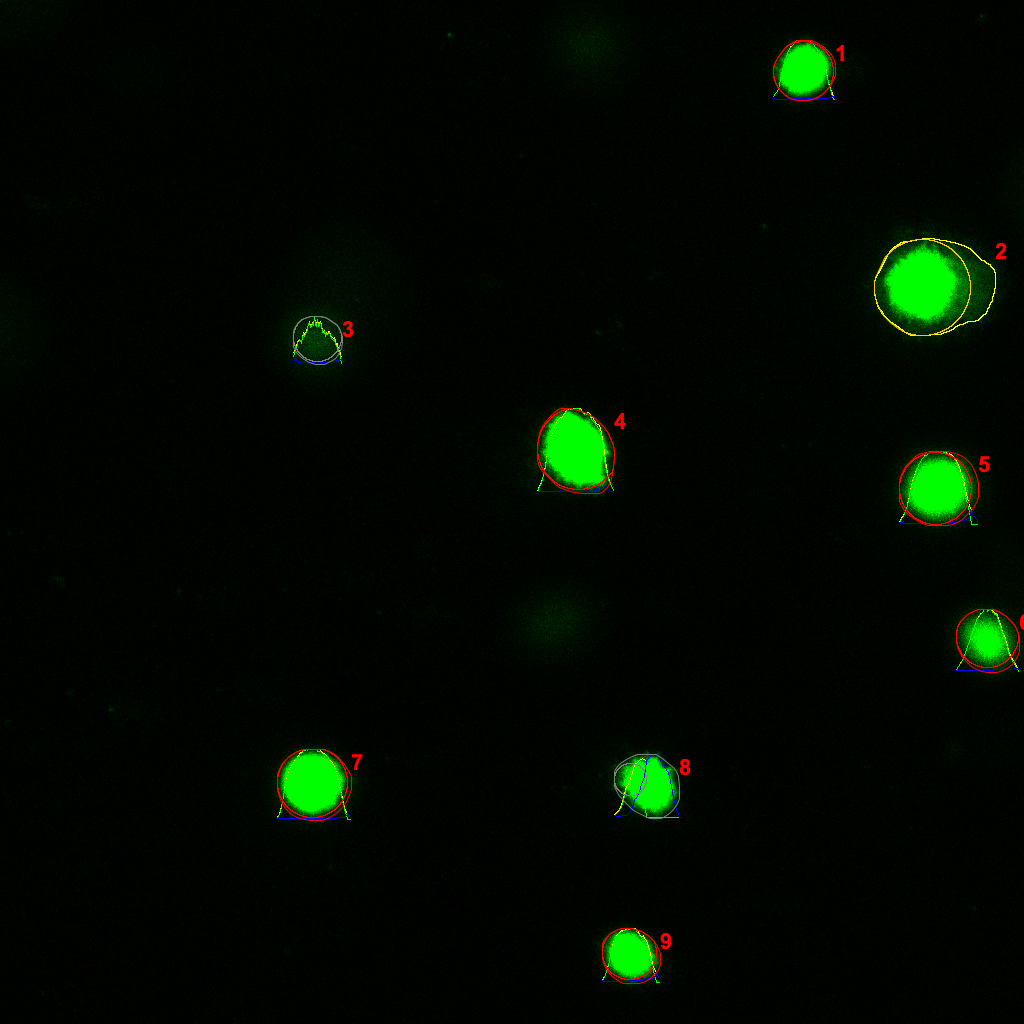

Supplement: Supplementary file 13 — Source data Fig. 6 [file 44321_2026_393_MOESM13_ESM.zip › Figure 6/6B/220915 Comet assay alkaline/output THZ531 6h 1/68_THZ531_6h_10x_Ccenter15_1AUall_rep1_Maximum.ome.tif_out.tif]

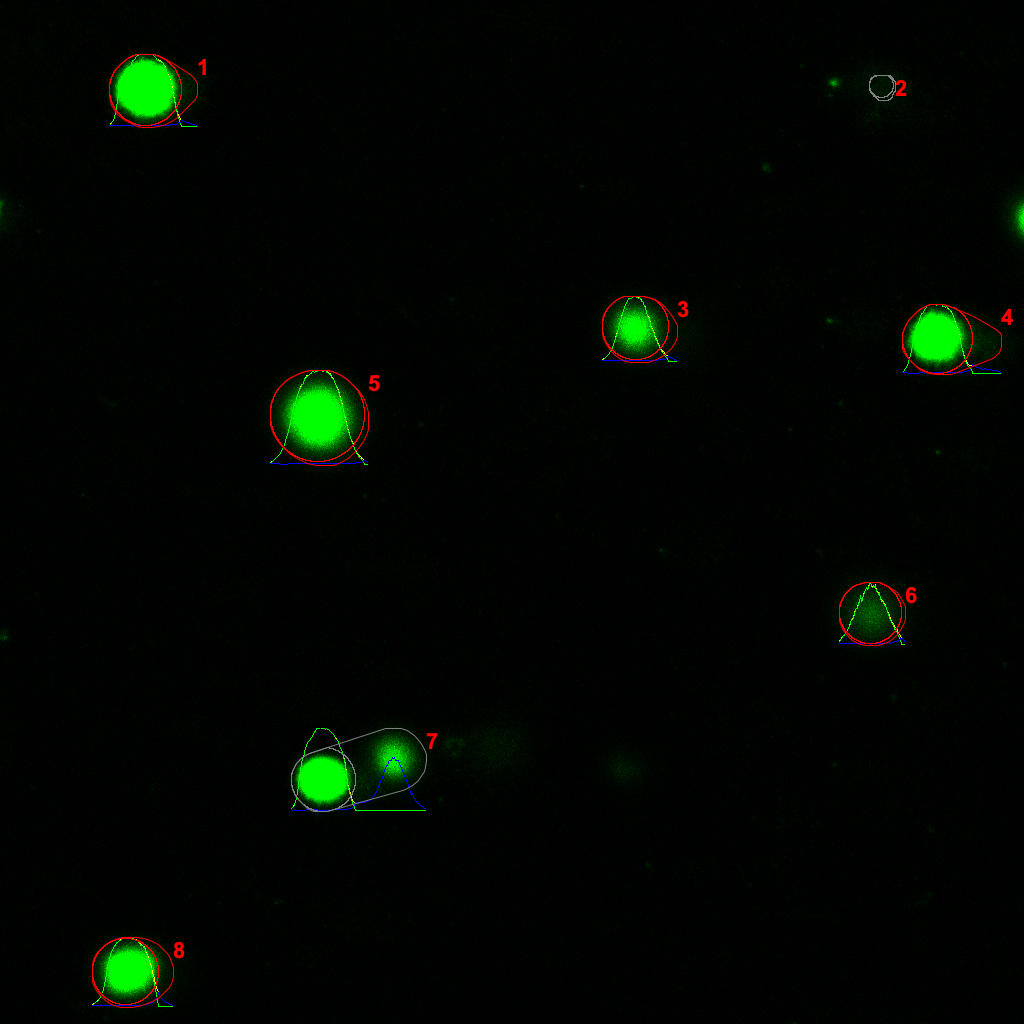

Supplement: Supplementary file 13 — Source data Fig. 6 [file 44321_2026_393_MOESM13_ESM.zip › Figure 6/6B/220915 Comet assay alkaline/output THZ531 6h 1/66_THZ531_6h_10x_Ccenter15_1AUall_rep1_Maximum.ome.tif_out.tif]

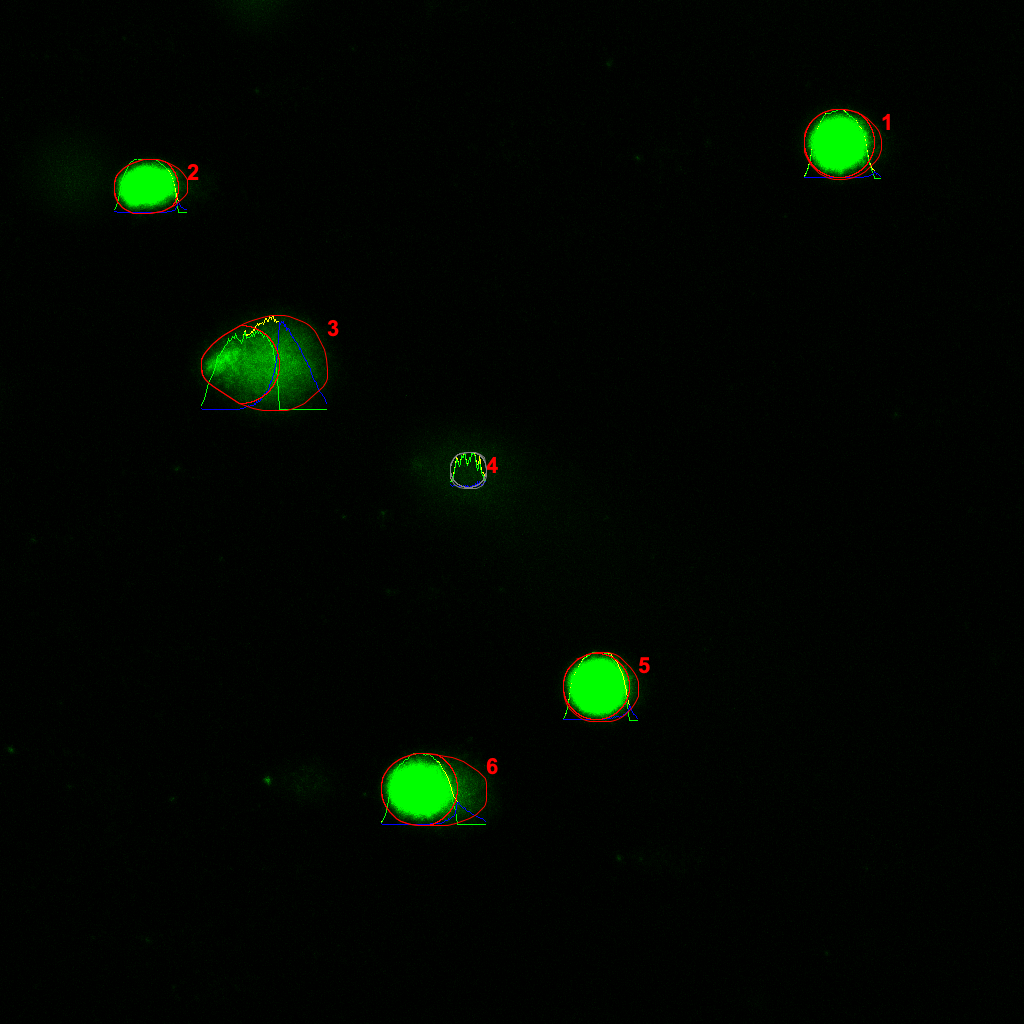

Supplement: Supplementary file 13 — Source data Fig. 6 [file 44321_2026_393_MOESM13_ESM.zip › Figure 6/6B/220915 Comet assay alkaline/output THZ531 6h 1/64_THZ531_6h_10x_Ccenter15_1AUall_rep1_Maximum.ome.tif_out.tif]

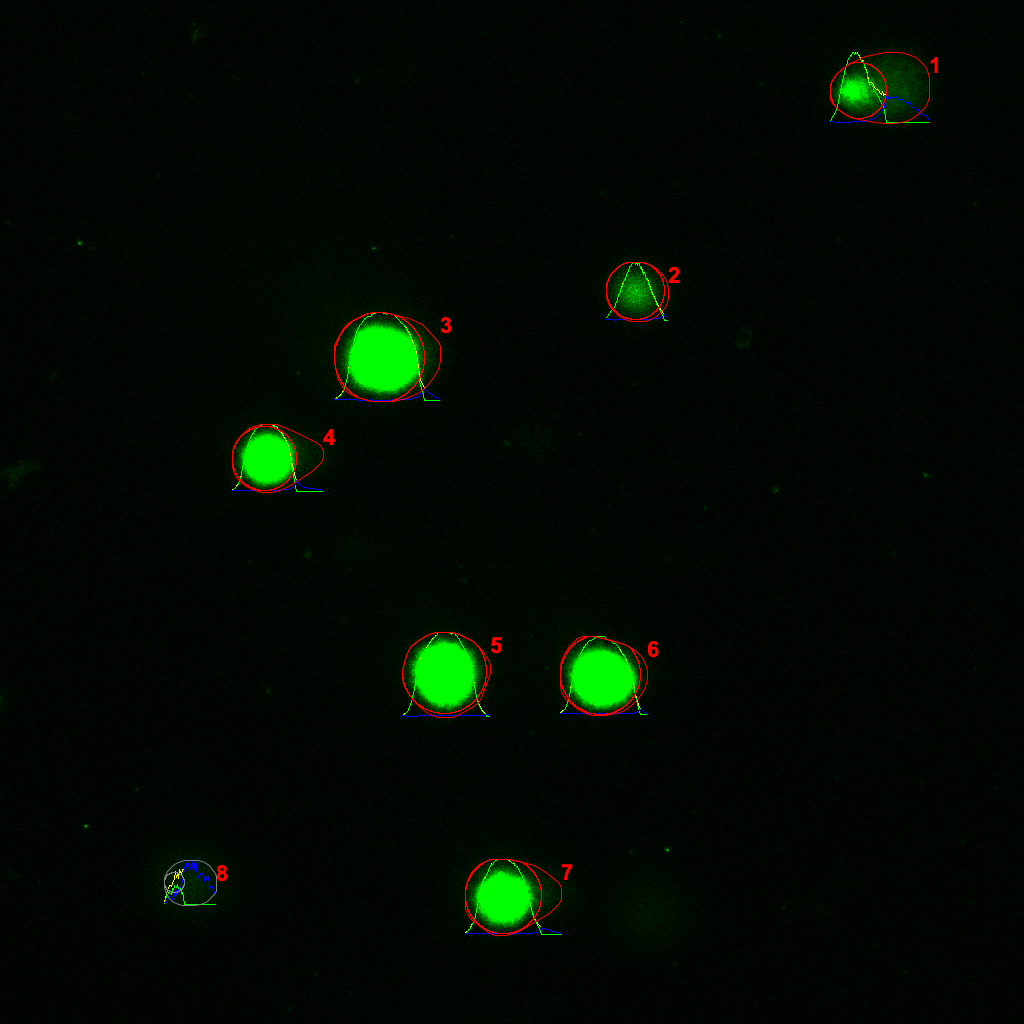

Supplement: Supplementary file 13 — Source data Fig. 6 [file 44321_2026_393_MOESM13_ESM.zip › Figure 6/6B/220915 Comet assay alkaline/output THZ531 6h 1/70_THZ531_6h_10x_Ccenter15_1AUall_rep1_Maximum.ome.tif_out.tif]

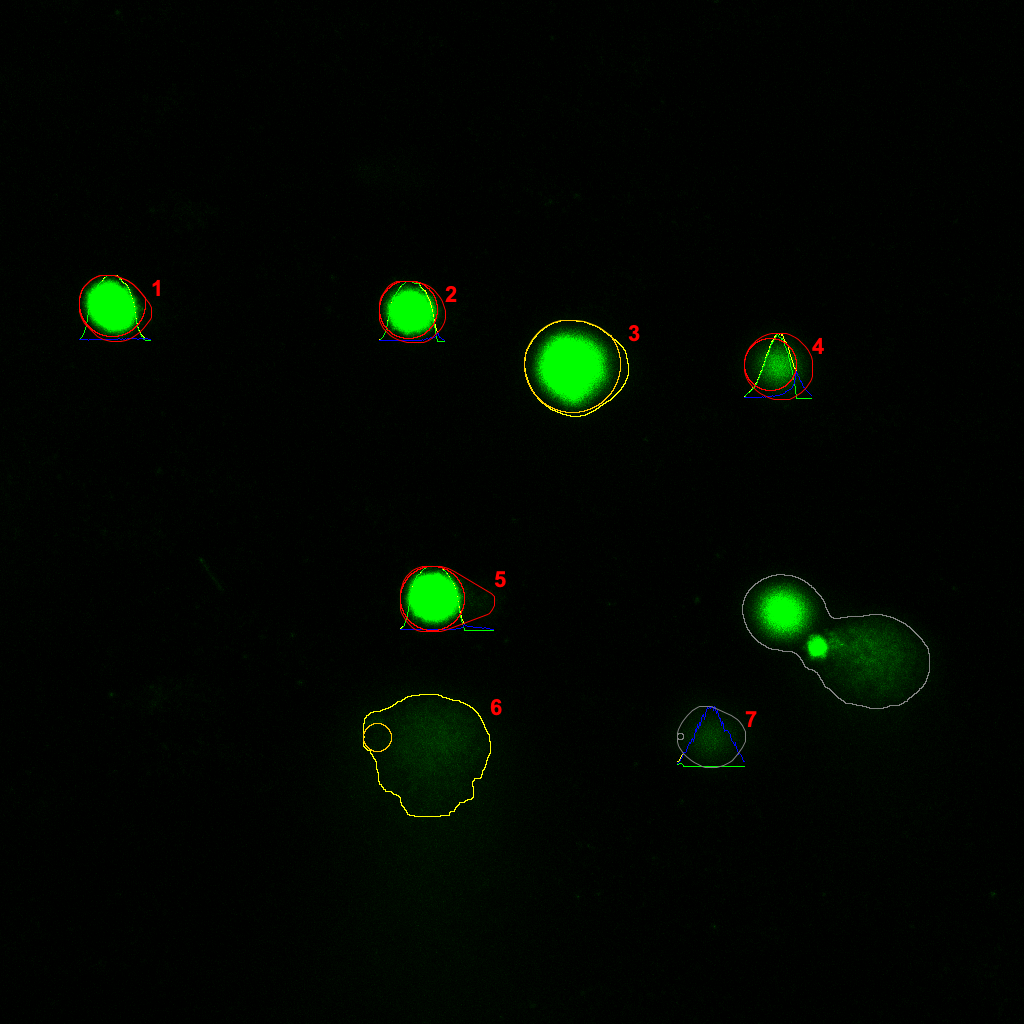

Supplement: Supplementary file 13 — Source data Fig. 6 [file 44321_2026_393_MOESM13_ESM.zip › Figure 6/6B/220915 Comet assay alkaline/output THZ531 6h 1/67_THZ531_6h_10x_Ccenter15_1AUall_rep1_Maximum.ome.tif_out.tif]

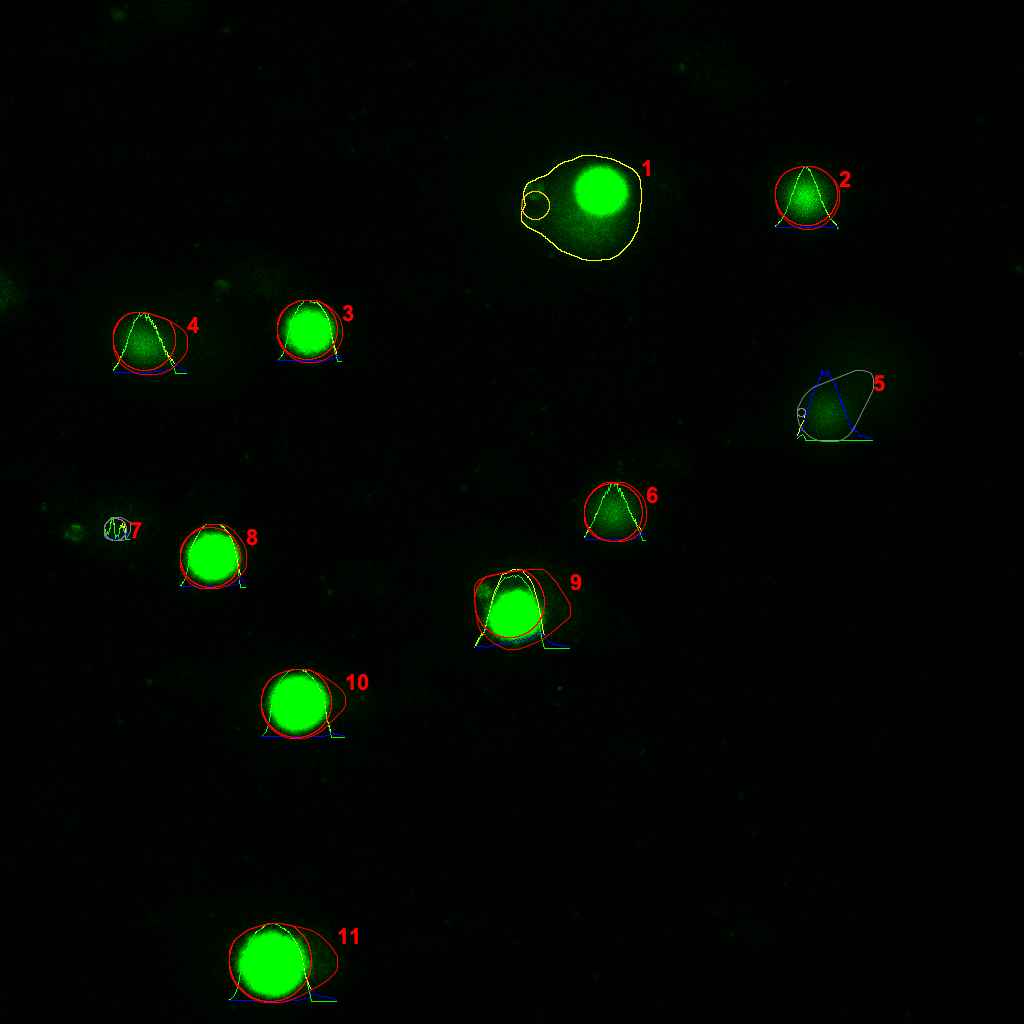

Supplement: Supplementary file 13 — Source data Fig. 6 [file 44321_2026_393_MOESM13_ESM.zip › Figure 6/6B/220915 Comet assay alkaline/output THZ531 6h 1/69_THZ531_6h_10x_Ccenter15_1AUall_rep1_Maximum.ome.tif_out.tif]

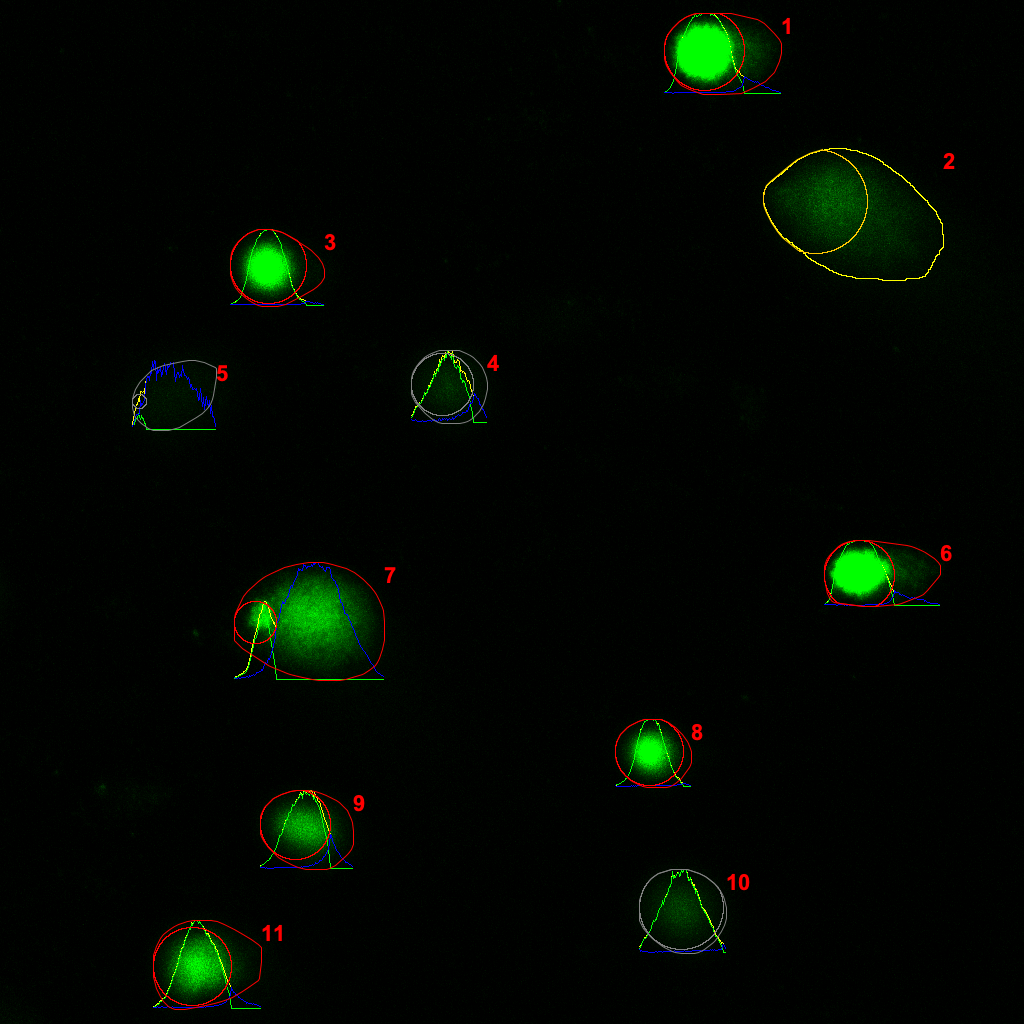

Supplement: Supplementary file 13 — Source data Fig. 6 [file 44321_2026_393_MOESM13_ESM.zip › Figure 6/6B/220915 Comet assay alkaline/output THZ531 6h 1/65_THZ531_6h_10x_Ccenter15_1AUall_rep1_Maximum.ome.tif_out.tif]

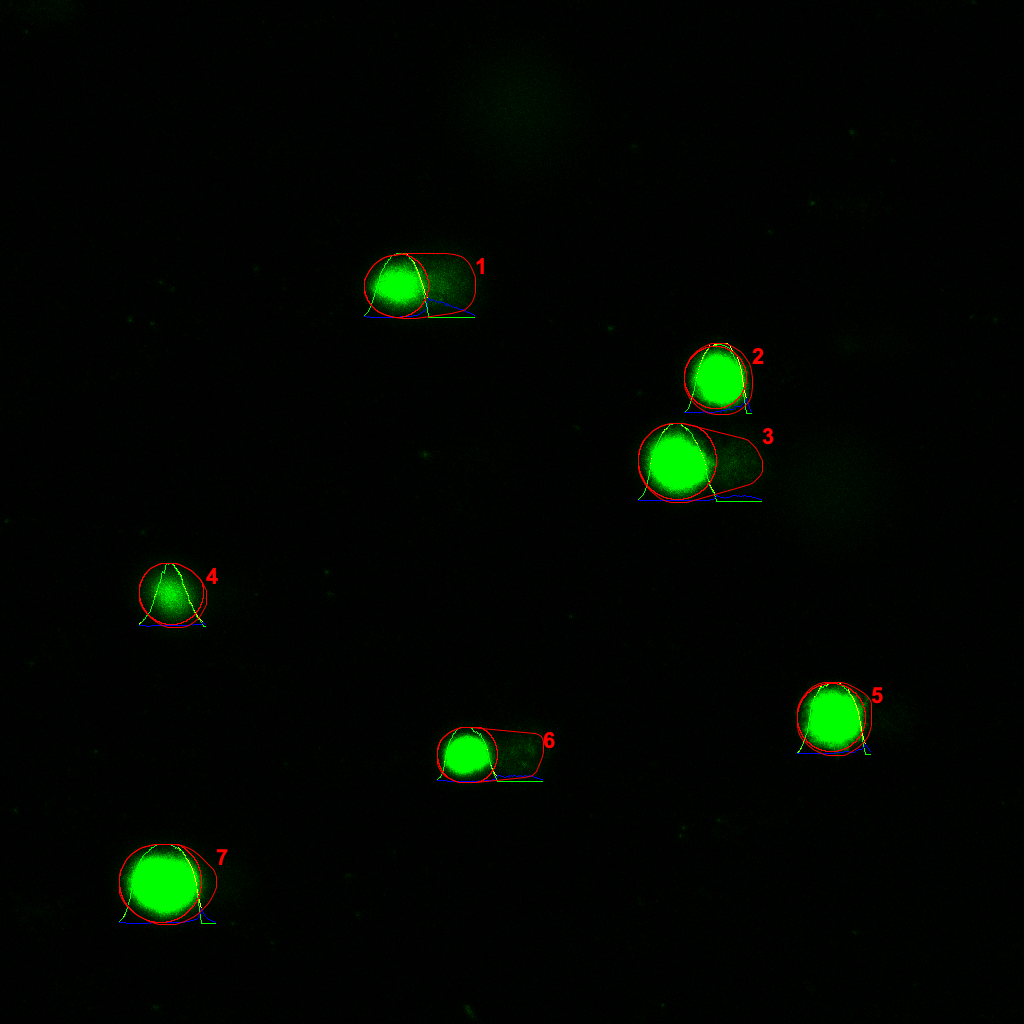

Supplement: Supplementary file 13 — Source data Fig. 6 [file 44321_2026_393_MOESM13_ESM.zip › Figure 6/6B/220915 Comet assay alkaline/output DMSO 1/1_DMSO_6h_10x_Ccenter15_1AUall_rep1_Maximum.ome.tif_out.tif]

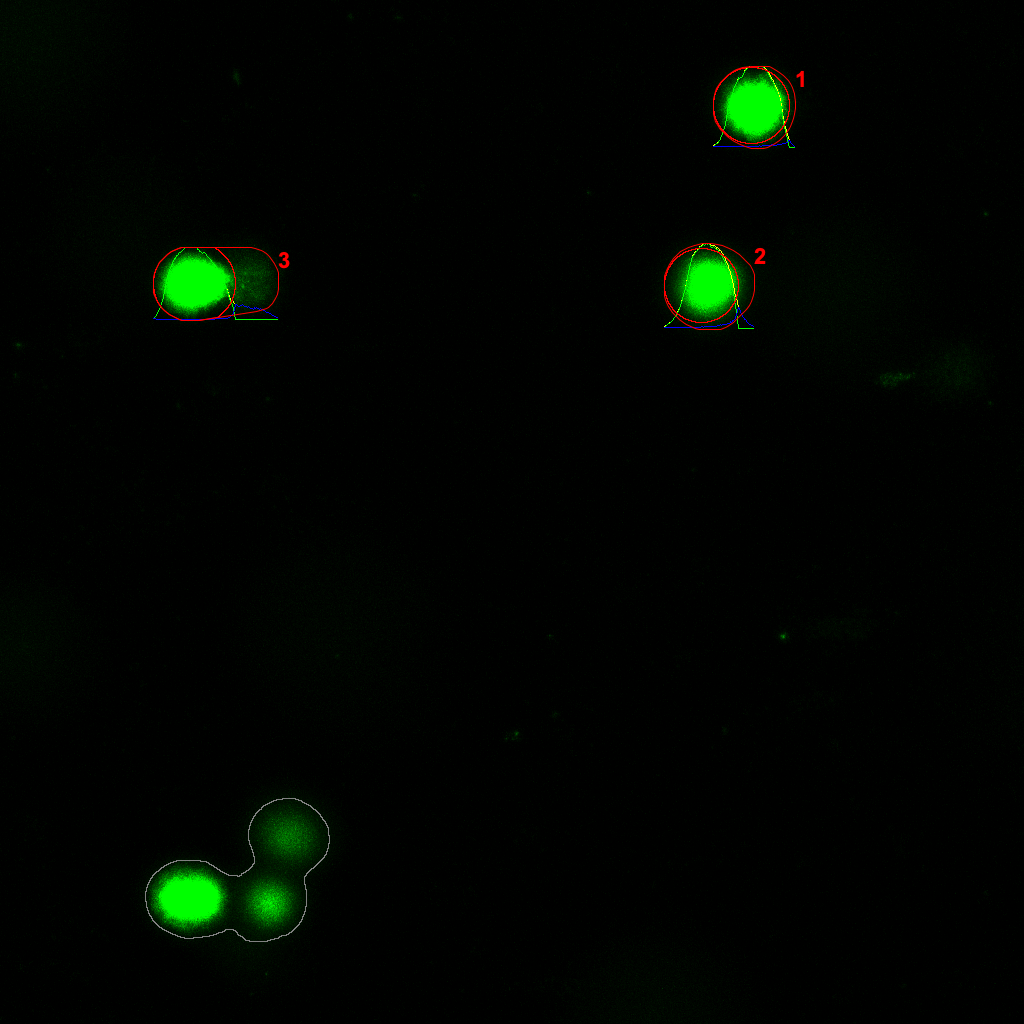

Supplement: Supplementary file 13 — Source data Fig. 6 [file 44321_2026_393_MOESM13_ESM.zip › Figure 6/6B/220915 Comet assay alkaline/output DMSO 1/3_DMSO_6h_10x_Ccenter15_1AUall_rep1_Maximum.ome.tif_out.tif]

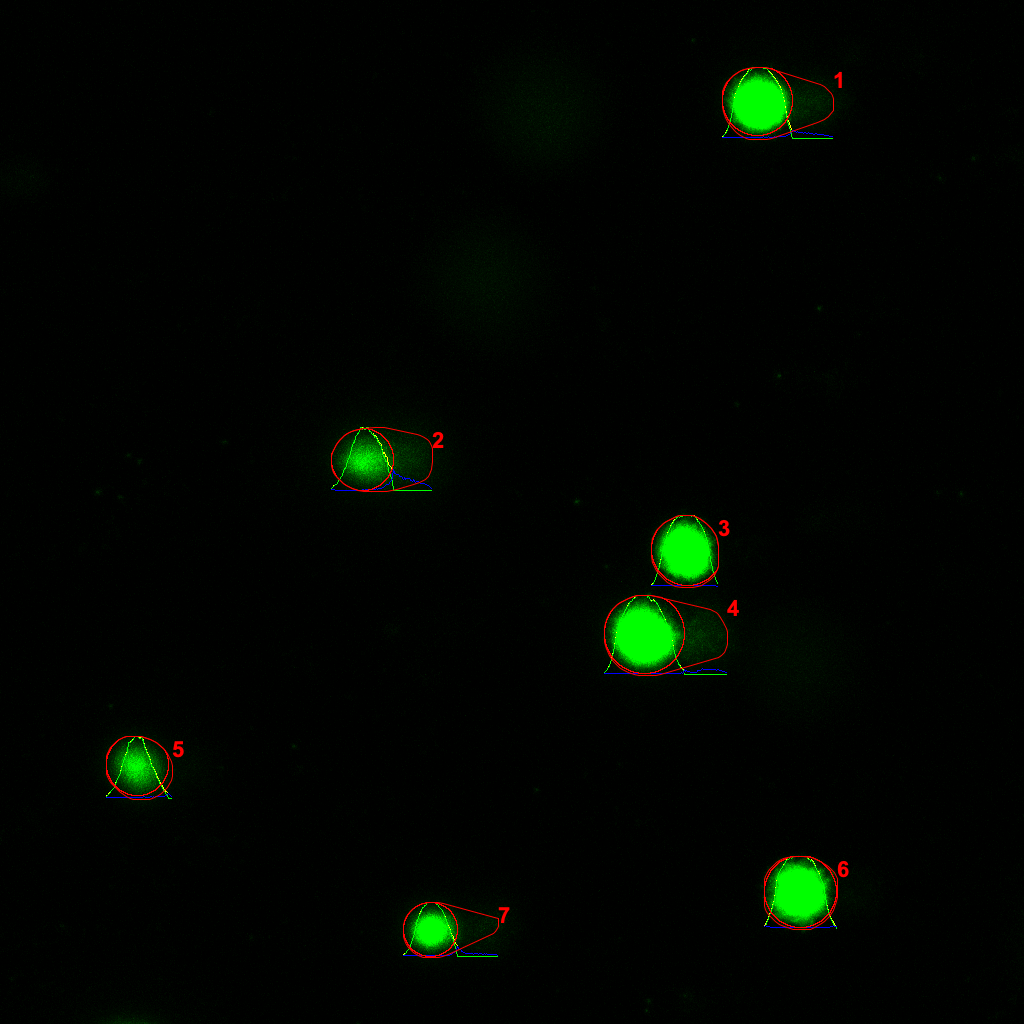

Supplement: Supplementary file 13 — Source data Fig. 6 [file 44321_2026_393_MOESM13_ESM.zip › Figure 6/6B/220915 Comet assay alkaline/output DMSO 1/2_DMSO_6h_10x_Ccenter15_1AUall_rep1_Maximum.ome.tif_out.tif]

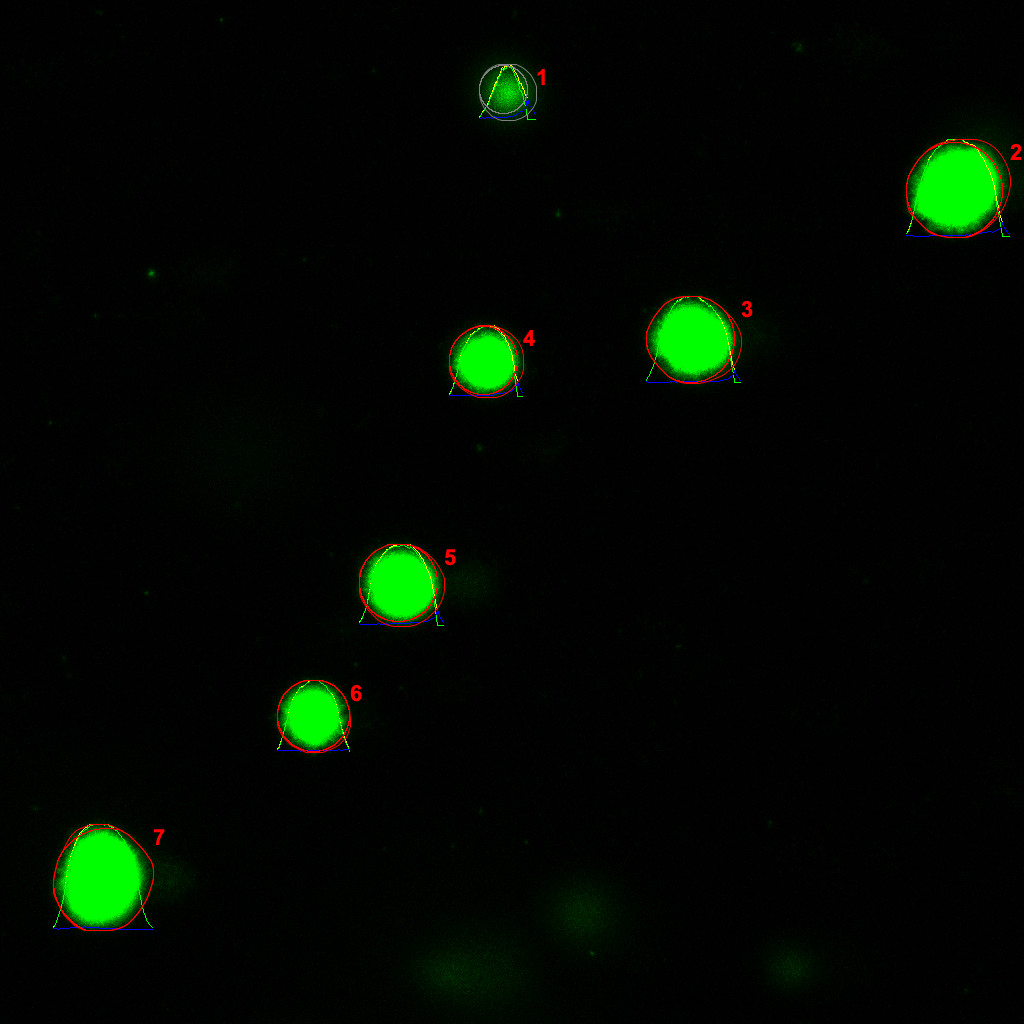

Supplement: Supplementary file 13 — Source data Fig. 6 [file 44321_2026_393_MOESM13_ESM.zip › Figure 6/6B/220915 Comet assay alkaline/output DMSO 1/9_DMSO_6h_10x_Ccenter15_1AUall_rep1_Maximum.ome.tif_out.tif]

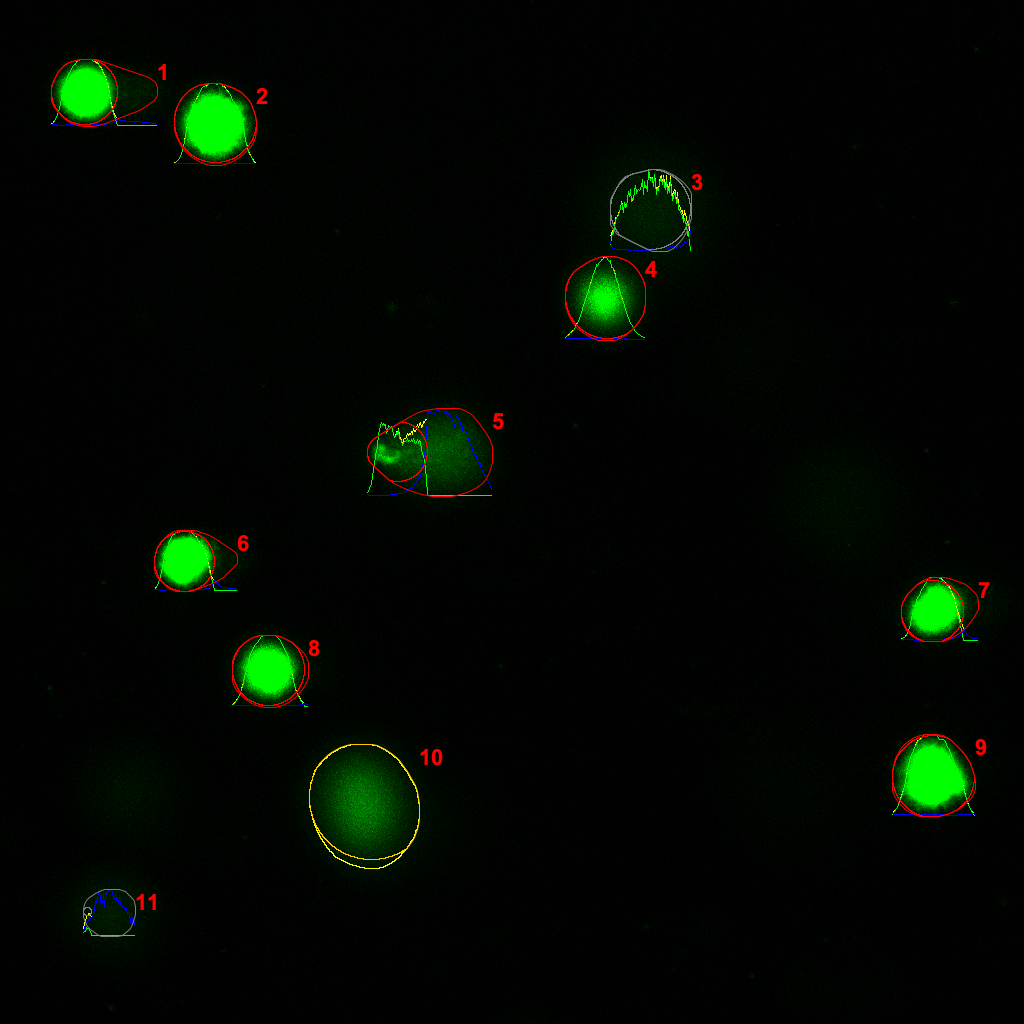

Supplement: Supplementary file 13 — Source data Fig. 6 [file 44321_2026_393_MOESM13_ESM.zip › Figure 6/6B/220915 Comet assay alkaline/output DMSO 1/7_DMSO_6h_10x_Ccenter15_1AUall_rep1_Maximum.ome.tif_out.tif]

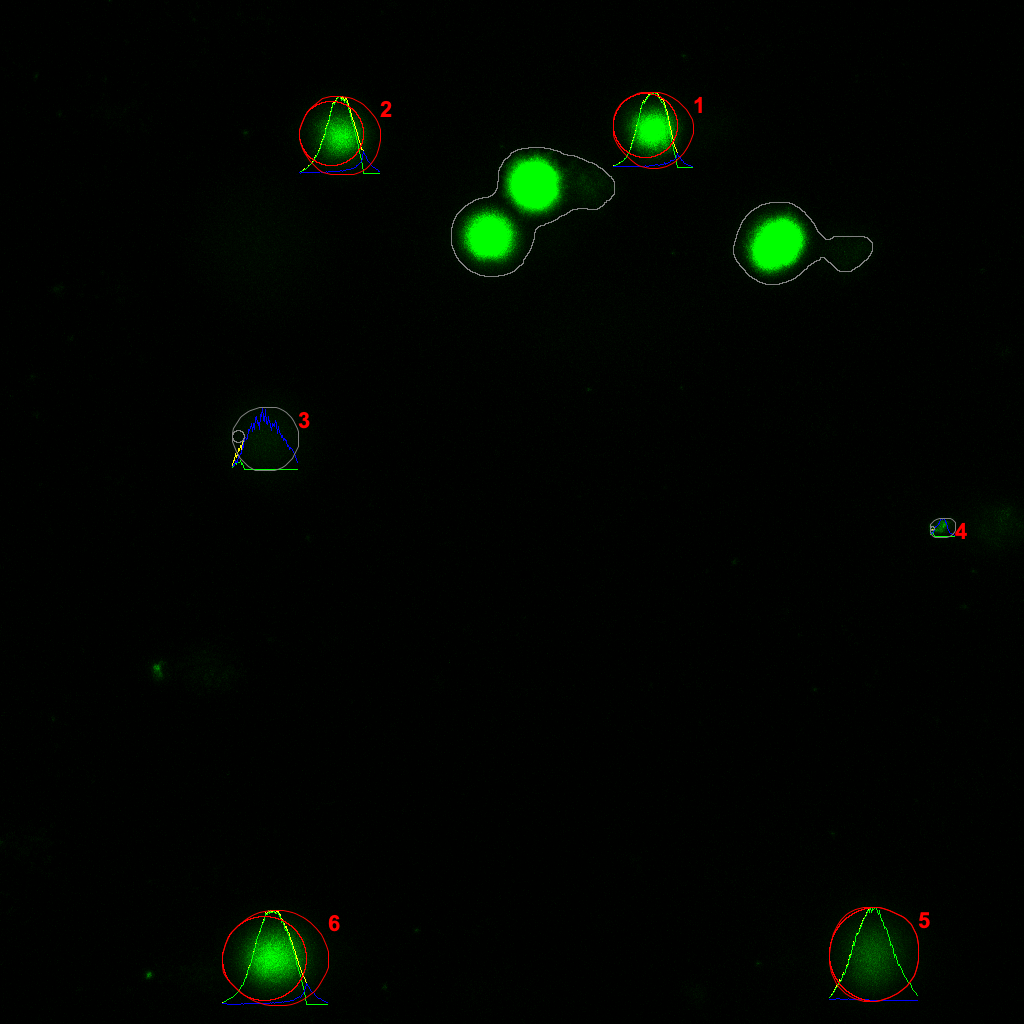

Supplement: Supplementary file 13 — Source data Fig. 6 [file 44321_2026_393_MOESM13_ESM.zip › Figure 6/6B/220915 Comet assay alkaline/output DMSO 1/8_DMSO_6h_10x_Ccenter15_1AUall_rep1_Maximum.ome.tif_out.tif]

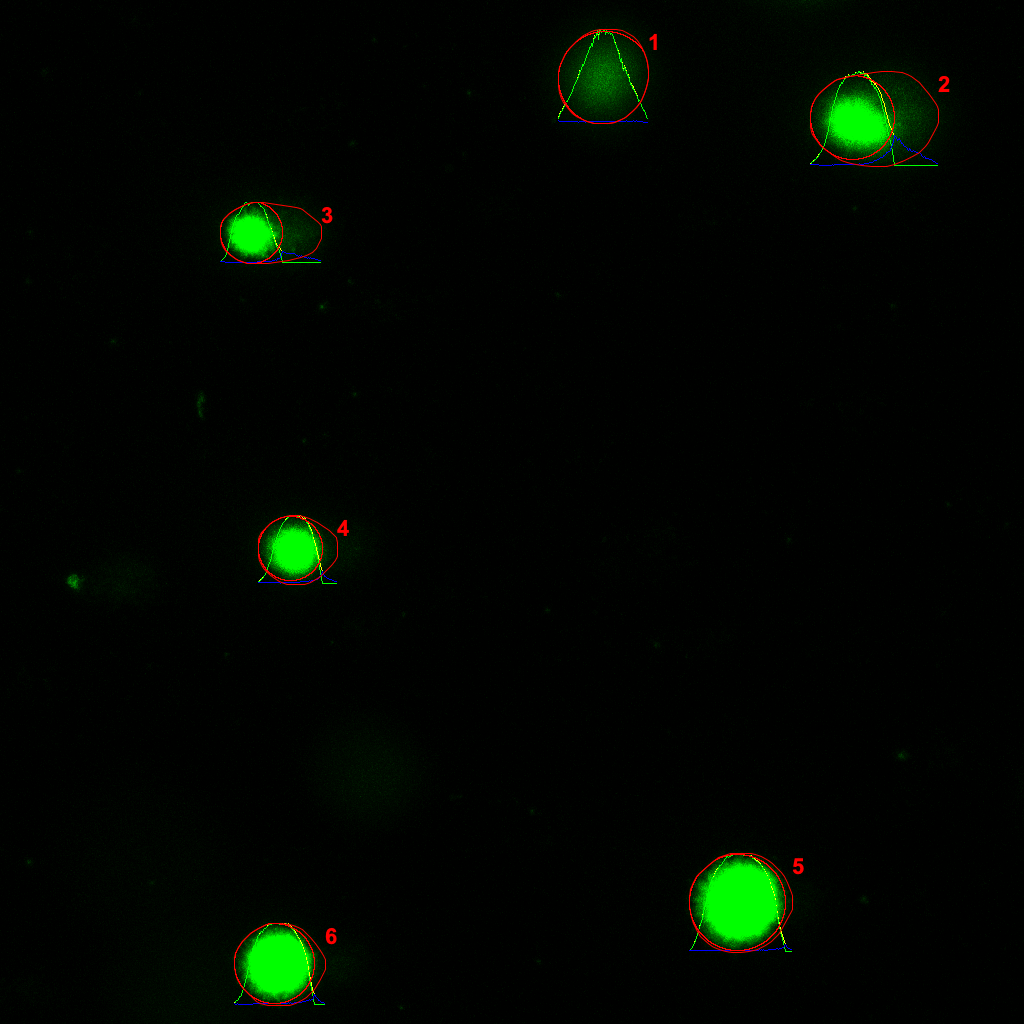

Supplement: Supplementary file 13 — Source data Fig. 6 [file 44321_2026_393_MOESM13_ESM.zip › Figure 6/6B/220915 Comet assay alkaline/output DMSO 1/6_DMSO_6h_10x_Ccenter15_1AUall_rep1_Maximum.ome.tif_out.tif]

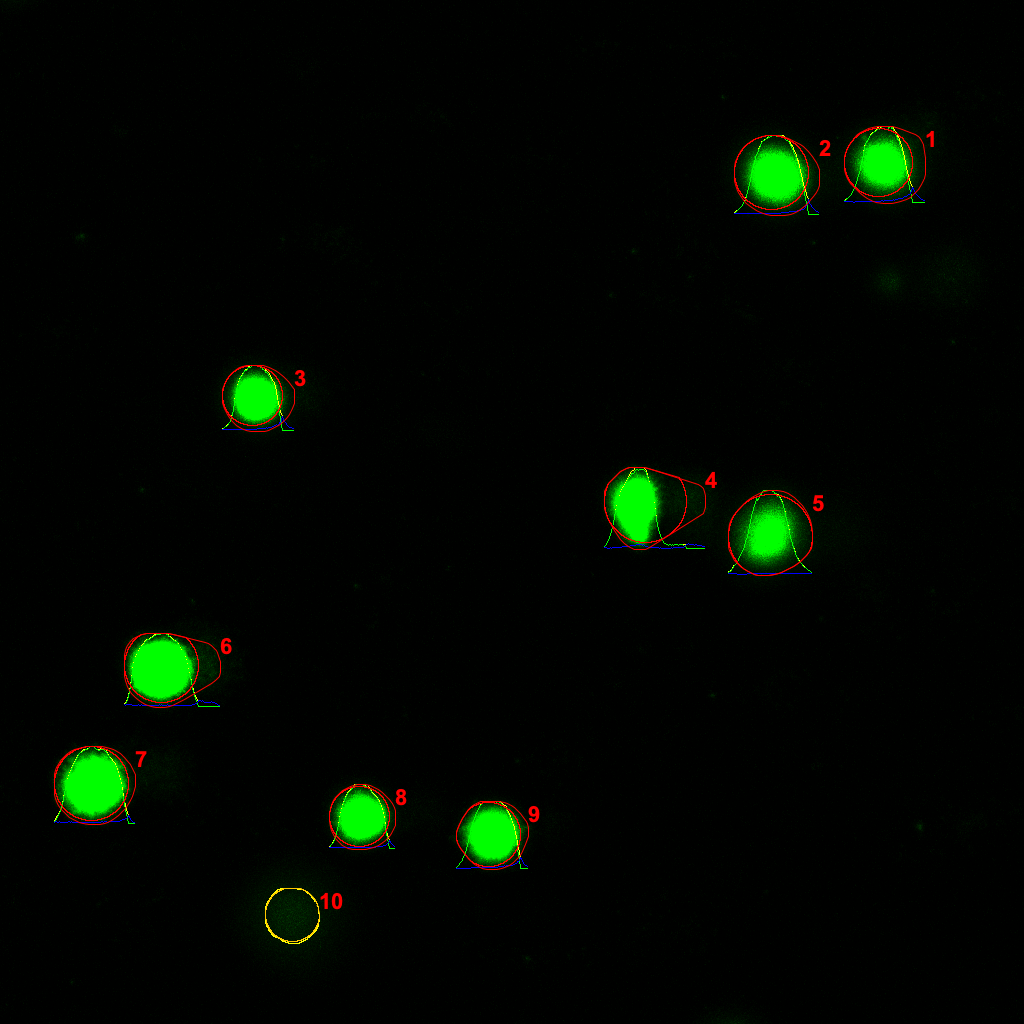

Supplement: Supplementary file 13 — Source data Fig. 6 [file 44321_2026_393_MOESM13_ESM.zip › Figure 6/6B/220915 Comet assay alkaline/output DMSO 1/4_DMSO_6h_10x_Ccenter15_1AUall_rep1_Maximum.ome.tif_out.tif]

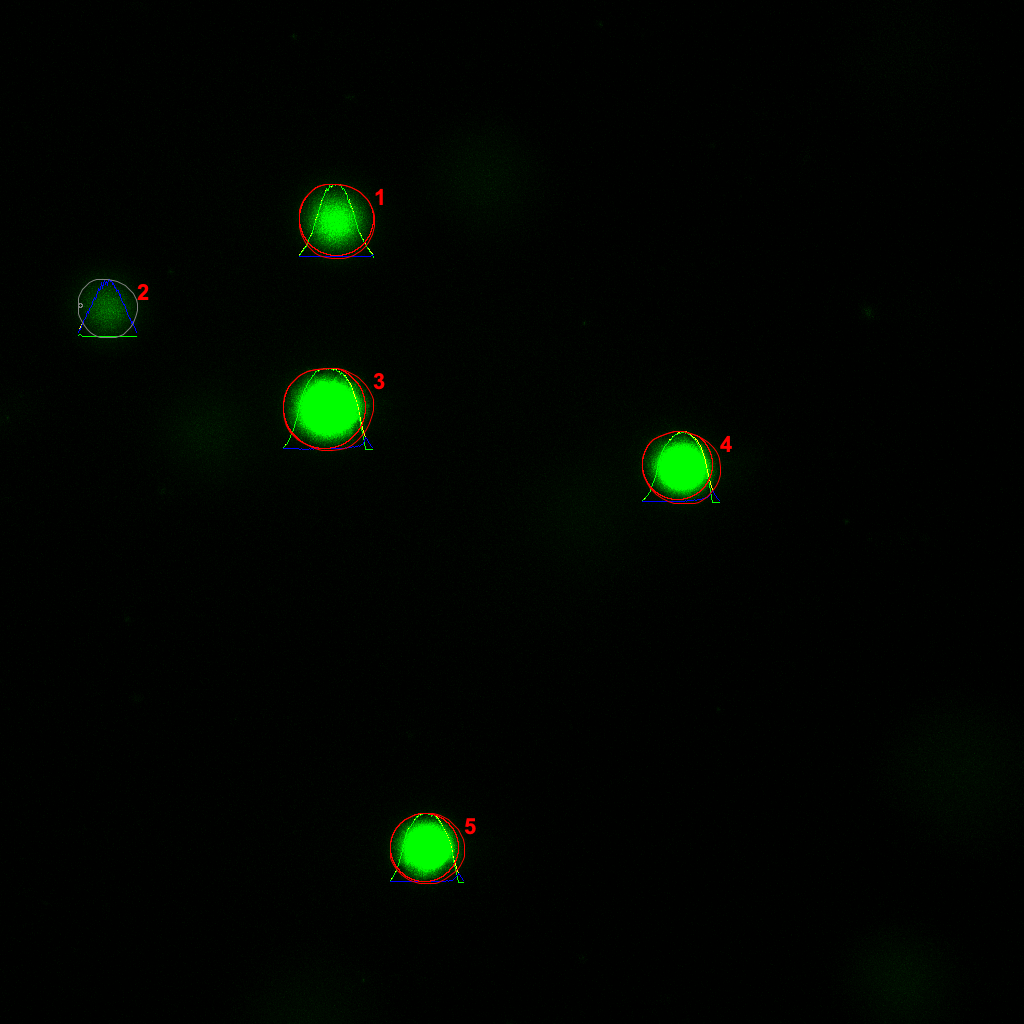

Supplement: Supplementary file 13 — Source data Fig. 6 [file 44321_2026_393_MOESM13_ESM.zip › Figure 6/6B/220915 Comet assay alkaline/output DMSO 1/10_DMSO_6h_10x_Ccenter15_1AUall_rep1_Maximum.ome.tif_out.tif]

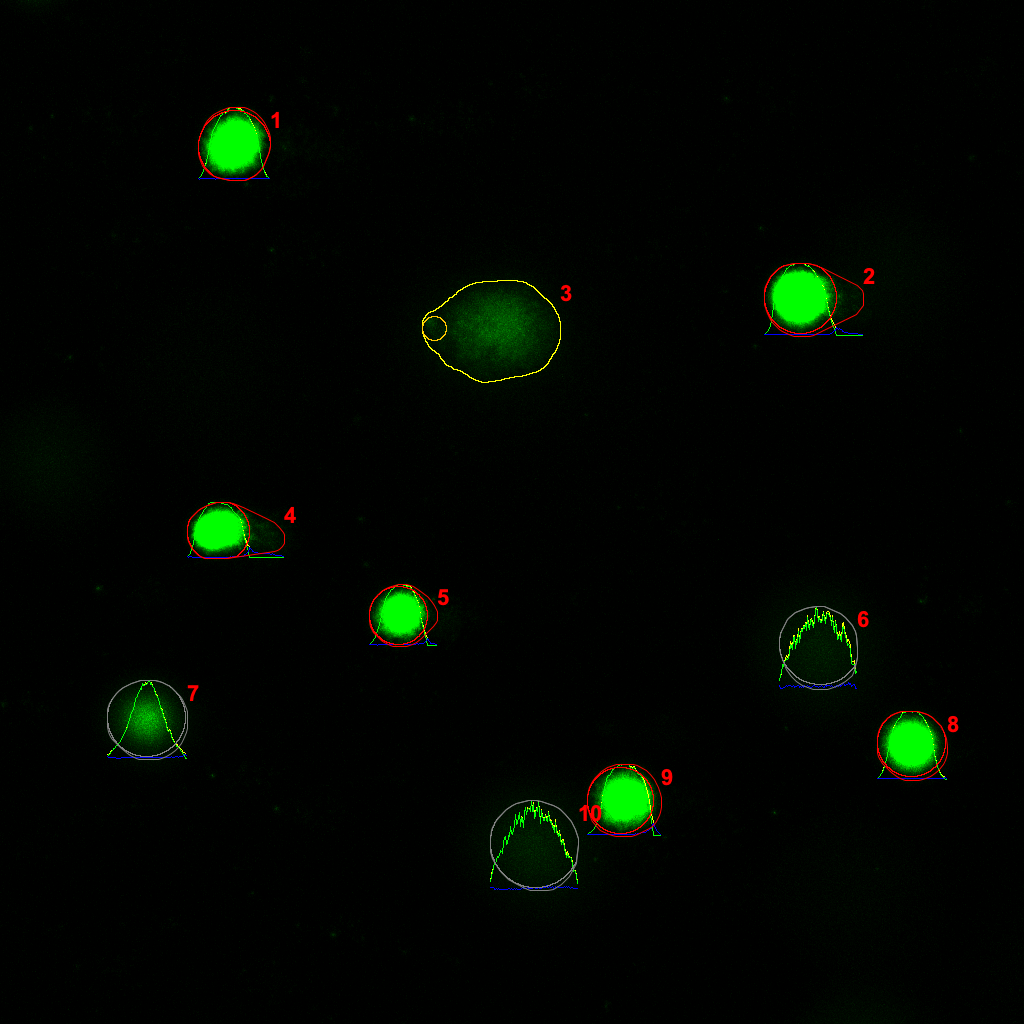

Supplement: Supplementary file 13 — Source data Fig. 6 [file 44321_2026_393_MOESM13_ESM.zip › Figure 6/6B/220915 Comet assay alkaline/output DMSO 1/5_DMSO_6h_10x_Ccenter15_1AUall_rep1_Maximum.ome.tif_out.tif]

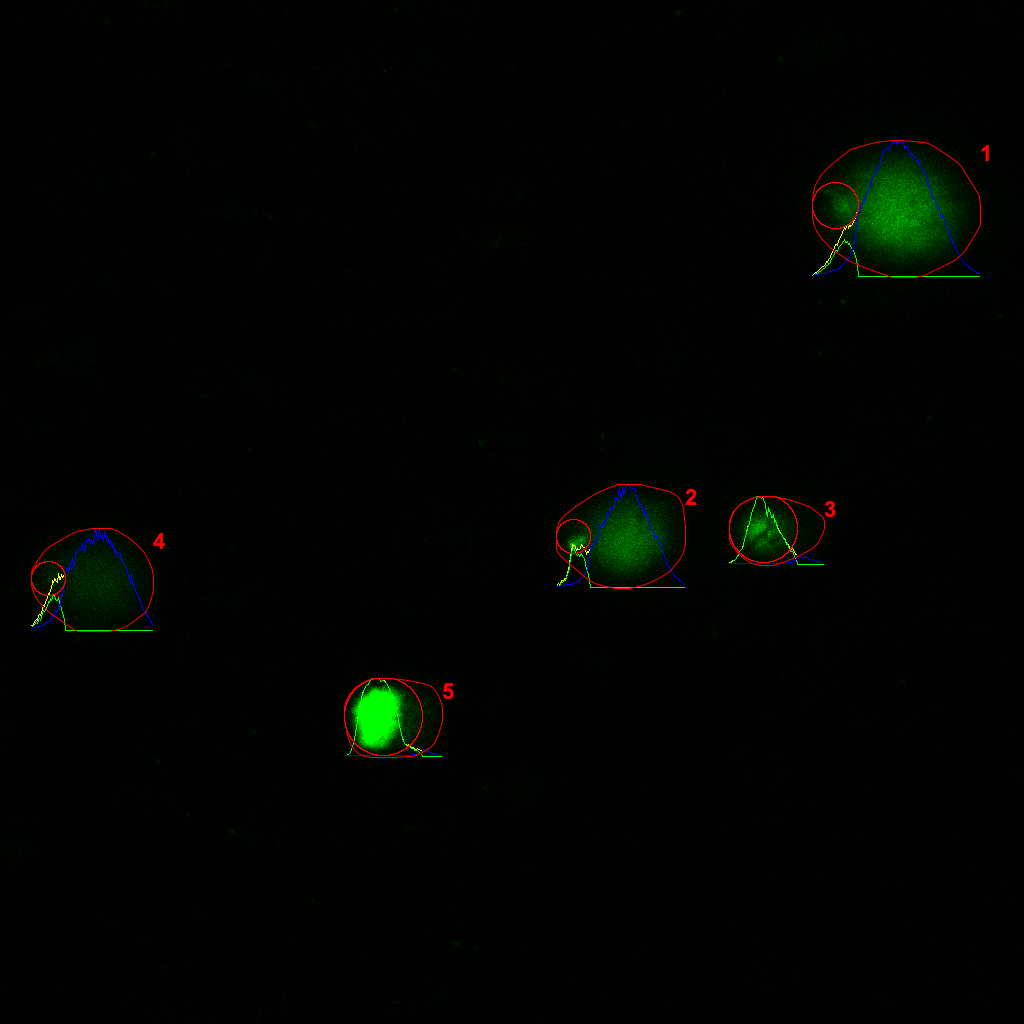

Supplement: Supplementary file 13 — Source data Fig. 6 [file 44321_2026_393_MOESM13_ESM.zip › Figure 6/6B/220915 Comet assay alkaline/output etoposide 1/30_Etoposide_4h_10x_Ccenter15_1AUall_rep1_Maximum.ome.tif_out.tif]

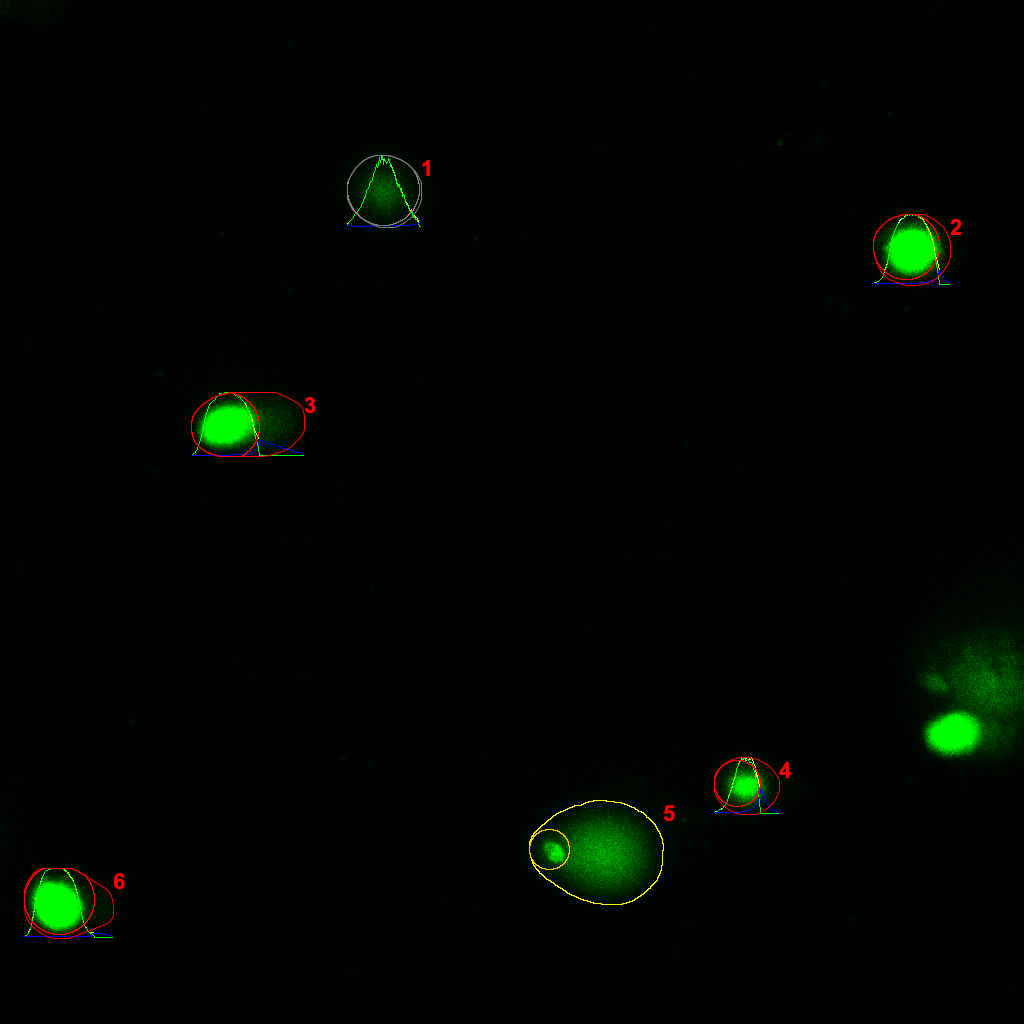

Supplement: Supplementary file 13 — Source data Fig. 6 [file 44321_2026_393_MOESM13_ESM.zip › Figure 6/6B/220915 Comet assay alkaline/output etoposide 1/28_Etoposide_4h_10x_Ccenter15_1AUall_rep1_Maximum.ome.tif_out.tif]

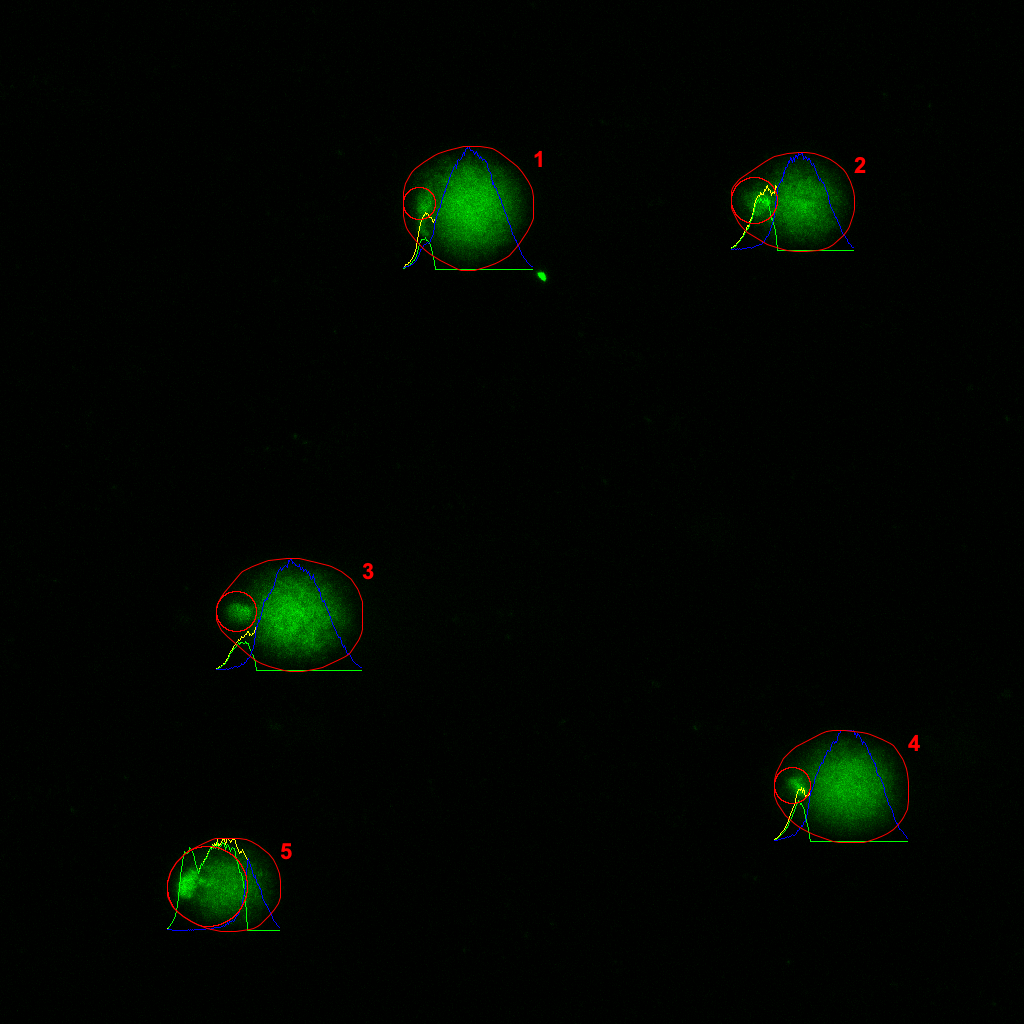

Supplement: Supplementary file 13 — Source data Fig. 6 [file 44321_2026_393_MOESM13_ESM.zip › Figure 6/6B/220915 Comet assay alkaline/output etoposide 1/23_Etoposide_4h_10x_Ccenter15_1AUall_rep1_Maximum.ome.tif_out.tif]

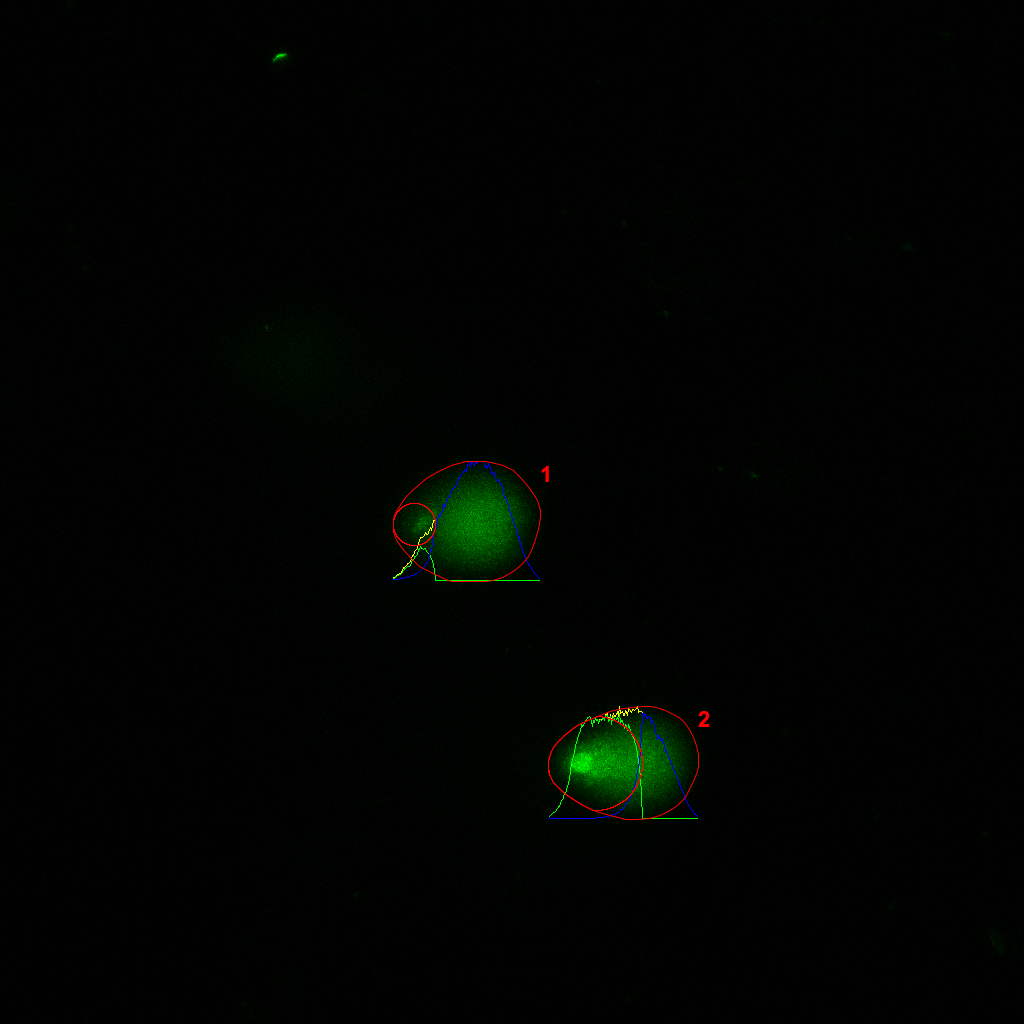

Supplement: Supplementary file 13 — Source data Fig. 6 [file 44321_2026_393_MOESM13_ESM.zip › Figure 6/6B/220915 Comet assay alkaline/output etoposide 1/26_Etoposide_4h_10x_Ccenter15_1AUall_rep1_Maximum.ome.tif_out.tif]

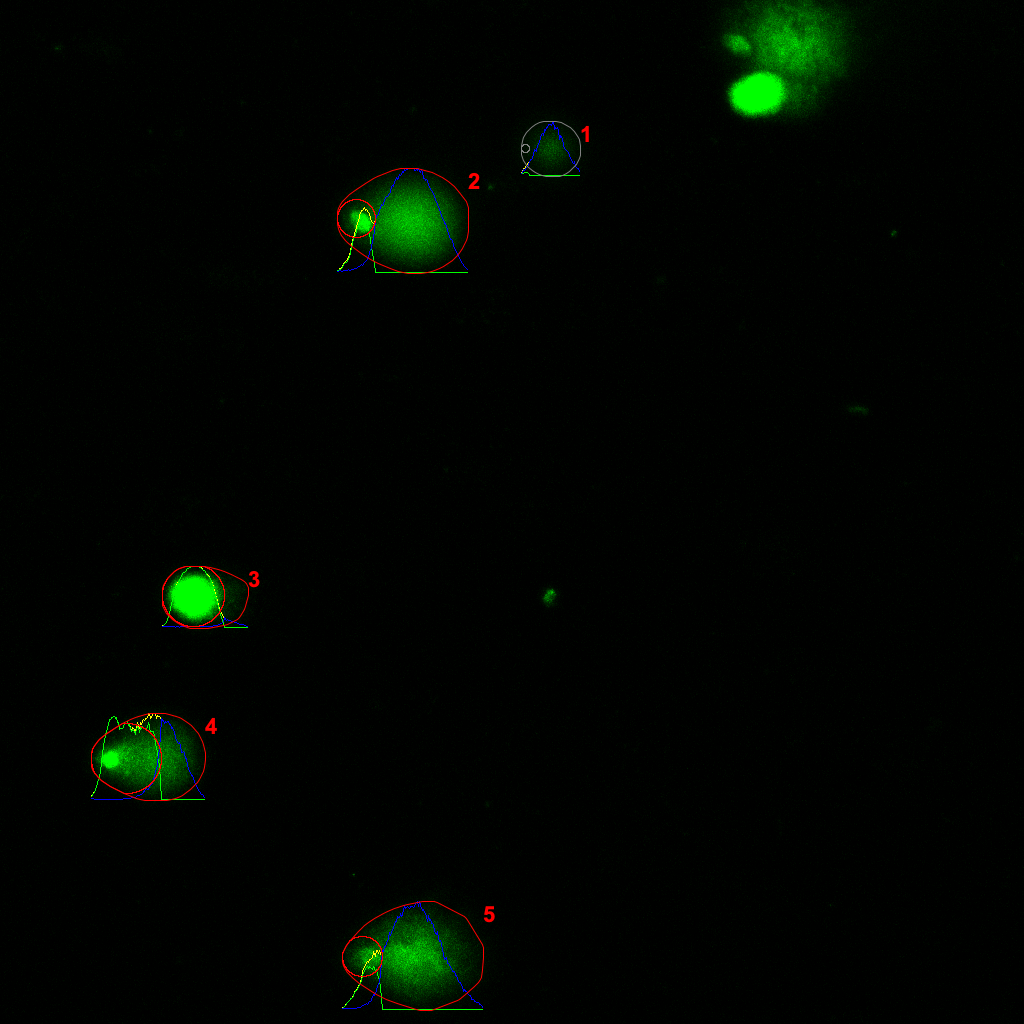

Supplement: Supplementary file 13 — Source data Fig. 6 [file 44321_2026_393_MOESM13_ESM.zip › Figure 6/6B/220915 Comet assay alkaline/output etoposide 1/21_Etoposide_4h_10x_Ccenter15_1AUall_rep1_Maximum.ome.tif_out.tif]

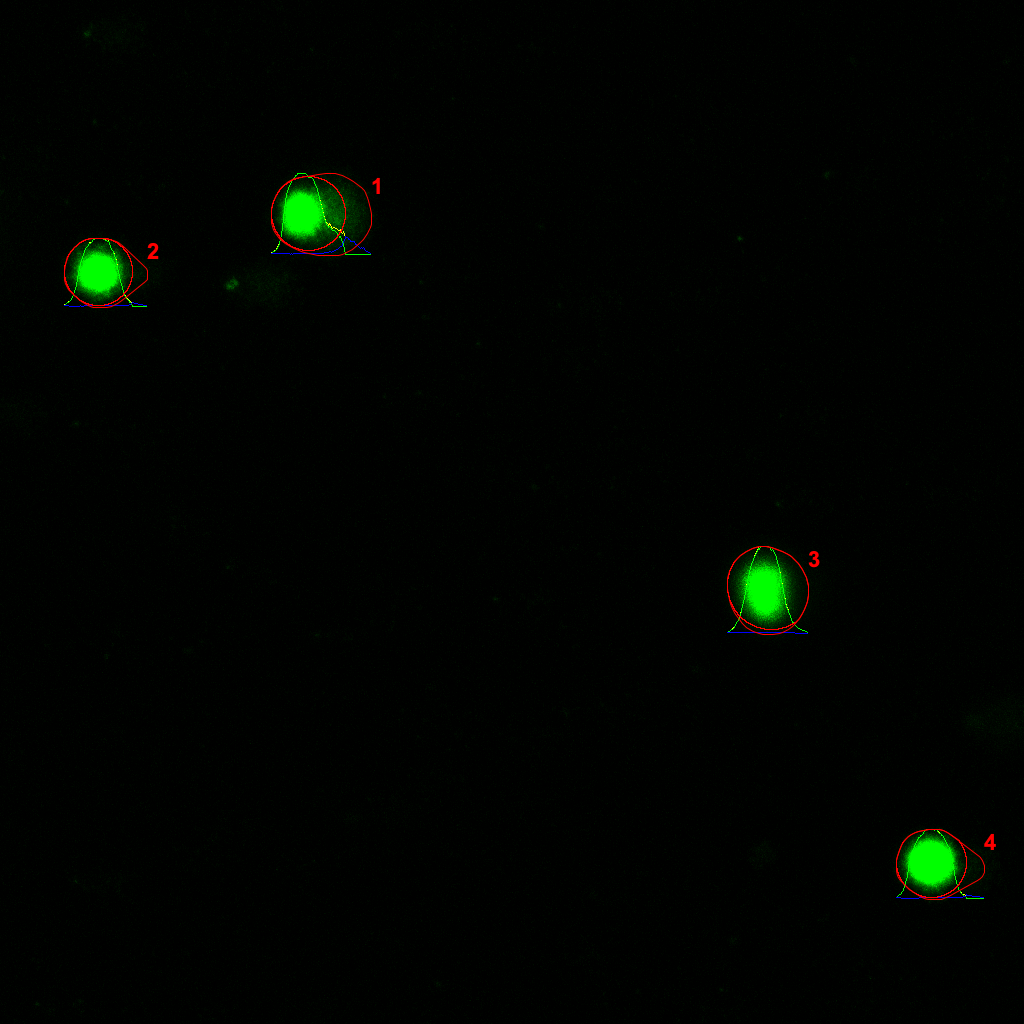

Supplement: Supplementary file 13 — Source data Fig. 6 [file 44321_2026_393_MOESM13_ESM.zip › Figure 6/6B/220915 Comet assay alkaline/output etoposide 1/24_Etoposide_4h_10x_Ccenter15_1AUall_rep1_Maximum.ome.tif_out.tif]

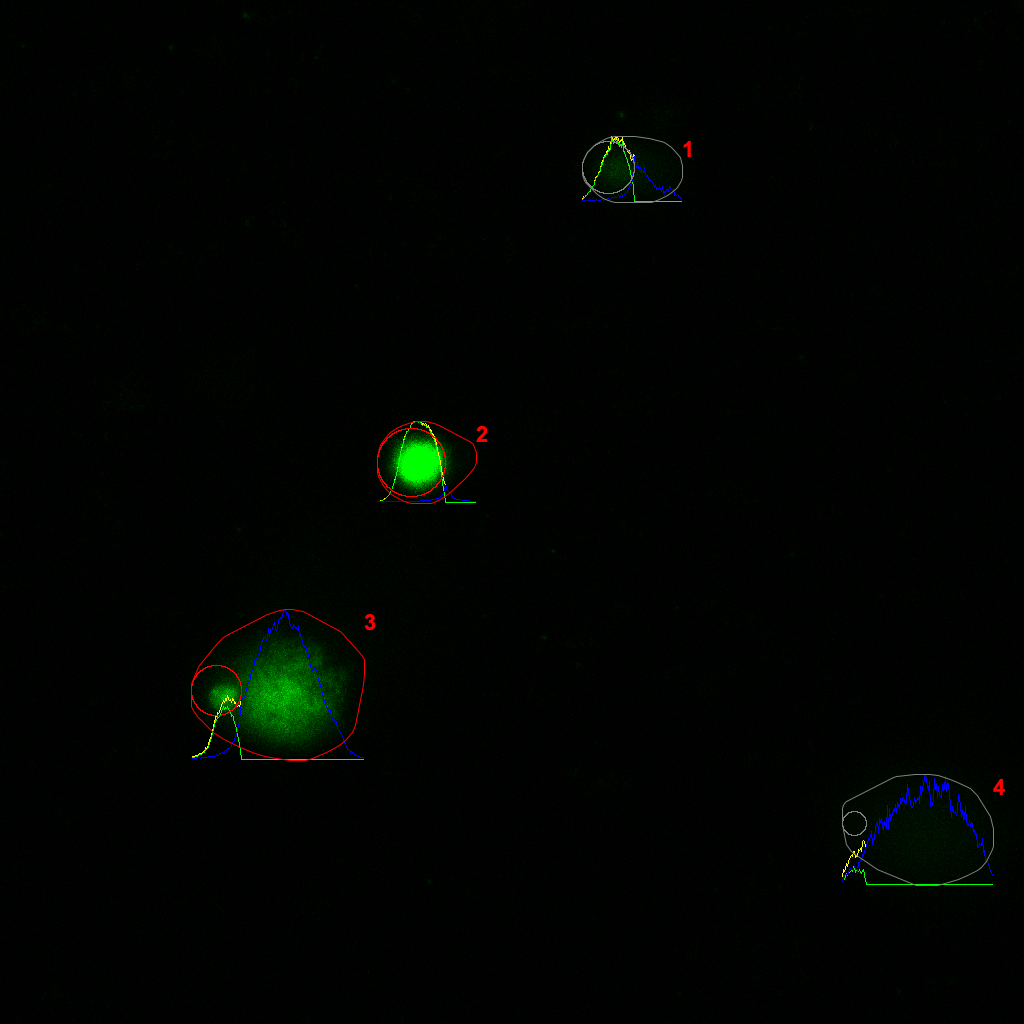

Supplement: Supplementary file 13 — Source data Fig. 6 [file 44321_2026_393_MOESM13_ESM.zip › Figure 6/6B/220915 Comet assay alkaline/output etoposide 1/27_Etoposide_4h_10x_Ccenter15_1AUall_rep1_Maximum.ome.tif_out.tif]

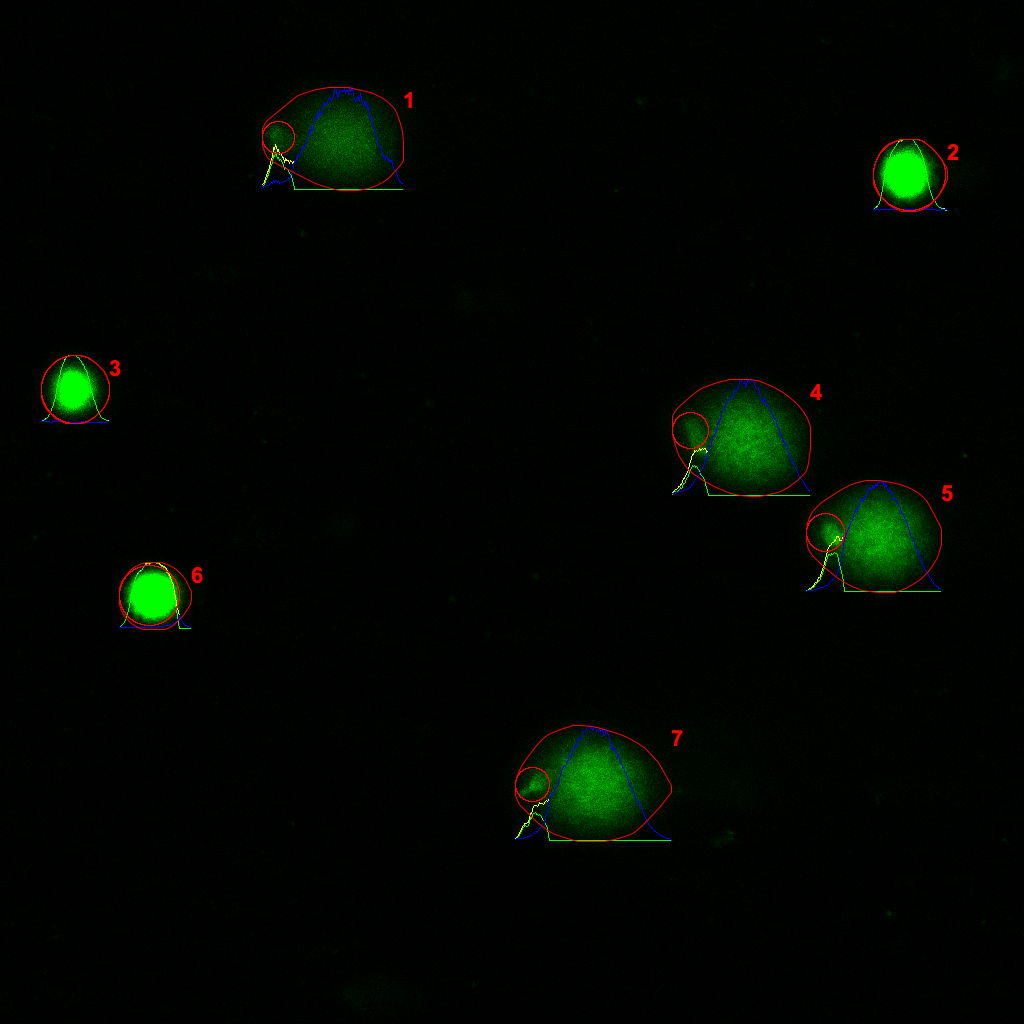

Supplement: Supplementary file 13 — Source data Fig. 6 [file 44321_2026_393_MOESM13_ESM.zip › Figure 6/6B/220915 Comet assay alkaline/output etoposide 1/22_Etoposide_4h_10x_Ccenter15_1AUall_rep1_Maximum.ome.tif_out.tif]

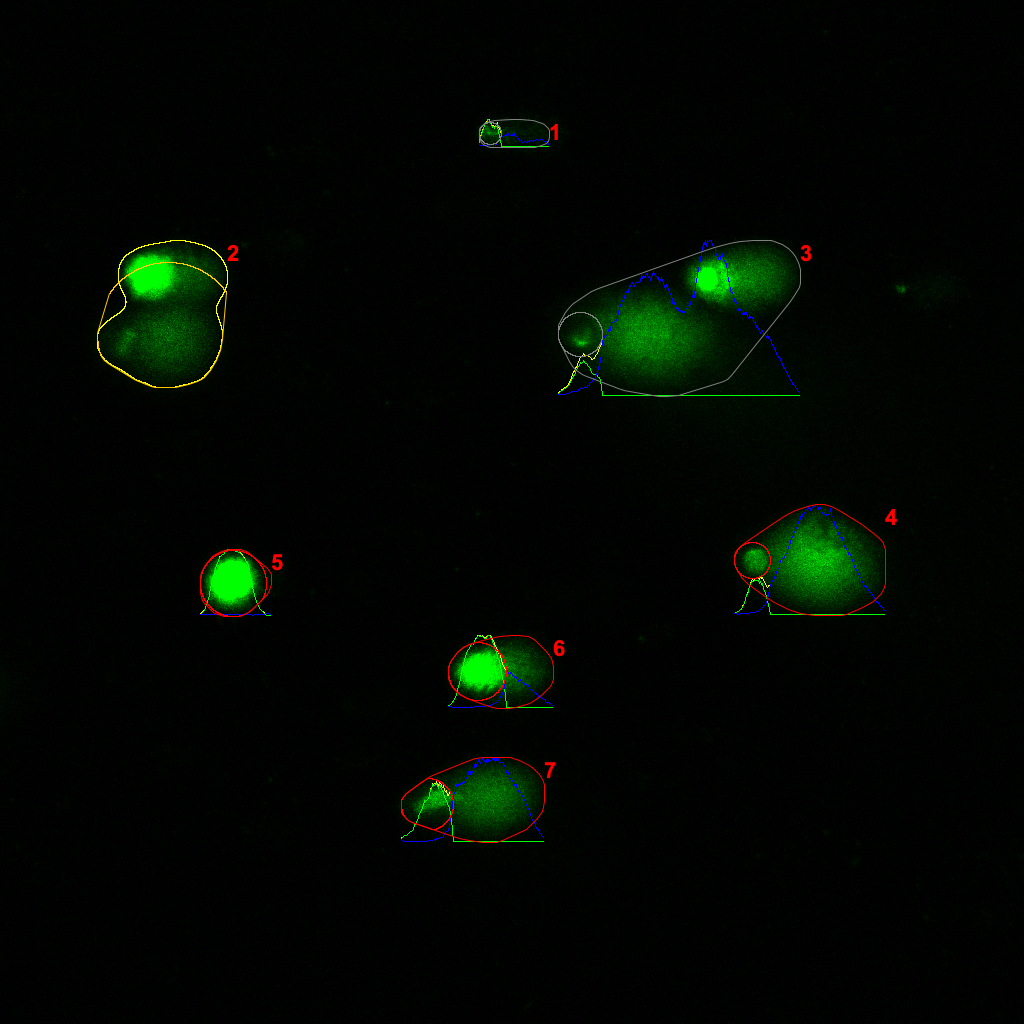

Supplement: Supplementary file 13 — Source data Fig. 6 [file 44321_2026_393_MOESM13_ESM.zip › Figure 6/6B/220915 Comet assay alkaline/output etoposide 1/25_Etoposide_4h_10x_Ccenter15_1AUall_rep1_Maximum.ome.tif_out.tif]

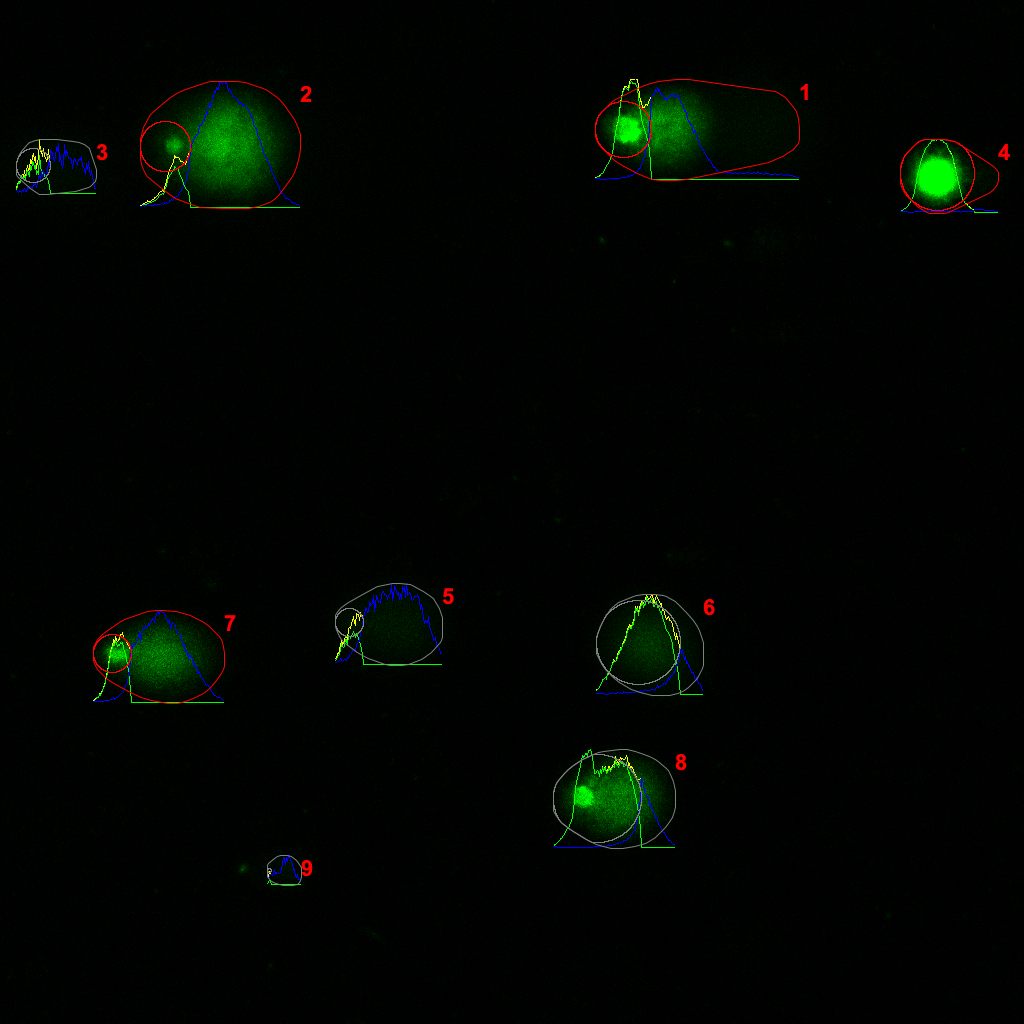

Supplement: Supplementary file 13 — Source data Fig. 6 [file 44321_2026_393_MOESM13_ESM.zip › Figure 6/6B/220915 Comet assay alkaline/output etoposide 1/29_Etoposide_4h_10x_Ccenter15_1AUall_rep1_Maximum.ome.tif_out.tif]

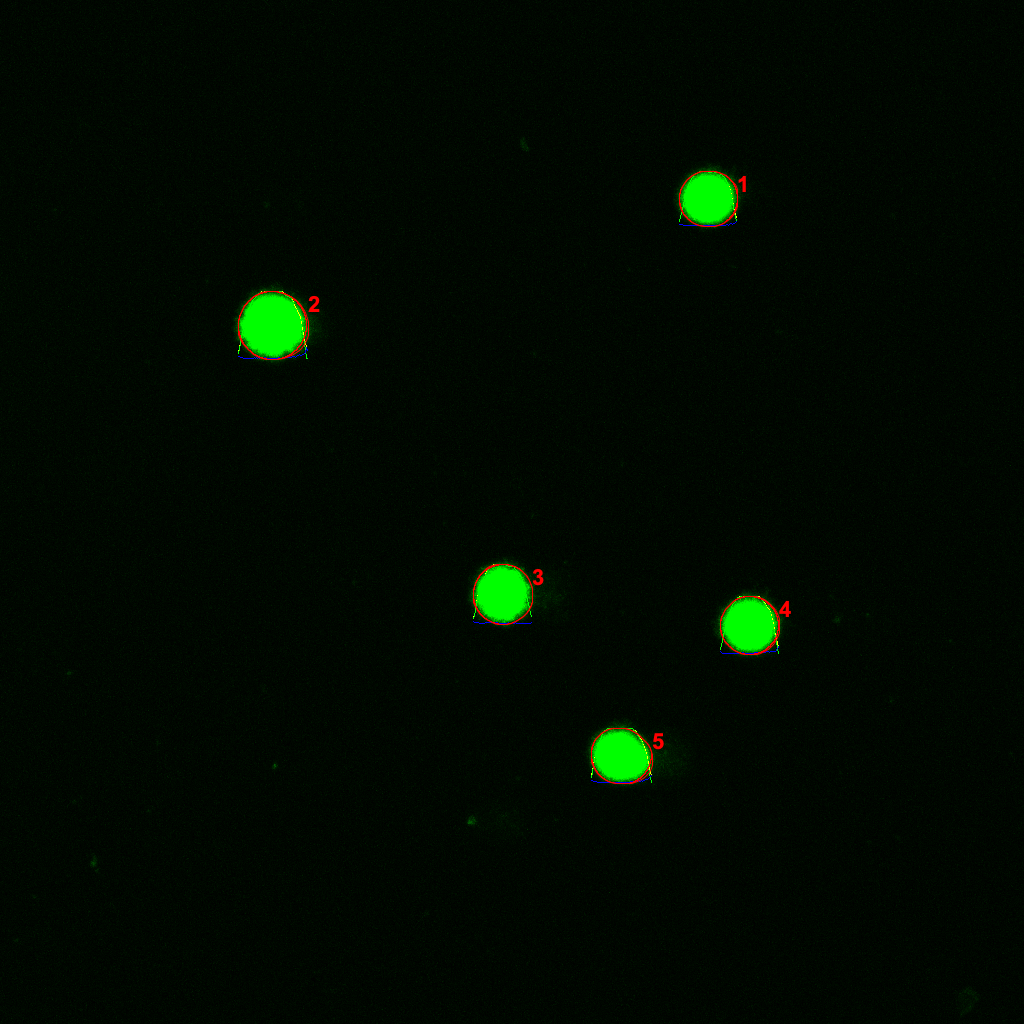

Supplement: Supplementary file 13 — Source data Fig. 6 [file 44321_2026_393_MOESM13_ESM.zip › Figure 6/6B/220915 Comet assay alkaline/output NVP2 6h 2/111_NVP2_6h_10x_Ccenter15_1AUall_rep2_Maximum.ome.tif_out.tif]

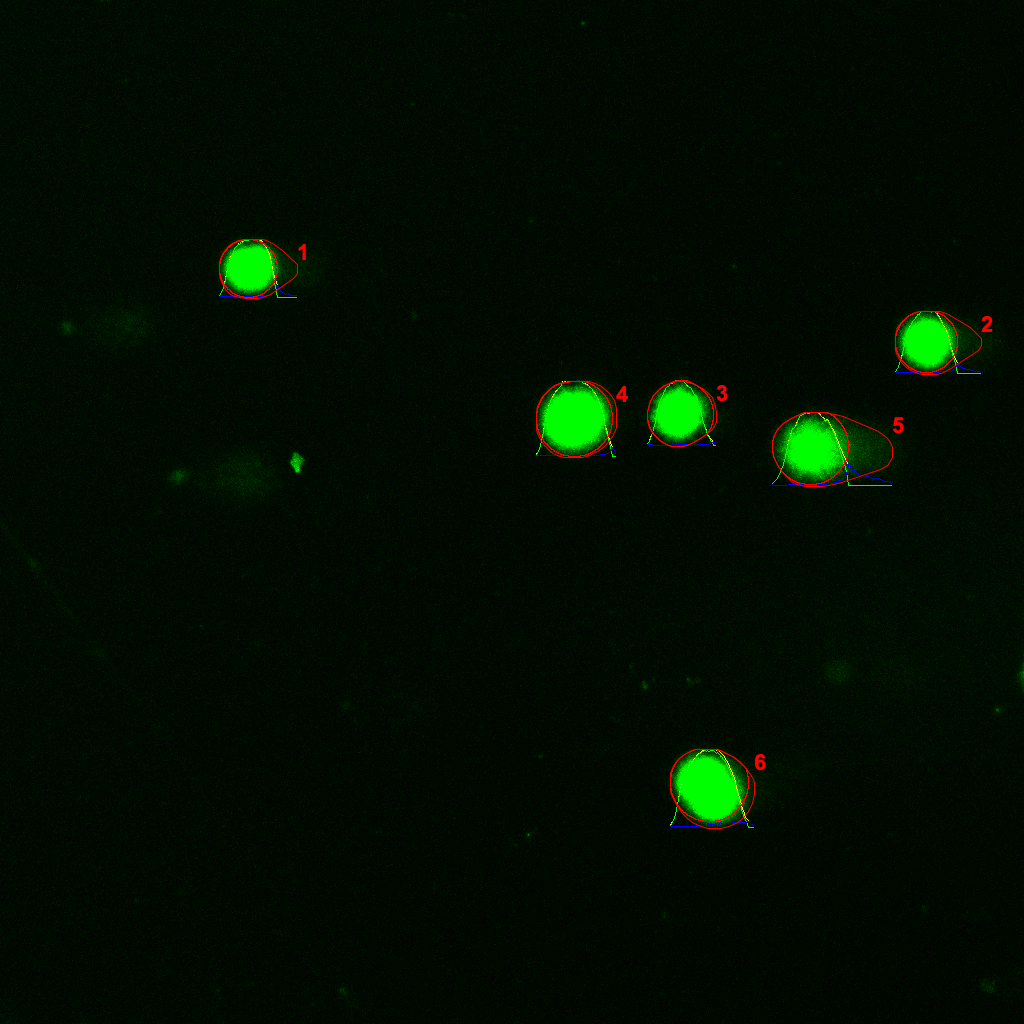

Supplement: Supplementary file 13 — Source data Fig. 6 [file 44321_2026_393_MOESM13_ESM.zip › Figure 6/6B/220915 Comet assay alkaline/output NVP2 6h 2/120_NVP2_6h_10x_Ccenter15_1AUall_rep2_Maximum.ome.tif_out.tif]

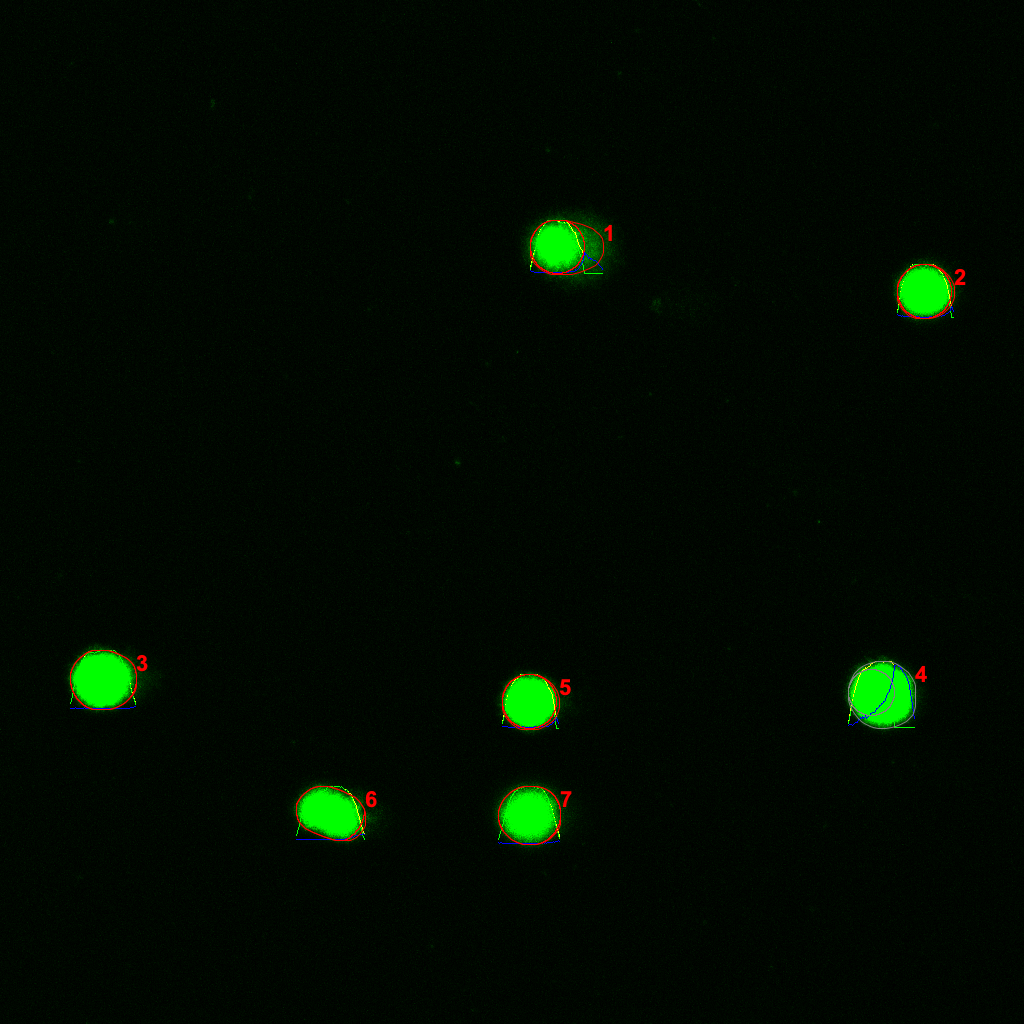

Supplement: Supplementary file 13 — Source data Fig. 6 [file 44321_2026_393_MOESM13_ESM.zip › Figure 6/6B/220915 Comet assay alkaline/output NVP2 6h 2/112_NVP2_6h_10x_Ccenter15_1AUall_rep2_Maximum.ome.tif_out.tif]

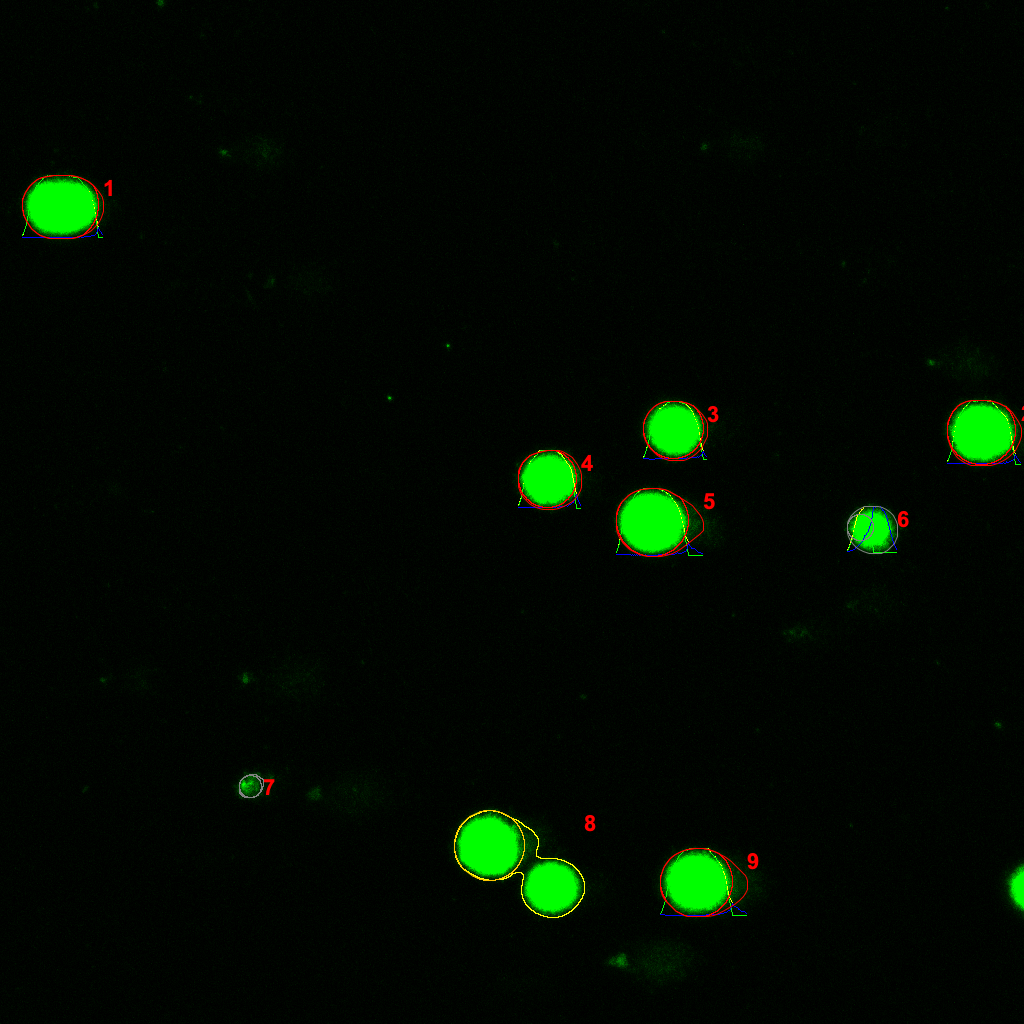

Supplement: Supplementary file 13 — Source data Fig. 6 [file 44321_2026_393_MOESM13_ESM.zip › Figure 6/6B/220915 Comet assay alkaline/output NVP2 6h 2/113_NVP2_6h_10x_Ccenter15_1AUall_rep2_Maximum.ome.tif_out.tif]

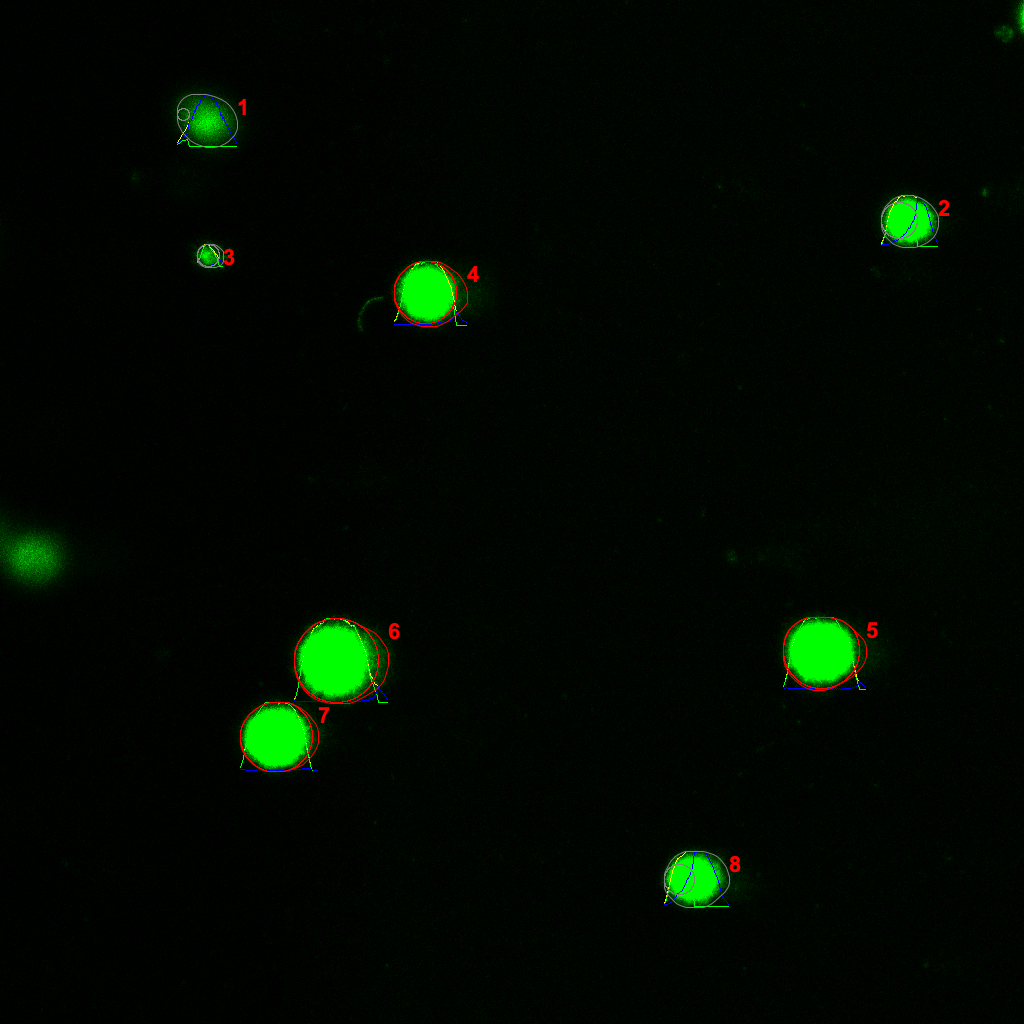

Supplement: Supplementary file 13 — Source data Fig. 6 [file 44321_2026_393_MOESM13_ESM.zip › Figure 6/6B/220915 Comet assay alkaline/output NVP2 6h 2/118_NVP2_6h_10x_Ccenter15_1AUall_rep2_Maximum.ome.tif_out.tif]

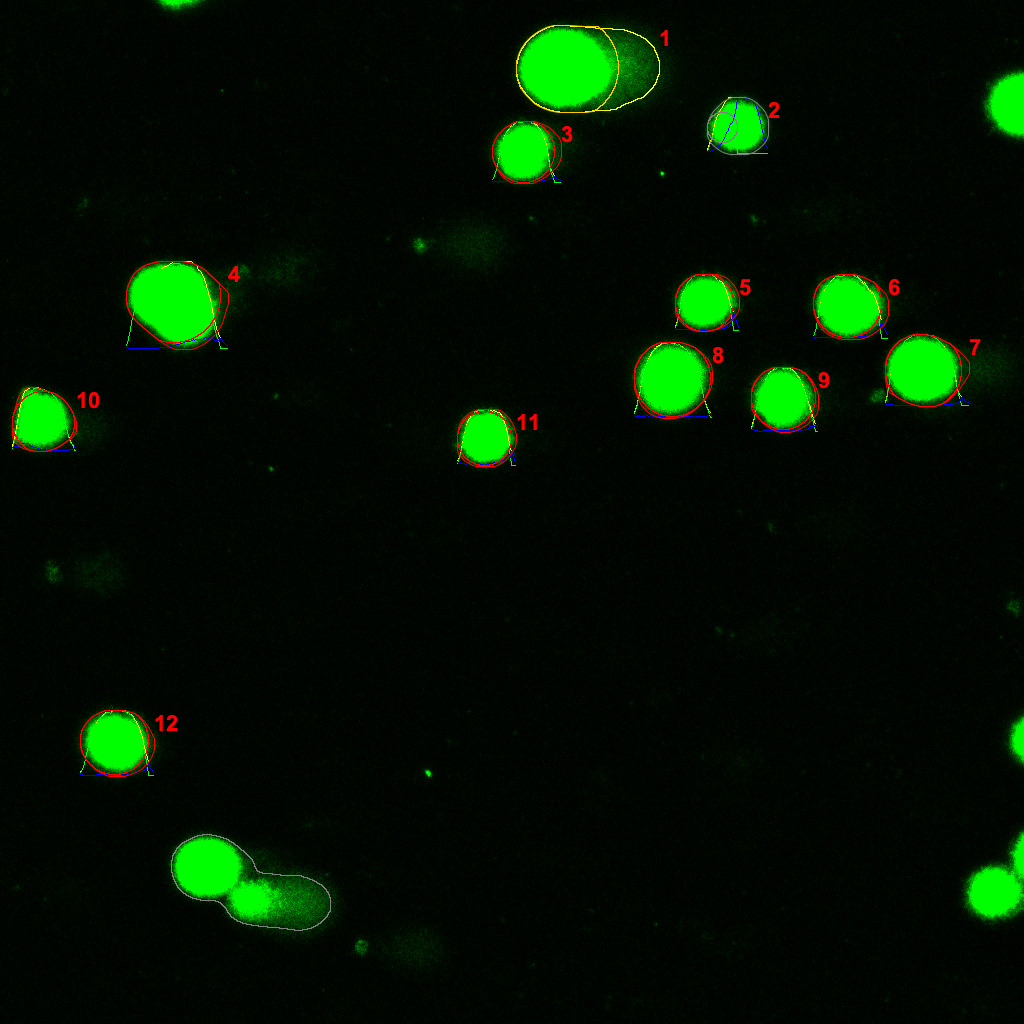

Supplement: Supplementary file 13 — Source data Fig. 6 [file 44321_2026_393_MOESM13_ESM.zip › Figure 6/6B/220915 Comet assay alkaline/output NVP2 6h 2/116_NVP2_6h_10x_Ccenter15_1AUall_rep2_Maximum.ome.tif_out.tif]

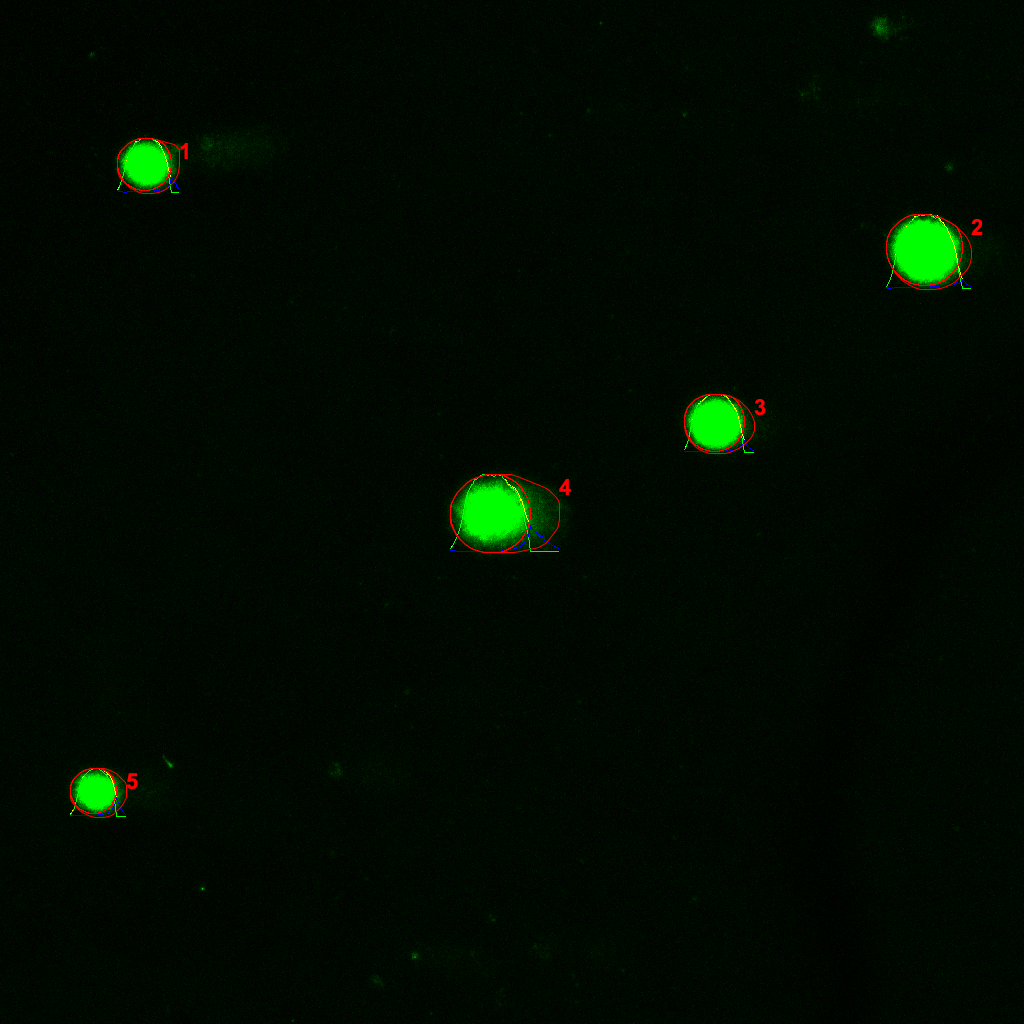

Supplement: Supplementary file 13 — Source data Fig. 6 [file 44321_2026_393_MOESM13_ESM.zip › Figure 6/6B/220915 Comet assay alkaline/output NVP2 6h 2/119_NVP2_6h_10x_Ccenter15_1AUall_rep2_Maximum.ome.tif_out.tif]

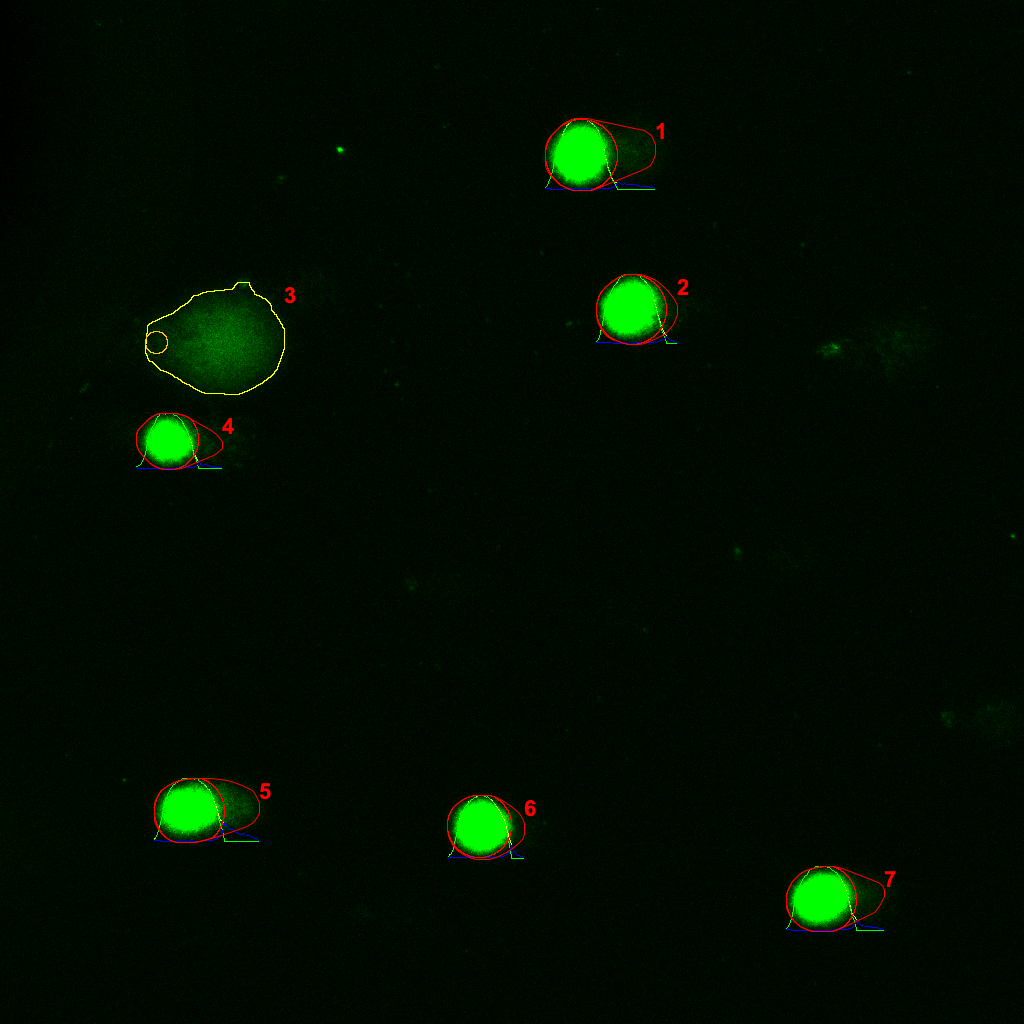

Supplement: Supplementary file 13 — Source data Fig. 6 [file 44321_2026_393_MOESM13_ESM.zip › Figure 6/6B/220915 Comet assay alkaline/output NVP2 6h 2/117_NVP2_6h_10x_Ccenter15_1AUall_rep2_Maximum.ome.tif_out.tif]

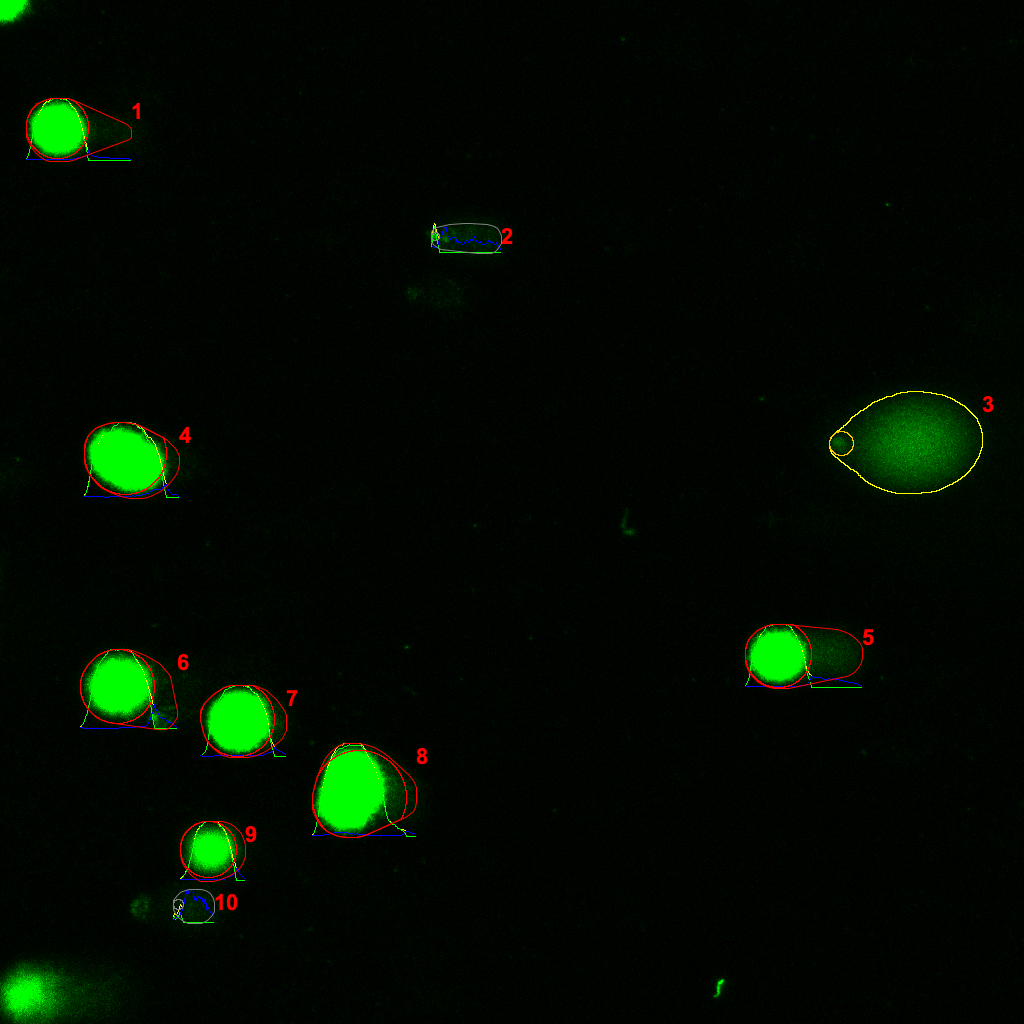

Supplement: Supplementary file 13 — Source data Fig. 6 [file 44321_2026_393_MOESM13_ESM.zip › Figure 6/6B/220915 Comet assay alkaline/output NVP2 6h 2/115_NVP2_6h_10x_Ccenter15_1AUall_rep2_Maximum.ome.tif_out.tif]

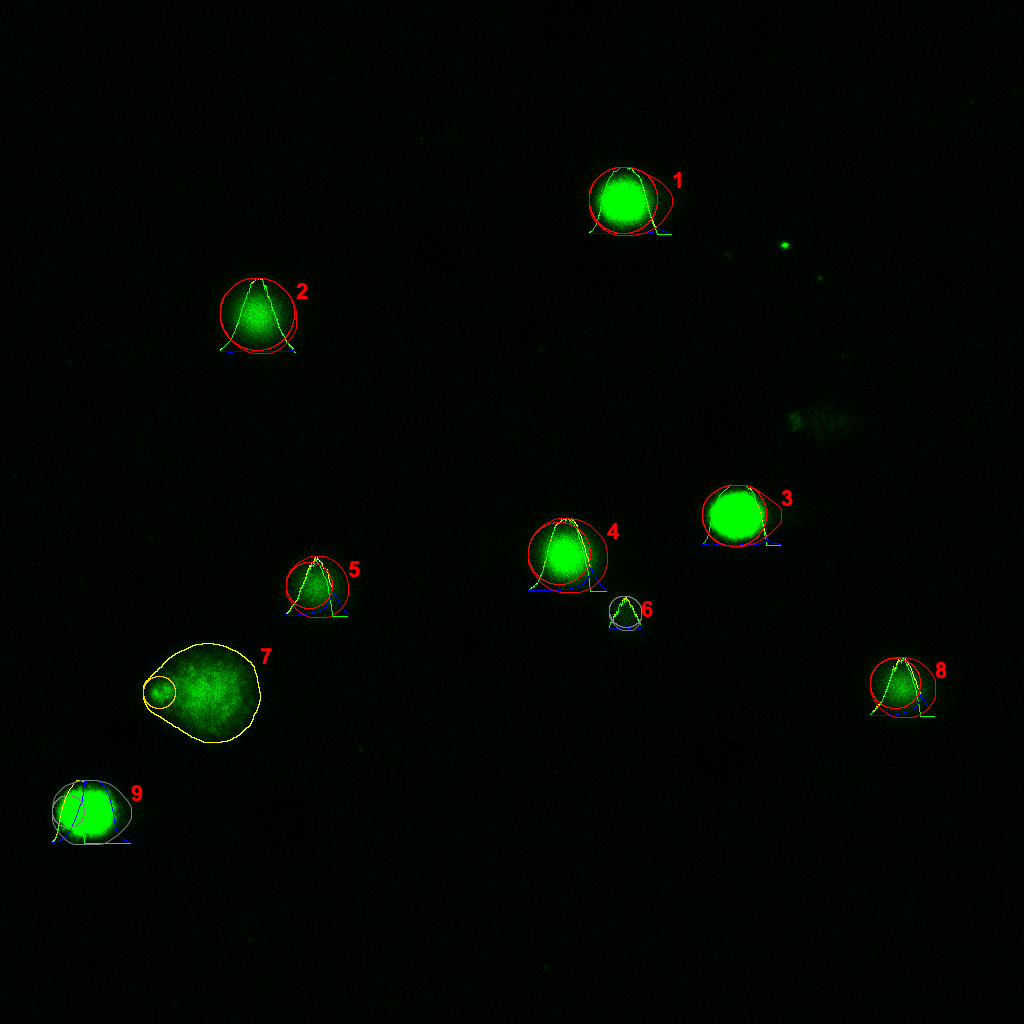

Supplement: Supplementary file 13 — Source data Fig. 6 [file 44321_2026_393_MOESM13_ESM.zip › Figure 6/6B/220915 Comet assay alkaline/output NVP2 6h 2/114_NVP2_6h_10x_Ccenter15_1AUall_rep2_Maximum.ome.tif_out.tif]

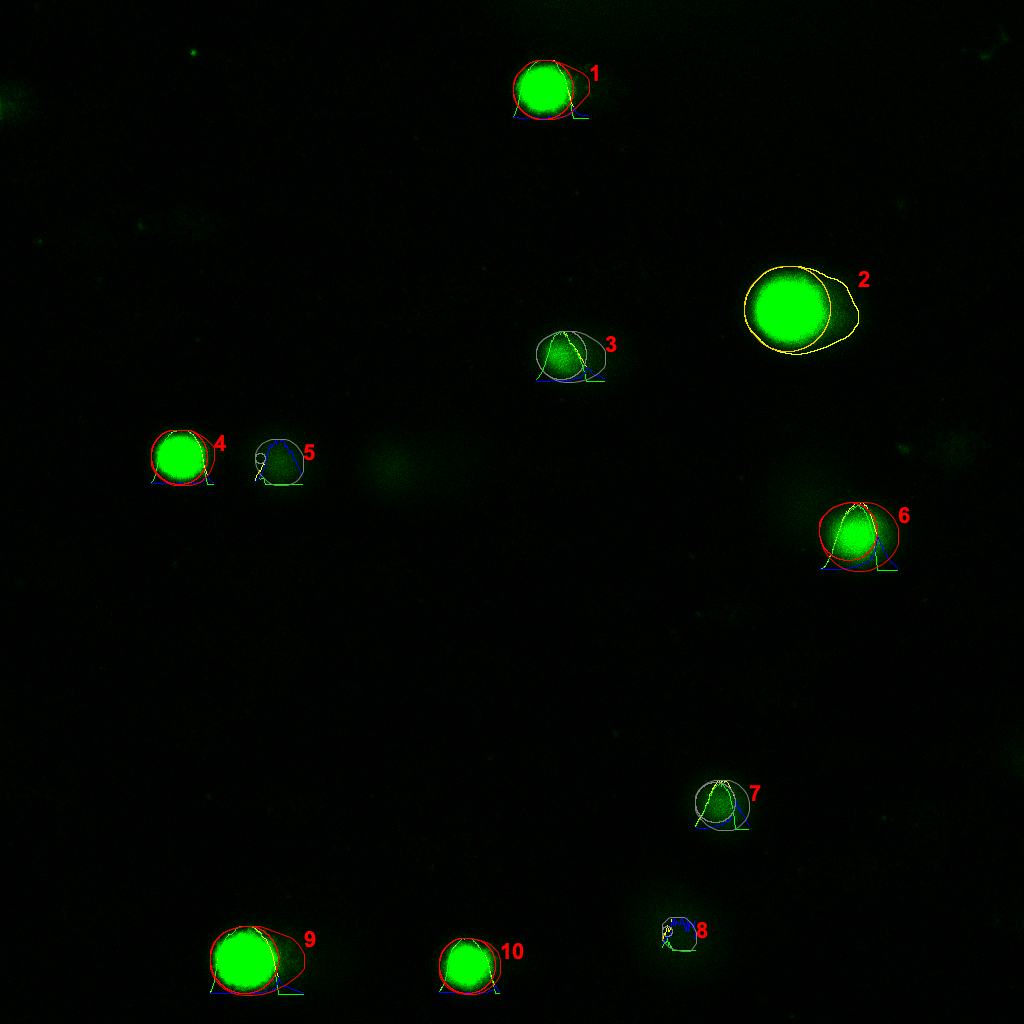

Supplement: Supplementary file 13 — Source data Fig. 6 [file 44321_2026_393_MOESM13_ESM.zip › Figure 6/6B/220915 Comet assay alkaline/output THZ531 6h 2/75_THZ531_6h_10x_Ccenter15_1AUall_rep2_Maximum.ome.tif_out.tif]

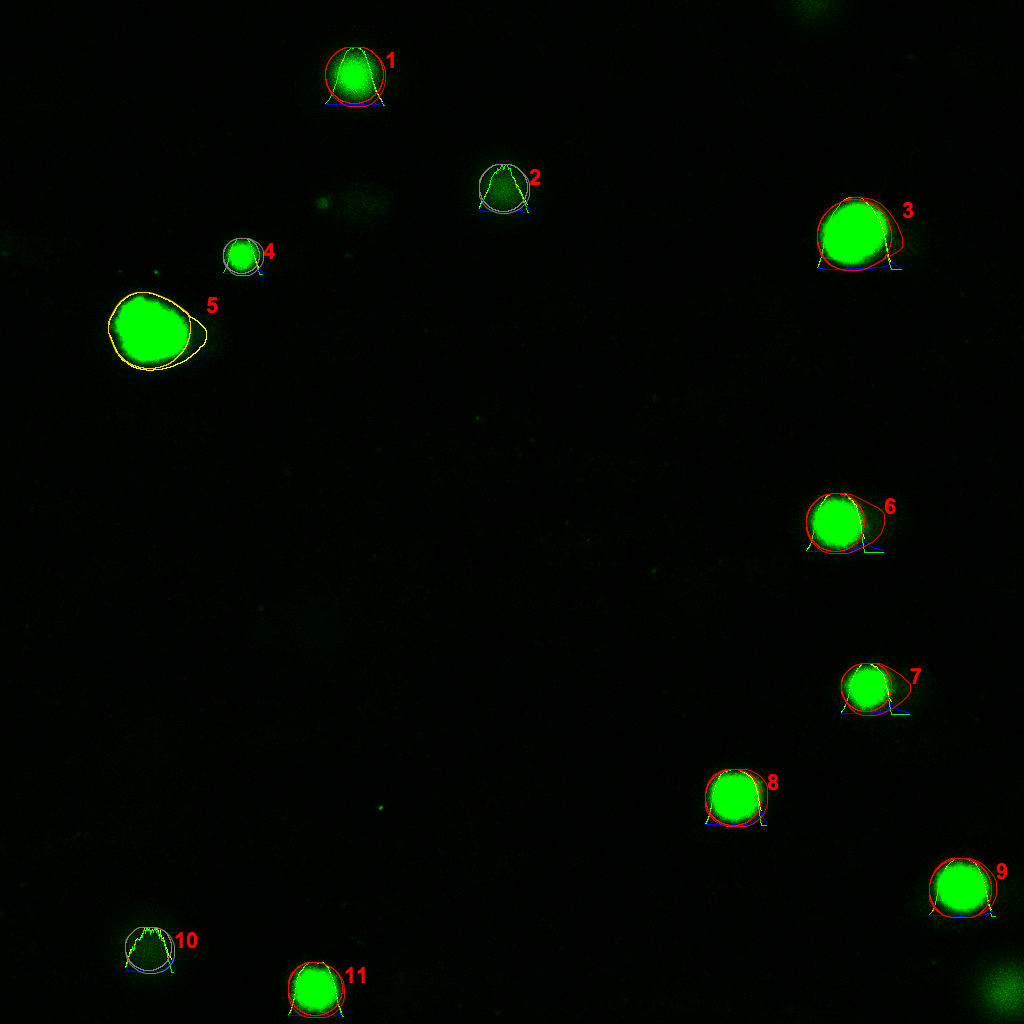

Supplement: Supplementary file 13 — Source data Fig. 6 [file 44321_2026_393_MOESM13_ESM.zip › Figure 6/6B/220915 Comet assay alkaline/output THZ531 6h 2/77_THZ531_6h_10x_Ccenter15_1AUall_rep2_Maximum.ome.tif_out.tif]

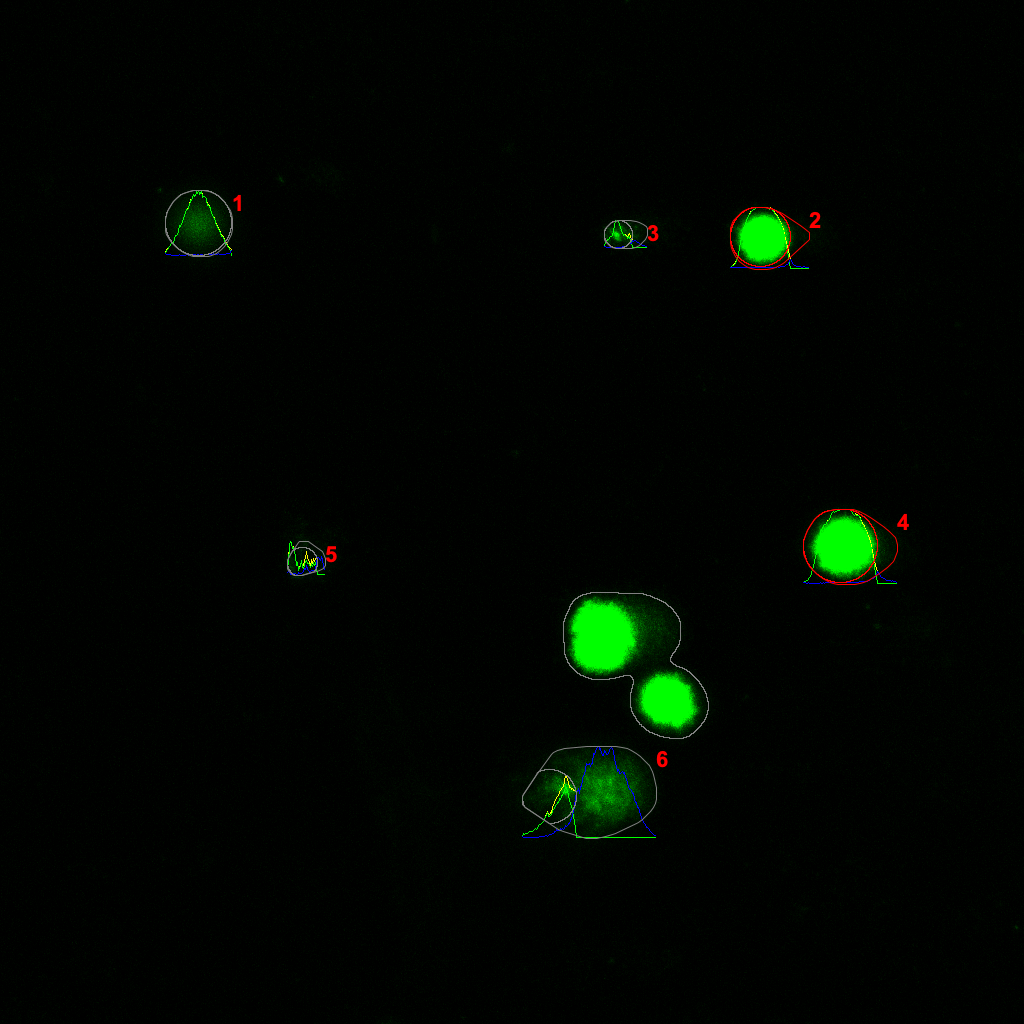

Supplement: Supplementary file 13 — Source data Fig. 6 [file 44321_2026_393_MOESM13_ESM.zip › Figure 6/6B/220915 Comet assay alkaline/output THZ531 6h 2/79_THZ531_6h_10x_Ccenter15_1AUall_rep2_Maximum.ome.tif_out.tif]

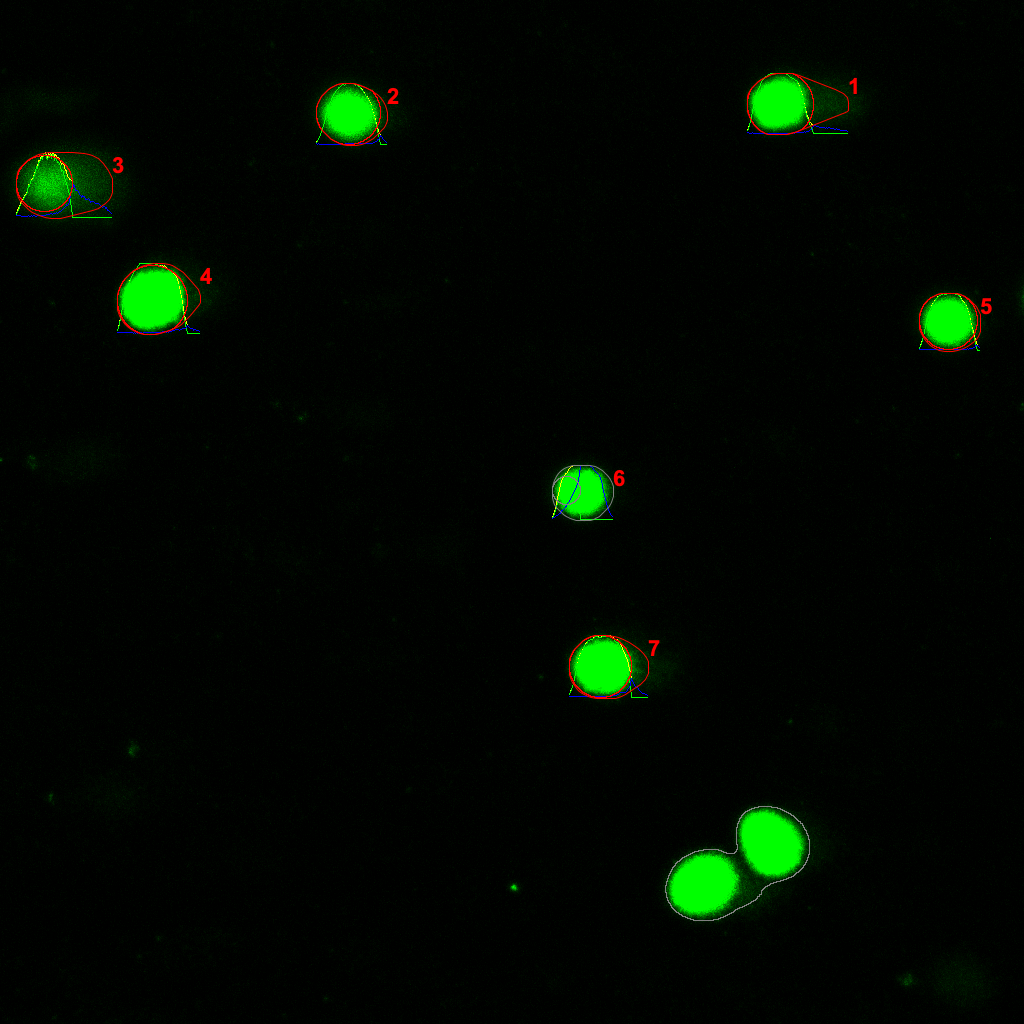

Supplement: Supplementary file 13 — Source data Fig. 6 [file 44321_2026_393_MOESM13_ESM.zip › Figure 6/6B/220915 Comet assay alkaline/output THZ531 6h 2/74_THZ531_6h_10x_Ccenter15_1AUall_rep2_Maximum.ome.tif_out.tif]

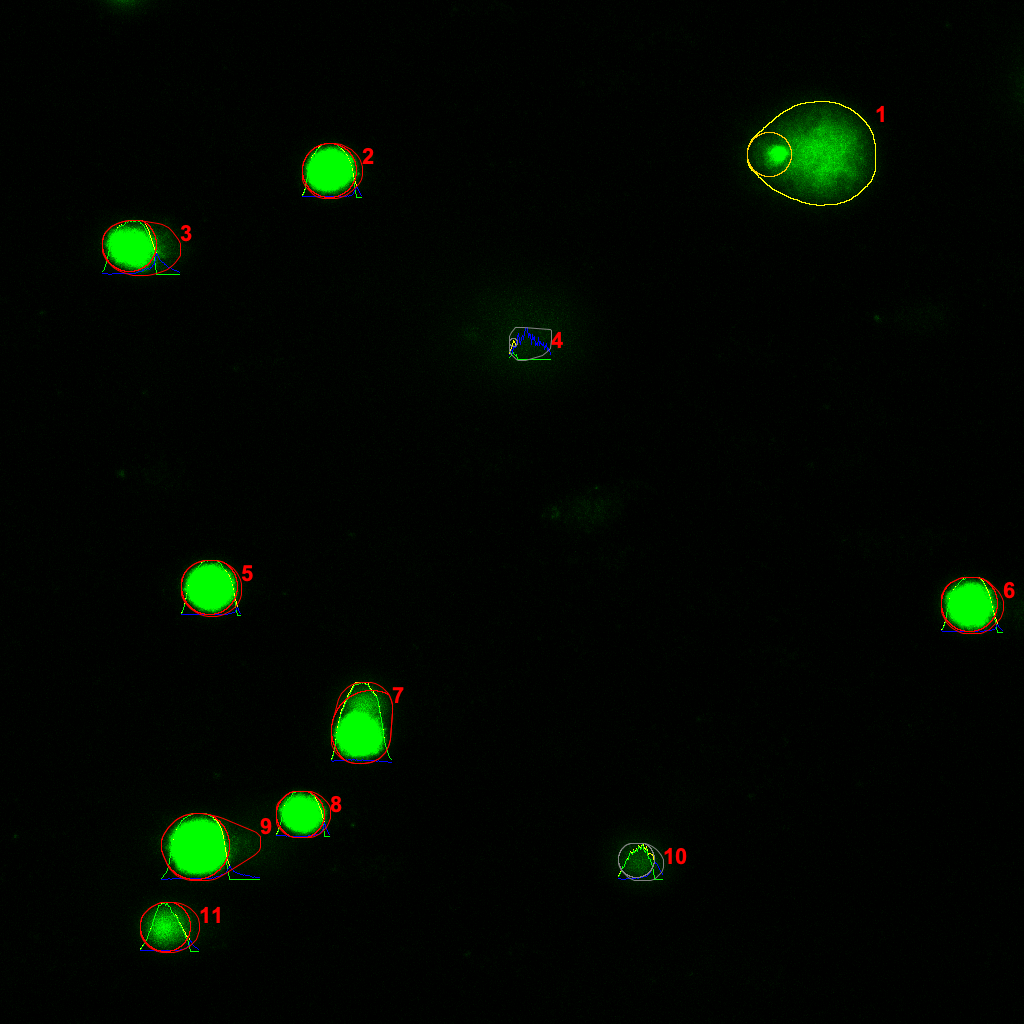

Supplement: Supplementary file 13 — Source data Fig. 6 [file 44321_2026_393_MOESM13_ESM.zip › Figure 6/6B/220915 Comet assay alkaline/output THZ531 6h 2/78_THZ531_6h_10x_Ccenter15_1AUall_rep2_Maximum.ome.tif_out.tif]

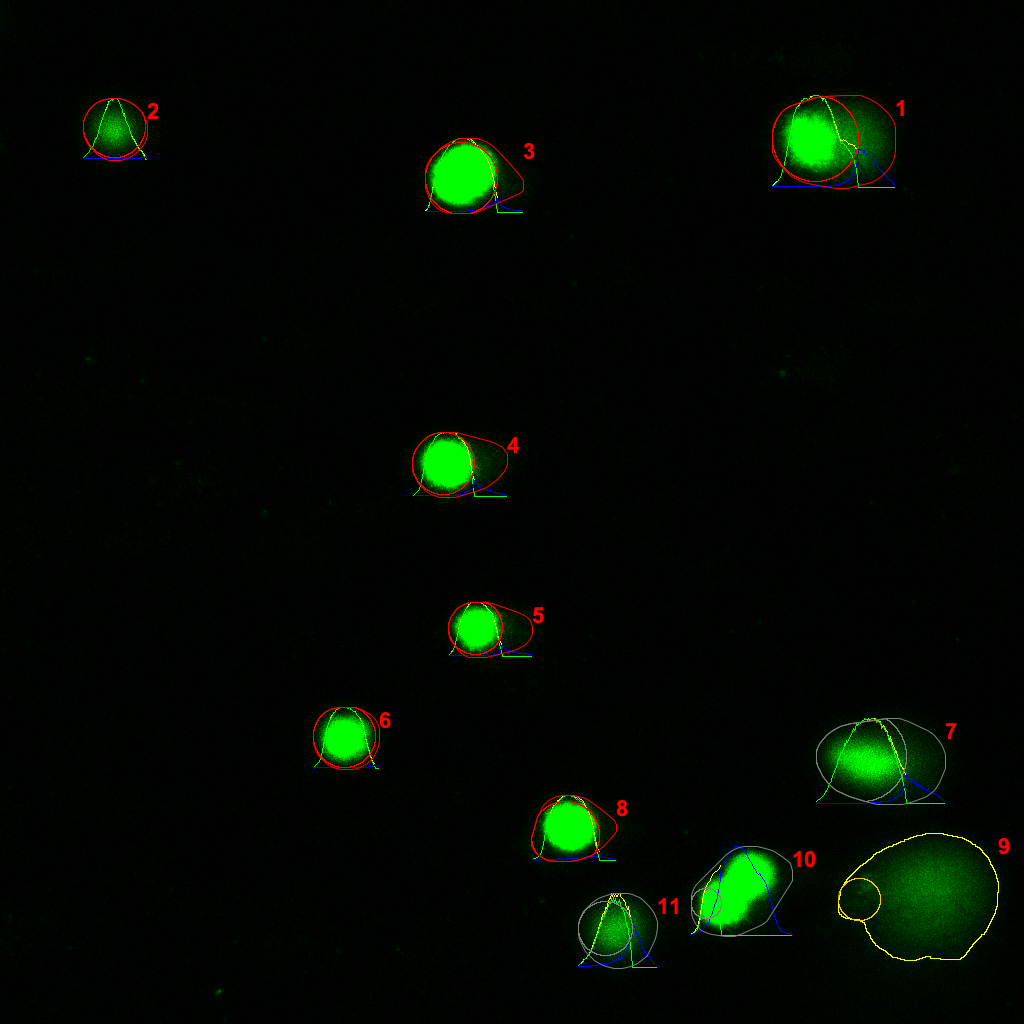

Supplement: Supplementary file 13 — Source data Fig. 6 [file 44321_2026_393_MOESM13_ESM.zip › Figure 6/6B/220915 Comet assay alkaline/output THZ531 6h 2/76_THZ531_6h_10x_Ccenter15_1AUall_rep2_Maximum.ome.tif_out.tif]

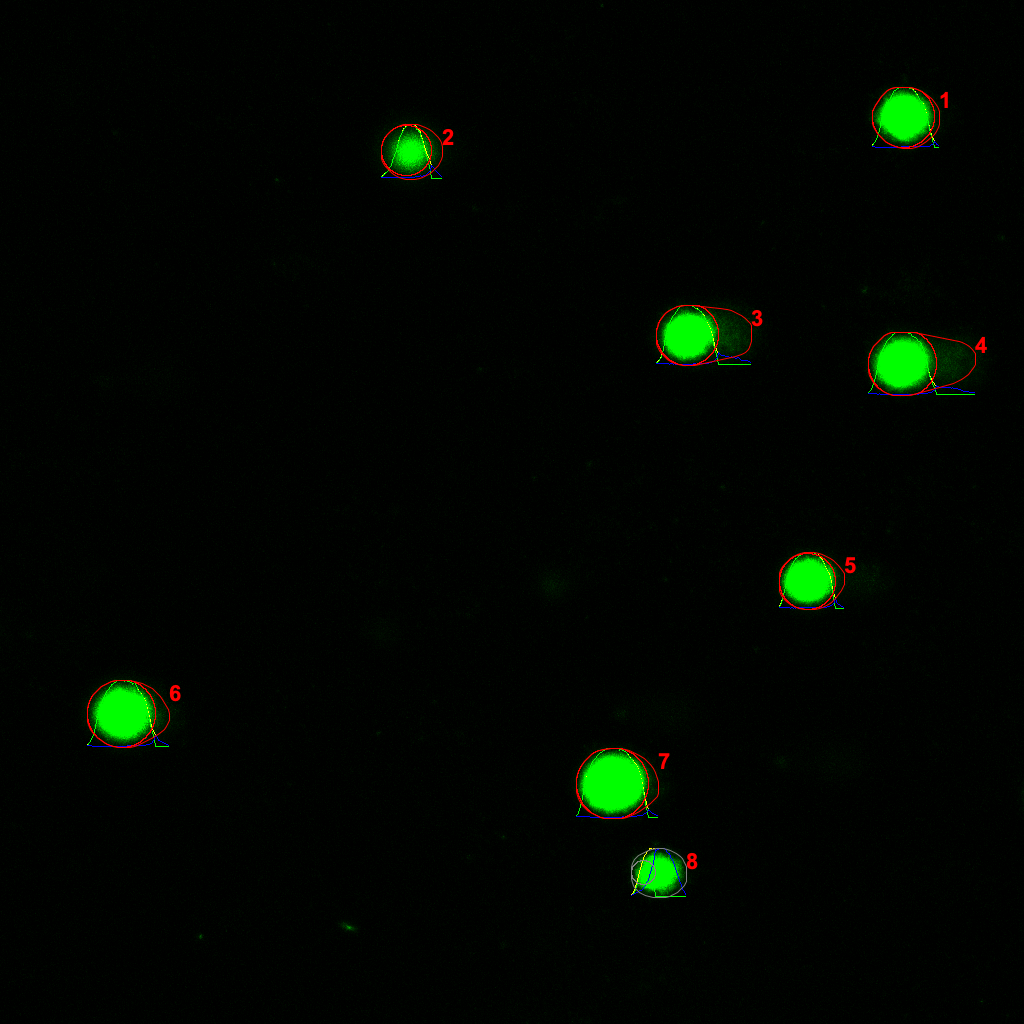

Supplement: Supplementary file 13 — Source data Fig. 6 [file 44321_2026_393_MOESM13_ESM.zip › Figure 6/6B/220915 Comet assay alkaline/output THZ531 6h 2/80_THZ531_6h_10x_Ccenter15_1AUall_rep2_Maximum.ome.tif_out.tif]

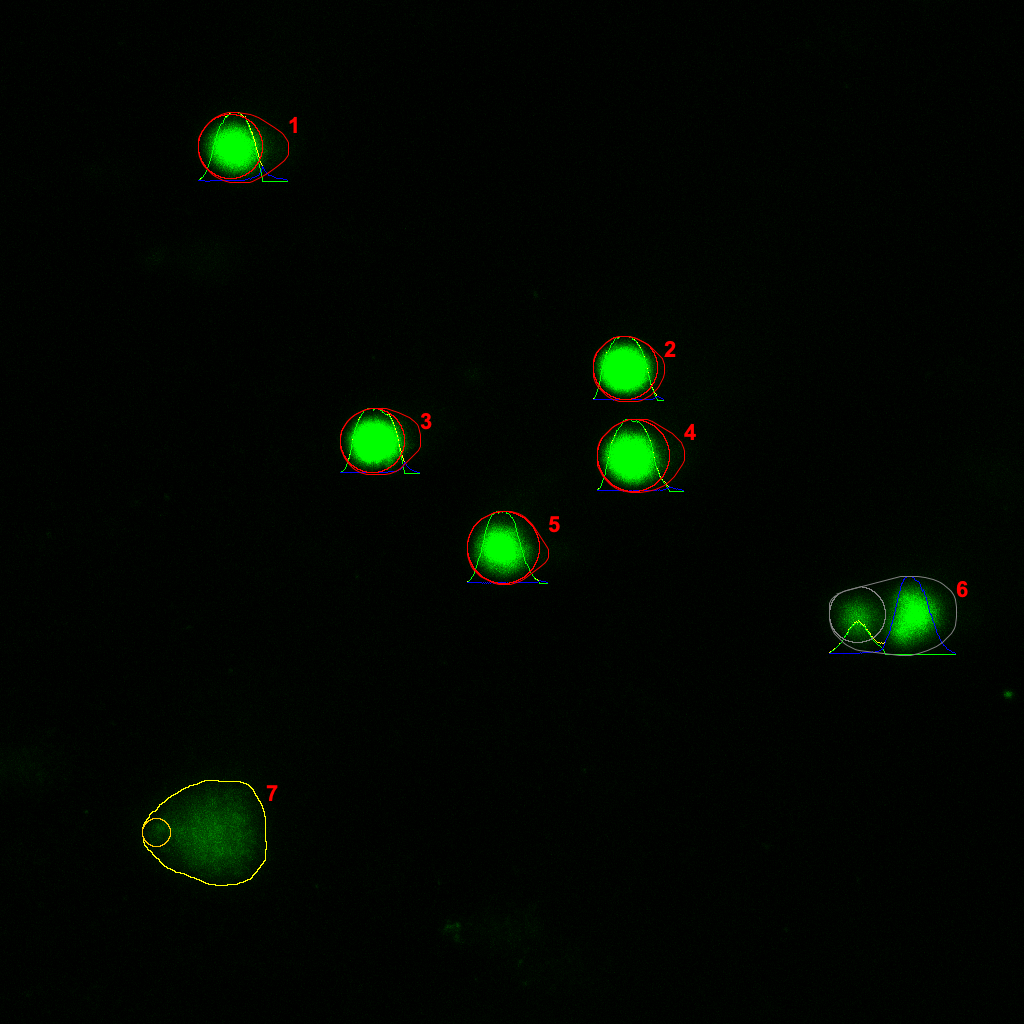

Supplement: Supplementary file 13 — Source data Fig. 6 [file 44321_2026_393_MOESM13_ESM.zip › Figure 6/6B/220915 Comet assay alkaline/output THZ531 6h 2/72_THZ531_6h_10x_Ccenter15_1AUall_rep2_Maximum.ome.tif_out.tif]

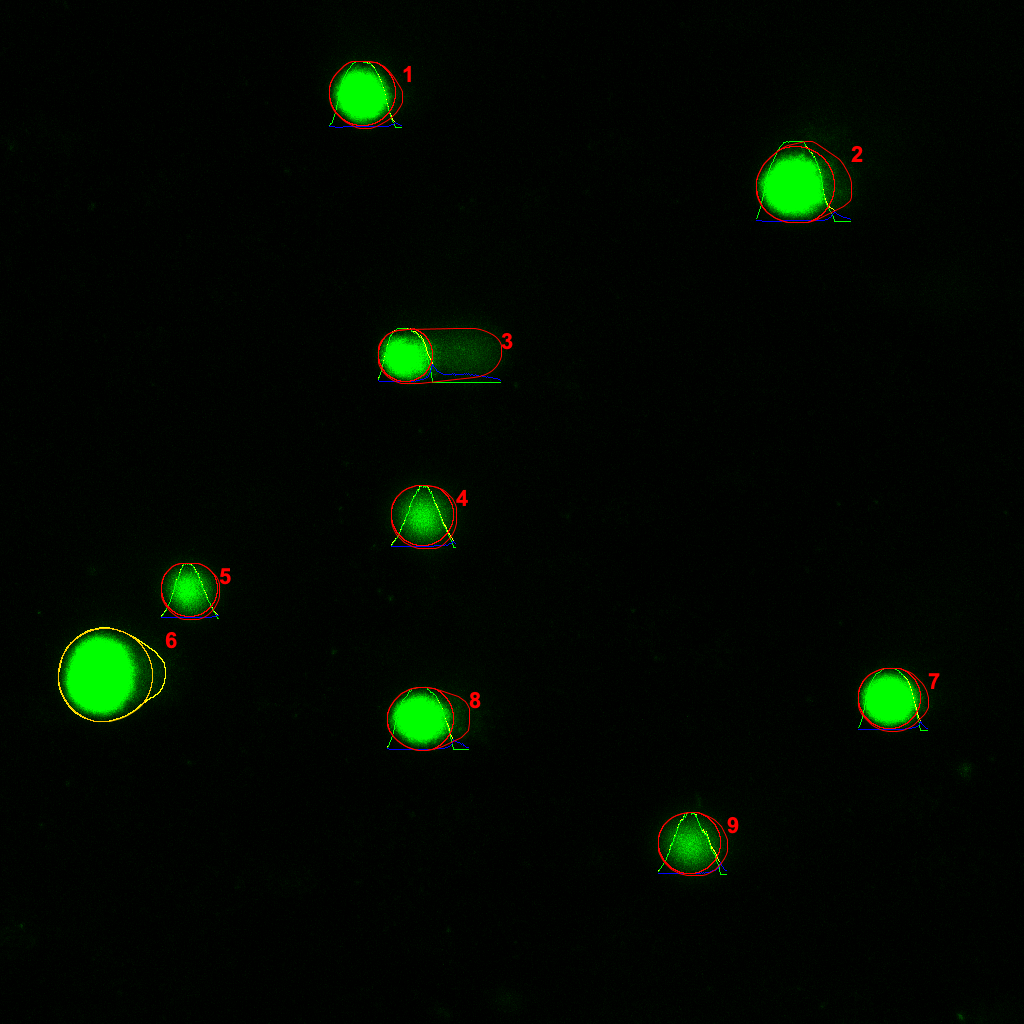

Supplement: Supplementary file 13 — Source data Fig. 6 [file 44321_2026_393_MOESM13_ESM.zip › Figure 6/6B/220915 Comet assay alkaline/output THZ531 6h 2/73_THZ531_6h_10x_Ccenter15_1AUall_rep2_Maximum.ome.tif_out.tif]

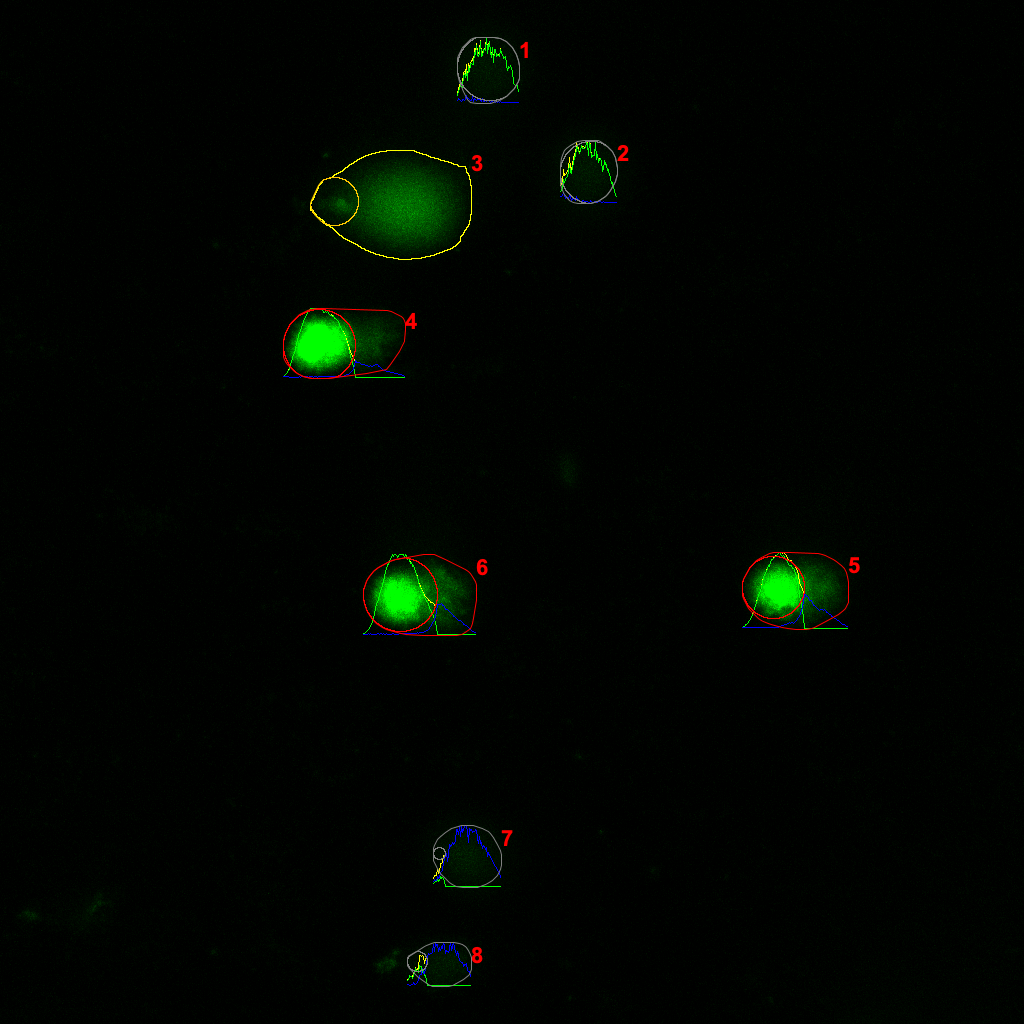

Supplement: Supplementary file 13 — Source data Fig. 6 [file 44321_2026_393_MOESM13_ESM.zip › Figure 6/6B/220915 Comet assay alkaline/output THZ531 6h 2/71_THZ531_6h_10x_Ccenter15_1AUall_rep2_Maximum.ome.tif_out.tif]

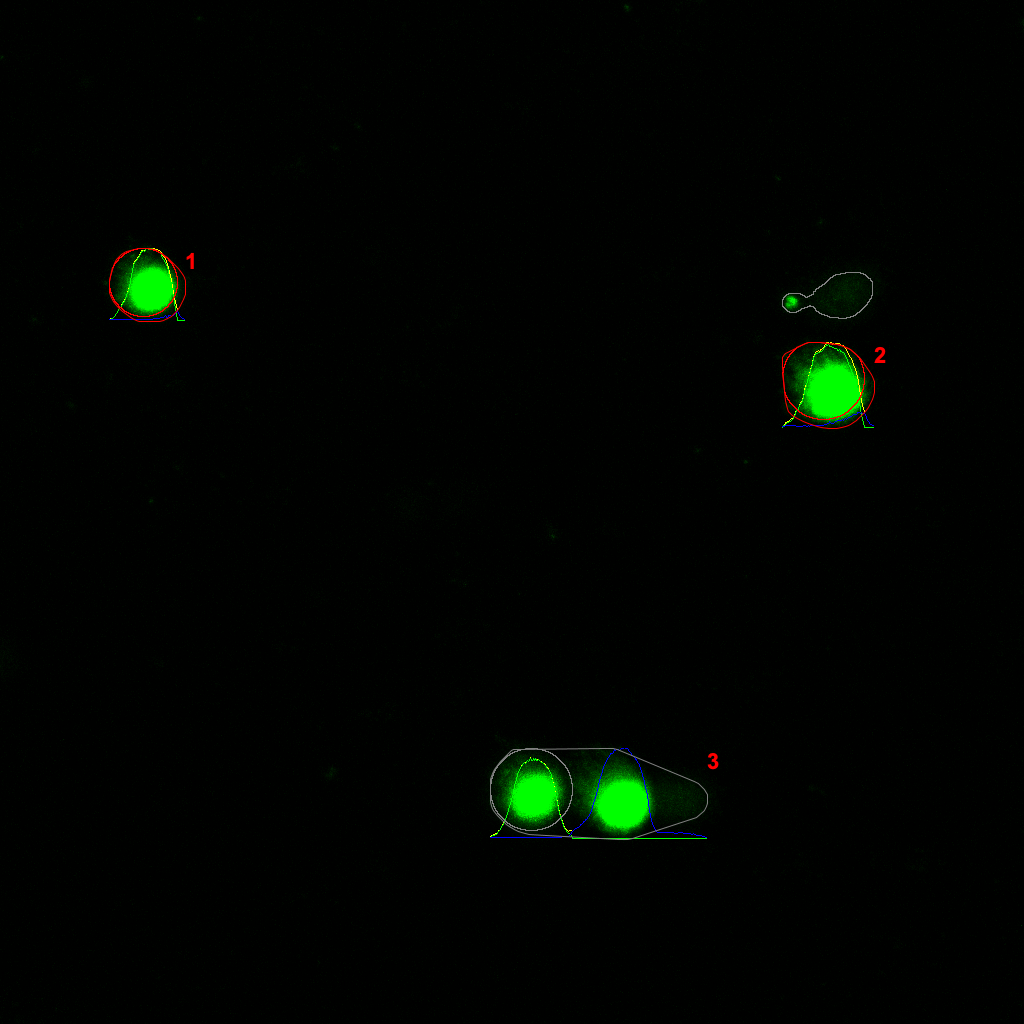

Supplement: Supplementary file 13 — Source data Fig. 6 [file 44321_2026_393_MOESM13_ESM.zip › Figure 6/6B/220915 Comet assay alkaline/output DMSO 2/18_DMSO_6h_10x_Ccenter15_1AUall_rep2_Maximum.ome.tif_out.tif]

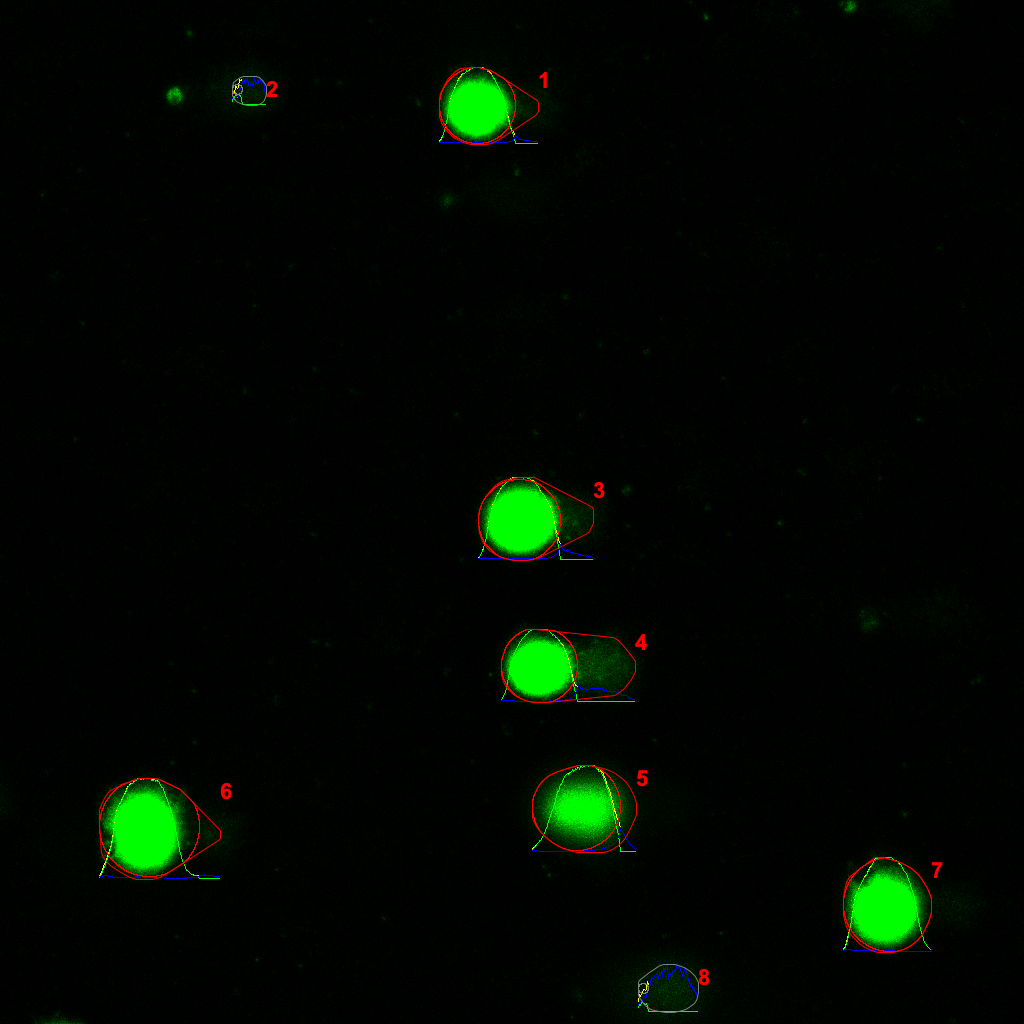

Supplement: Supplementary file 13 — Source data Fig. 6 [file 44321_2026_393_MOESM13_ESM.zip › Figure 6/6B/220915 Comet assay alkaline/output DMSO 2/16_DMSO_6h_10x_Ccenter15_1AUall_rep2_Maximum.ome.tif_out.tif]

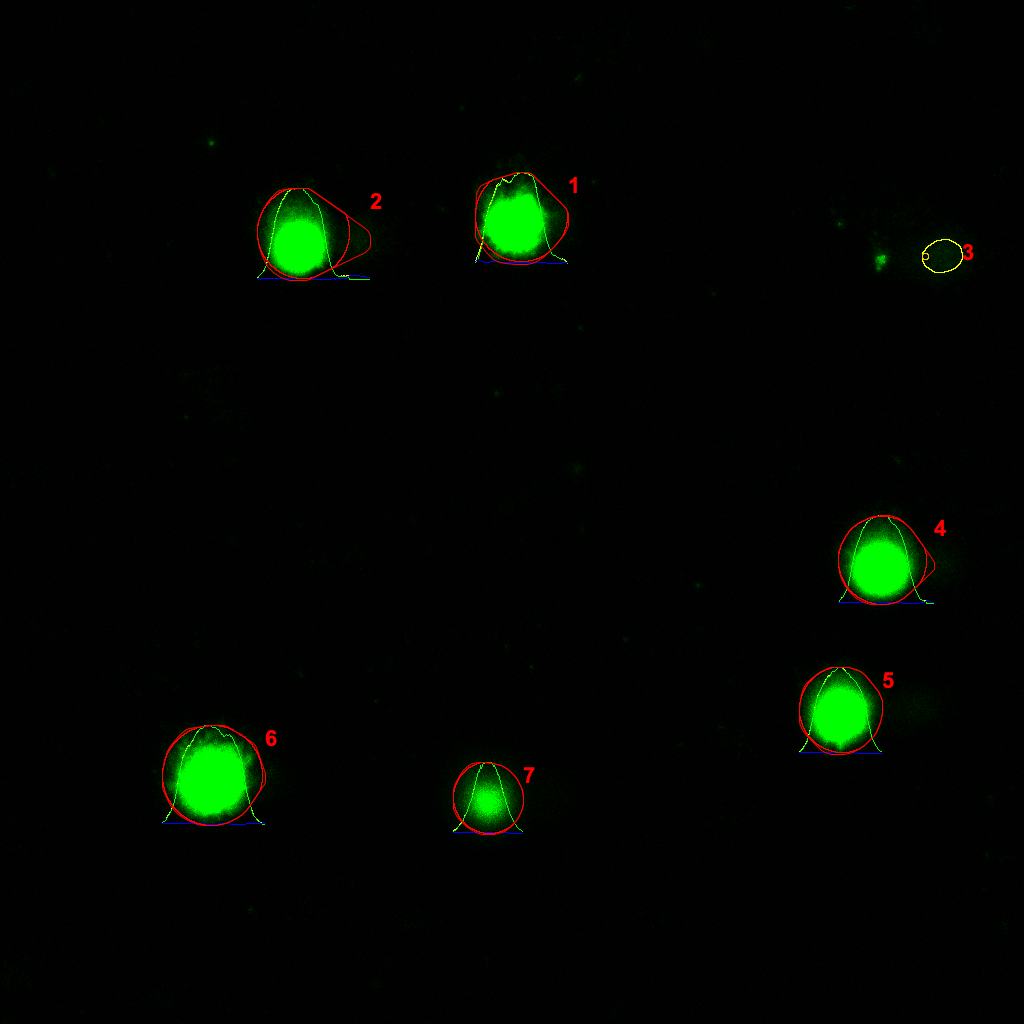

Supplement: Supplementary file 13 — Source data Fig. 6 [file 44321_2026_393_MOESM13_ESM.zip › Figure 6/6B/220915 Comet assay alkaline/output DMSO 2/19_DMSO_6h_10x_Ccenter15_1AUall_rep2_Maximum.ome.tif_out.tif]
