# Supplementary material for: CDK12/CDK13 inhibition disrupts transcriptional elongation and replication fork progression in glioblastoma
Source: EMBO Mol Med. 2026 Mar 25;18(5):1592–624. doi: 10.1038/s44321-026-00393-w (PMC13179391; doi:10.1038/s44321-026-00393-w)
Supplement: Supplementary file 9 — Source data Fig. 2 [file 44321_2026_393_MOESM9_ESM.zip › Figure 2/2A/Readme.rtf]

README – Figure 2A (CDK12 IHC in Control and Glioblastoma Tissue)Files IncludedControl tissue:2A Control tissue C12_CDK12.tif, 2A Control tissue C12_CDK12 close-up_40x.tifGlioblastoma tissue: 2A Glioblastoma C17_CDK12.tif, 2A Glioblastoma C17_CDK12 close-up_40x.tif, 2A Glioblastoma C19_CDK12.tif, 2A Glioblastoma C19 CDK12 close-up_40x.tif, 2A Glioblastoma C21_CDK12.tif, 2A Glioblastoma C21_CDK12 close-up_40x.tifDescription: These files contain the raw immunohistochemistry (IHC) images used in Figure 2A showing CDK12 expression in non-neoplastic control brain tissue, and glioblastoma patient samples (patients C17, C19, C21; patient IDs correspond to Appendix Table S1 in the manuscript).
