# Supplementary material for: CDK12/CDK13 inhibition disrupts transcriptional elongation and replication fork progression in glioblastoma
Source: EMBO Mol Med. 2026 Mar 25;18(5):1592–624. doi: 10.1038/s44321-026-00393-w (PMC13179391; doi:10.1038/s44321-026-00393-w)
Supplement: Supplementary file 9 — Source data Fig. 2 [file 44321_2026_393_MOESM9_ESM.zip › Figure 2/2C/Readme.rtf]

README – Figure 2C Description:This folder contains the raw TIFF images used for Figure 2C, showing representative organoids treated with different inhibitors (Abemaciclib, Lomustine, SR-4835, THZ531) at various concentrations and at two timepoints: 0 h (baseline), 72 h post-treatmentThese images illustrate the morphological effects of each drug on patient-derived organoids.File Naming Convention: Each file follows: 2C_<Drug>_<Timepoint>_<Dose>.tiffWhere: Drug = Abemaciclib, Lomustine, SR4835, THZ531Timepoint = 0h or 72hDose = concentration in µM, e.g. 0.5uM, 2uM, 32uM, or Control
