# Supplementary material for: CDK12/CDK13 inhibition disrupts transcriptional elongation and replication fork progression in glioblastoma
Source: EMBO Mol Med. 2026 Mar 25;18(5):1592–624. doi: 10.1038/s44321-026-00393-w (PMC13179391; doi:10.1038/s44321-026-00393-w)
Supplement: Supplementary file 9 — Source data Fig. 2 [file 44321_2026_393_MOESM9_ESM.zip › Figure 2/2D/Readme.rtf]

README – Figure 2D Files included: 2D P3.xlsx, 2D T188.xlsx, 2D T434.xlsx, 2D T470.xlsxDescription: These Excel files contain the raw viability measurements used to generate the dose–response curves shown in Figure 2D. Each file corresponds to a single patient-derived organoid model treated with a panel of inhibitors.The data represent % viability normalized to DMSO controls, measured after 72 h treatment.Data Structure: Each Excel file includesDose (µM) values for each inhibitorTriplicate viability measurements (n = 3 per treatment dose)Normalized viability (%)DMSO control measurements (n = 18 pooled)
