# Supplementary material for: CDK12/CDK13 inhibition disrupts transcriptional elongation and replication fork progression in glioblastoma
Source: EMBO Mol Med. 2026 Mar 25;18(5):1592–624. doi: 10.1038/s44321-026-00393-w (PMC13179391; doi:10.1038/s44321-026-00393-w)
Supplement: Supplementary file 9 — Source data Fig. 2 [file 44321_2026_393_MOESM9_ESM.zip › Figure 2/2E/Readme.rtf]

README – Figure 2E (IC50 Values for Organoid Models)File included: 2E_IC50.csvDescription: This CSV file contains the IC50 values (µM) for four patient-derived organoid models (P3, T188, T434, T470) treated with the inhibitors used in Figure 2C–D: THZ531, SR-4835, Abemaciclib, LomustineThese IC50 values were used to generate the dot plot shown in Figure 2E.
