# Supplementary material for: CDK12/CDK13 inhibition disrupts transcriptional elongation and replication fork progression in glioblastoma
Source: EMBO Mol Med. 2026 Mar 25;18(5):1592–624. doi: 10.1038/s44321-026-00393-w (PMC13179391; doi:10.1038/s44321-026-00393-w)
Supplement: Supplementary file 10 — Source data Fig. 3 [file 44321_2026_393_MOESM10_ESM.zip › Figure 3/3E/Readme.rtf]

README – Figure 3E (U87-MG Proliferation Assay)File: 3E_U87MG_THZ531_proliferation.csvDescription: This file contains the raw proliferation assay data used to generate Figure 3E, showing the effect of SR-4835 treatment on U87-MG glioma cells in vitro.Cell proliferation was measured over time under the indicated treatment conditions.
