# Supplementary material for: CDK12/CDK13 inhibition disrupts transcriptional elongation and replication fork progression in glioblastoma
Source: EMBO Mol Med. 2026 Mar 25;18(5):1592–624. doi: 10.1038/s44321-026-00393-w (PMC13179391; doi:10.1038/s44321-026-00393-w)
Supplement: Supplementary file 10 — Source data Fig. 3 [file 44321_2026_393_MOESM10_ESM.zip › Figure 3/3B/Readme.rtf]

README – Figure 3B (Brain Slice Invasion Assay – Raw Microscopy Data)Description: This folder contains the raw confocal microscopy image files (.czi) used for Figure 3B, illustrating the invasion of H2B-mCherry–expressing G7 spheroids into murine organotypic brain slices under control and THZ531 treatment conditions.All images are provided in their original acquisition format as generated by the microscope, without post-processing.Folder Structure: Images are organized by treatment condition and biological replicate.Each subfolder contains 7–11 raw .czi image files, corresponding to individual imaging fields or timepoints.Note:The condition “Images 5uM drug 2” is missing because the corresponding spheroid was not available for imaging.Technical Details: File format: .czi (Zeiss proprietary format) can be opened using Fiji.
