# Supplementary material for: CDK12/CDK13 inhibition disrupts transcriptional elongation and replication fork progression in glioblastoma
Source: EMBO Mol Med. 2026 Mar 25;18(5):1592–624. doi: 10.1038/s44321-026-00393-w (PMC13179391; doi:10.1038/s44321-026-00393-w)
Supplement: Supplementary file 10 — Source data Fig. 3 [file 44321_2026_393_MOESM10_ESM.zip › Figure 3/3C/Readme.rtf]

README – Figure 3C (Quantification of Invasion Distance)File: Source data migration analysis RFP cells.xlsxDescription: This Excel file contains the quantitative invasion distance measurements used to generate Figure 3C, assessing the invasion of H2B-mCherry (RFP)–expressing G7 spheroids into murine organotypic brain slices under control (DMSO) and THZ531-treated conditions.The file includes:Individual invasion distance measurements per spheroid/imageGrouped by treatment condition (control vs THZ531)Values used to calculate mean ± SEM, as shown in the figureEach data point corresponds to a single spheroid or imaging field derived from the raw .czi images provided in Figure 3B.
