# Supplementary material for: CDK12/CDK13 inhibition disrupts transcriptional elongation and replication fork progression in glioblastoma
Source: EMBO Mol Med. 2026 Mar 25;18(5):1592–624. doi: 10.1038/s44321-026-00393-w (PMC13179391; doi:10.1038/s44321-026-00393-w)
Supplement: Supplementary file 10 — Source data Fig. 3 [file 44321_2026_393_MOESM10_ESM.zip › Figure 3/3D/Readme.rtf]

README – Figure 3D (SR-4835 Dose–Response in U87-MG Cells)File: 3D_U-87_SR-4835_DRC.csvDescription: This file contains the raw dose–response viability data used to generate Figure 3D, showing the response of U87-MG glioma cells to increasing concentrations of SR-4835.
