# Supplementary material for: CDK12/CDK13 inhibition disrupts transcriptional elongation and replication fork progression in glioblastoma
Source: EMBO Mol Med. 2026 Mar 25;18(5):1592–624. doi: 10.1038/s44321-026-00393-w (PMC13179391; doi:10.1038/s44321-026-00393-w)
Supplement: Supplementary file 10 — Source data Fig. 3 [file 44321_2026_393_MOESM10_ESM.zip › Figure 3/3A/Readme.rtf]

README – Figure 3A (Glioblastoma Cell Migration Speed)File: 3A_migration_average_std_exp_180219.xlsxDescription:This file contains the quantified migration speed data used to generate the boxplot shown in Figure 3A.Migration speeds were measured for four GSCs under the indicated treatments. The EGFR inhibitor Gefitinib was included as a positive control.Data Structure: The Excel file contains:Average migration speed values per conditionStandard deviation (SD) valuesData derived from 4–6 independent time-lapse movies per conditionEach data point represents the mean migration speed from one movie, and the boxplot summarizes these averages.
