# Supplementary material for: CDK12/CDK13 inhibition disrupts transcriptional elongation and replication fork progression in glioblastoma
Source: EMBO Mol Med. 2026 Mar 25;18(5):1592–624. doi: 10.1038/s44321-026-00393-w (PMC13179391; doi:10.1038/s44321-026-00393-w)
Supplement: Supplementary file 10 — Source data Fig. 3 [file 44321_2026_393_MOESM10_ESM.zip › Figure 3/3F/Readme.rtf]

README – Figure 3F (In Vivo Tumor Volume and Body Weight)Files included: 3F_Top_panel_Tumour_Volume.csv, 3F_bottom_panel_Relative_BW.csvDescription: These files contain the in vivo longitudinal data used to generate Figure 3F, showing the effects of the indicated treatments on tumor growth and mouse body weight over time. The top panel displays tumor volume, while the bottom panel shows relative body weight, serving as a measure of treatment tolerability.
