# Supplementary material for: CDK12/CDK13 inhibition disrupts transcriptional elongation and replication fork progression in glioblastoma
Source: EMBO Mol Med. 2026 Mar 25;18(5):1592–624. doi: 10.1038/s44321-026-00393-w (PMC13179391; doi:10.1038/s44321-026-00393-w)
Supplement: Supplementary file 10 — Source data Fig. 3 [file 44321_2026_393_MOESM10_ESM.zip › Figure 3/3G/Readme.rtf]

README – Figure 3G (IC50 Values for cancer cell lines)File included: SR-4835 IC50.csvDescription: This CSV file contains the SR-4835 IC50 values (µM) for cancer cell lines from pancreatic, ovarian, uterine, prostate and GBM cancers. The cell line information is provided in Dataset_EV3.
