# Supplementary material for: CDK12/CDK13 inhibition disrupts transcriptional elongation and replication fork progression in glioblastoma
Source: EMBO Mol Med. 2026 Mar 25;18(5):1592–624. doi: 10.1038/s44321-026-00393-w (PMC13179391; doi:10.1038/s44321-026-00393-w)
Supplement: Supplementary file 11 — Source data Fig. 4 [file 44321_2026_393_MOESM11_ESM.zip › Figure 4/4A/4A_uncropped_western_blots.pptx]

## Slide 1
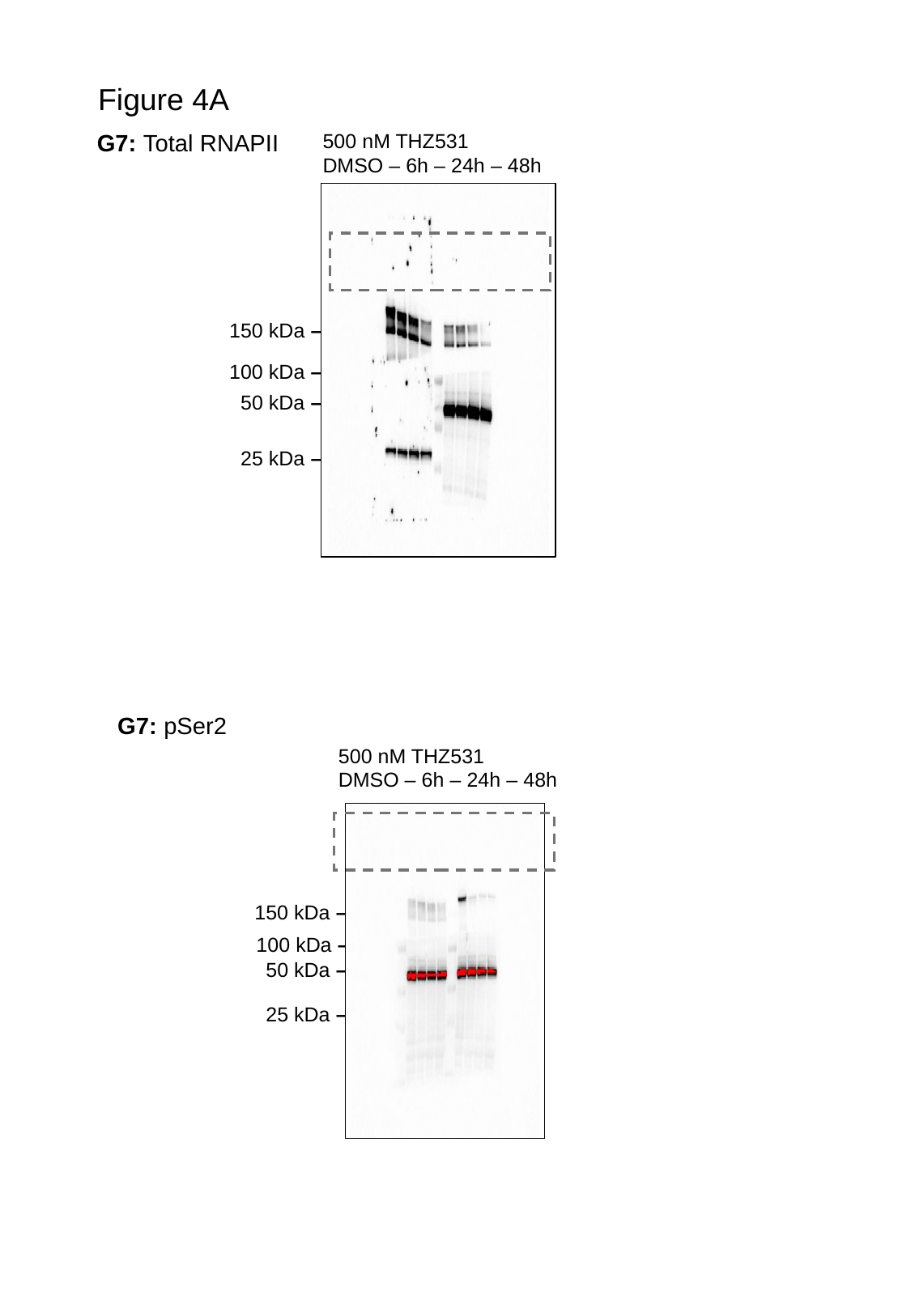

Figure 4A
G7: Total RNAPII
500 nM THZ531
DMSO – 6h – 24h – 48h
150 kDa –
100 kDa –
50 kDa –
25 kDa –
G7: pSer2
500 nM THZ531
DMSO – 6h – 24h – 48h
150 kDa –
100 kDa –
50 kDa –
25 kDa –

## Slide 2
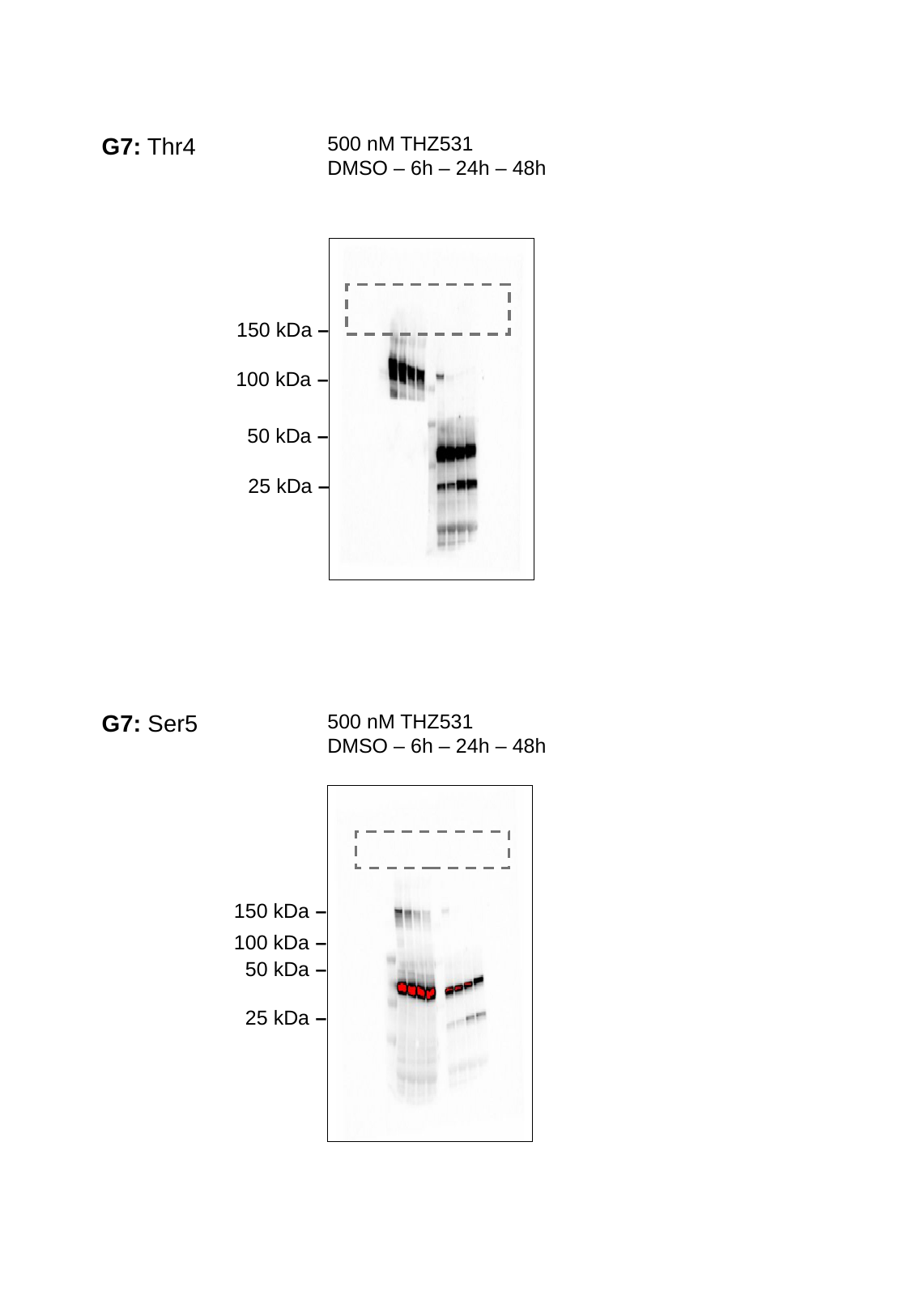

G7: Thr4
500 nM THZ531
DMSO – 6h – 24h – 48h
150 kDa –
100 kDa –
50 kDa –
25 kDa –
G7: Ser5
500 nM THZ531
DMSO – 6h – 24h – 48h
150 kDa –
100 kDa –
50 kDa –
25 kDa –

## Slide 3
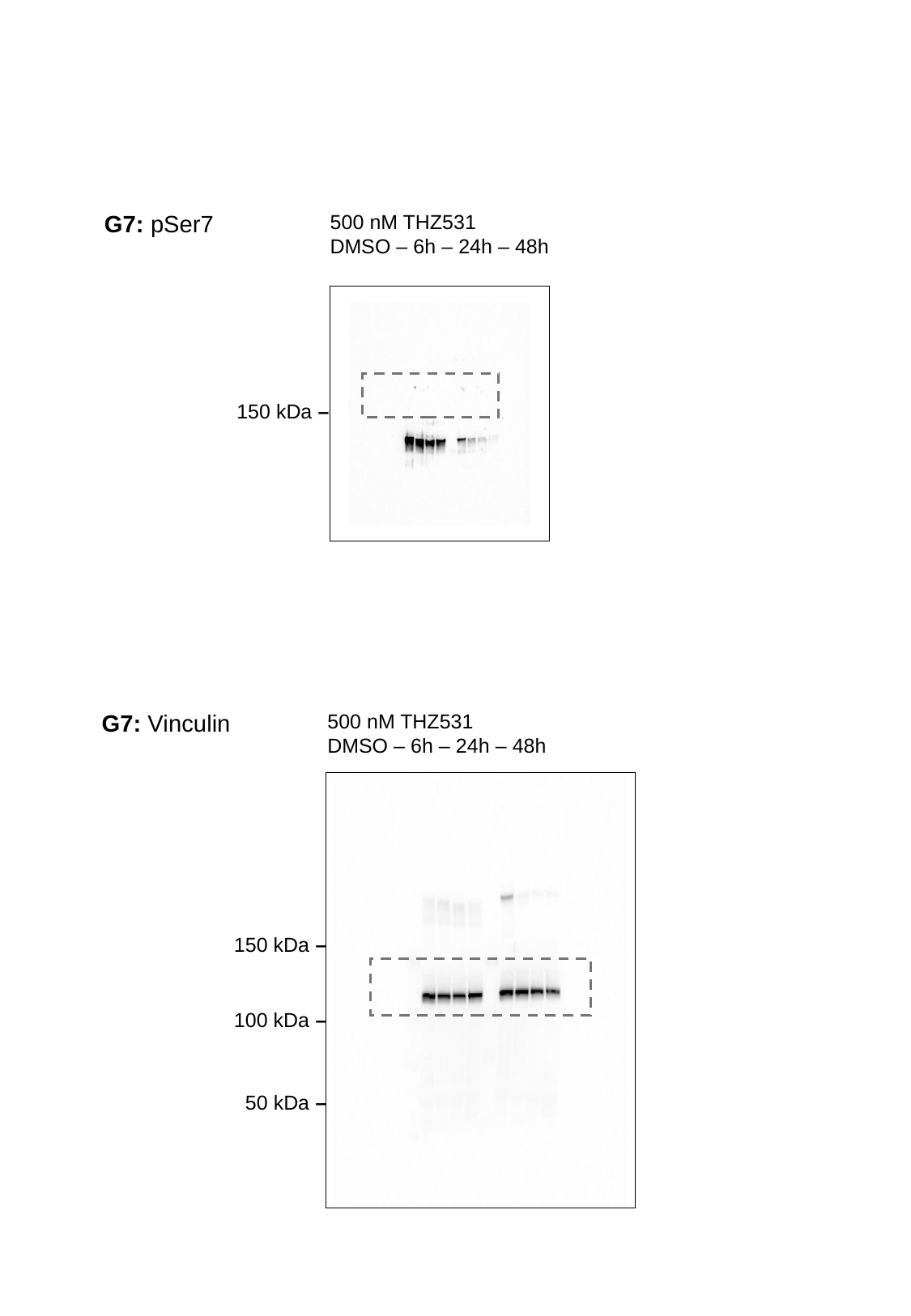

G7: pSer7
500 nM THZ531
DMSO – 6h – 24h – 48h
150 kDa –
G7: Vinculin
500 nM THZ531
DMSO – 6h – 24h – 48h
150 kDa –
100 kDa –
50 kDa –

## Slide 4
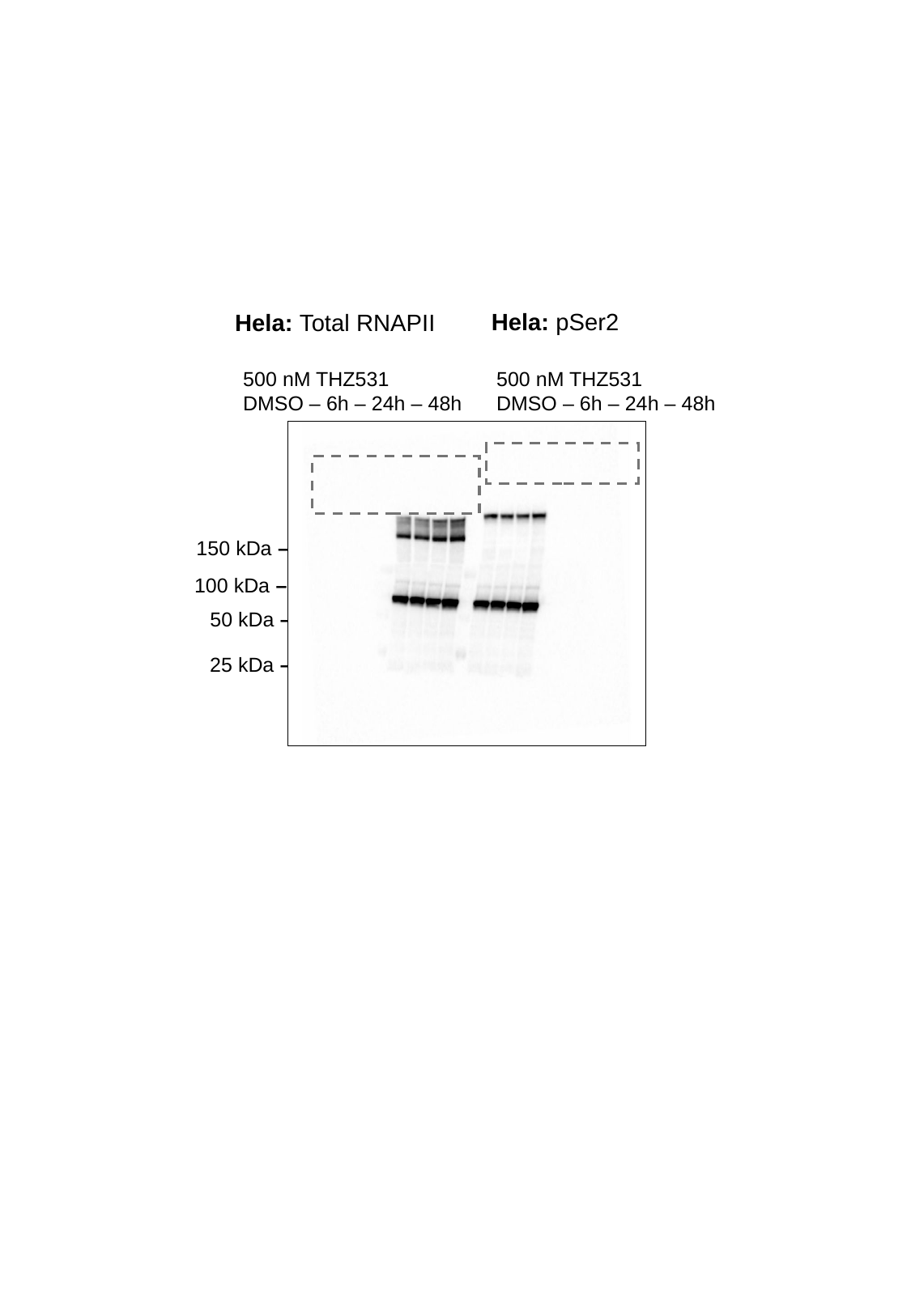

Hela: pSer2
Hela: Total RNAPII
500 nM THZ531
DMSO – 6h – 24h – 48h
500 nM THZ531
DMSO – 6h – 24h – 48h
150 kDa –
100 kDa –
50 kDa –
25 kDa –

## Slide 5
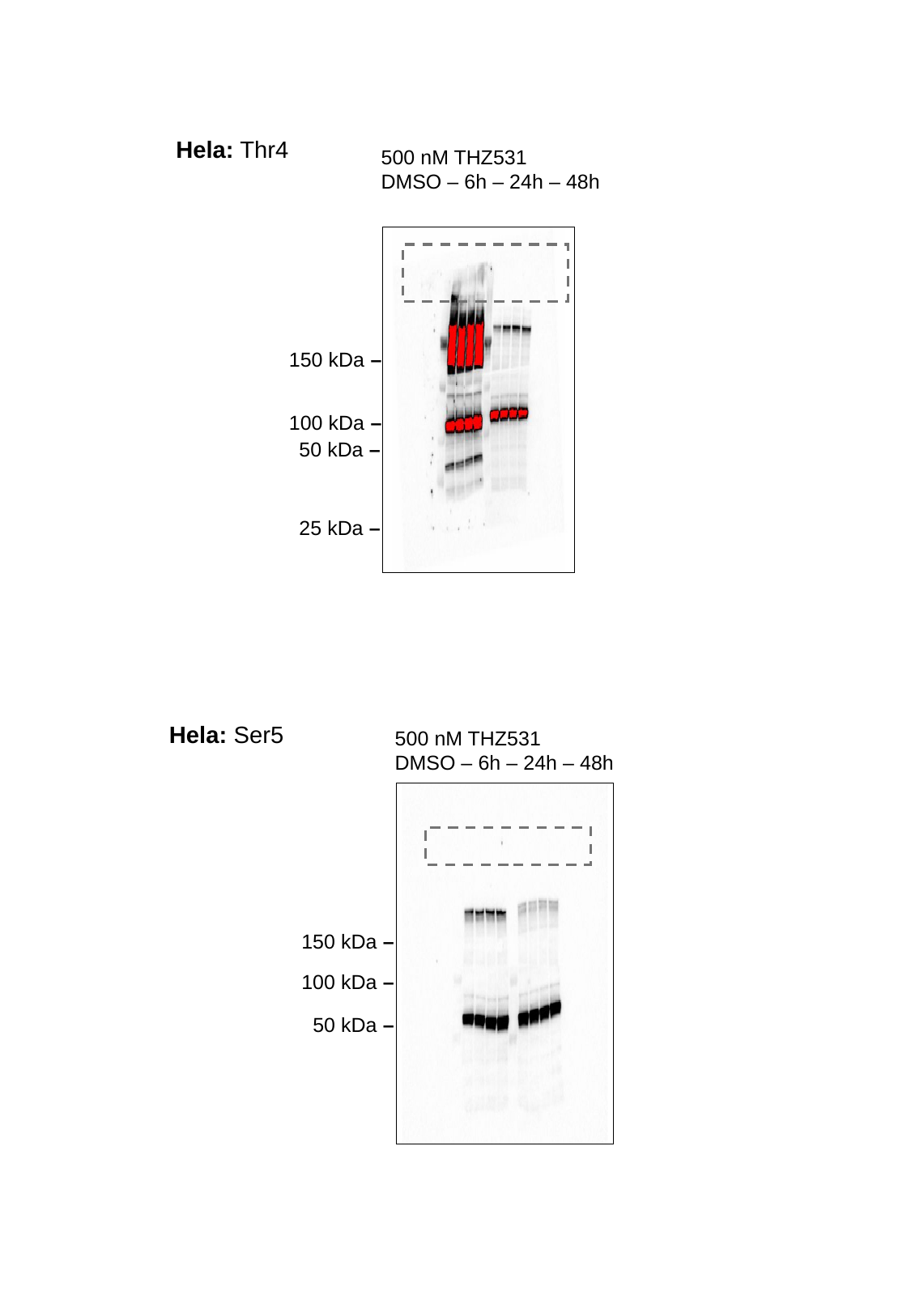

Hela: Thr4
500 nM THZ531
DMSO – 6h – 24h – 48h
150 kDa –
100 kDa –
50 kDa –
25 kDa –
Hela: Ser5
500 nM THZ531
DMSO – 6h – 24h – 48h
150 kDa –
100 kDa –
50 kDa –

## Slide 6
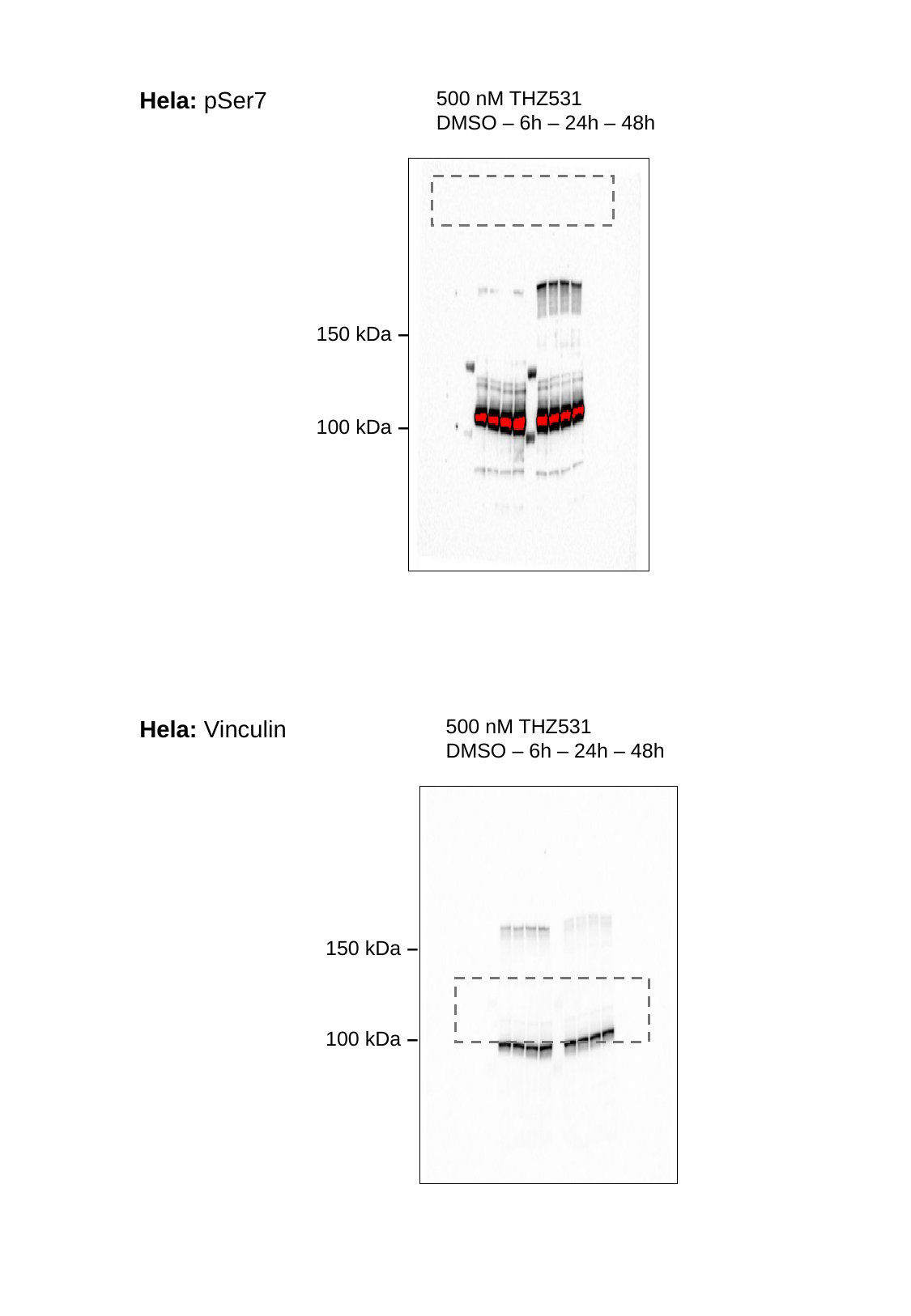

Hela: pSer7
500 nM THZ531
DMSO – 6h – 24h – 48h
150 kDa –
100 kDa –
Hela: Vinculin
500 nM THZ531
DMSO – 6h – 24h – 48h
150 kDa –
100 kDa –
