# Supplementary material for: CDK12/CDK13 inhibition disrupts transcriptional elongation and replication fork progression in glioblastoma
Source: EMBO Mol Med. 2026 Mar 25;18(5):1592–624. doi: 10.1038/s44321-026-00393-w (PMC13179391; doi:10.1038/s44321-026-00393-w)
Supplement: Supplementary file 11 — Source data Fig. 4 [file 44321_2026_393_MOESM11_ESM.zip › Figure 4/4A/Readme.rtf]

README – Figure 4A (western blot images of RNAPII species)Files included: 4A_uncropped_western_blots.docx; 4A_uncropped_western_blots.pptx; PolII_westernblots.docxDescription: 4A_uncropped_western_blots.docx containes unannotated and uncropped western blots in the order presented in figure 4A. 4A_uncropped_western_blots.pptx contains the same western blot images, annotated with kDa and protein probed for. PolII_westernblots.docx contains the cropped western blots as presented in figure 4A
