# Supplementary material for: CDK12/CDK13 inhibition disrupts transcriptional elongation and replication fork progression in glioblastoma
Source: EMBO Mol Med. 2026 Mar 25;18(5):1592–624. doi: 10.1038/s44321-026-00393-w (PMC13179391; doi:10.1038/s44321-026-00393-w)
Supplement: Supplementary file 12 — Source data Fig. 5 [file 44321_2026_393_MOESM12_ESM.zip › Figure 5/5A/Readme.rtf]

README – Figure 5A (Cell Cycle Analysis by EdU and DNA Content)Files included: export_G7 par Deo_6h DMSO II_006_Interphase.fcs, export_G7 par Deo_6h 500nM I_007_Interphase.fcs, export_G7 par Deo_24h DMSO I_025_Interphase.fcs, export_G7 par Deo_24h 500nM II_028_Interphase.fcs, export_G144_6h DMSO II_012_M-.fcs, export_G144_6h 500nM I_013_M-.fcs, export_G144_24h DMSO II_020_M-.fcs, export_G144_24h 500nM I_021_M-.fcsDescription: This folder contains the raw flow cytometry data (FCS format) used to generate Figure 5A, showing cell cycle profiles of GSCs, G7 and G144 following CDK12/CDK13 inhibition.Cells were treated with DMSO or 500 nM THZ531 for 6 h or 24 h. 
