# Supplementary material for: CDK12/CDK13 inhibition disrupts transcriptional elongation and replication fork progression in glioblastoma
Source: EMBO Mol Med. 2026 Mar 25;18(5):1592–624. doi: 10.1038/s44321-026-00393-w (PMC13179391; doi:10.1038/s44321-026-00393-w)
Supplement: Supplementary file 12 — Source data Fig. 5 [file 44321_2026_393_MOESM12_ESM.zip › Figure 5/5E/Readme.rtf]

Schematic diagram
