# Supplementary material for: CDK12/CDK13 inhibition disrupts transcriptional elongation and replication fork progression in glioblastoma
Source: EMBO Mol Med. 2026 Mar 25;18(5):1592–624. doi: 10.1038/s44321-026-00393-w (PMC13179391; doi:10.1038/s44321-026-00393-w)
Supplement: Supplementary file 12 — Source data Fig. 5 [file 44321_2026_393_MOESM12_ESM.zip › Figure 5/5B/Readme.rtf]

README – Figure 5B (Cell Cycle Distribution Summary)Files included: G7 par Deo_6h DMSO I_005.fcs, G7 par Deo_6h DMSO II_006.fcs, G7 par Deo_6h 500nM I_007.fcs, G7 par Deo_6h 500nM II_008.fcs, G7 par Deo_24h DMSO I_025.fcs, G7 par Deo_24h DMSO II_026.fcs, G7 par Deo_24h 500nM I_027.fcs, G7 par Deo_24h 500nM II_028.fcsDescription: This folder contains the raw flow cytometry data (FCS format) used to quantify cell cycle phase distributions shown in Figure 5B.
