# Supplementary material for: CDK12/CDK13 inhibition disrupts transcriptional elongation and replication fork progression in glioblastoma
Source: EMBO Mol Med. 2026 Mar 25;18(5):1592–624. doi: 10.1038/s44321-026-00393-w (PMC13179391; doi:10.1038/s44321-026-00393-w)
Supplement: Supplementary file 13 — Source data Fig. 6 [file 44321_2026_393_MOESM13_ESM.zip › Figure 6/6B/Readme.rtf]

Readme – Figure 6BFolder: 220915 Comet assay alkaline/This folder contains the tif-files from 2 repeats of alkaline comet assay for conditions: DMSO, epoposide, NVP2 6h, and THZ531 6h, organised in their respective folders. 
