# Supplementary material for: CDK12/CDK13 inhibition disrupts transcriptional elongation and replication fork progression in glioblastoma
Source: EMBO Mol Med. 2026 Mar 25;18(5):1592–624. doi: 10.1038/s44321-026-00393-w (PMC13179391; doi:10.1038/s44321-026-00393-w)
Supplement: Supplementary file 13 — Source data Fig. 6 [file 44321_2026_393_MOESM13_ESM.zip › Figure 6/6E/Readme.rtf]

README – Figure 6E (DNA Fiber Assay: Replication Fork Speed)File: Fiber_Ctrl_THZ_NVP.csvDescription: This file contains the replication fork speed measurements used to generate Figure 6E, based on DNA fiber assays performed in GSCs G7. Cells were treated with:Vehicle (control)THZ531 (500 nM, 6 h)NVP-2 (100 nM, 6 h)Replication dynamics were assessed by sequential incorporation of CldU and IdU thymidine analogs.
