# Supplementary material for: CDK12/CDK13 inhibition disrupts transcriptional elongation and replication fork progression in glioblastoma
Source: EMBO Mol Med. 2026 Mar 25;18(5):1592–624. doi: 10.1038/s44321-026-00393-w (PMC13179391; doi:10.1038/s44321-026-00393-w)
Supplement: Supplementary file 13 — Source data Fig. 6 [file 44321_2026_393_MOESM13_ESM.zip › Figure 6/6D/Readme.rtf]

README – Figure 6D Description: 6D shows a representative figure illustrating the DNA fiber assay workflow used in this study, including sequential labeling with CldU and IdU following the indicated treatments.All quantitative measurements derived from DNA fiber assays (replication fork speed) are provided in the Figure 6E source data.
