# Supplementary material for: CDK12/CDK13 inhibition disrupts transcriptional elongation and replication fork progression in glioblastoma
Source: EMBO Mol Med. 2026 Mar 25;18(5):1592–624. doi: 10.1038/s44321-026-00393-w (PMC13179391; doi:10.1038/s44321-026-00393-w)
Supplement: Supplementary file 13 — Source data Fig. 6 [file 44321_2026_393_MOESM13_ESM.zip › Figure 6/6C/Readme.rtf]

README – Figure 6C (Quantification of Alkaline Comet Assay)File: 6C_Alkaline_Comet_assay.csvDescription: This file contains the quantitative analysis of DNA damage used to generate Figure 6C, based on the alkaline comet assay performed in G7 glioblastoma stem cells.DNA damage is reported as tail moment / % DNA in tail, derived from the raw comet images provided in Figure 6B.The CSV file includes:DMSO (control)THZ531 (500 nM, 6 h)NVP-2 (50 nM, 6 h)Etoposide (20 µM, positive control)
