# Supplementary figures and images for: CDK12/CDK13 inhibition disrupts transcriptional elongation and replication fork progression in glioblastoma (part 2 of 2)
Source: EMBO Mol Med. 2026 Mar 25;18(5):1592–624. doi: 10.1038/s44321-026-00393-w (PMC13179391; doi:10.1038/s44321-026-00393-w)

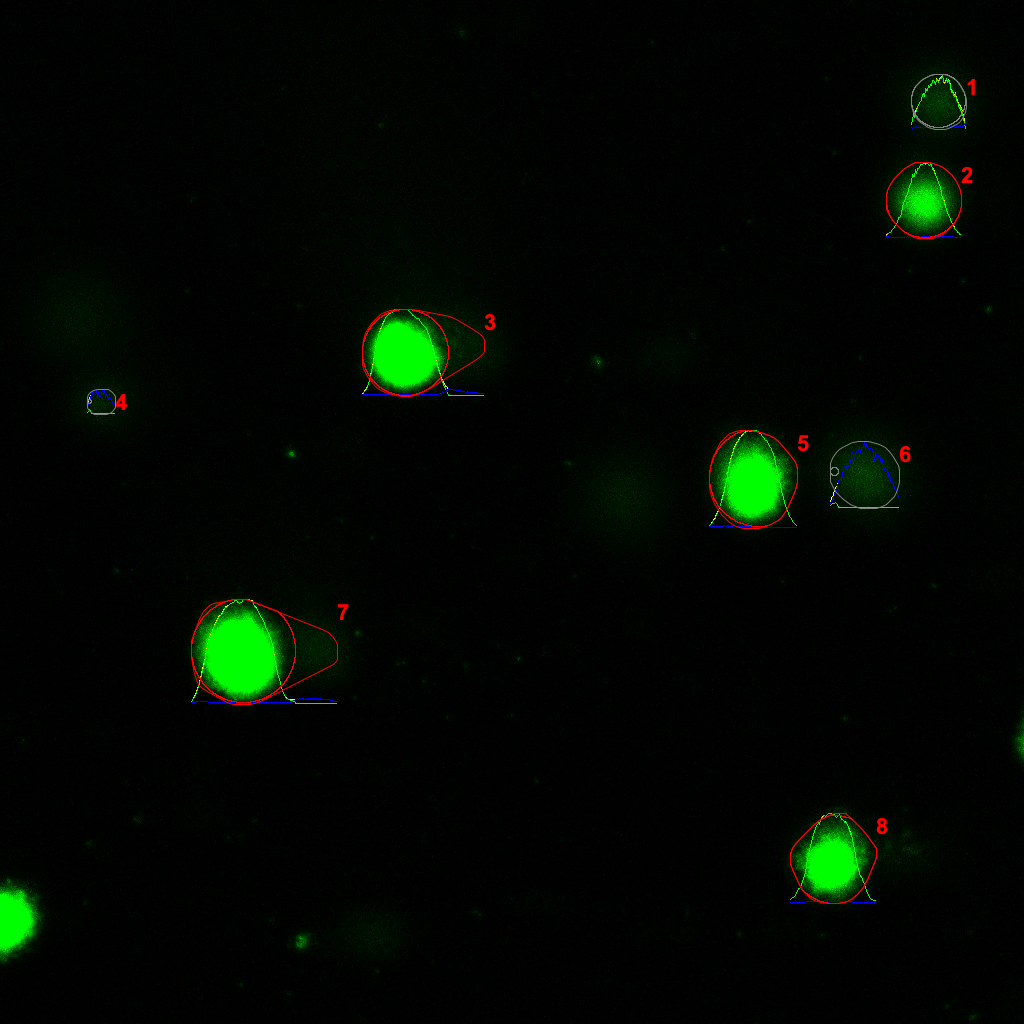

Supplement: Supplementary file 13 — Source data Fig. 6 [file 44321_2026_393_MOESM13_ESM.zip › Figure 6/6B/220915 Comet assay alkaline/output DMSO 2/17_DMSO_6h_10x_Ccenter15_1AUall_rep2_Maximum.ome.tif_out.tif]

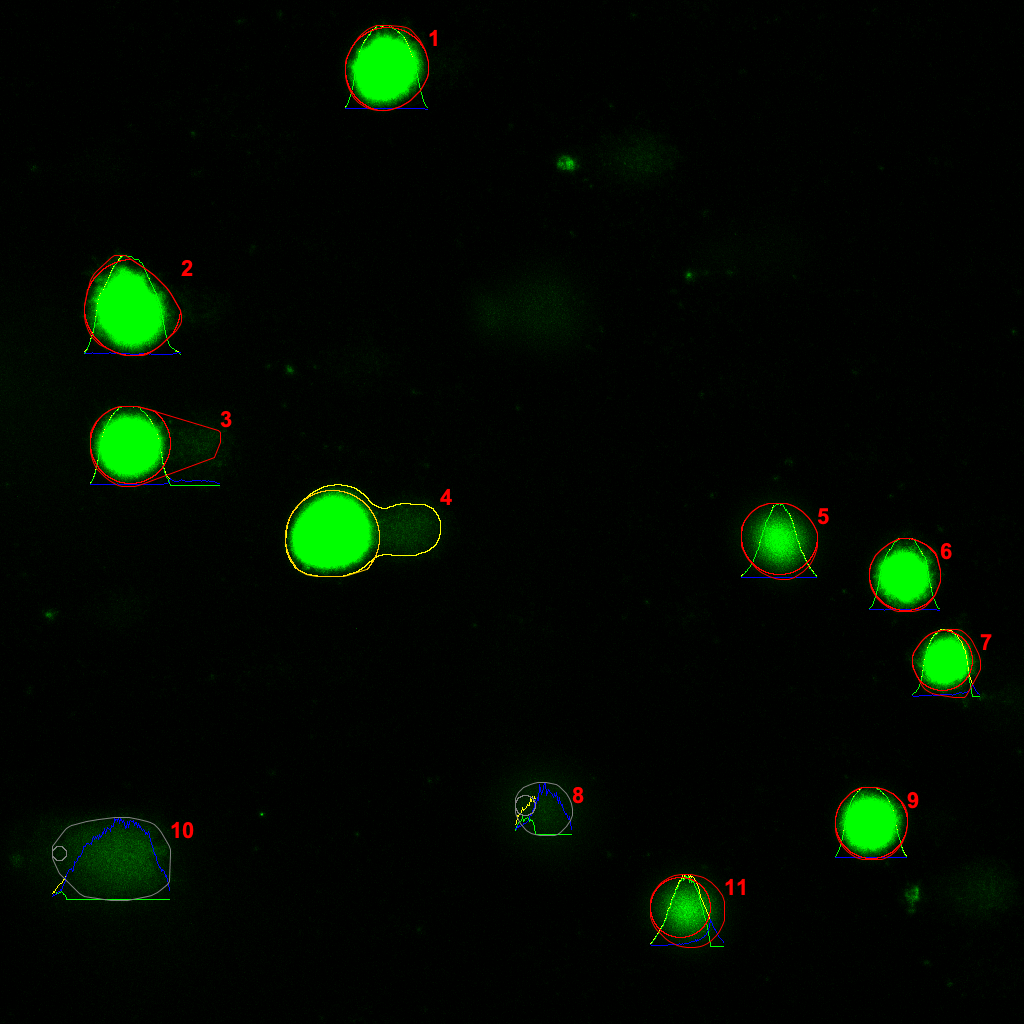

Supplement: Supplementary file 13 — Source data Fig. 6 [file 44321_2026_393_MOESM13_ESM.zip › Figure 6/6B/220915 Comet assay alkaline/output DMSO 2/15_DMSO_6h_10x_Ccenter15_1AUall_rep2_Maximum.ome.tif_out.tif]

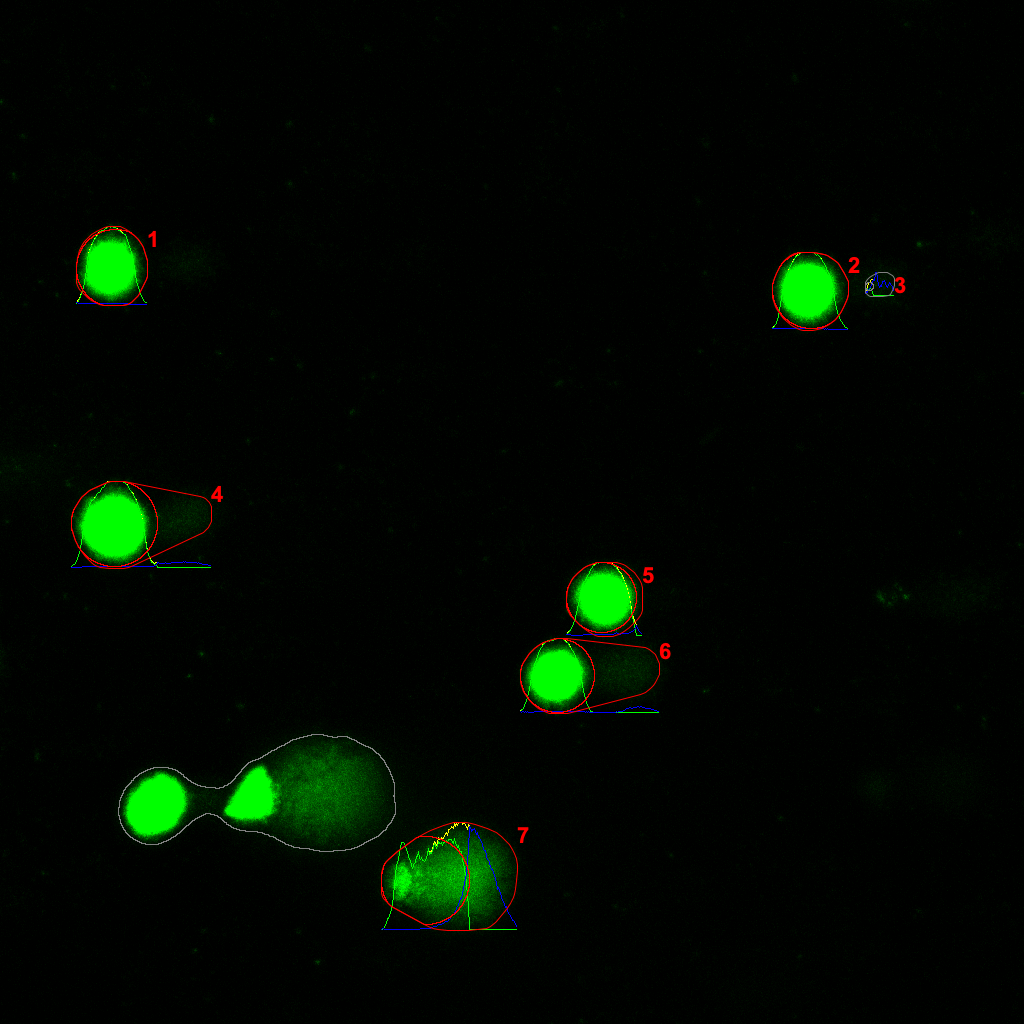

Supplement: Supplementary file 13 — Source data Fig. 6 [file 44321_2026_393_MOESM13_ESM.zip › Figure 6/6B/220915 Comet assay alkaline/output DMSO 2/14_DMSO_6h_10x_Ccenter15_1AUall_rep2_Maximum.ome.tif_out.tif]

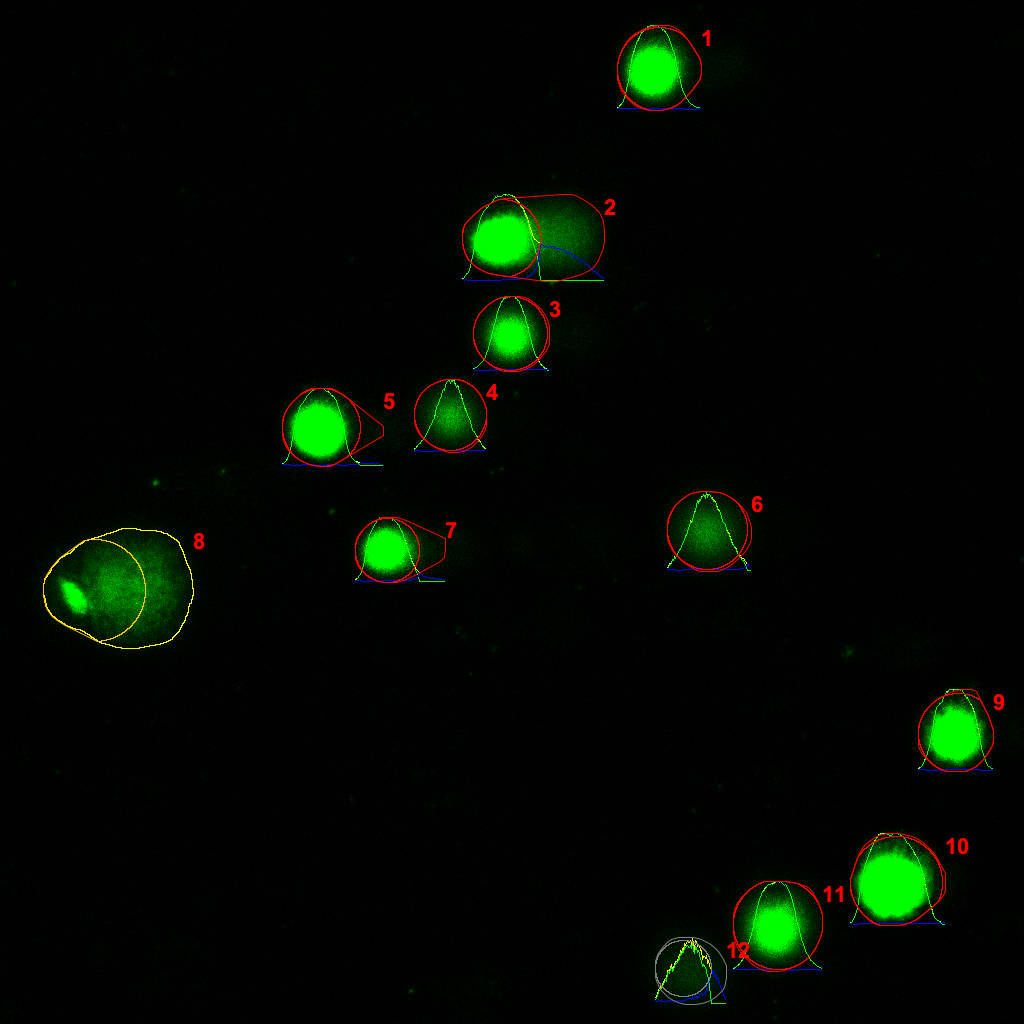

Supplement: Supplementary file 13 — Source data Fig. 6 [file 44321_2026_393_MOESM13_ESM.zip › Figure 6/6B/220915 Comet assay alkaline/output DMSO 2/11_DMSO_6h_10x_Ccenter15_1AUall_rep2_Maximum.ome.tif_out.tif]

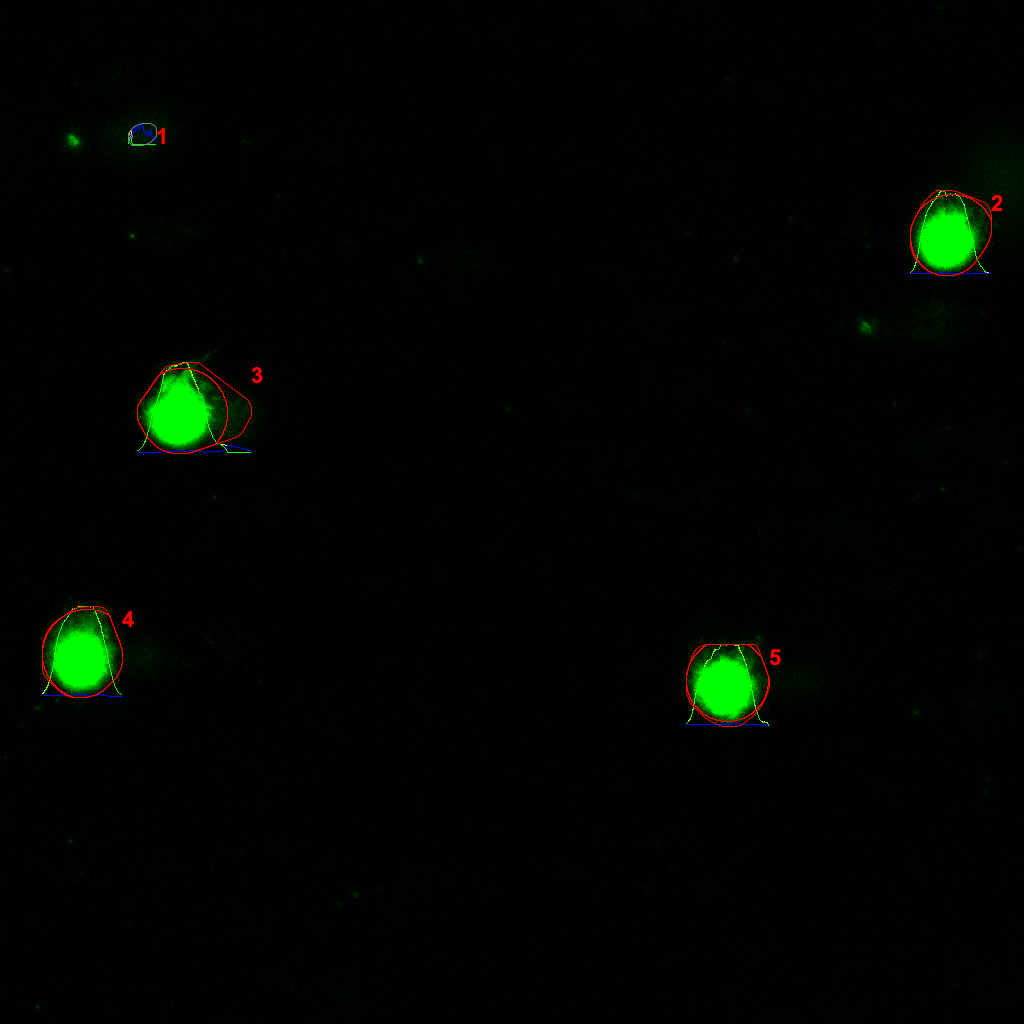

Supplement: Supplementary file 13 — Source data Fig. 6 [file 44321_2026_393_MOESM13_ESM.zip › Figure 6/6B/220915 Comet assay alkaline/output DMSO 2/20_DMSO_6h_10x_Ccenter15_1AUall_rep2_Maximum.ome.tif_out.tif]

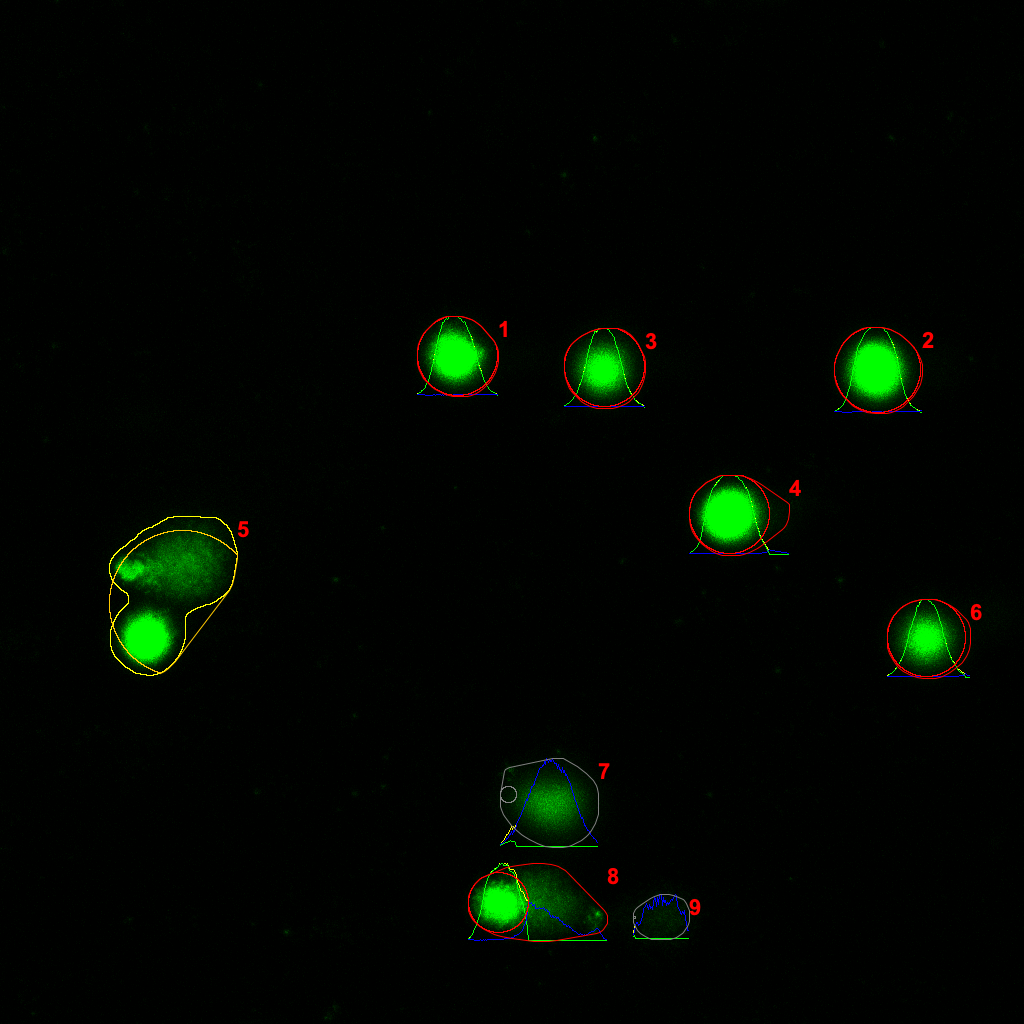

Supplement: Supplementary file 13 — Source data Fig. 6 [file 44321_2026_393_MOESM13_ESM.zip › Figure 6/6B/220915 Comet assay alkaline/output DMSO 2/12_DMSO_6h_10x_Ccenter15_1AUall_rep2_Maximum.ome.tif_out.tif]

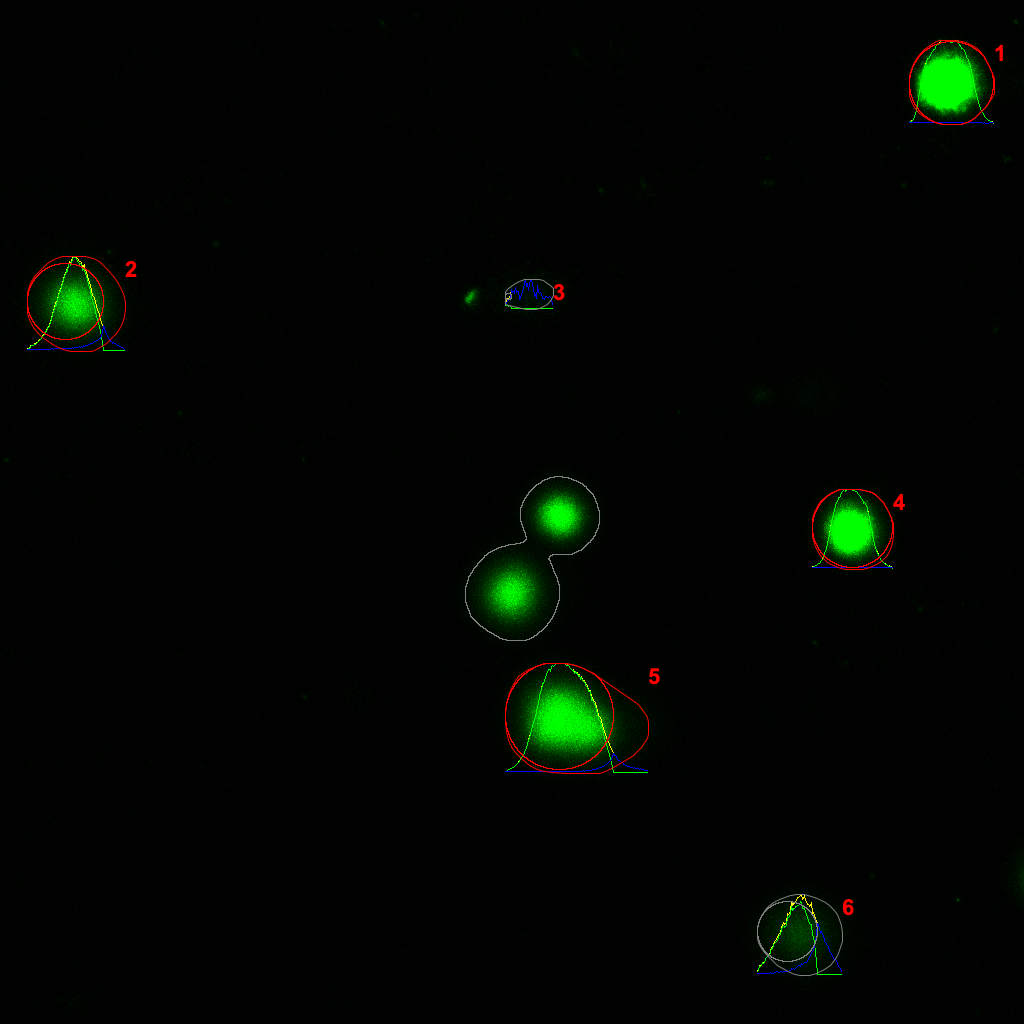

Supplement: Supplementary file 13 — Source data Fig. 6 [file 44321_2026_393_MOESM13_ESM.zip › Figure 6/6B/220915 Comet assay alkaline/output DMSO 2/13_DMSO_6h_10x_Ccenter15_1AUall_rep2_Maximum.ome.tif_out.tif]

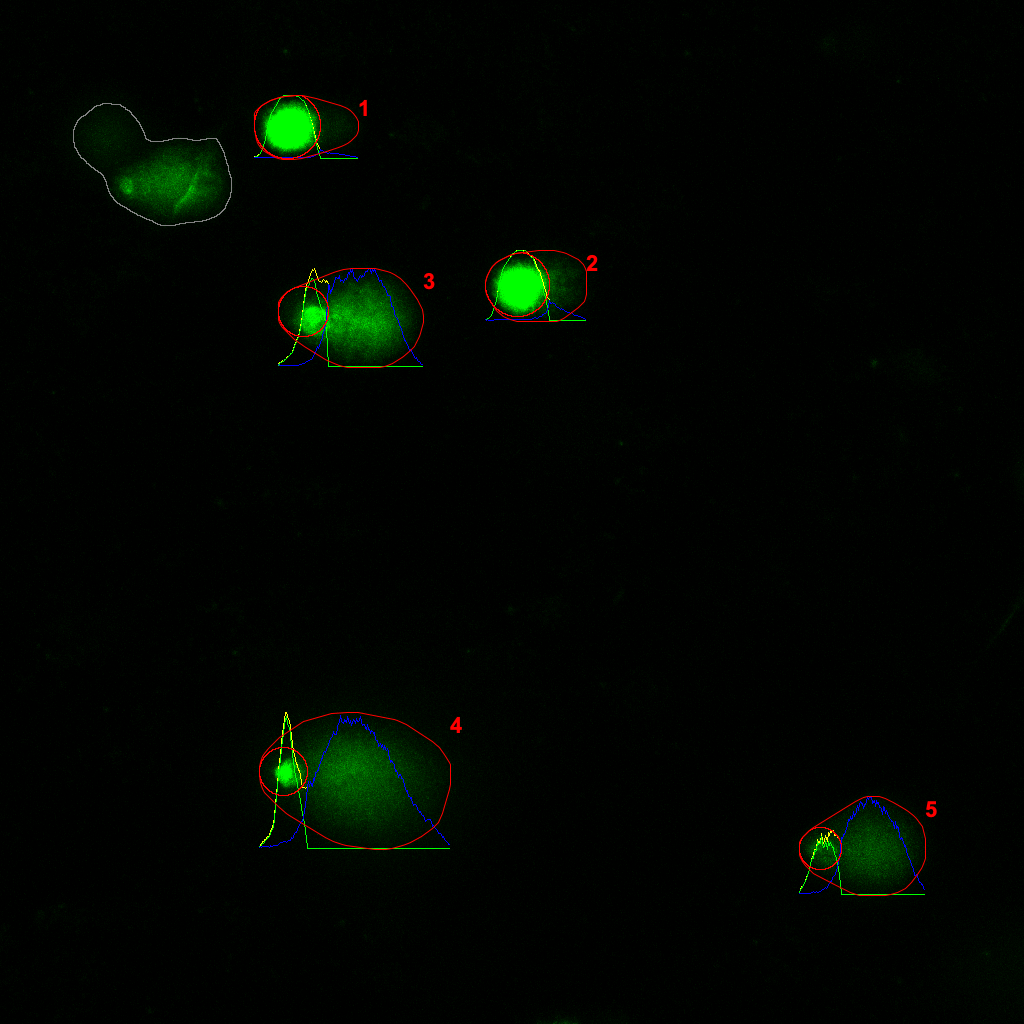

Supplement: Supplementary file 13 — Source data Fig. 6 [file 44321_2026_393_MOESM13_ESM.zip › Figure 6/6B/220915 Comet assay alkaline/output etoposide 2/39_Etoposide_4h_10x_Ccenter15_1AUall_rep2_Maximum.ome.tif_out.tif]

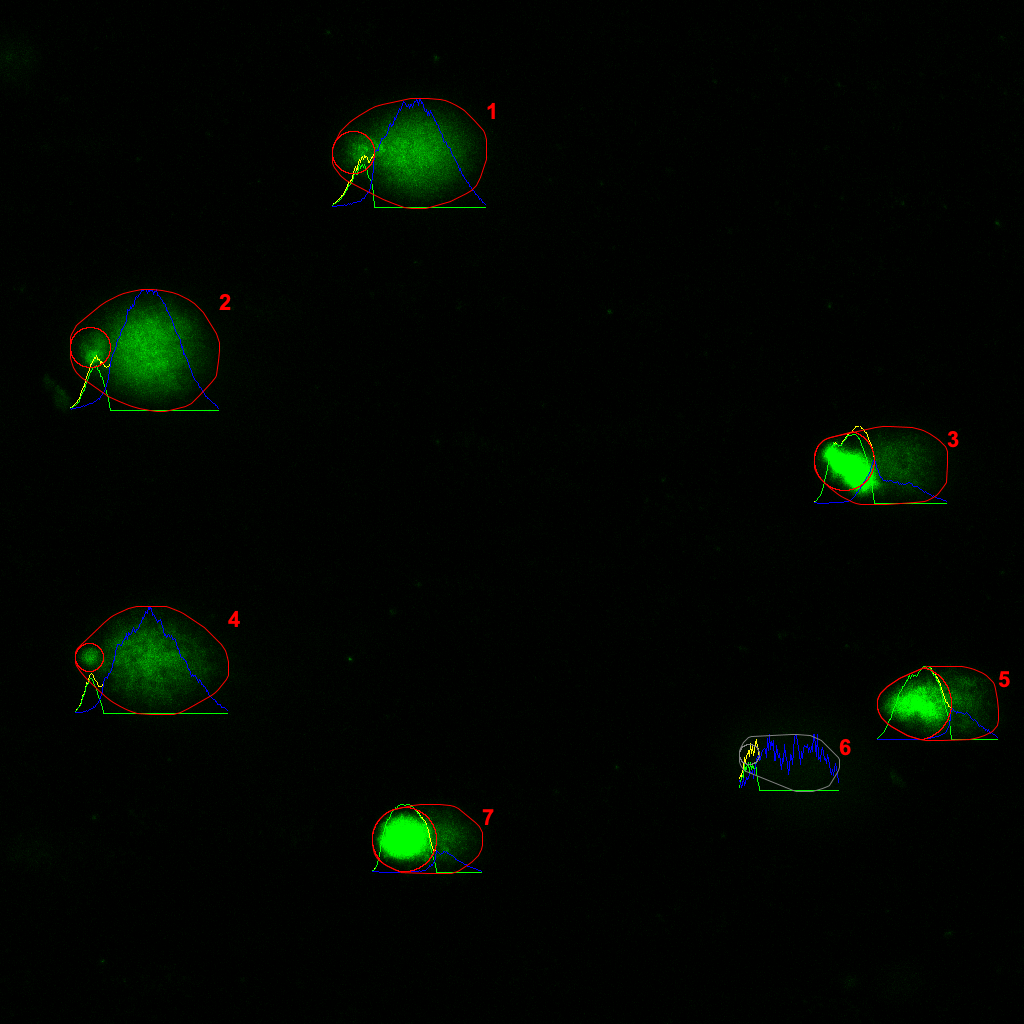

Supplement: Supplementary file 13 — Source data Fig. 6 [file 44321_2026_393_MOESM13_ESM.zip › Figure 6/6B/220915 Comet assay alkaline/output etoposide 2/35_Etoposide_4h_10x_Ccenter15_1AUall_rep2_Maximum.ome.tif_out.tif]

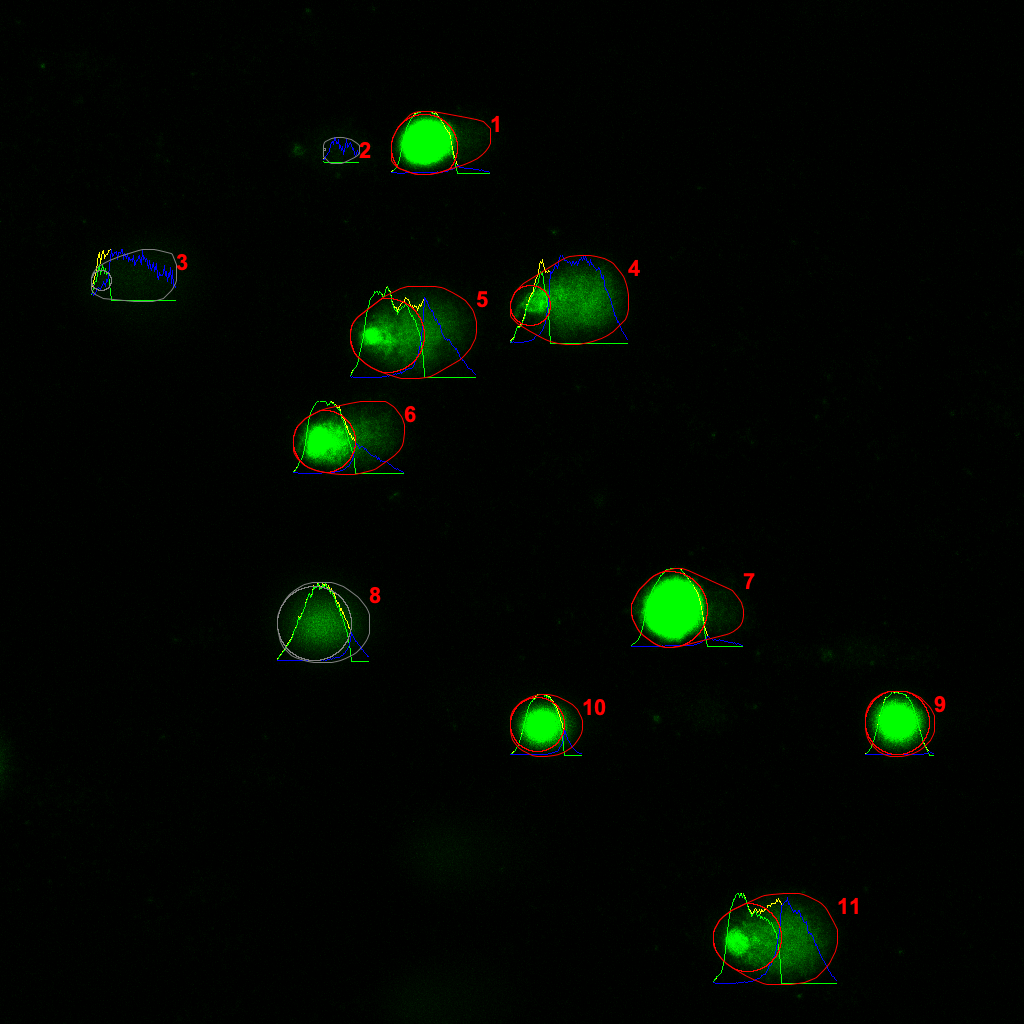

Supplement: Supplementary file 13 — Source data Fig. 6 [file 44321_2026_393_MOESM13_ESM.zip › Figure 6/6B/220915 Comet assay alkaline/output etoposide 2/37_Etoposide_4h_10x_Ccenter15_1AUall_rep2_Maximum.ome.tif_out.tif]

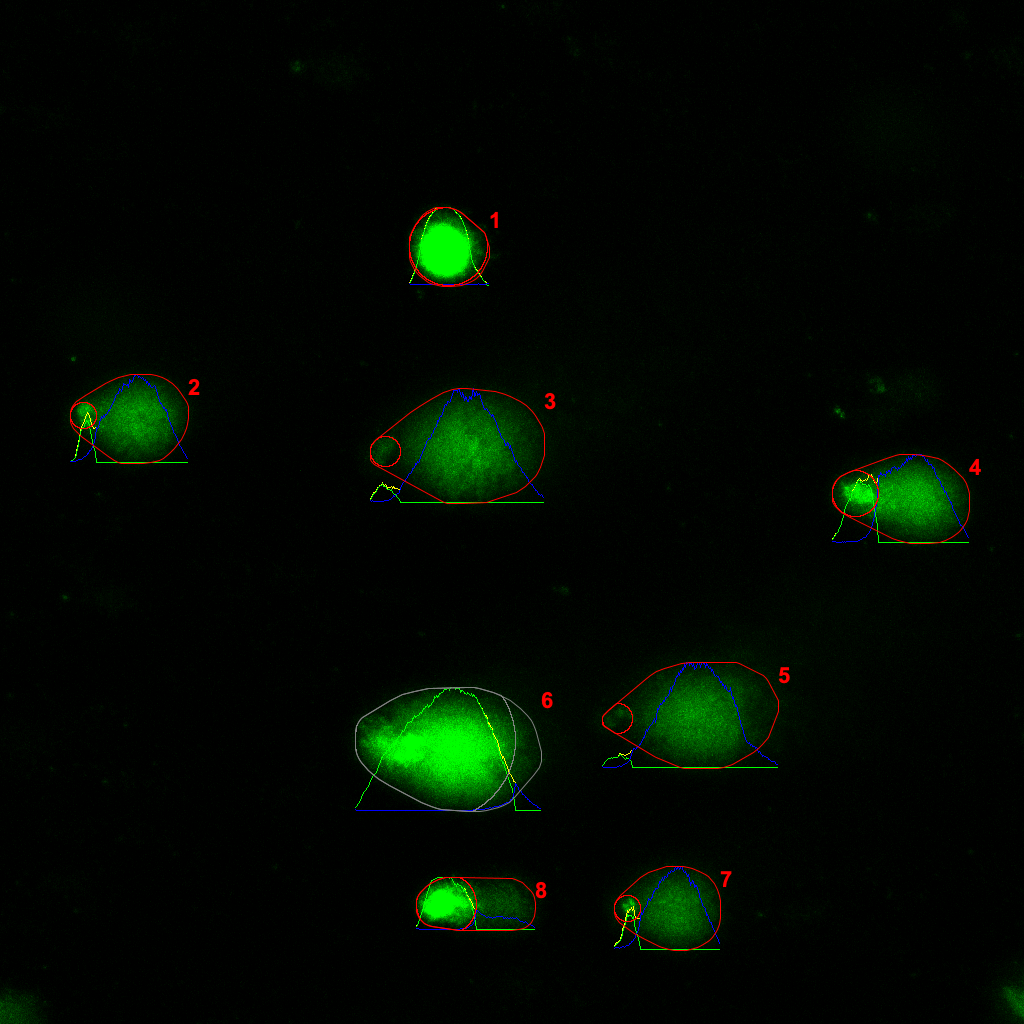

Supplement: Supplementary file 13 — Source data Fig. 6 [file 44321_2026_393_MOESM13_ESM.zip › Figure 6/6B/220915 Comet assay alkaline/output etoposide 2/32_Etoposide_4h_10x_Ccenter15_1AUall_rep2_Maximum.ome.tif_out.tif]

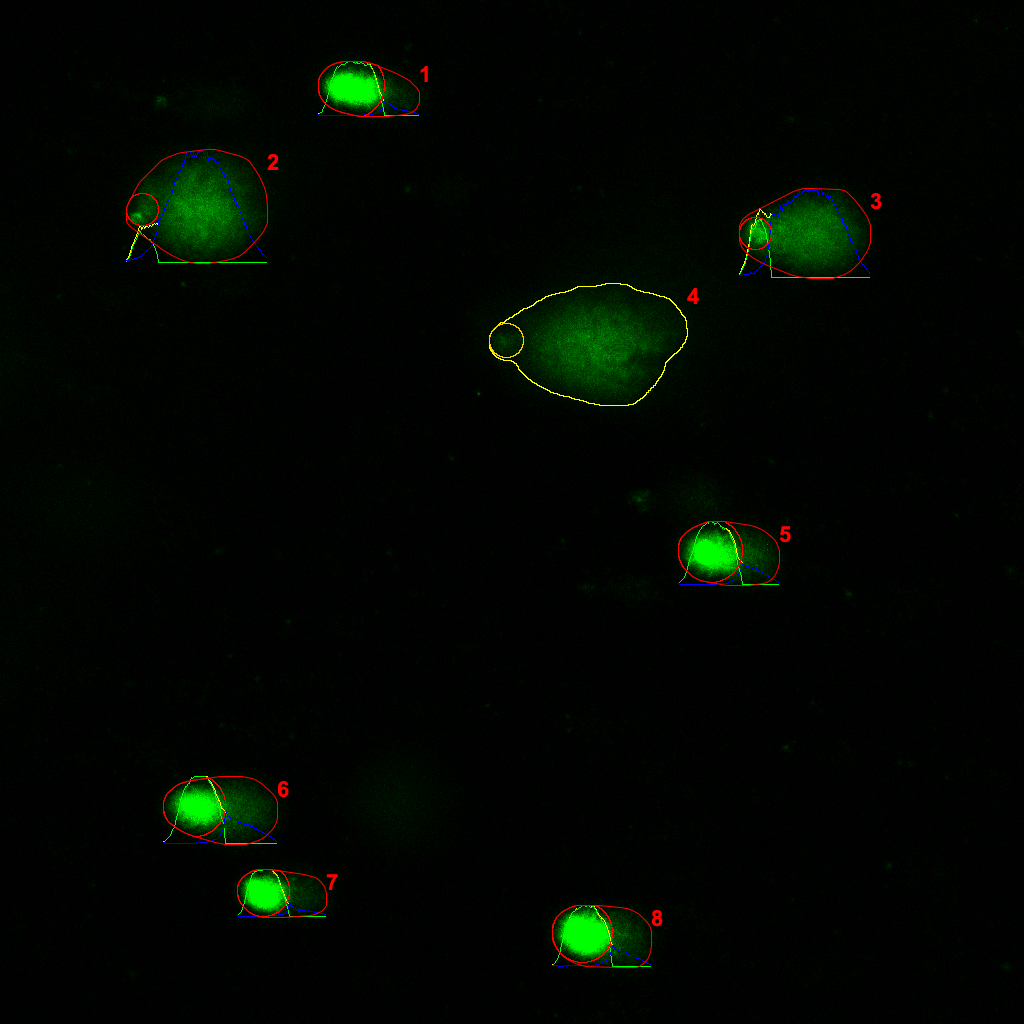

Supplement: Supplementary file 13 — Source data Fig. 6 [file 44321_2026_393_MOESM13_ESM.zip › Figure 6/6B/220915 Comet assay alkaline/output etoposide 2/31_Etoposide_4h_10x_Ccenter15_1AUall_rep2_Maximum.ome.tif_out.tif]

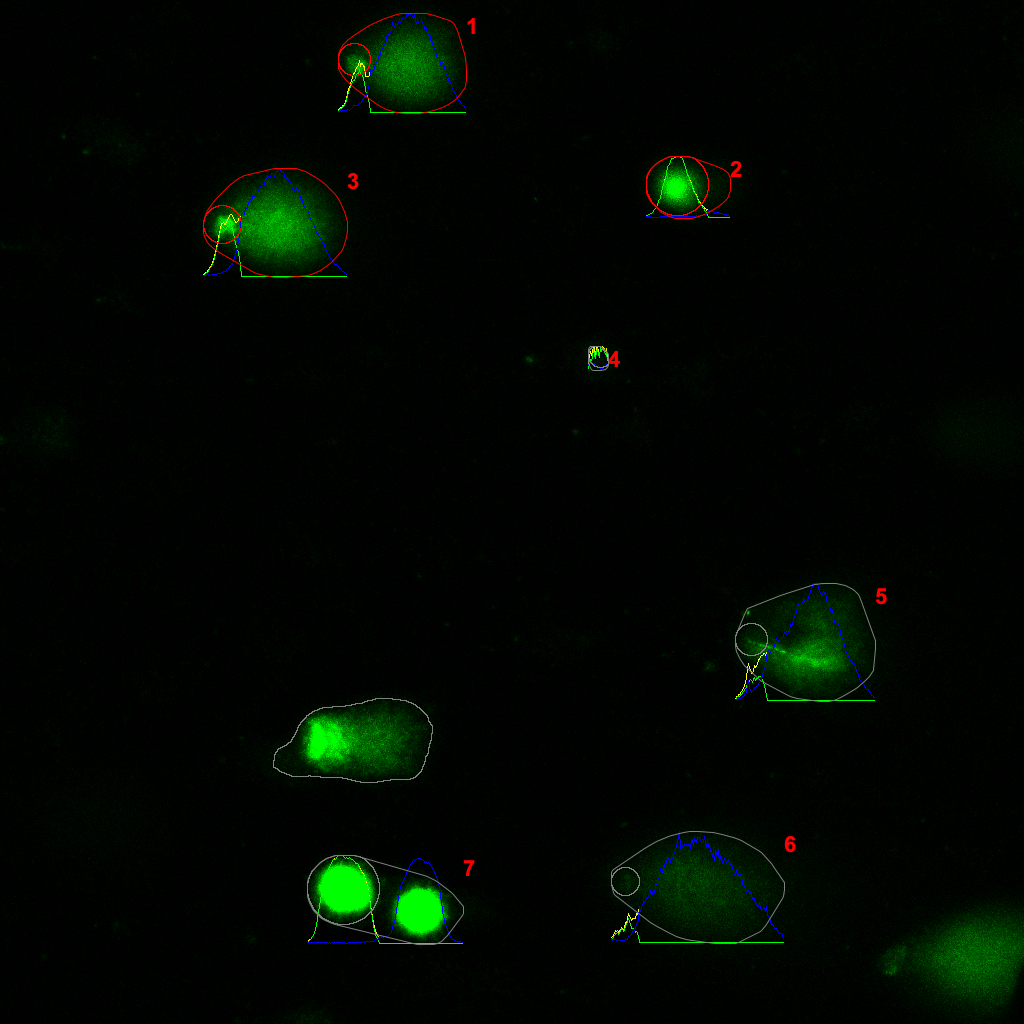

Supplement: Supplementary file 13 — Source data Fig. 6 [file 44321_2026_393_MOESM13_ESM.zip › Figure 6/6B/220915 Comet assay alkaline/output etoposide 2/34_Etoposide_4h_10x_Ccenter15_1AUall_rep2_Maximum.ome.tif_out.tif]

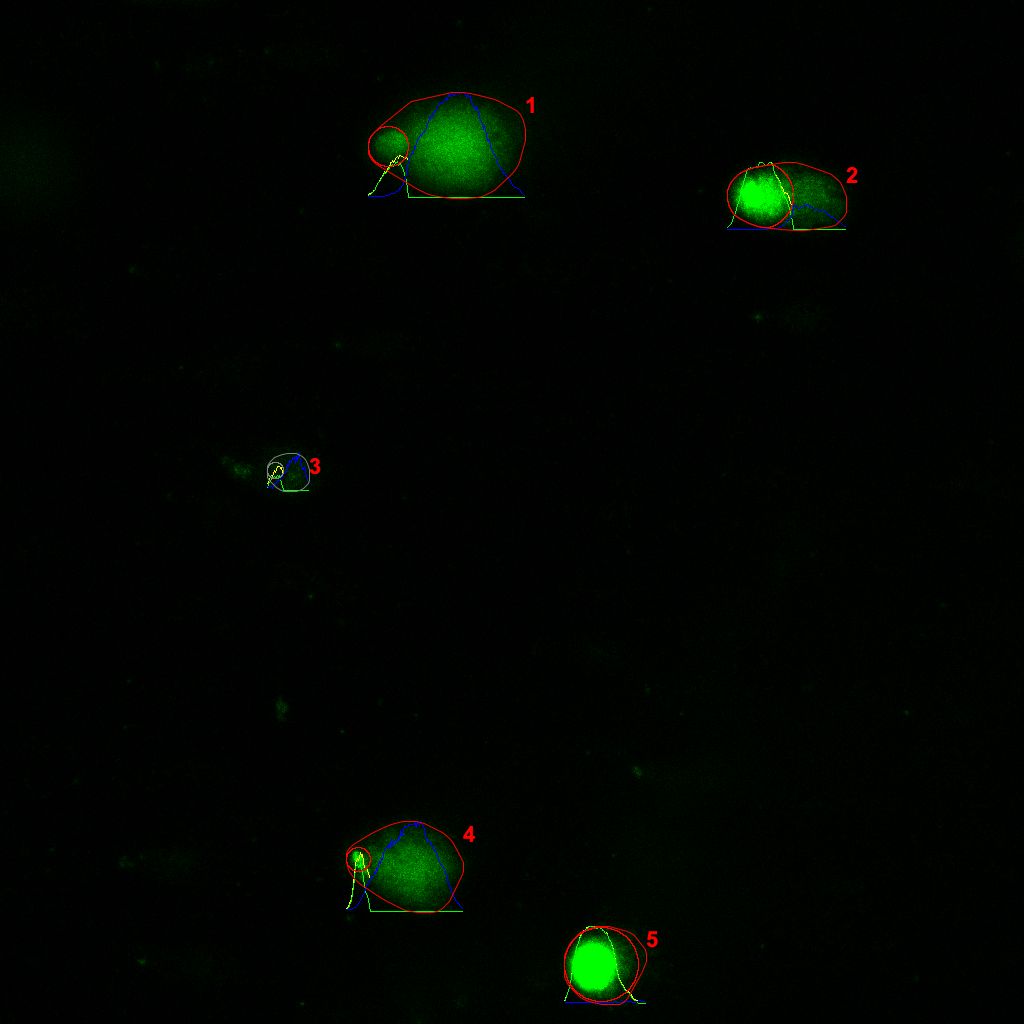

Supplement: Supplementary file 13 — Source data Fig. 6 [file 44321_2026_393_MOESM13_ESM.zip › Figure 6/6B/220915 Comet assay alkaline/output etoposide 2/33_Etoposide_4h_10x_Ccenter15_1AUall_rep2_Maximum.ome.tif_out.tif]

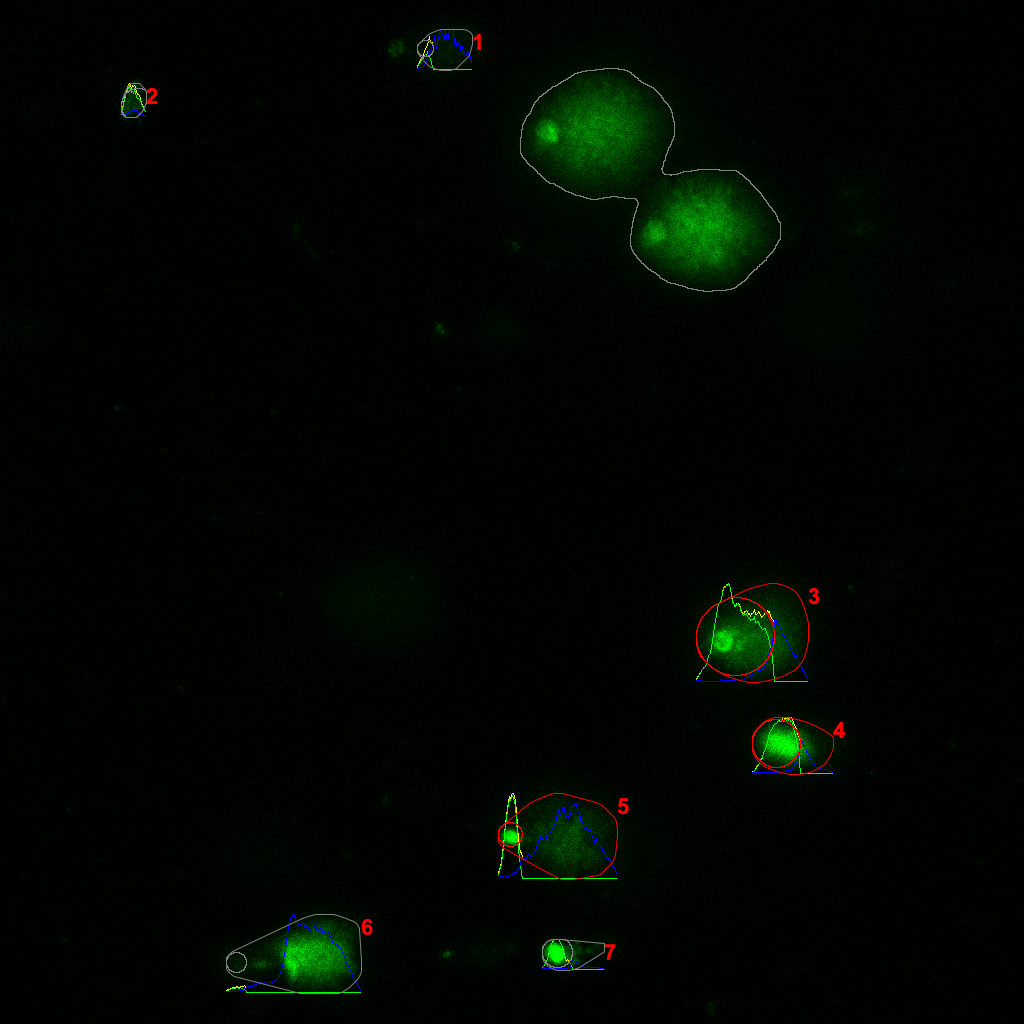

Supplement: Supplementary file 13 — Source data Fig. 6 [file 44321_2026_393_MOESM13_ESM.zip › Figure 6/6B/220915 Comet assay alkaline/output etoposide 2/36_Etoposide_4h_10x_Ccenter15_1AUall_rep2_Maximum.ome.tif_out.tif]

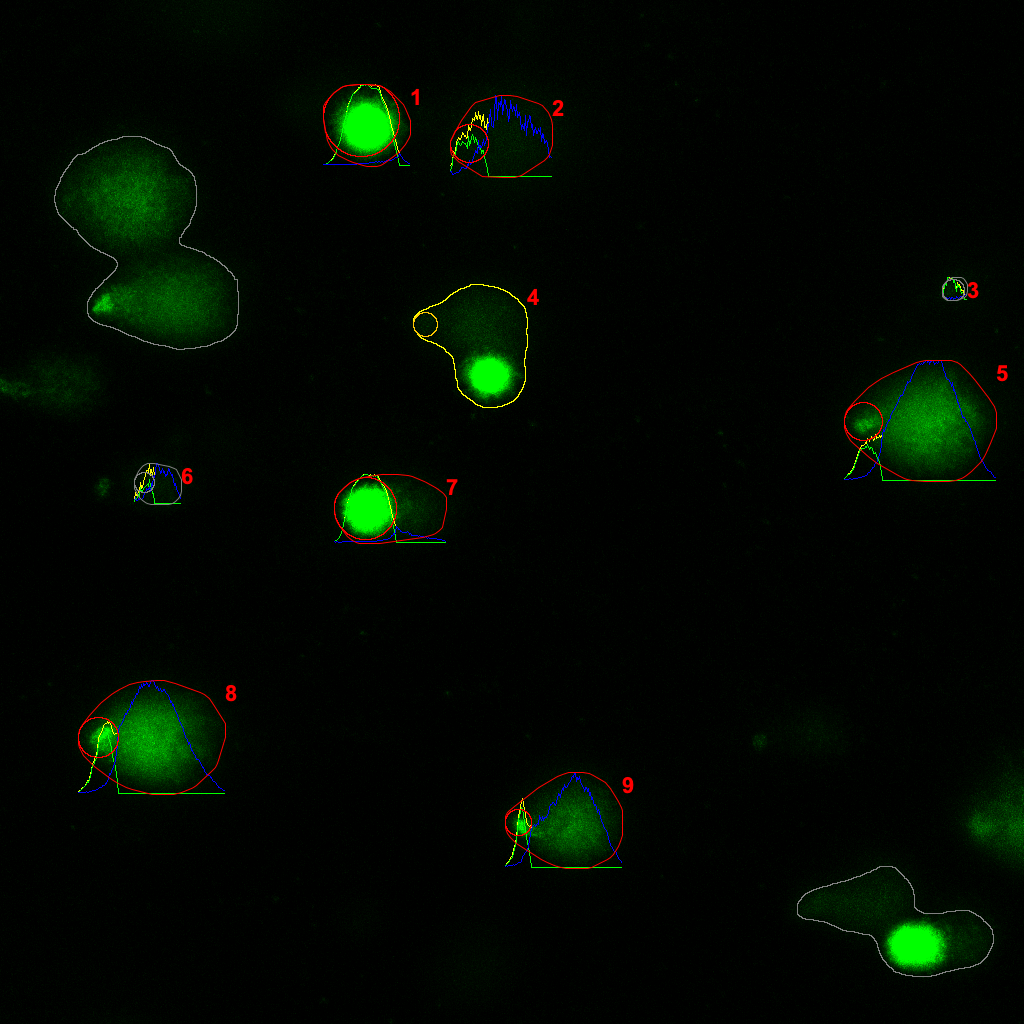

Supplement: Supplementary file 13 — Source data Fig. 6 [file 44321_2026_393_MOESM13_ESM.zip › Figure 6/6B/220915 Comet assay alkaline/output etoposide 2/40_Etoposide_4h_10x_Ccenter15_1AUall_rep2_Maximum.ome.tif_out.tif]

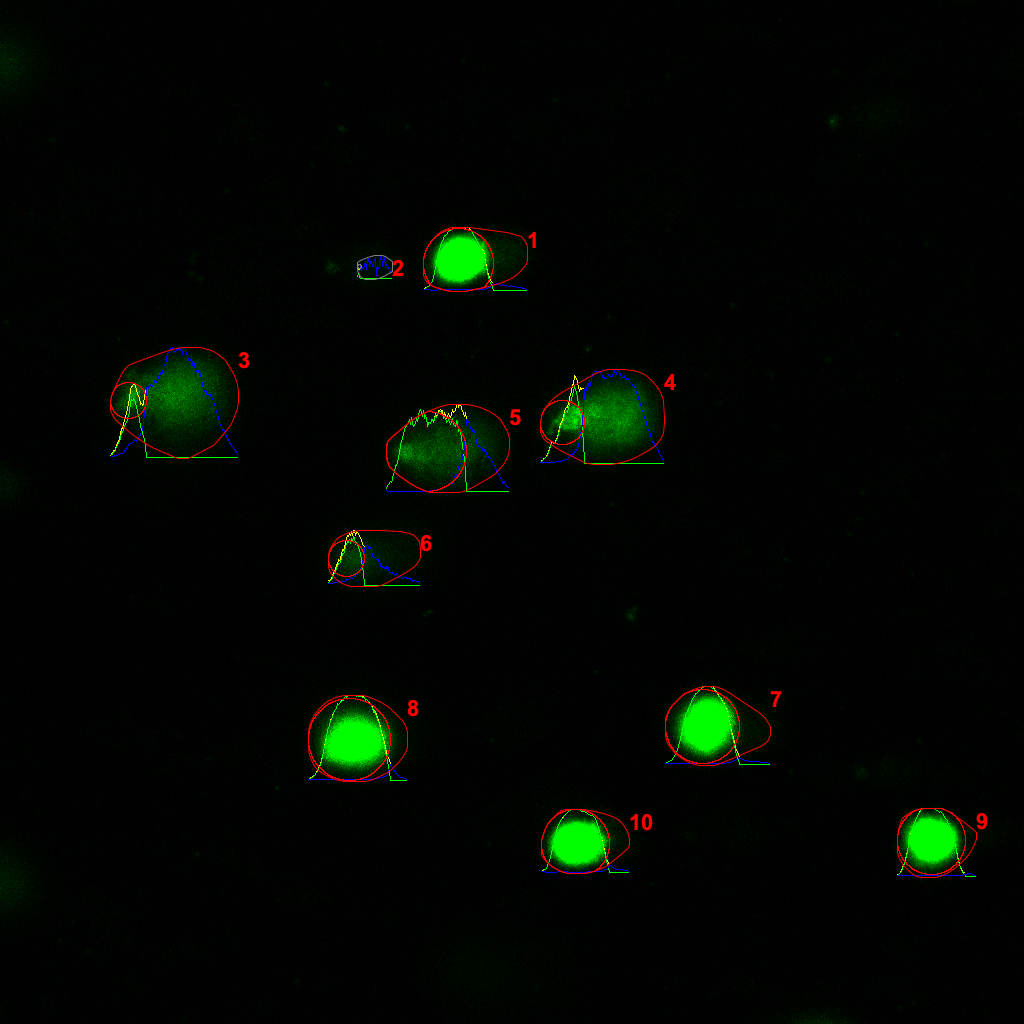

Supplement: Supplementary file 13 — Source data Fig. 6 [file 44321_2026_393_MOESM13_ESM.zip › Figure 6/6B/220915 Comet assay alkaline/output etoposide 2/38_Etoposide_4h_10x_Ccenter15_1AUall_rep2_Maximum.ome.tif_out.tif]
