# Supplementary material for: CDK12/CDK13 inhibition disrupts transcriptional elongation and replication fork progression in glioblastoma
Source: EMBO Mol Med. 2026 Mar 25;18(5):1592–624. doi: 10.1038/s44321-026-00393-w (PMC13179391; doi:10.1038/s44321-026-00393-w)
Supplement: Supplementary file 14 — Expanded View Figures [file 44321_2026_393_MOESM14_ESM.pdf]

Expanded View Figures

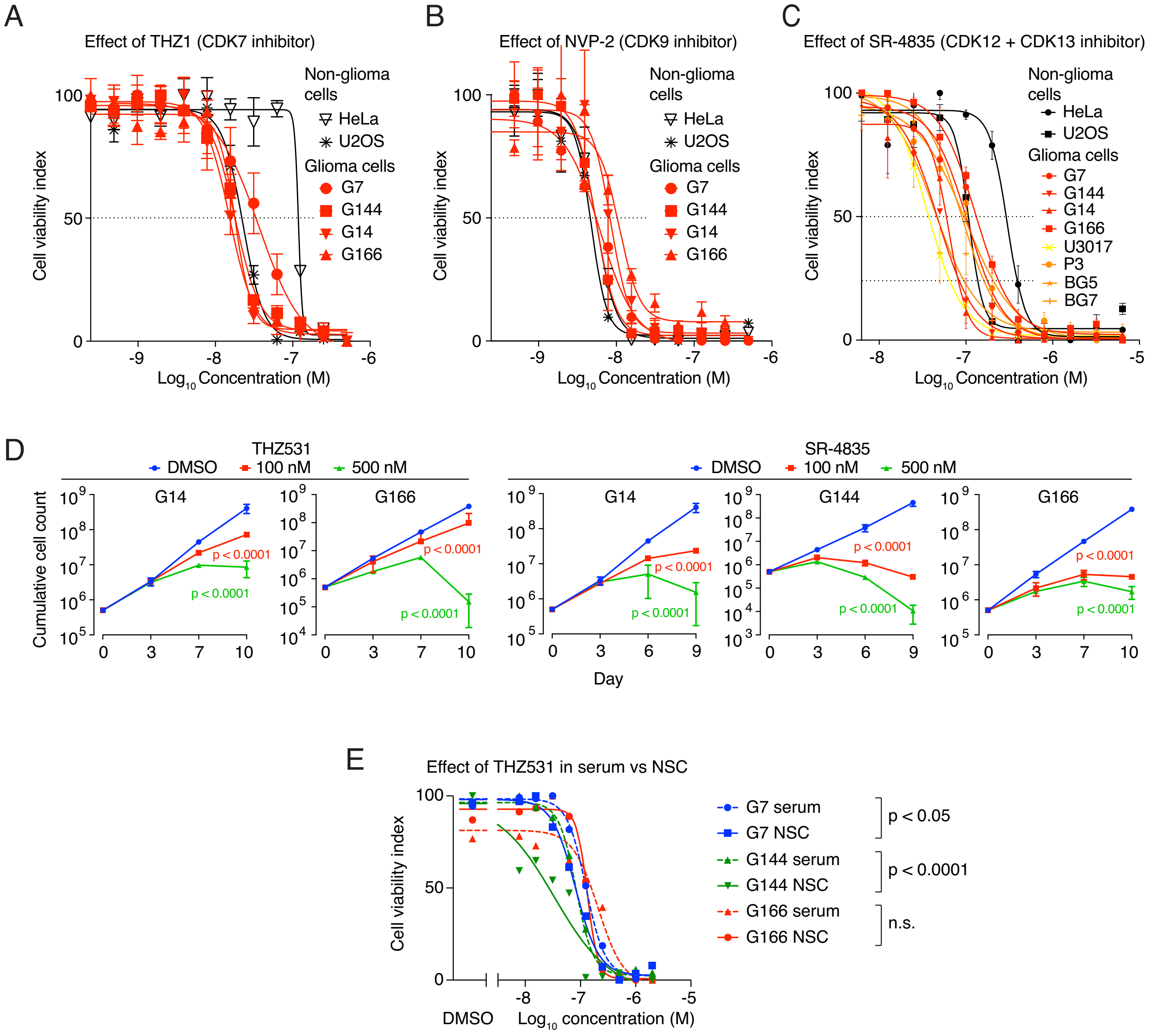

Figure EV1. Effect of tCDK inhibition on GSC viability and proliferation.

(**A**) Four high grade glioblastoma cell lines and two non-glioblastoma cell lines were treated with increasing doses of THZ1. After 72 h, cells were subjected to an MTT assay. The graph displays a dose-response curve with percent cell viability relative to the DMSO control for each cell line. Data represent mean ± SD of three replicates. (**B**) Four high-grade glioblastoma cell lines and two non-glioblastoma cell lines were treated with increasing doses of NVP-2. After 72 h, cells were subjected to an MTT assay. The graph displays a dose-response curve with percent cell viability relative to the DMSO control for each cell line. Data represent mean ± SD of three replicates. (**C**) Eight high-grade glioblastoma cell lines and two non-glioblastoma cell lines were treated with increasing doses of SR-4835. After 72 h, cells were subjected to an MTT assay. The graph displays a dose-response curve with percent cell viability relative to the DMSO control for each cell line. Data represent mean ± SD of three replicates. (**D**) In vitro cell proliferation assay of GSCs treated as indicated. Data represent mean ± SD of two replicates. Data were analyzed by two-way ANOVA followed by Tukey's multiple comparisons test, with significant differences as compared to the control. Exact p-values are provided in Appendix Table S4. (**E**) Three high-grade glioblastoma cell lines were cultured in serum-free or serum-containing media (with serum added immediately before the viability assay) and were treated with increasing doses of THZ531. After 72 h, the cell viability was assessed using CellTiter-Glo. The graph displays a dose-response curve with percent cell viability relative to the DMSO control for each cell line. Data represent mean ± SD of three replicates. Data were analyzed using two-way ANOVA followed by Tukey's multiple comparisons test. G7 serum vs. G7 NSC: $p = 0.010252731$; G144 serum vs. G144 NSC: $p = 9.56E\text{-}13$.

© The Author(s)

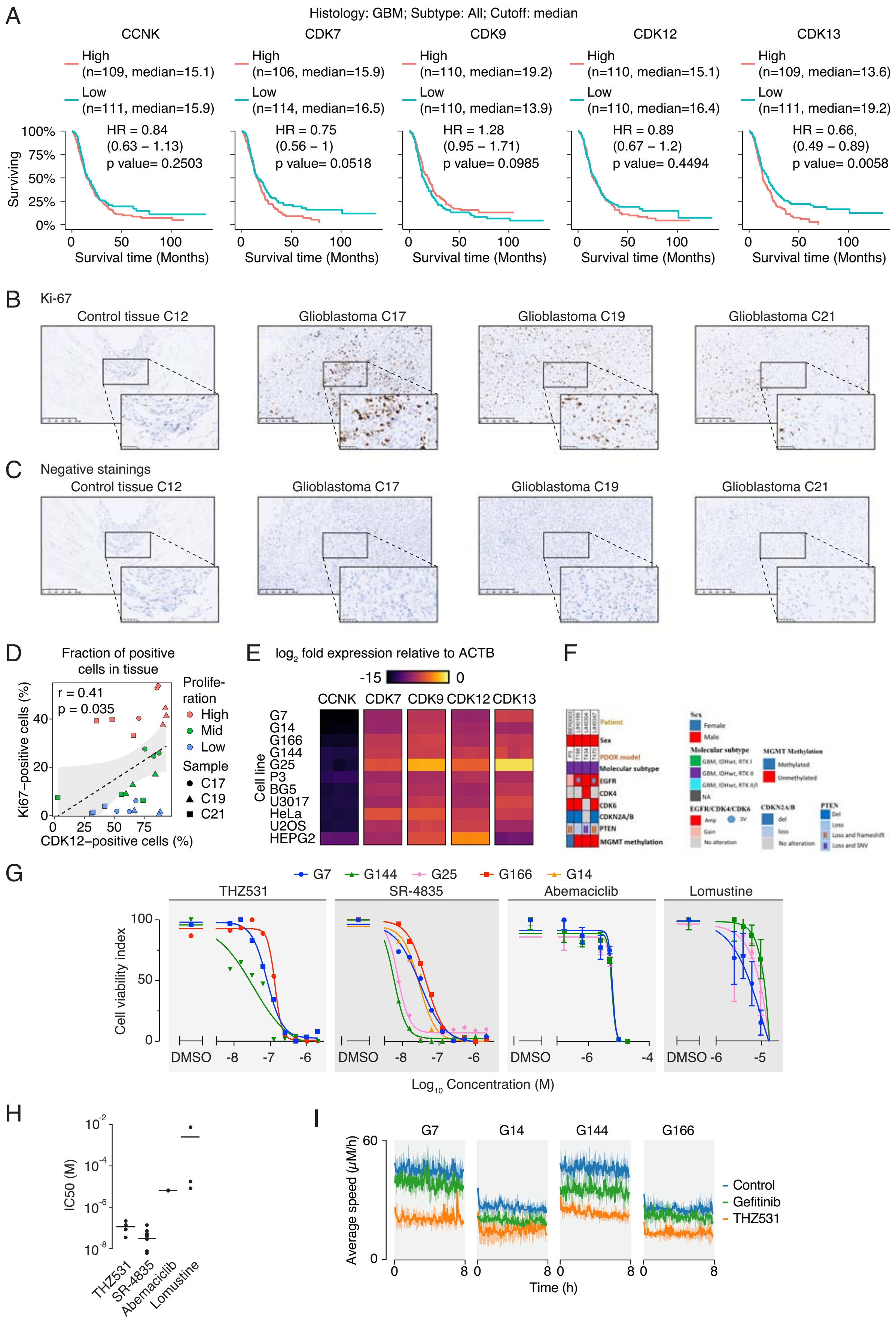
A
Histology: GBM; Subtype: All; Cutoff: median
CCNK
High (n=109, median=15.1)
Low (n=111, median=15.9)
HR = 0.84 (0.63 – 1.13) p value= 0.2503
CDK7
High (n=106, median=15.9)
Low (n=114, median=16.5)
HR = 0.75 (0.56 – 1) p value= 0.0518
CDK9
High (n=110, median=19.2)
Low (n=110, median=13.9)
HR = 1.28 (0.95 – 1.71) p value= 0.0985
CDK12
High (n=110, median=15.1)
Low (n=110, median=16.4)
HR = 0.89 (0.67 – 1.2) p value= 0.4494
CDK13
High (n=109, median=13.6)
Low (n=111, median=19.2)
HR = 0.66, (0.49 – 0.89) p value= 0.0058
Surviving
Survival time (Months)
B Ki-67
Control tissue C12
Glioblastoma C17
Glioblastoma C19
Glioblastoma C21
C Negative stainings
Control tissue C12
Glioblastoma C17
Glioblastoma C19
Glioblastoma C21
D Fraction of positive cells in tissue
r = 0.41
p = 0.035
Ki67–positive cells (%)
CDK12–positive cells (%)
Prolife-ration
High
Mid
Low
Sample
C17
C19
C21
E log2 fold expression relative to ACTB
-15 0
CCNK CDK7 CDK9 CDK12 CDK13
Cell line
G7
G14
G166
G144
G25
P3
BG5
U3017
HeLa
U2OS
HEPG2
F
G
G7 G144 G25 G166 G14
THZ531
SR-4835
Abemaciclib
Lomustine
Cell viability index
DMSO
Log10 Concentration (M)
H
IC50 (M)
THZ531
SR-4835
Abemaciclib
Lomustine
I
G7 G14 G144 G166
Average speed (μM/h)
Control
Gefitinib
THZ531
Time (h)

Figure EV2. tCDK expression correlates with glioblastoma patient survival, proliferation, and sensitivity to CDK12/CDK13 inhibition.

(**A**) Kaplan–Meier survival analyses generated using GlioVis showing the association between overall survival of glioblastoma patients and expression levels of tCDKs. Patients were stratified into high and low expression groups based on median gene expression. Statistical significance was assessed using the log-rank test. (**B**) Representative images of Ki-67 immunohistochemistry in cortex/infiltration zone/cell-rich tumor of glioblastoma patients. (**C**) Negative stainings for the patient samples used in the study. (**D**) Correlation analysis of CDK12-positive cells and proliferation status in patient tissue is shown in Figs. 2A and EV2B. One-way ANOVA was used to compare CDK12 expression across low, intermediate, and high proliferation categories. Correlation calculated with Spearman correlation, $r = 0.41$; $p = 0.035$. (**E**) Heatmap showing the mRNA expression of tCDKs in the cell lines used in the study. (**F**) Summary of the patient characteristics of the GBM organoid models. (**G**) Four high-grade glioblastoma cell lines were treated with increasing doses of inhibitors as indicated. After 72 h, the cell viability was assessed using CellTiter Glo. The graph displays a dose-response curve with percent cell viability relative to the DMSO control for each cell line. Data represent mean ± SD of three replicates. (**H**) Dot-plot showing IC50 values for dose response of inhibitors on a panel of GSCs shown in (**G**). (**I**) Effect of THZ531 treatment on the migration of glioblastoma cells. The average speed of migration is plotted over time. Gefitinib, an EGFR inhibitor is used a positive control.

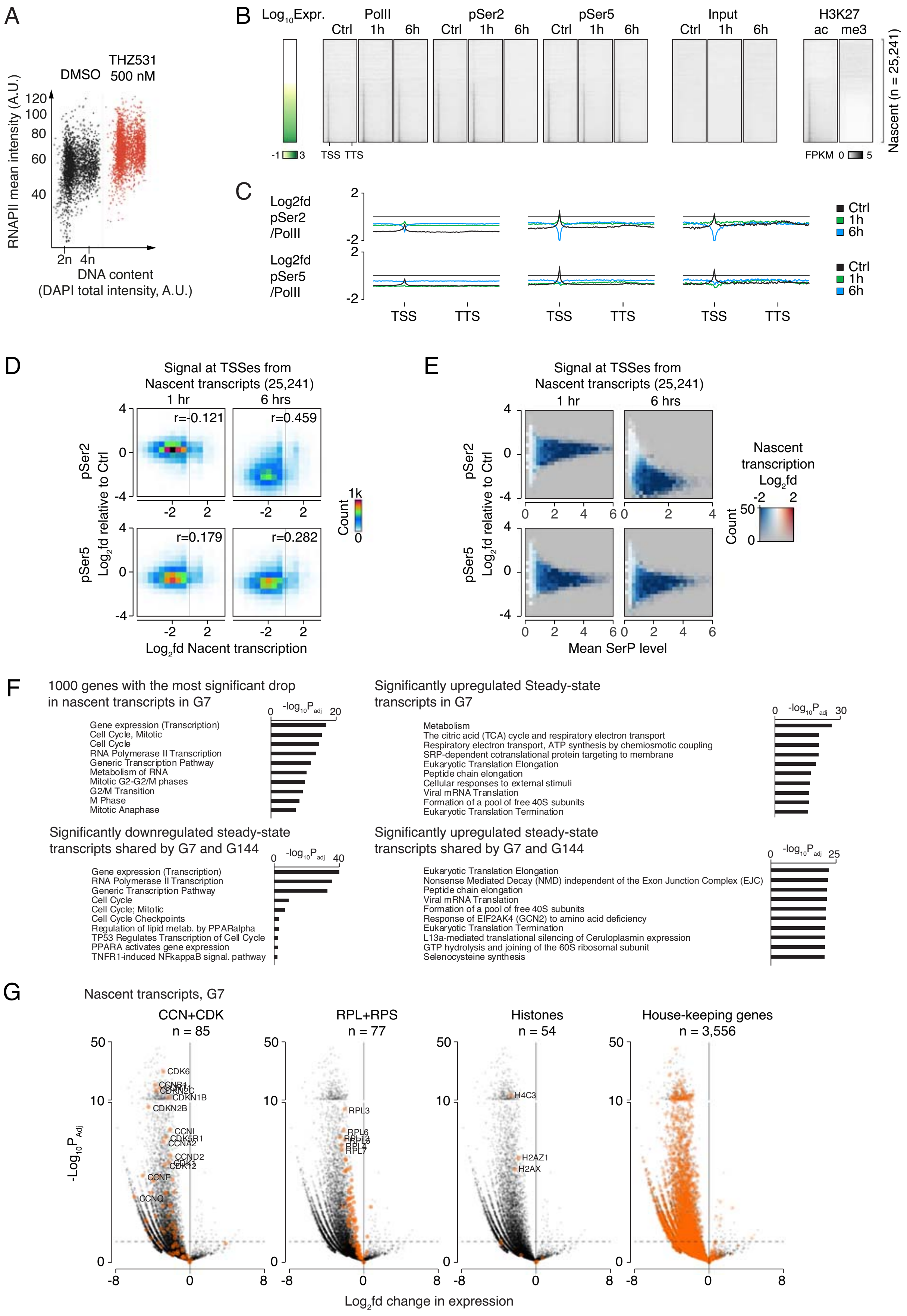
A
DMSO
THZ531 500 nM
RNAPII mean intensity (A.U.)
120
100
80
60
40
2n 4n
DNA content
(DAPI total intensity, A.U.)
B
Log10Expr.
PolII
pSer2
pSer5
Input
H3K27
Ctrl 1h 6h
ac me3
-1 3
TSS TTS
FPKM 0 5
Nascent (n = 25,241)
C
Log2fd pSer2 /PolII
Log2fd pSer5 /PolII
Ctrl
1h
6h
TSS TTS
D
Signal at TSSes from Nascent transcripts (25,241)
1 hr
6 hrs
r=-0.121
r=0.459
r=0.179
r=0.282
pSer2
pSer5
Log2fd relative to Ctrl
Count
1k
0
Log2fd Nascent transcription
E
Signal at TSSes from Nascent transcripts (25,241)
1 hr
6 hrs
Nascent transcription Log2fd
Count
Mean SerP level
F
1000 genes with the most significant drop in nascent transcripts in G7
-log10Padj
Gene expression (Transcription)
Cell Cycle, Mitotic
Cell Cycle
RNA Polymerase II Transcription
Generic Transcription Pathway
Metabolism of RNA
Mitotic G2-G2/M phases
G2/M Transition
M Phase
Mitotic Anaphase
Significantly upregulated Steady-state transcripts in G7
Metabolism
The citric acid (TCA) cycle and respiratory electron transport
Respiratory electron transport, ATP synthesis by chemiosmotic coupling
SRP-dependent cotranslational protein targeting to membrane
Eukaryotic Translation Elongation
Peptide chain elongation
Cellular responses to external stimuli
Viral mRNA Translation
Formation of a pool of free 40S subunits
Eukaryotic Translation Termination
Significantly downregulated steady-state transcripts shared by G7 and G144
Gene expression (Transcription)
RNA Polymerase II Transcription
Generic Transcription Pathway
Cell Cycle
Cell Cycle; Mitotic
Cell Cycle Checkpoints
Regulation of lipid metab. by PPARalpha
TP53 Regulates Transcription of Cell Cycle
PPARA activates gene expression
TNFR1-induced NFkappaB signal. pathway
Significantly upregulated steady-state transcripts shared by G7 and G144
Eukaryotic Translation Elongation
Nonsense Mediated Decay (NMD) independent of the Exon Junction Complex (EJC)
Peptide chain elongation
Viral mRNA Translation
Formation of a pool of free 40S subunits
Response of EIF2AK4 (GCN2) to amino acid deficiency
Eukaryotic Translation Termination
L13a-mediated translational silencing of Ceruloplasmin expression
GTP hydrolysis and joining of the 60S ribosomal subunit
Selenocysteine synthesis
G
Nascent transcripts, G7
CCN+CDK n = 85
RPL+RPS n = 77
Histones n = 54
House-keeping genes n = 3,556
-Log10PAdj
CDK6
CDKN1B
CDKN2B
CCNI
CCNA2
CCND2
CCNF
CCNO
RPL3
H4C3
H2AZ1
H2AX
Log2fd change in expression

Figure EV3. Effect of CDK12/CDK13 inhibition on genome wide RNAPII phosphorylation and mRNA synthesis in GSCs.

(**A**) QIBC analysis of pSer2 for G7 cells treated with vehicle or 500 nM THZ531 for 6 h. (**B**) Heatmaps of Cut&Run signal from RNAPII, RNAPII phosphorylation states, selected histone mark modifications, as well as input at gene bodies and immediate upstream and downstream regions (±25% of gene length) from G7 cells treated with THZ531 for either 1 or 6 h, and DMSO controls. Genes were ordered vertically based on their total expression level. The horizontal extent of each gene and the upstream and downstream regions corresponding to a quarter of the gene length is fitted within the same visual space in the heatmaps, regardless of its absolute extent. TSS and TTS illustrate transcription start sites and termination sites, respectively. Cut&Run and input levels are FPKM normalized. (**C**) Graphs of average Cut&Run signal from RNAPII phosphorylation states normalized to RNAPII levels at all gene bodies and surrounding loci. RNAPII and RNAPII modification states were obtained as described in Fig. 4A. The horizontal extent of each gene and the upstream and downstream regions corresponding to half a gene length is fitted within the same visual space in the heatmaps regardless of its absolute extent. TSS and TTS illustrate transcription start sites and termination sites, respectively. Cut&Run levels are FPKM normalized. (**D**) 2D-histograms showing the relationship between changes in nascent transcription (X-axis) and pSer2 (top) or pSer5 (bottom) at TSSs after 1 h (left) and 6 h (right). (**E**) Colored MA-plots showing the combined relationship between changes pSer2 (top) or pSer5 (bottom) at TSSs after 1 h (left) and 6 h (right) colored according to changes in nascent transcript levels. (**F**) Bar diagrams showing the most significantly enriched gene ontology (GO) terms from selected subsets of genes from steady-state and nascent RNA. X-axes represent −log10 *p* values from Fisher's one-tailed tests adjusted for multiple testing using g:Profiler's default g:SCS algorithm. (**G**) Volcano plots showing the overall transcriptional differences in nascent transcripts, as in Fig. 4L, but with certain gene populations highlighted. X-axes show the log2 fold difference in transcription in G7 cells treated with THZ531 for 6 h compared to DMSO controls. Y-axes show the −log10 transformed *p* values from two-tailed Wald tests Benjamini–Hochberg corrected for multiple testing. Colored dots illustrate the transcriptional changes of the listed gene populations.

© The Author(s)

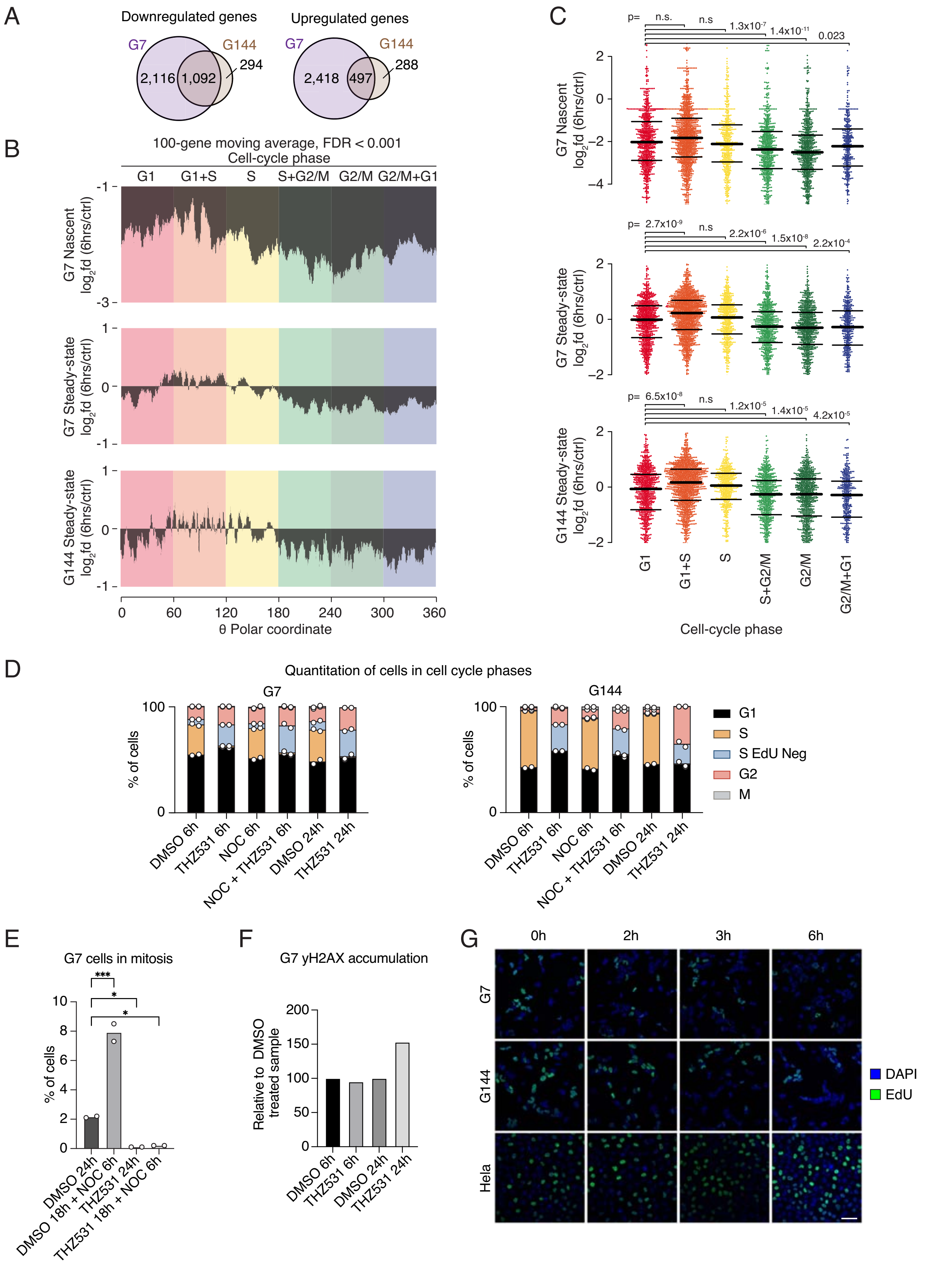
A
Downregulated genes
G7
G144
2,116
1,092
294
Upregulated genes
G7
G144
2,418
497
288
B
100-gene moving average, FDR < 0.001
Cell-cycle phase
G1
G1+S
S
S+G2/M
G2/M
G2/M+G1
G7 Nascent log2fd (6hrs/ctrl)
G7 Steady-state log2fd (6hrs/ctrl)
G144 Steady-state log2fd (6hrs/ctrl)
θ Polar coordinate
C
G7 Nascent log2fd (6hrs/ctrl)
G7 Steady-state log2fd (6hrs/ctrl)
G144 Steady-state log2fd (6hrs/ctrl)
p= n.s. n.s 1.3x10-7 1.4x10-11 0.023
p= 2.7x10-9 n.s 2.2x10-6 1.5x10-8 2.2x10-4
p= 6.5x10-8 n.s 1.2x10-5 1.4x10-5 4.2x10-5
G1
G1+S
S
S+G2/M
G2/M
G2/M+G1
Cell-cycle phase
D
Quantitation of cells in cell cycle phases
G7
G144
% of cells
DMSO 6h
THZ531 6h
NOC 6h
NOC + THZ531 6h
DMSO 24h
THZ531 24h
G1
S
S EdU Neg
G2
M
E
G7 cells in mitosis
% of cells
DMSO 24h
DMSO 18h + NOC 6h
THZ531 24h
THZ531 18h + NOC 6h
F
G7 yH2AX accumulation
Relative to DMSO treated sample
DMSO 6h
THZ531 6h
DMSO 24h
THZ531 24h
G
0h
2h
3h
6h
G7
G144
Hela
DAPI
EdU

© The Author(s)

Figure EV4. Effect of CDK12/CDK13 inhibition on GSC cell cycle.

(**A**) Venn diagrams illustrating the overlap in the populations of genes being downregulated (left) as well as upregulated (right) in G7 cells compared to G144 cells. (**B**) Graphs illustrates the moving average of transcriptional changes in nascent (top) or steady-state (middle, bottom) transcripts from G7 (top, middle) or G144 (bottom) cells treated with THZ531 for 6 h compared to DMSO controls. Transcripts were ordered according to the previously published classification of cell-cycle timing. Only transcripts, for which transcriptional timing could be assessed with an FDR-value of 0.001 or better, were included. (**C**) Beeswarm plots of transcriptional changes as in (**B**), with transcripts grouped into six overall groups based on previously published transcriptional timing. *P* values were obtained using Mann–Whitney *U*-tests and Bonferroni-corrected for multiple testing. (**D**) Bar diagrams of cell cycle distributions of G7 and G144 cells treated as indicated. Doses used: THZ531 (500 nM), Nocodazole (1 µg/mL). (**E**) Flow cytometry analysis of % of G7 cells in mitosis following 500 nM THZ531 ± 1 µg/mL Nocodazole treatment for 24 h. Data were analyzed by one-way ANOVA followed by Dunnett's post hoc test *$p < 0.05$, ***$p < 0.001$. Significant differences as compared to the control: DMSO 18 h + NOC 6 h: $p = 0.0004$; THZ531 24 h: $p = 0.0194$; THZ531 18 h + NOC 6 h: $p = 0.0231$. Data represent mean ± SD of two replicates for (**D**, **E**). (**F**) Flow cytometric assays of γH2AX accumulation in G7 cells treated with 500 nM THZ531 for 6 and 24 h. (**G**) Cells were treated with 500 nM THZ531 at indicated times, and EdU incorporation was performed in the last 1 h by adding 10 µM EdU. Cell staining was done using Click-IT chemistry according to the manufacturer's instructions. Scale bar: ~50 µm (approximated based on a comparable image acquired using the same imaging setup).

© The Author(s)

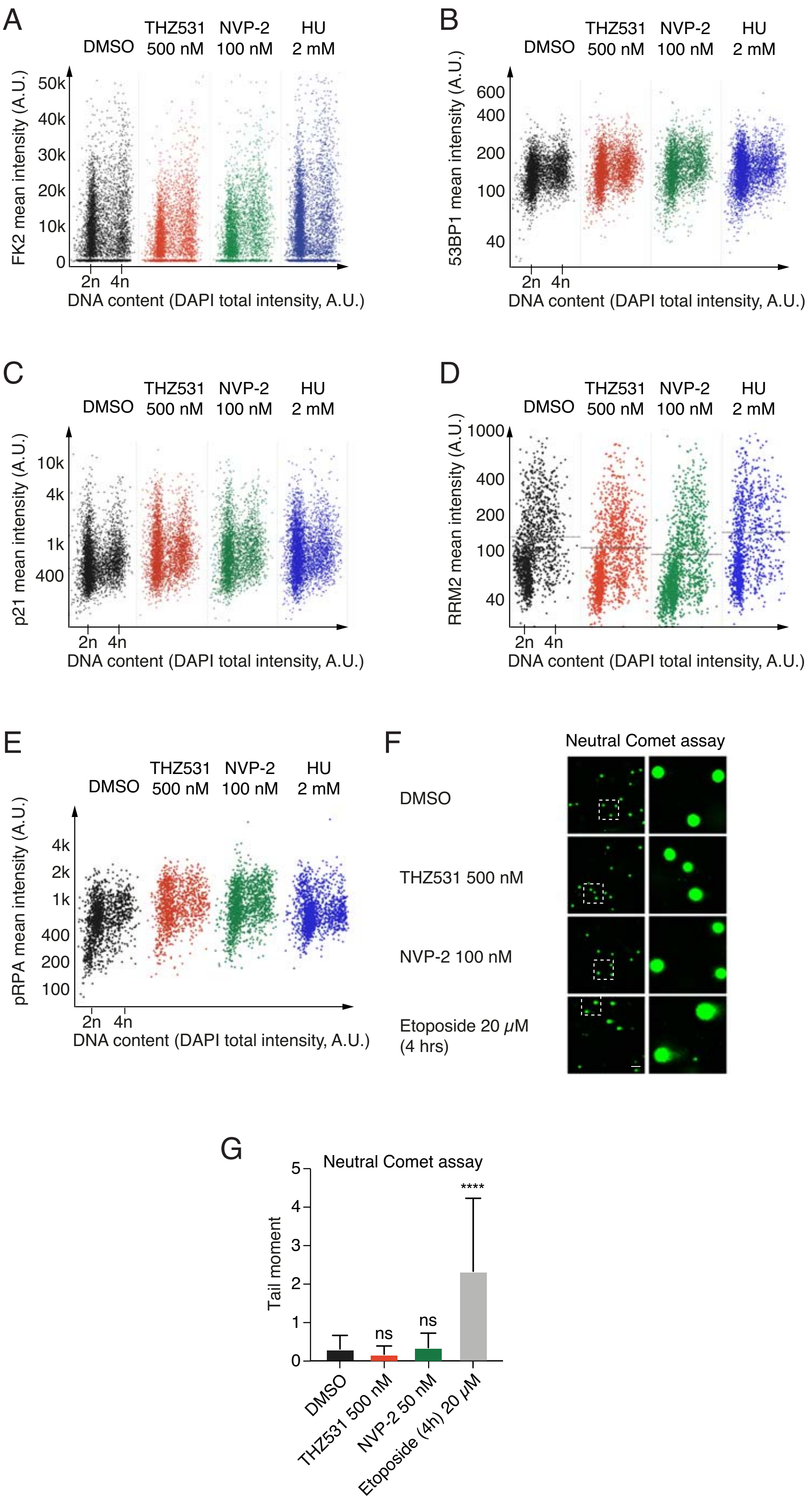
A
DMSO
THZ531 500 nM
NVP-2 100 nM
HU 2 mM
FK2 mean intensity (A.U.)
50k
40k
30k
20k
10k
0
2n 4n
DNA content (DAPI total intensity, A.U.)
B
DMSO
THZ531 500 nM
NVP-2 100 nM
HU 2 mM
53BP1 mean intensity (A.U.)
600
400
200
100
40
2n 4n
DNA content (DAPI total intensity, A.U.)
C
DMSO
THZ531 500 nM
NVP-2 100 nM
HU 2 mM
p21 mean intensity (A.U.)
10k
4k
1k
400
2n 4n
DNA content (DAPI total intensity, A.U.)
D
DMSO
THZ531 500 nM
NVP-2 100 nM
HU 2 mM
RRM2 mean intensity (A.U.)
1000
400
200
100
40
2n 4n
DNA content (DAPI total intensity, A.U.)
E
DMSO
THZ531 500 nM
NVP-2 100 nM
HU 2 mM
pRPA mean intensity (A.U.)
4k
2k
1k
400
200
100
2n 4n
DNA content (DAPI total intensity, A.U.)
F
Neutral Comet assay
DMSO
THZ531 500 nM
NVP-2 100 nM
Etoposide 20 μM
(4 hrs)
G
Neutral Comet assay
Tail moment
5
4
3
2
1
0
ns
ns
****
DMSO
THZ531 500 nM
NVP-2 50 nM
Etoposide (4h) 20 μM

 © The Author(s)

Figure EV5. Effect of CDK12/CDK13 inhibition on markers of replication stress and DDR in GSCs.

(**A–E**) QIBC analyses of (**A**) FK2, (**B**) 53BP1, (**C**) p21, (**D**) RRM2, and (**E**) pRPA expression (Y-axis) relative to DNA content (X-axis) in G7 cells under vehicle control, 500 nM THZ531 treatment, and control treatments with 100 nM NVP-2 and 2 mM HU. (**F**) Neutral comet assays showing tail movements depicting DNA damage in G7 cells for vehicle control, 500 nM THZ531 treatment, and control treatments with 50 nM NVP-2 and 20 µM Etoposide. Scale bar: 100 µm. (**G**) Quantification of the tail moments. Approximately 100 cells were measured per sample. Data were presented as mean ± SD of duplicates from three biological replicates. Statistical analysis by one-way ANOVA with Kruskal–Wallis test and Dunn's multiple comparison correction, significant differences as compared to the control: Etoposide (4 h) 20 µM: <0.000000000000001. *$p < 0.05$, **$p < 0.01$, ***$p < 0.001$. ****$p < 0.0001$.
